# Evaluation of the Web-Based OutsidePlay-ECE Intervention to Influence Early Childhood Educators’ Attitudes and Supportive Behaviors Toward Outdoor Play: Randomized Controlled Trial

**DOI:** 10.2196/36826

**Published:** 2022-06-10

**Authors:** Mariana Brussoni, Christina S Han, Yingyi Lin, John Jacob, Fritha Munday, Megan Zeni, Melanie Walters, Eva Oberle

**Affiliations:** 1 Department of Pediatrics Faculty of Medicine University of British Columbia Vancouver, BC Canada; 2 British Columbia Injury Research & Prevention Unit British Columbia Children’s Hospital Research Institute Vancouver, BC Canada; 3 Human Early Learning Partnership School of Population and Public Health University of British Columbia Vancouver, BC Canada; 4 BC Children’s Hospital Research Institute Vancouver, BC Canada; 5 School of Population and Public Health University of British Columbia Vancouver, BC Canada; 6 Spatial Sciences Institute University of Southern California Los Angeles, CA United States; 7 Faculty of Education University of British Columbia Vancouver, BC Canada; 8 Child Care Services University of British Columbia Vancouver, BC Canada

**Keywords:** risk perception, risky play, risk reframing, early childhood education, tolerance of risk in play, child care, outside play, preschool

## Abstract

**Background:**

Outdoor play is critical to children’s healthy development and well-being. Early learning and childcare centers (ELCCs) are important venues for increasing children’s outdoor play opportunities, and early childhood educators’ (ECE) perception of outdoor play can be a major barrier to outdoor play. The OutsidePlay-ECE risk-reframing intervention is a fully automated and open access web-based intervention to reframe ECEs’ perceptions of the importance of outdoor play and risk in play and to promote a change in their practice in supporting it in ELCC settings. We grounded the intervention in social cognitive theory and behavior change techniques.

**Objective:**

The aim of this study is to evaluate the effectiveness of the OutsidePlay-ECE web-based risk-reframing intervention.

**Methods:**

We conducted a single-blind randomized controlled trial in Canada between December 2020 and June 2021 to test the OutsidePlay-ECE risk-reframing intervention for ECEs. We recruited participants using social media and mass emails through our partner and professional networks. We invited ECEs and administrators working in an ELCC, who can speak, read, and understand English. We randomized consented participants to the intervention or control condition. The participants allocated to the intervention condition received a link to the OutsidePlay-ECE intervention. Participants allocated to the control condition read the Position Statement on Active Outdoor Play, a 4-page document on research and recommendations for action in addressing barriers to outdoor play. The primary outcome was a change in tolerance of risk in play. The secondary outcome was goal attainment. We collected data on the web via REDCap (Vanderbilt University) at baseline and 1 week and 3 months after intervention.

**Results:**

A total of 563 participants completed the baseline survey, which assessed their demographics and tolerance of risk in play. They were then randomized: 281 (49.9%) to the intervention and 282 (50.1%) to the control condition. Of these, 136 (48.4%) and 220 (78%) participants completed the baseline requirements for the intervention and control conditions, respectively. At 1 week after intervention, 126 (44.8%) and 209 (74.1%) participants completed follow-up assessments, respectively, and at 3 months after intervention, 119 (42.3%) and 195 (69.1%) participants completed the assessments, respectively. Compared with participants in the control condition, participants in the intervention group had significantly higher tolerance of risk in play at 1 week (*β=*.320; *P*=.001) and 3 months after intervention (*β=*.251; *P*=.009). Intention-to-treat analyses replicated these findings (*β=*.335; *P*<.001 and *β=*.271; *P*=.004, respectively). No significant intervention effect was found for goal attainment outcomes (odds ratio 1.124, 95% CI 0.335-3.774; *P*=.85).

**Conclusions:**

The results of this randomized controlled trial demonstrated that the OutsidePlay-ECE intervention was effective and had a sustained effect in increasing ECEs’ and administrators’ tolerance of risk in play. It was not effective in increasing goal attainment.

**Trial Registration:**

ClinicalTrials.gov NCT04624932; https://clinicaltrials.gov/ct2/show/NCT04624932

**International Registered Report Identifier (IRRID):**

RR2-10.2196/31041

## Introduction

### Background

Children’s opportunities for outdoor play have been decreasing over successive generations in many developed countries [1,2]. This decline is concerning because outdoor play is integral to children’s physical and mental health [3,4]. The literature consistently illustrates that children who engage in outdoor play more often demonstrate increased physical activity [5-8], which has subsequent benefits for their physical health (eg, lower blood pressure, lower BMI, lower obesity, and healthy development of bone mineral density) [9-11]. In addition, outdoor play increases children’s social competency and self-esteem [12,13].

Over the past few decades, several factors have been proposed to explain the overall decline in children’s outdoor play. Increasingly, risk-averse cultural norms have resulted in ubiquitous adult supervision and playground equipment that offer little challenge [1,14-16]. This intersects with 21st century parenting norms. Influenced by anxieties about children’s educational attainment and safety, risk-averse caregiving prioritizes children’s achievement at the expense of play and encourages the heavy surveillance of children’s activities [14,16-19].

To address these concerns and influence parents’ perception of the importance of outdoor play, reduce their fears regarding the risks taken in play, and help them develop supportive parenting behaviors toward outdoor play, we built the OutsidePlay web-based risk-reframing intervention [20], where we first developed a module for parents (OutsidePlay-Parents). We found that this intervention was effective in changing the tolerance of risk in play among mothers of children aged 6 to 10 years [21]. Building on this work, we developed a new module for early childhood educators (ECEs; OutsidePlay-ECE) on the OutsidePlay intervention. Similar to the OutsidePlay-Parents module, the new module for the ECE uses social cognitive theory and behavior change techniques to address ECEs’ attitudes and behaviors toward outdoor play and its inherent risks [22,23]. For example, self-reflection questions highlighted incompatible beliefs to help participants think differently about assumed barriers (eg, the benefit participants obtained from the risks they took in play as a child vs their focus on limiting risk for the children in their care). A full description of the intervention mapping approach we followed in its development has been previously published, and details of the intervention components are provided in the OutsidePlay-ECE Intervention section in Methods [24]. The OutsidePlay-ECE intervention is a fully automated and open access web-based intervention. The intervention mobilizes evidence-based behavior change techniques underpinned in social cognitive theory to change ECE’s perception of outdoor play and practices and to facilitate behavior change by setting attainable goals in support of children’s outdoor play in early learning childcare center (ELCC) settings [24].

For many children, most of their waking hours are spent in an ELCC, which can be an invaluable opportunity to provide them with high-quality opportunities for outdoor play, particularly for children who may have limited access to outdoor play in their home environments [25,26]. Unfortunately, this opportunity has not been fully leveraged because of various limiting factors. For instance, amid societal risk aversion trends, ECEs face many actual and perceived barriers that are primarily linked to safety concerns [27,28]. Canadian ELCCs require a license to operate and need to follow their provincial or territorial childcare licensing guidelines, which are often interpreted by ECEs in restrictive ways [27,28]. Furthermore, these barriers intersect with ECEs’ cultural backgrounds and the level of confidence, knowledge, and experience in promoting and accommodating children’s outdoor play, as well as support received (perceived and actual) from their colleagues and the ELCC administration [27-29].

### Objectives

The aim of this study was to report the results of a randomized controlled trial (RCT) evaluating the effectiveness of the OutsidePlay-ECE intervention in increasing ECEs’ and ELCC administrators’ tolerance of risk in play and attain a behavior change goal related to providing outdoor play opportunities for children in their ELCC. Given the positive RCT results that we obtained on the effectiveness of the OutsidePlay-Parent intervention previously developed for parents [20], we hypothesized that participants completing the intervention for ECEs would have significantly greater increase in tolerance of risk in play than those in the control condition at 1 week and 3 months after intervention. We also hypothesized that a greater proportion of ECEs in the intervention condition would attain their behavior change goal than those in the control condition at 1 week and 3 months after intervention.

## Methods

### Study Design

We used a single-blind (researchers and outcome assessors), 2-parallel condition RCT. We conducted this study between December 1, 2020, and June 30, 2021, in Canada and collected measures at baseline and at 1 week and 3 months after intervention. Our primary outcome was the change in tolerance of risk in play at either follow-up time point. The secondary outcome was the participants’ goal attainment at either follow-up time point. The details on the intervention’s theoretical framework, development, content, and the RCT study protocol can be found in the study by Brussoni et al [24]. We registered our RCT with the US National Institutes of Health Protocol Registration and Results System (ClinicalTrials.gov NCT04624932). We followed the CONSORT-EHEALTH (Consolidated Standards of Reporting Trials of Electronic and Mobile Health Applications and Online Telehealth) guidelines for reporting this trial [30] (Multimedia Appendix 1).

### Ethics Approval and Privacy

The RCT was approved by the University of British Columbia and Children’s and Women’s Health Center of British Columbia Research Ethics Board (H19-03644). We conducted the RCT (including the intervention) entirely on the web; thus, there was no human involvement, except when participants had inquiries and reported technical issues via email. The sole identifiable information collected was the participants’ email addresses, which were required for sending allocated study material, follow-up measures, and reminders. We did not export the participants’ email addresses for data analysis. We assigned each participant a study number that did not include identifiable personal information.

### Participant Recruitment

We recruited participants between December 1, 2020, and March 15, 2021, via social media posts on Facebook, Facebook advertisements, Twitter, and Instagram. We also circulated mass recruitment emails through partners and professional networks. Potential participants completed a web-based survey in REDCap (Research Electronic Data Capture; Vanderbilt University), an electronic data capture tool hosted by and stored in the British Columbia Children’s Hospital Research Institute server [31]. We included a complete description and procedure for the study in the web-based survey. This allowed participants to self-assess their eligibility and to consent on the web, with the capability of downloading the consent form, if they decided to participate in our study.

We temporarily halted participant recruitment and participation from December 18, 2020, to January 4, 2021, to accommodate the Christmas and New Year holidays, during which most ELCCs were closed. We made this decision to secure more valid participant responses to the goal attainment question in the 1 week after intervention follow-up time point, asking “Did you accomplish your goal?” which concerned their behavior in promoting children’s outdoor play, specifically in their ELCC. We posted a message on the REDCap enrollment survey informing participants of this interruption and the date that RCT recruitment would resume.

### Eligibility and Exclusion Criteria

Eligible participants were adult ECEs and ELCC administrators currently working in Canada, who could speak, read, and understand English. Given that ECEs work closely with, and are influenced by, ELCC administrators, we included both ECEs and ELCC administrators in this RCT. We did not have any exclusion criteria. We included participants deemed eligible according to the aforementioned eligibility criteria. As the RCT was conducted entirely on the web, computer and internet literacy was an implicit de facto eligibility criterion. Eligible and interested participants provided consent by downloading the consent form for review and selecting a checkbox to participate. We then invited the enrolled participants to complete the baseline survey, which included sociodemographic questions and a questionnaire that assessed participant tolerance of risk in play, and enter their email address.

### Randomization and Blinding

We automatically randomized the enrolled participants who completed the baseline survey in REDCap to 1 of the 2 conditions: intervention and control. The participants had an equal (50%) likelihood of being assigned to each condition. We generated the randomization schedule beforehand by the Sealed Envelope service (Sealed Envelope Ltd) using randomized permuted blocks of sizes 4, 6, and 8. We then transferred the list to REDCap. We concealed allocation to the researchers during participant assignment and data analysis. We sent participants a unique link to their materials upon completion of the baseline survey and randomization. The nature of the intervention did not permit blinding of the participants. They may have intuited which condition they were allocated to, based on the details of the 2 conditions provided in the consent form: intervention (eg, web-based intervention) and control (ie, a PDF document). In addition, there has always been a risk that 2 or more participants from the same ELCC participated in the study and have become exposed to a condition different from theirs by communicating with their peers. We believe this scenario would be unlikely given that we recruited participants across Canada, and as such, we did not implement any precaution.

### OutsidePlay-ECE Intervention

**Figure 1:**
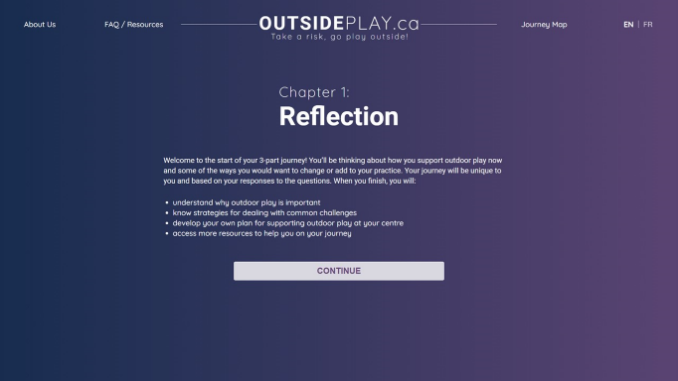
Screenshots of the OutsidePlay-ECE intervention landing page.

The goal of the OutsidePlay-ECE intervention is to reframe ECEs’ perception of the importance of outdoor play and its inherent risks and promote a change in their practice in supporting children’s outdoor play in ELCC settings. We designed OutsidePlay-ECE for ECE as a fully automated web-based risk-reframing intervention. It consists of 3 chapters, which are guided by the animated character of the first author (MB) and include self-reflection questions. We (the study authors) developed the OutsidePlay-ECE intervention following the intervention mapping process [32]. Social cognitive theory [33] provides a theoretical basis for the selection of behavior change techniques [22] adopted in the intervention. According to social cognitive theory, individuals are motivated to change behavior when their self-efficacy is high (eg, “I am capable of supporting more opportunities for outdoor play for children at my center”), are dissatisfied with their current state (eg, “I do not provide children at my center with enough opportunities for outdoor play”), and believe that changing their behavior will lead to the preferred outcome (eg, “Outdoor play will benefit children”) [32]. The OutsidePlay-ECE landing page is shown in Figure 1, and the complete screenshots of the OutsidePlay-ECE intervention are shown in Multimedia Appendix 2.

Our protocol paper [20] provides a full description of OutsidePlay-ECE intervention and details regarding its development. In brief, this intervention consists of 3 chapters, guiding participants through a journey. Chapter 1 prepares participants to position themselves on why they want to promote children’s outdoor play at their center by reflecting on their own childhood play and considering how children play in their center. Chapter 2 includes a series of videos presenting common challenging scenarios: (1) communicating with parents and caregivers, (2) rough-and-tumble play, (3) play at heights, (4) conflict resolution, (5) play with loose parts, and (6) play at speed. These scenarios evolved based on the 6 categories of risky play [34] and were then selected based on ECE feedback. For each scenario, the ECE must decide regarding what they allow children to do. For example, in the rough-and-tumble play scenario, 2 children start sword fighting with sticks, and the ECE is asked to decide whether to stop the children or talk to them about consent and safety. On the basis of the ECE’s choice, the video continues to show the outcome of the ECE’s decision. Each scenario includes a summary video by an experienced ECE, highlighting the main take-home messages of that scenario. The objective of Chapter 2 is to reflect on the barriers and challenges ECEs often encounter at their center while accommodating or promoting children’s outdoor play and provide them with clear actions to address them. The last chapter summarizes the learning in the previous 2 chapters and invites participants to think of an achievable goal and create a plan to accomplish it. Their journey, including their goals and plans, can be downloaded or sent to their email.

The OutsidePlay-ECE intervention focused on social cognitive theory constructs: outcome expectation; knowledge, barriers and opportunities; observational learning; self-efficacy; behavioral skills; and intentions [20]. The behavior change techniques related to information about consequences (health, social, and emotional), social comparison, framing and reframing and incompatible beliefs, problem solving, instructions on how to perform the behavior, demonstration of the behavior, social comparison, comparative imagining of future outcomes and goal setting (behavior and outcome), problem solving, action planning, and credible sources [20]. Details regarding the constructs and their associated behavior change techniques are outlined in our protocol paper [20].

We soft launched the OutsidePlay-ECE intervention on December 1, 2020, for the RCT and froze the content during the RCT (ie, we did not make any changes) and analysis. This means that we released the intervention only for RCT purposes with no publicity push before its full launch to the public. The participants were allocated to the intervention condition with a link to the OutsidePlay-ECE intervention. It could be completed in up to 100 minutes, depending on the participants’ movements through each chapter. Participants could also return to the intervention at their convenience, picking up from where they left off previously, provided that they did not delete their browser cache and http cookies. REDCap sent out a maximum of 3 automated reminders at 24, 48, and 60 hours after completion of the baseline survey and at the 1 week and 3 months after intervention follow-ups.

### Comparison Condition

We asked participants in the control condition to review a PDF of the Position Statement on Active Outdoor Play, a 4-page document with information on research and recommendations for action in addressing barriers to outdoor play [4,35]. We estimated that the participants took 15 to 20 minutes to read through the document. We did not send out automated reminders to participants in the control condition because once they opened the Position Statement on Active Outdoor Play, and closed their survey at that point, we considered that they completed the baseline requirement. However, we reminded them up to 3 times (24, 48, and 72 hours), if they did not finish the survey measures at any follow-up time point (ie, 1 week and 3 months after intervention).

### Outcome Measures

**Figure 2:**
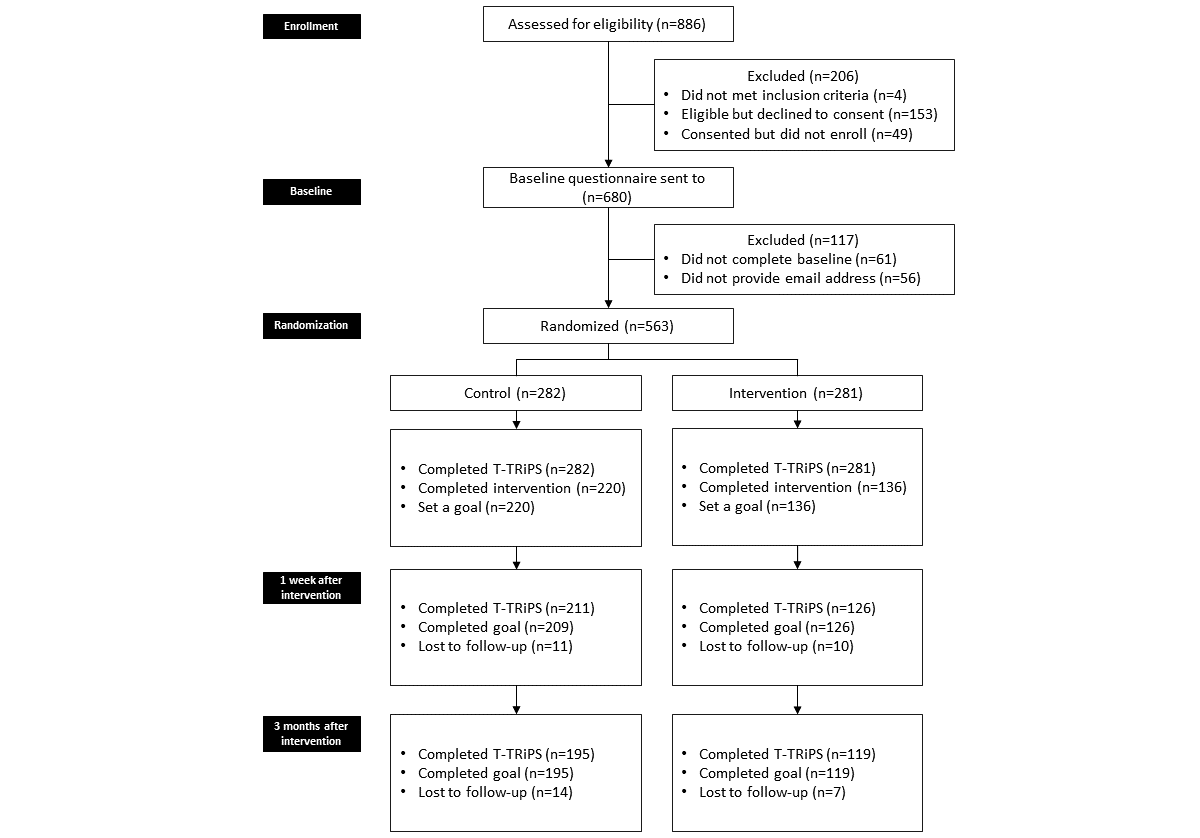
Study flow diagram.

Our primary outcome measure was change in the total score on the Teacher Tolerance of Risk in Play Scale (T-TRiPS), a validated, reliable 26-item measure with dichotomous yes or no responses on items that reflect the 6 categories of risky play (great heights, high speed, dangerous tools, dangerous elements, rough-and-tumble, and disappear or get lost) [36] proposed by Sandseter [34]. The T-TRiPS is a modified version of the Tolerance of Risk in Play Scale for parents [37], which measures teachers’ perceptions of risk. We assessed the psychometric properties of T-TRiPS in our sample using Rasch analysis, which considers the respondent’s ability to choose a *correct* (in the case of this study, the *yes* response) item and the difficulty of each item [38]. Rasch analysis converts categorical responses to interval data. This analysis was conducted using the mirt package in R software (R Core Team, version 4.0.0) [39,40]. Rasch analysis of the baseline data (563 participants completed T-TRiPS; Figure 2) resulted in dropping 1 item (“Do you wait to see how well the children in your center manage challenges before getting involved?”) owing to local dependence, such that this item was highly correlated with several other items on the T-TRiPS. The remaining 25 items resulted in the following model fit: root mean square error of approximation=0.060 (90% CI 0.056-0.065), standardized root mean square residual=0.101, Tucker-Lewis index=0.899, comparative fit index=0.899, and empirical reliability=0.851. *θ* standardized scores from the Rasch analysis of the final 25-item T-TRiPS ranged from −3.839 to 3.847, with a mean of −0.000 (SD 1.224). A higher standardized score indicated a higher tolerance of risk in play.

Our secondary outcome measure was self-reported behavior change, measured by participants’ self-reported progress in attaining the goal they set for themselves. At each follow-up time point, participants were reminded of their goal and asked, “Did you accomplish your goal?” with dichotomous yes or no responses.

We assessed the primary outcome measure at baseline and at 1 week and 3 months after intervention. We only assessed the secondary outcome measure at 1 week and 3 months after intervention because at baseline, they could not have accomplished a goal they had not yet set. We paid the participants US $25 via electronic transfer upon completing the baseline questionnaire and allocated intervention. We then paid them US $16 at each of the follow-up time points at 1 week and 3 months after intervention. In addition, we issued participants in the intervention condition a professional development certificate for 100 minutes upon completion of the OutsidePlay-ECE intervention.

### Statistical Analyses

We conducted all statistical analyses in Stata (StataCorp, version 15) [41].

### Power

For a sample size of 206 ECEs and ELCC administrators in total, a linear mixed model examining the impact of intervention relative to control, including an interaction with time, was calculated to have 80% power at a *P*=.05 level of significance to detect a difference of 0.75 between the intervention and control conditions on the T-TRiPS when the SD is 1.82, and the correlation between repeated observations is 0.75. From our previous work [20,21], we anticipated requiring 324 complete baseline requirements among ECEs and ELCC administrators who would then be randomized into the 2 conditions. We assumed a 75% retention rate at the 1 week after intervention follow-up time point (n=242) and an 85% retention rate at our 3 months after intervention follow-up time point, which would result in a final sample of 206 ECEs, corresponding to 103 in each condition.

### Descriptive Analysis

To compare the raw outcome differences between conditions at each time point, for continuous outcomes (TRiPS scores), we used 1-way ANOVA or Kruskal-Wallis *H* test (if variance is not equal between conditions). For categorical outcomes (goal attainment), we used the chi-square test. Significance level was set at *P*=.05.

### Effect of the Intervention

We concluded that linear and generalized linear mixed effects models with random intercepts and unstructured covariance were fit to analyze the effects of the intervention on T-TRiPS scores and goal attainment, respectively. In other words, we used the mixed effects regression analysis to examine (1) whether T-TRiPS scores changed between 1 week and 3 months after intervention and (2) whether these changes were greater in the intervention condition (ie, the OutsidePlay-ECE intervention) than in the control condition. We used intent-to-treat analysis of T-TRiPS scores that used the last observation carried forward as the method of imputation, because these participants only completed the baseline survey and did not complete the intervention, it is reasonable to expect their T-TRiPS scores to remain the same throughout the study period. Unstandardized (ie, raw) *β* coefficients were reported, which were interpreted as the change in T-TRiPS scores when comparing the intervention condition with the control condition at baseline.

Similar to the T-TRiPS analysis, we conducted a generalized mixed effects regression analysis to examine the effect of the intervention on goal attainment, when comparing the control condition at the 1 week after intervention follow-up time point with the intervention condition at the 3 months after intervention follow-up time point. An intention-to-treat analysis of goal attainment was not performed because of the absence of baseline data. To establish a goal, participants had to complete either interventions (eg, the OutsidePlay-ECE intervention or the Position Statement on Active Outdoor Play). Consequently, there was no basis for imputing the values of goal attainment. We calculated odds ratios, which were interpreted as the odds of attaining goals for the intervention condition at the 3 months after intervention follow-up time point, divided by the odds of the control condition at 1 week after intervention (relative effect size). We also calculated the absolute effect size, that is, risk differences and the probability of attaining a goal in the intervention group minus the probability in the control condition.

## Results

### Overview

Figure 2 shows the flow diagram of the study. A total of 563 ECEs were randomly allocated to 1 of the 2 intervention conditions using REDCap. Of these, 420 (74.6%) completed the baseline requirement, which included completion of the baseline survey and the intervention and setting up their goals. Although randomization produced roughly equal numbers of participants allocated to each condition, the intervention condition experienced the most dropouts (145/281, 51.6%) at the time of baseline when completing the intervention as compared to dropouts in the control condition (62/282, 21.9%). The intervention condition involved more time commitment from the participants, as completing the OutsidePlay-ECE intervention typically took up to 100 minutes compared with 15 to 20 minutes for the control condition. However, of the participants completing the intervention, we only lost 5.0% (11/220) and 6.6% (14/209) of the participants to follow-up at 1 week and 3 months after intervention respectively, versus 7.3% (10/136) and 5.5% (7/126) of the participants in the control condition. We confirmed fidelity to the intervention through a review of participants’ responses within each chapter of the intervention.

The University of British Columbia/Children’s and Women’s Health Center of British Columbia Research Ethics Board categorized the intervention as low risk and not associated with any harm. No privacy breaches or technical problems affected the participants. Although we tried to accommodate participants’ varying internet bandwidths by automatically adjusting the media resolution (eg, high or low), this did not resolve issues caused by some users accessing the intervention from old or incompatible devices.

### Sample Characteristics

We included baseline sociodemographic data from 563 ECEs and ELCC administrators who were randomized to a condition in our analyses (baseline characteristics between the 2 conditions are presented in Table 1). We did not observe any statistically significant differences between the baseline conditions with regard to all sociodemographic characteristics. We also compared sociodemographic data between the control and intervention conditions at the 1 week and 3 months after intervention follow-up time points, and no statistically significant differences were found.

We compared the sociodemographic characteristics among those who completed the baseline survey and between those who were randomized (N=563) and those who were not randomized (N=56), as these participants did not provide email address to proceed with the study and found that those who were randomized were more likely to be female (544/563, 96.6% vs 51/56, 91.1%, respectively; *P*=.04), less likely to be ECEs (392/563, 69.6% vs 48/56, 85.7% respectively; *P*=.04), more likely to be ELCC administrators (165/563, 29.3% vs 8/56, 14.3% respectively; *P*=.04), worked for a longer time in the field (mean 10.13, SD 9.33 vs mean 7.27, SD 7.64 years, respectively; *P*=.03), were more likely to be from British Columbia (258/560, 46.1% vs 15/53, 28.3% respectively; *P*=.007), less likely to be from Ontario (130/560, 23.2% vs 23/53, 43.4% respectively; *P*=.007), worked at a center with fewer children (for the number of children between 1 and 24: 186/557, 33.4% vs 8/51, 15.7% respectively; *P*=.03 and for the number of children ≥49: 217/557, 39% vs 26/51, 51% respectively; *P*=.03), and fewer staff (for the number of staff between 1 and 5: 202/551, 36.7% vs 10/52, 19.2% respectively; *P*=.006 and for the number of staff ≥13: 173/551, 31.4% vs 27/52, 51.9% respectively; *P*=.006). We did not find any statistical differences in other sociodemographic characteristics.

**Table 1:** Baseline characteristics between the 2 intervention conditions.

Characteristics of participants who completed the baseline survey		Control (n=282)	Intervention (n=281)	Total (N=563)
**Sex (N=563), n (%)**				
	Male	8 (2.8)	8 (2.9)	16 (2.8)
	Female	272 (96.4)	272 (96.8)	544 (96.6)
	Other	2 (0.7)	0 (0)	2 (0.4)
	Prefer not to answer	0 (0)	1 (0.4)	1 (0.2)
**Age (years; N=563), n (%)**				
	19 to 24	26 (9.3)	33 (11.8)	59 (10.5)
	25 to 30	72 (25.6)	55 (19.6)	127 (22.6)
	31 to 40	71 (25.3)	86 (30.7)	157 (28)
	41 to 50	66 (23.5)	64 (22.9)	130 (23.2)
	51 to 60	32 (11.4)	36 (12.8)	61 (12.1)
	61 to 70	13 (4.6)	6 (2.1)	19 (3.4)
	≥71	1 (0.4)	0 (0)	1 (0.2)
	Prefer not to answer	1 (0.4)	1 (0.4)	2 (0.4)
**Language (N=563), n (%)**				
	English	263 (93.3)	261 (92.9)	524 (93.1)
	Other^a^	19 (6.7)	20 (7.1)	39 (6.9)
**Role (N=563), n (%)**				
	ECE^b^	203 (72)	189 (67.3)	392 (69.6)
	ECE administrator	75 (26.6)	90 (32)	165 (29.3)
	Other^c^	4 (1.4)	2 (0.7)	6 (1.1)
Working in the field (years; N=530), mean (SD)		10.22 (9.51)	10.05 (9.16)	10.14 (9.33)
Working at the current center (years; N=530), mean (SD)		6.09 (7.41)	5.18 (5.98)	5.63 (6.74)
**Province of employment (N=560), n (%)**				
	Alberta	6 (2.1)	7 (2.5)	13 (2.3)
	British Columbia	129 (45.9)	129 (46.2)	258 (46.1)
	Manitoba	3 (1.1)	2 (0.7)	5 (0.9)
	New Brunswick	52 (18.5)	44 (15.8)	96 (17.1)
	Newfoundland and Labrador	8 (2.9)	12 (4.3)	20 (3.6)
	Nova Scotia	13 (4.6)	9 (3.2)	22 (3.9)
	Ontario	64 (22.8)	66 (23.7)	130 (23.2)
	Prince Edward Island	0 (0)	2 (0.7)	2 (0.4)
	Quebec	2 (0.7)	4 (1.4)	6 (1.1)
	Saskatchewan	4 (1.4)	4 (1.4)	8 (1.4)
**Whether the center is licensed (N=544), n (%)**				
	Yes	266 (97.4)	264 (97.4)	530 (97.4)
	No	7 (2.6)	7 (2.6)	14 (2.6)
**Children at the center (N=557), n (%)**				
	Small: 1 to 24	91 (32.7)	95 (34.1)	186 (33.4)
	Medium: 25 to 48	76 (27.3)	78 (28.0)	154 (27.7)
	Large: ≥49	111 (39.9)	106 (38.0)	217 (39.0)
**Staff at the center (N=551), n (%)**				
	Small: 1 to 5	100 (36.2)	102 (37.1)	202 (36.7)
	Medium: 6 to 12	85 (30.8)	91 (33.1)	176 (31.9)
	Large: ≥13	91 (33.0)	82 (29.8)	173 (31.4)
**Whether the center has a designated outdoor space for children (N=557), n (%)**				
	Yes	270 (97.1)	270 (96.8)	540 (97)
	No	8 (2.9)	9 (3.2)	17 (3)
**Quality of the center’s outdoor space for children (N=539), n (%)**				
	Very good	96 (35.6)	88 (32.7)	184 (34.1)
	Good	95 (35.2)	110 (40.9)	205 (38.0)
	Acceptable	65 (24.1)	60 (22.3)	125 (23.2)
	Poor	12 (4.4)	11 (4.1)	23 (4.3)
	Very poor	2 (0.7)	0 (0)	3 (0.4)
Time children spent playing outdoors at the center (hours; N=556), mean (SD)		2.01 (1.15)	2.14 (1.13)	2.08 (1.14)
**Feeling supported by colleagues in general (N=559), n (%)**				
	Yes	253 (89.7)	250 (90.3)	503 (90.0)
	No	11 (3.9)	14 (5.1)	25 (4.5)
	Feeling partially supported	8 (2.8)	4 (1.4)	12 (2.1)
	N/A^d^	10 (3.5)	9 (3.2)	19 (3.4)

^a^Arabic (n=3), Cantonese (n=3), Chinese (n=2), Croatian (n=2), Gujarati (n=2), Hindi (n=1), Hungarian (n=1), Korean (n=6), Mandarin (n=1), Minnan (a Chinese dialect; n=1), Nepali (n=1), Punjabi (n=4), Serbian (n=1), Sinhala (n=1), Sinhalese (n=1), Slovak (n=2), Spanish (n=2), Tagalog (n=2), Tamil (n=2), Turkish (n=1), and Dutch (n=1).

^b^ECE: early childhood educator.

^c^Includes childcare provider consultant (n=1), child and youth care (n=1), no ECE (n=2), classroom teacher (n=1), and instructor at college (n=1).

^d^N/A: not applicable; for example, the participant is the only staff member.

### Primary Outcome: T-TRiPS

Table 2 presents the description of T-TRiPS scores by intervention conditions and time points, without accounting for time effects, the interaction of intervention by time effects. We did not find any statistical differences in T-TRiPS scores between different conditions at baseline among those who reported T-TRiPS scores or among those who completed the intervention at baseline. At both the 1 week and 3 months after intervention follow-up time points, T-TRiPS scores were significantly higher in the intervention condition than in the control condition (1 week: mean −0.156, SD 1.304, for the control condition and mean 0.262, SD 1.117, for the intervention condition, *P*=.003; and 3 months: mean −0.118, SD 1.400, for the control condition and mean 0.200, SD 1.211, for the intervention condition, *P*=.04).

Table 3 describes the findings of the mixed effects regression analysis, considering intervention effects, time effects, and iteration of intervention by time effects. Participants who completed the intervention condition had significantly higher T-TRiPS scores than those who completed the control condition at 1 week (0.320, 95% CI 0.135-0.505; *P*=.001) and 3 months (0.251, 95% CI 0.062-0.440; *P*=.009) after intervention, indicating sustained change. Results of the intention-to-treat analyses for the effects of the intervention on T-TRiPS scores largely replicated the aforementioned analyses, indicating that ECEs and ELCC administrators in the intervention condition were significantly more likely to increase their T-TRiPS scores at 1 week (0.335, 95% CI 0.156-0.514; *P*<.001) and 3 months (0.271, 95% CI 0.088-0.454; *P*<.004) after intervention compared with those in the control condition.

**Table 2:** Description of the Teacher Tolerance of Risk in Play Scale scores by intervention conditions and time points.

Evaluation period	Sample size, N	Control, mean (SD)	Intervention, mean (SD)	*P* value for 1-way ANOVA
Baseline	563	0.040 (1.207)	−0.040 (1.243)	.44
Completed intervention	356	0.017 (1.211)	0.123 (1.196)	.86
1 week after intervention	337	−0.156 (1.304)	0.262 (1.117)	.003
3 months after intervention	314	−0.118 (1.400)	0.200 (1.211)	.04

**Table 3:** Mixed effects regression analysis for the Teacher Tolerance of Risk in Play Scale (T-TRiPS) θ score.

Regression and condition comparisons			Coefficients (95% CI)	*P* value for coefficients	*P* value for joint test of main effects	
*Raw T-TRiPS* *θ* *scores (N=356, for those who were randomized to a condition, completed baseline T-TRiPS measure, and completed the intervention)^a^*						
	Intervention effects: intervention versus control		0.100 (−0.169 to 0.369)	.47		.02
	**Time effects**					.99
		1 week versus baseline	−0.154 (−0.267 to −0.041)	.007		
		3 months versus baseline	−0.124 (−0.240 to −0.008)	.04		
	**Intervention by time effects**					.002
		Intervention versus control by 1 week versus baseline	0.320 (0.135 to 0.505)	.001		
		Intervention versus control by 3 months versus baseline	0.251 (0.062 to 0.440)	.009		
*Intention-to-treat analysis (imputed T-TRiPS* *θ* *scores; N=563, for those who were randomized to a condition and completed baseline T-TRiPS measure)*						
	Intervention effects: intervention versus control		0.019 (−0.217 to 0.254)	.88		.054
	**Time effects**					.96
		1 week versus baseline	−0.156 (−0.268 to −0.044)	.006		
		3 months versus baseline	−0.126 (−0.241 to −0.011)	.03		
	**Intervention by time effects**					<.001
		Intervention versus control by 1 week versus baseline	0.335 (0.156 to 0.514)	<.001		
		Intervention versus control by 3 months versus baseline	0.271 (0.088 to 0.454)	.004		

^a^Italicization denotes two separate sets of analysis.

### Secondary Outcome: Goal Attainment

We asked participants to think of one tangible and achievable goal and created a feasible plan to accomplish it. Participant goals varied widely from “I could add more building tools” to “Bring this topic and learning opportunity up with my colleagues at our daily check-in.” Table 4 presents the description of goal attainment by condition and time point, without accounting for time effects and the interaction between time and intervention effects. We did not find any statistically significant differences in the secondary outcome, goal attainment, between the 2 conditions at either 1 week after intervention (141/209, 67.5% for the control condition and 94/126, 74.6% for the intervention condition; *P*=.17) or 3 months after intervention (163/195, 83.6% for the control condition and 106/119, 89.1% for the intervention condition; *P*=.18).

Table 5 presents the results of the generalized mixed effects regression analysis. There was no statistical difference in goal attainment between participants in the intervention and control condition at 3 months after intervention compared with 1 week after intervention (odds ratio 1.124, 95% CI 0.335-3.774; *P*=.85).

**Table 4:** Goal attainment by intervention condition and time point.

Evaluation period and goal attainment		Control, n (%)		Intervention, n (%)	Sample size, N			*P* value for chi-square	
**1 week after intervention**						335			.17
	Yes	141 (67.5)	94 (74.6)						
	No	68 (32.5)	32 (25.4)						
**3 months after intervention**						314			.18
	Yes	163 (83.6)	106 (89.1)						
	No	32 (26.4)	13 (10.9)						

**Table 5:** Results of the mixed effects regression analysis for goal attainment by intervention condition and time.

Regression and condition comparisons^a^	Relative effect size		Absolute effect sizes	
	Odds ratios (95% CI)	*P* value	Risk differences (95% CI)	*P* value
Intervention effects: intervention versus control	2.046 (0.740 to 5.655)	.17	0.071 (−0.019 to 0.162)	.12
Time effects: 3 months versus 1 week	5.749 (2.664 to 12.407)	<.001	0.158 (0.098 to 0.218)	<.001
Intervention by time effects: intervention versus control by 3 months versus 1 week	1.124 (0.335 to 3.774)	.85	−0.018 (−0.115 to 0.079)	.72

^a^N=335, who were randomized to a condition and set a goal at baseline and completed goal attainment measures at a follow-up time point.

## Discussion

### Principal Findings

Our RCT tested the effectiveness of the web-based OutsidePlay-ECE intervention in changing ECEs’ and ELCC administrators’ tolerance of risk in play and the attainment of their personalized goals for change to support children’s outdoor play within the ELCC. The RCT results partially support our hypotheses. ECEs and ELCC administrators receiving the intervention reported significantly higher increases in their tolerance of risk in play at 1 week after intervention than participants in the control condition. These differences remained significant at 3 months after intervention. There were no significant differences in goal attainment. These results are consistent with the findings of a previous RCT testing the OutsidePlay-Parent intervention, which also found significantly greater increases in tolerance of risk in play for intervention versus control participants at 1 week and 3 months after intervention [20].

There are several possibilities for the lack of a significant effect of the intervention on the secondary outcome, goal attainment. Findings reported for the OutsidePlay-Parent intervention [21] were similar, and it was postulated that the null finding may have resulted from the participants’ goals being too ambitious or not sufficiently actionable. To limit participants from developing overly ambitious or less actionable goals, a short clip of video in the last chapter was included to encourage participants to consider one thing that they can do to support children’s outdoor play: “It shouldn’t be something too big or complicated. Make sure it is concrete and achievable—that you don’t feel overwhelmed by it.” In addition, we provided some basic actionable goals and stressed that this was meant to be the first step in a journey toward change. We recognized that addressing the many barriers and challenges to outdoor play in ELCC environments would require complex intervention at multiple organizational levels (eg, the individual, relationship with colleagues, and licensing regulations) [42,43]. The OutsidePlay-ECE intervention was designed to open their minds to a different way of thinking and approach, which would be the first step in the process. Although participants may have thought that they were setting a manageable goal, they may have subsequently realized that it was more ambitious than anticipated and evaluated their actions as insufficient. Furthermore, it is possible that although the intervention successfully opened their minds to a new way of thinking, as evidenced by the increase in tolerance of risk in play, it did not sufficiently influence their self-efficacy in implementing the change, even a small one, and that a more intensive and complex intervention is required to shift behavior.

As ECE attitudes toward outdoor play and risk-taking in play have a major impact on children’s outdoor play in ELCCs [27], avenues for shifting attitudes are necessary to foster changes in outdoor play provision. The OutsidePlay-ECE intervention was efficient and effective in changing ECEs and ELCC administrators’ tolerance to the risk of children’s outdoor play. Given the ease of distribution, no cost to users, and low resource requirement for ongoing maintenance of the web-based tool, the OutsidePlay-ECE intervention can be easily deployed in these efforts. This is particularly relevant during the COVID-19 pandemic, when increased outdoor time is recommended as a major strategy for reducing transmission [44]. Outdoor play is a means for improving children’s mental health and coping strategies [45,46], and a web-based tool is the most feasible way to deliver such an intervention. In conclusion, the findings support the use of OutsidePlay-ECE as an intervention to improve outdoor play in ELCCs.

### Limitations

Our study had several limitations. First, although social cognitive theory and respective behavior change techniques have been mapped [20], we did not test the hypothesized relationships between the theory constructs and outcomes. This limits our ability to better understand why we observed sustained changes in T-TRiPS and why we did not observe such changes in goal attainment. Given the significant findings on T-TRiPS, future research can explore the potential causal pathways leading to intervention effectiveness. Second, participant attrition was greater in the intervention condition than in the control condition. We did not find any sociodemographic differences between the 2 conditions among participants who remained in the study. However, attrition is a concern for eHealth interventions [47], and the field would benefit from further research on the factors that influence participant retention. Third, we conducted a study during the COVID-19 pandemic, which included initial ELCC closures. The data collection period occurred after most ELCCs resumed operations. However, practices remained in flux as Canadian ELCCs received rapidly evolving provincial and federal guidance regarding recommended procedures, whereas understanding and implementation of the guidance was challenging and varied between centers. Although this may have provided novel insights into ECEs’ and ELCC administrators’ perceptions of children’s outdoor play in the specific context of the pandemic, the findings may have differed in other conditions. Fourth, data collection spanned winter and spring, and it is unclear whether seasonal changes influence risk tolerance. However, few of the questions within the T-TRiPS would be expected to differ with seasons, as they assess more general risk attitudes (eg, “Do you wait to see how well the children in your centre manage challenges before getting involved?”). Finally, we did not implement a system to monitor dwell time on the Position Statement on Active Outdoor Play, which is the document shared with participants in the control condition. Therefore, we did not know whether the participants read the document or how long they took to do so. In future research, this information could offer opportunities for further analyses.

### Conclusions

ELCCs are important settings for influencing early childhood, and these childcare experiences can impact lifelong health, development, and well-being trajectories [48]. High-quality early childhood education can mitigate the effects of early adversity and reduce inequities in more disadvantaged children [49]. Research is growing on the importance of outdoor play to children’s physical, social, and cognitive development; risk perception; and mental health [14,16-19], and it is necessary to ensure children’s regular and repeated access to outdoor play opportunities, particularly in ELCCs. To facilitate this, ECEs need to understand the essence and benefits of risky outdoor play for children and how best to provide and accommodate it. Our RCT results demonstrated that the OutsidePlay-ECE web-based intervention is effective in increasing ECEs’ and ELCC administrators’ risk tolerance in children’s outdoor play. As an easily accessible and free resource, the OutsidePlay-ECE has great potential to support early childhood education practices. For example, it can be integrated into ECE professional development, provided as a standalone ECE program, and revisited over time to help ECEs deepen their understanding and expand their practice related to outdoor play provision.

## Appendix

Multimedia Appendix 1 CONSORT-eHEALTH checklist (V 1.6.1).

Multimedia Appendix 2 Complete screenshots of the OutsidePlay-ECE intervention.

## Abbreviations

CONSORT-EHEALTH: Consolidated Standards of Reporting Trials of Electronic and Mobile Health Applications and Online Telehealth
ECE: early childhood educator
ELCC: early learning childcare center
RCT: randomized controlled trial
REDCap: Research Electronic Data Capture
T-TRiPS: Teacher Tolerance of Risk in Play Scale

## Data Availability

All data, password-protected and stored in the secure network at the British Columbia Children’s Hospital Research Institute, will be available from MB upon reasonable request within 5 years of the completion of the study.
