# Supplementary material for: Evaluation of the Web-Based OutsidePlay-ECE Intervention to Influence Early Childhood Educators’ Attitudes and Supportive Behaviors Toward Outdoor Play: Randomized Controlled Trial
Source: J Med Internet Res. 2022 Jun 10;24(6):e36826. doi: 10.2196/36826 (PMC9233267; doi:10.2196/36826)
Supplement: Multimedia Appendix 1 [file jmir_v24i6e36826_app1.pdf]

CONSORT-EHEALTH (V 1.6.1) - Submission/Publication Form

http://tinyurl.com/consort-ehealth-v1-6

Your name *
First Last
Mariana Brussoni
Your answer

Primary Affiliation (short), City, Country *
University of Toronto, Toronto, Canada
University of British Columbia
Your answer

Your e-mail address *
mbrussoni@bcchr.ca
Your answer

Title of your manuscript *
Provide the (draft) title of your manuscript.
Results of the OutsidePlay-ECE Study Randomized Controlled Trial to Evaluate a Web-Based Intervention to Influence Early Childhood Educators’ Attitudes and Supportive Behaviors Toward Outdoor PlayTrial
Your answer

Name of your App/Software/Intervention *
If there is a short and a long/alternate name, write the short name first and add the long name in brackets.
Outsideplay.ca
Your answer

Evaluated Version (if any)
e.g. "V1", "Release 2017-03-01", "Version 2.0.27913"
V1
Your answer

Language(s) *
What language is the intervention/app in? If multiple languages are available, separate by comma (e.g. "English, French")
English, French

Your answer

URL of your Intervention Website or App
e.g. a direct link to the mobile app on app in appstore (itunes, Google Play), or URL of the website. If the intervention is a DVD or hardware, you can also link to an Amazon page.
https://outsideplay.ca
URL of an image/screenshot (optional)
Your answer

Accessibility *
Can an enduser access the intervention presently?
access is free and open

Primary Medical Indication/Disease/Condition *
e.g. "Stress", "Diabetes", or define the target group in brackets after the condition, e.g. "Autism (Parents of children with)", "Alzheimers (Informal Caregivers of)"
Risk Aversion
Your answer

Primary Outcomes measured in trial *
comma-separated list of primary outcomes reported in the trial
Tolerance of Risk in Play - Teacher
Your answer

Secondary/other outcomes
Are there any other outcomes the intervention is expected to affect?
Your answer
Goal attainment

Recommended "Dose" *
What do the instructions for users say on how often the app should be used?
As needed
Approx. Percentage of Users (starters) still using the app as recommended after 3 months *
unknown / not evaluated

Overall, was the app/intervention effective? *
yes: all primary outcomes were significantly better in intervention group vs control

Article Preparation Status/Stage *
At which stage in your article preparation are you currently (at the time you fill in this form)
not submitted yet - in late draft status, just before submission

Journal *
If you already know where you will submit this paper (or if it is already submitted), please provide the journal name (if it is not JMIR, provide the journal name under "other")
Journal of Medical Internet Research (JMIR)

Is this a full powered effectiveness trial or a pilot/feasibility trial? *
Fully powered

Manuscript tracking number *
If this is a JMIR submission, please provide the manuscript tracking number under "other" (The ms tracking number can be found in the submission acknowledgement email, or when you login as author in JMIR. If the paper is already published in JMIR, then the ms tracking number is the four-digit number at the end of the DOI, to be found at the bottom of each published article in JMIR)
no ms number (yet) / not (yet) submitted to / published in JMIR
Other:

TITLE AND ABSTRACT

1a) TITLE: Identification as a randomized trial in the title

A Web-Based Risk Reframing Intervention to Influence Early Childhood Educators' Attitudes and Supportive Behaviors Toward Outdoor Play: Randomized Controlled Trial

1a) Does your paper address CONSORT item 1a? *
I.e does the title contain the phrase "Randomized Controlled Trial"? (if not, explain the reason under "other")
yes

1a-i) Identify the mode of delivery in the title
Identify the mode of delivery. Preferably use "web-based" and/or "mobile" and/or "electronic game" in the title. Avoid ambiguous terms like "online", "virtual", "interactive". Use "Internet-based" only if Intervention includes non-web-based Internet components (e.g. email), use "computer-based" or "electronic" only if offline products are used. Use "virtual" only in the context of "virtual reality" (3-D worlds). Use "online" only in the context of "online support groups". Complement or substitute product names with broader terms for the class of products (such as "mobile" or "smart phone" instead of "iphone"), especially if the application runs on different platforms.

subitem not at all important

1
2
3
4
5

essential

Does your paper address subitem 1a-i? *

Copy and paste relevant sections from manuscript title (include quotes in quotation marks "like this" to indicate direct quotes from your manuscript), or elaborate on this item by providing additional information not in the ms, or briefly explain why the item is not applicable/relevant for your study
Your answer
"Results of the OutsidePlay-ECE Study Randomized Controlled Trial to Develop and Evaluate a Web-Based Intervention to Influence Early Childhood Educators' Attitudes and Supportive Behaviors Toward Outdoor Play"

1a-ii) Non-web-based components or important co-interventions in title
Mention non-web-based components or important co-interventions in title, if any (e.g., "with telephone support").
subitem not at all important
1
2
3
4
5
essential
Does your paper address subitem 1a-ii?
Copy and paste relevant sections from manuscript title (include quotes in quotation marks "like this" to indicate direct quotes from your manuscript), or elaborate on this item by providing additional information not in the ms, or briefly explain why the item is not applicable/relevant for your study
Your answer
Our study included only web-based components

1a-iii) Primary condition or target group in the title
Mention primary condition or target group in the title, if any (e.g., "for children with Type I Diabetes") Example: A Web-based and Mobile Intervention with Telephone Support for Children with Type I Diabetes: Randomized Controlled Trial
subitem not at all important
1
2
3
4
5
essential
Does your paper address subitem 1a-iii? *
Copy and paste relevant sections from manuscript title (include quotes in quotation marks "like this" to indicate direct quotes from your manuscript), or elaborate on this item by

providing additional information not in the ms, or briefly explain why the item is not applicable/relevant for your study

Your answer

“Results of the OutsidePlay-ECE Study Randomized Controlled Trial to Develop and Evaluate a Web-Based Intervention to Influence Early Childhood Educators’ Attitudes and Supportive Behaviors Toward Outdoor Play”

1b) ABSTRACT: Structured summary of trial design, methods, results, and conclusions

NPT extension: Description of experimental treatment, comparator, care providers, centers, and blinding status.

1b-i) Key features/functionalities/components of the intervention and comparator in the METHODS section of the ABSTRACT

Mention key features/functionalities/components of the intervention and comparator in the abstract. If possible, also mention theories and principles used for designing the site. Keep in mind the needs of systematic reviewers and indexers by including important synonyms. (Note: Only report in the abstract what the main paper is reporting. If this information is missing from the main body of text, consider adding it)

subitem not at all important

1
2
3
4
5

essential

Does your paper address subitem 1b-i? *

Copy and paste relevant sections from the manuscript abstract (include quotes in quotation marks "like this" to indicate direct quotes from your manuscript), or elaborate on this item by providing additional information not in the ms, or briefly explain why the item is not applicable/relevant for your study

Your answer

" The OutsidePlay-ECE risk-reframing intervention is a fully-automated and open access web-based intervention to reframe early childhood educators’ perception of the importance of outdoor play and risk in play, and to promote a change in their practice in supporting for children’s outdoor play in early learning and childcare center settings. We grounded the intervention in social cognitive theory and behavior change techniques.”

1b-ii) Level of human involvement in the METHODS section of the ABSTRACT

Clarify the level of human involvement in the abstract, e.g., use phrases like “fully automated” vs. “therapist/nurse/care provider/physician-assisted” (mention number and expertise of providers involved, if any). (Note: Only report in the abstract what the main paper is reporting. If this information is missing from the main body of text, consider adding it)

subitem not at all important

1
2
3
4
5

essential

Does your paper address subitem 1b-ii?
Copy and paste relevant sections from the manuscript abstract (include quotes in quotation marks "like this" to indicate direct quotes from your manuscript), or elaborate on this item by providing additional information not in the ms, or briefly explain why the item is not applicable/relevant for your study
Your answer
" The OutsidePlay-ECE risk-reframing intervention is a fully-automated and open access web-based intervention to reframe early childhood educators' perception of the importance of outdoor play and risk in play, and to promote a change in their practice in supporting for children's outdoor play in early learning and childcare center settings."

1b-iii) Open vs. closed, web-based (self-assessment) vs. face-to-face assessments in the METHODS section of the ABSTRACT
Mention how participants were recruited (online vs. offline), e.g., from an open access website or from a clinic or a closed online user group (closed usergroup trial), and clarify if this was a purely web-based trial, or there were face-to-face components (as part of the intervention or for assessment). Clearly say if outcomes were self-assessed through questionnaires (as common in web-based trials). Note: In traditional offline trials, an open trial (open-label trial) is a type of clinical trial in which both the researchers and participants know which treatment is being administered. To avoid confusion, use "blinded" or "unblinded" to indicated the level of blinding instead of "open", as "open" in web-based trials usually refers to "open access" (i.e. participants can self-enrol). (Note: Only report in the abstract what the main paper is reporting. If this information is missing from the main body of text, consider adding it)

subitem not at all important

1
2
3
4
5

essential

Does your paper address subitem 1b-iii?
Copy and paste relevant sections from the manuscript abstract (include quotes in quotation marks "like this" to indicate direct quotes from your manuscript), or elaborate on this item by providing additional information not in the ms, or briefly explain why the item is not applicable/relevant for your study
Your answer

“We conducted recruitment primarily through social media and mass emails through our partner and professional networks. We invited those interested in participating to self-assess their eligibility: working as an early childhood educator and/or administrator in an early learning and childcare center in Canada, and able to speak, read and understand English.”

1b-iv) RESULTS section in abstract must contain use data
Report number of participants enrolled/assessed in each group, the use/uptake of the intervention (e.g., attrition/adherence metrics, use over time, number of logins etc.), in addition to primary/secondary outcomes. (Note: Only report in the abstract what the main paper is reporting. If this information is missing from the main body of text, consider adding it)

subitem not at all important

1
2
3
4
5

essential

Does your paper address subitem 1b-iv?
Copy and paste relevant sections from the manuscript abstract (include quotes in quotation marks "like this" to indicate direct quotes from your manuscript), or elaborate on this item by providing additional information not in the ms, or briefly explain why the item is not applicable/relevant for your study
Your answer
“total of 563 participants completed baseline survey consisting of sociodemographic questions and a questionnaire that assessed participant tolerance for risk in play, self-efficacy, and stage of change. These participants were then randomized: 281 in the intervention, and 282 in the control condition. Of 281 participants who were allocated to the intervention condition, 199 completed the baseline requirement (i.e., completion of the baseline survey and the intervention, and setting-up their goal). Respectively, of 282 participants who were allocated to the control condition, 221 completed the baseline requirement. At 1-week post intervention 126 and 209 participants completed assessments for each condition, respectively, and at 3-month post intervention, 119 and 195 completed the assessments, respectively.”

1b-v) CONCLUSIONS/DISCUSSION in abstract for negative trials
Conclusions/Discussions in abstract for negative trials: Discuss the primary outcome - if the trial is negative (primary outcome not changed), and the intervention was not used, discuss whether negative results are attributable to lack of uptake and discuss reasons. (Note: Only report in the abstract what the main paper is reporting. If this information is missing from the main body of text, consider adding it)

subitem not at all important

1

2
3
4
5
essential

Does your paper address subitem 1b-v?
Copy and paste relevant sections from the manuscript abstract (include quotes in quotation marks "like this" to indicate direct quotes from your manuscript), or elaborate on this item by providing additional information not in the ms, or briefly explain why the item is not applicable/relevant for your study
Your answer
“Compared to participants in the control condition, participants in the intervention had significantly higher tolerance of risk in play at 1-week (β=0.323, P= 0.001) and 3-month post-intervention (β=0.342, P= 0.001), after controlling for sociodemographic covariates. Intention-to-treat analysis replicated these findings (β=0.348, P= 0.001 and β=0.35, P= 0.001, respectively). No significant intervention effect was found for the goal attainment outcome.”

INTRODUCTION

2a) In INTRODUCTION: Scientific background and explanation of rationale

2a-i) Problem and the type of system/solution
Describe the problem and the type of system/solution that is object of the study: intended as stand-alone intervention vs. incorporated in broader health care program? Intended for a particular patient population? Goals of the intervention, e.g., being more cost-effective to other interventions, replace or complement other solutions? (Note: Details about the intervention are provided in “Methods” under 5)
subitem not at all important
1
2
3
4
5
essential

Does your paper address subitem 2a-i? *
Copy and paste relevant sections from the manuscript (include quotes in quotation marks "like this" to indicate direct quotes from your manuscript), or elaborate on this item by providing additional information not in the ms, or briefly explain why the item is not applicable/relevant for your study
Your answer
“we developed a new version for early childhood educators (ECEs) (OutsidePlay-ECE) on the OutsidePlay intervention. Similar to the OutsidePlay-Parents module, the new module for the ECE used health behavior change theory and techniques to address ECEs’ attitudes and behaviors towards outdoor play and its inherent risks 22,23”
And,

“For many children, most of their waking hours are spent in an ELCC and this can be an invaluable opportunity to provide children with high quality opportunities for outdoor play, particularly for children who may have limited access to outdoor play in their home environments 25,26. Unfortunately, this opportunity is not fully leveraged due to various limiting factors. Amid the societal risk aversion trend and childcare licensing guidelines interpreted in restrictive ways, ECEs face many actual and perceived barriers that are primarily linked to safety concerns 27,28. Furthermore, these barriers intersect with individual ECEs’ cultural backgrounds, and level of confidence, knowledge and experience in promoting and accommodating children’s outdoor play, as well as support received (perceived and actual) from their colleagues and ELCC administration 27–29.”

2a-ii) Scientific background, rationale: What is known about the (type of) system

Scientific background, rationale: What is known about the (type of) system that is the object of the study (be sure to discuss the use of similar systems for other conditions/diagnoses, if appropiate), motivation for the study, i.e. what are the reasons for and what is the context for this specific study, from which stakeholder viewpoint is the study performed, potential impact of findings [2]. Briefly justify the choice of the comparator.

subitem not at all important

1

2

3

4

5

essential

Does your paper address subitem 2a-ii? *

Copy and paste relevant sections from the manuscript (include quotes in quotation marks "like this" to indicate direct quotes from your manuscript), or elaborate on this item by providing additional information not in the ms, or briefly explain why the item is not applicable/relevant for your study

Your answer

“The intervention mobilizes evidence-based behavior change techniques underpinned in social cognitive theory to change ECE’s perception of outdoor play and practices in support of children’s outdoor play in early learning childcare center (ELCC) settings 20.”

2b) In INTRODUCTION: Specific objectives or hypotheses

Does your paper address CONSORT subitem 2b? *

Copy and paste relevant sections from the manuscript (include quotes in quotation marks "like this" to indicate direct quotes from your manuscript), or elaborate on this item by providing additional information not in the ms, or briefly explain why the item is not applicable/relevant for your study

Your answer

"This paper reports the results of a randomized controlled trial (RCT) evaluating the efficacy of the OutsidePlay-ECE intervention to increase ECEs and ELCC administrators' tolerance of risk in play, and attain a behavior change goal related to providing outdoor play opportunities for children in their ELCC.."

METHODS

3a) Description of trial design (such as parallel, factorial) including allocation ratio

Does your paper address CONSORT subitem 3a? *
Copy and paste relevant sections from the manuscript (include quotes in quotation marks "like this" to indicate direct quotes from your manuscript), or elaborate on this item by providing additional information not in the ms, or briefly explain why the item is not applicable/relevant for your study
Your answer
"We used a single-blind (researchers and outcome assessors) two-parallel condition RCT."
And
"Participants had equal (50%) likelihood of being assigned to each condition."

3b) Important changes to methods after trial commencement (such as eligibility criteria), with reasons

Does your paper address CONSORT subitem 3b? *
Copy and paste relevant sections from the manuscript (include quotes in quotation marks "like this" to indicate direct quotes from your manuscript), or elaborate on this item by providing additional information not in the ms, or briefly explain why the item is not applicable/relevant for your study
Your answer
There was not change to methods after trial commencement.

3b-i) Bug fixes, Downtimes, Content Changes
Bug fixes, Downtimes, Content Changes: ehealth systems are often dynamic systems. A description of changes to methods therefore also includes important changes made on the intervention or comparator during the trial (e.g., major bug fixes or changes in the functionality or content) (5-iii) and other "unexpected events" that may have influenced study design such as staff changes, system failures/downtimes, etc. [2].

subitem not at all important

1
2
3
4
5

essential

Does your paper address subitem 3b-i?
Copy and paste relevant sections from the manuscript (include quotes in quotation marks "like this" to indicate direct quotes from your manuscript), or elaborate on this item by providing additional information not in the ms, or briefly explain why the item is not applicable/relevant for your study
Your answer
“We temporarily halted participant recruitment and participation from December 18, 2020 to January 4, 2021, to accommodate the Christmas and New Year’s holidays, during which most ELCCs were closed. We made this decision to secure a more valid participant responses to the goal attainment question in the 1-week post-intervention follow-up time point, asking “Did you accomplish your goal?”, which concerned their behavior in promoting children’s outdoor play specifically in their ELCC. We posted a message on the REDCap enrollment survey informing participants of this interruption and the date that RCT recruitment would resume.”

4a) Eligibility criteria for participants

Does your paper address CONSORT subitem 4a? *
Copy and paste relevant sections from the manuscript (include quotes in quotation marks "like this" to indicate direct quotes from your manuscript), or elaborate on this item by providing additional information not in the ms, or briefly explain why the item is not applicable/relevant for your study
Your answer
" Eligible participants were adult ECEs and/or ELCC administrators in Canada who can speak, read and understand English."

4a-i) Computer / Internet literacy
Computer / Internet literacy is often an implicit “de facto” eligibility criterion - this should be explicitly clarified.

subitem not at all important

1
2
3
4
5

essential

Does your paper address subitem 4a-i?
Copy and paste relevant sections from the manuscript (include quotes in quotation marks "like this" to indicate direct quotes from your manuscript), or elaborate on this item by providing additional information not in the ms, or briefly explain why the item is not applicable/relevant for your study
Your answer
“As the study was conducted entirely online, computer and Internet literacy was in fact an implicit “de facto” eligibility criterion.”

4a-ii) Open vs. closed, web-based vs. face-to-face assessments:
Open vs. closed, web-based vs. face-to-face assessments: Mention how participants were recruited (online vs. offline), e.g., from an open access website or from a clinic, and clarify if this was a purely web-based trial, or there were face-to-face components (as part of the intervention or for assessment), i.e., to what degree got the study team to know the participant. In online-only trials, clarify if participants were quasi-anonymous and whether having multiple identities was possible or whether technical or logistical measures (e.g., cookies, email confirmation, phone calls) were used to detect/prevent these.

subitem not at all important

1

2

3

4

5

essential

Does your paper address subitem 4a-ii? *
Copy and paste relevant sections from the manuscript (include quotes in quotation marks "like this" to indicate direct quotes from your manuscript), or elaborate on this item by providing additional information not in the ms, or briefly explain why the item is not applicable/relevant for your study

Your answer
"We recruited participants between December 1, 2020 to March 15, 2021 via social media posts – FaceBook, FaceBook Ads, Twitter and Instagram – and mass email through partner and professional networks. Potential participants completed an online survey in the REDCap electronic data capture tool, which was hosted by and stored in the British Columbia Children's Hospital Research Institute server 31. We included a complete description and procedure of the study on the online survey allowing participants to self-assess their eligibility, and to consent online with the capability of downloading the consent form, if they decided to participate in our study."

4a-iii) Information giving during recruitment
Information given during recruitment. Specify how participants were briefed for recruitment and in the informed consent procedures (e.g., publish the informed consent documentation as appendix, see also item X26), as this information may have an effect on user self-selection, user expectation and may also bias results.

subitem not at all important

1

2

3

4

5

essential

Does your paper address subitem 4a-iii?
Copy and paste relevant sections from the manuscript (include quotes in quotation marks "like this" to indicate direct quotes from your manuscript), or elaborate on this item by providing additional information not in the ms, or briefly explain why the item is not applicable/relevant for your study
Your answer
"We included a complete description and procedure of the study on the online survey allowing participants to self-assess their eligibility, and to consent online with the capability of downloading the consent form, if they decided to participate in our study.)."

4b) Settings and locations where the data were collected

Does your paper address CONSORT subitem 4b? *
Copy and paste relevant sections from the manuscript (include quotes in quotation marks "like this" to indicate direct quotes from your manuscript), or elaborate on this item by providing additional information not in the ms, or briefly explain why the item is not applicable/relevant for your study
Your answer
" Participant data were collected and stored in the British Columbia Children's Hospital Research Institute REDCap server. "

4b-i) Report if outcomes were (self-)assessed through online questionnaires
Clearly report if outcomes were (self-)assessed through online questionnaires (as common in web-based trials) or otherwise.

subitem not at all important

1
2
3
4
5

essential

Does your paper address subitem 4b-i? *
Copy and paste relevant sections from the manuscript (include quotes in quotation marks "like this" to indicate direct quotes from your manuscript), or elaborate on this item by providing additional information not in the ms, or briefly explain why the item is not applicable/relevant for your study
Your answer
"Our secondary outcome measure was self-reported behavior change, measured by participants' self-reported progress on attaining the goal they set for themselves. At each follow-up time point, participants were reminded of their goal and asked "Did you accomplish your goal?" with dichotomous yes/no responses."

4b-ii) Report how institutional affiliations are displayed
Report how institutional affiliations are displayed to potential participants [on ehealth media], as affiliations with prestigious hospitals or universities may affect volunteer rates, use, and reactions with regards to an intervention.(Not a required item – describe only if this may bias results)

subitem not at all important

1
2
3
4
5

essential

Does your paper address subitem 4b-ii?
Copy and paste relevant sections from the manuscript (include quotes in quotation marks "like this" to indicate direct quotes from your manuscript), or elaborate on this item by providing additional information not in the ms, or briefly explain why the item is not applicable/relevant for your study
Your answer
"The RCT was approved by the University of British Columbia / Children's and Women's Health Center of British Columbia Research Ethics Board (H19-03644)"

5) The interventions for each group with sufficient details to allow replication, including how and when they were actually administered

5-i) Mention names, credential, affiliations of the developers, sponsors, and owners
Mention names, credential, affiliations of the developers, sponsors, and owners [6] (if authors/evaluators are owners or developer of the software, this needs to be declared in a "Conflict of interest" section or mentioned elsewhere in the manuscript).

subitem not at all important

1
2
3
4
5

essential

Does your paper address subitem 5-i?
Copy and paste relevant sections from the manuscript (include quotes in quotation marks "like this" to indicate direct quotes from your manuscript), or elaborate on this item by providing additional information not in the ms, or briefly explain why the item is not applicable/relevant for your study
Your answer
" The RCT was approved by the University of British Columbia / Children's and Women's Health Center of British Columbia Research Ethics Board (H19-03644)."
And

“This RCT study was funded by the Lawson Foundation, Grant #GRT 2019-79. The Lawson Foundation was not involved in any aspect of study design or writing of the manuscript. MB is supported by salary awards from the British Columbia Children’s Hospital Research Institute. We are further grateful to the Government of Canada – Early Learning and Child Care – under the Social Development Partnership program, Project #016554719 for funding for the development of the OutsidePlay-ECE intervention. We thank the Digital Lab at the University of British Columbia Department of Pediatrics for providing partnership and technical support in development and programing of the OutsidePlay intervention.”

5-ii) Describe the history/development process
Describe the history/development process of the application and previous formative evaluations (e.g., focus groups, usability testing), as these will have an impact on adoption/use rates and help with interpreting results.

subitem not at all important

1
2
3
4
5

essential

Does your paper address subitem 5-ii?
Copy and paste relevant sections from the manuscript (include quotes in quotation marks "like this" to indicate direct quotes from your manuscript), or elaborate on this item by providing additional information not in the ms, or briefly explain why the item is not applicable/relevant for your study
Your answer
“MB conceived of the study. CH, FM, MZ, MW, TC and EO assisted MB with development of the OutsidePlay-ECE intervention content. MB and JJ led development of the intervention design, with contribution from CH. YL performed statistical analysis. CH and YL wrote the first full draft of this manuscript. All authors read and approved the final manuscript.
And,
" For complete details on the development and content of the OutsidePlay intervention, please refer to our research protocol paper."

5-iii) Revisions and updating
Revisions and updating. Clearly mention the date and/or version number of the application/intervention (and comparator, if applicable) evaluated, or describe whether the intervention underwent major changes during the evaluation process, or whether the development and/or content was “frozen” during the trial. Describe dynamic components such as news feeds or changing content which may have an impact on the replicability of the intervention (for unexpected events see item 3b).

subitem not at all important

1

2
3
4
5

essential

Does your paper address subitem 5-iii?
Copy and paste relevant sections from the manuscript (include quotes in quotation marks "like this" to indicate direct quotes from your manuscript), or elaborate on this item by providing additional information not in the ms, or briefly explain why the item is not applicable/relevant for your study
Your answer
" The intervention was soft launched on December 1, 2020 for the RCT and the content was frozen during the RCT and analysis."

5-iv) Quality assurance methods
Provide information on quality assurance methods to ensure accuracy and quality of information provided [1], if applicable.

subitem not at all important

1
2
3
4
5

essential

Does your paper address subitem 5-iv?
Copy and paste relevant sections from the manuscript (include quotes in quotation marks "like this" to indicate direct quotes from your manuscript), or elaborate on this item by providing additional information not in the ms, or briefly explain why the item is not applicable/relevant for your study
Your answer
"We sent out participants in the intervention up to three sets of automated reminders via email per time point within 24-, 48-, and 60 hours of completion of the baseline survey through the REDCap (...) Likewise, we sent out automatic reminders within 24-, 48-, and 60-hour when 1-week and 3-month post-intervention follow-up measures were deployed by the REDCap, if they did not finish the measure at any given follow-up time points."
However, our study did not include any additional quality assurance measure to ensure accuracy and quality of information provided by participants as the study was strictly automated and administered online.

5-v) Ensure replicability by publishing the source code, and/or providing screenshots/screen-capture video, and/or providing flowcharts of the algorithms used
Ensure replicability by publishing the source code, and/or providing screenshots/screen-capture video, and/or providing flowcharts of the algorithms used. Replicability (i.e., other

researchers should in principle be able to replicate the study) is a hallmark of scientific reporting.

subitem not at all important

1
2
3
4
5

essential

Does your paper address subitem 5-v?

Copy and paste relevant sections from the manuscript (include quotes in quotation marks "like this" to indicate direct quotes from your manuscript), or elaborate on this item by providing additional information not in the ms, or briefly explain why the item is not applicable/relevant for your study

Your answer

" Multimedia Appendix 2: Complete screenshots of the OutsidePlay-ECE intervention "

5-vi) Digital preservation

Digital preservation: Provide the URL of the application, but as the intervention is likely to change or disappear over the course of the years; also make sure the intervention is archived (Internet Archive, webcitation.org, and/or publishing the source code or screenshots/videos alongside the article). As pages behind login screens cannot be archived, consider creating demo pages which are accessible without login.

subitem not at all important

1
2
3
4
5

essential

Does your paper address subitem 5-vi?

Copy and paste relevant sections from the manuscript (include quotes in quotation marks "like this" to indicate direct quotes from your manuscript), or elaborate on this item by providing additional information not in the ms, or briefly explain why the item is not applicable/relevant for your study

Your answer

The https://outsideplay.ca will be sponsored and preserved by the BC Children's Hospital Research Institute and the University of British Columbia until the content become obsolete and Dr. Brussoni decides to close down the site.

5-vii) Access

Access: Describe how participants accessed the application, in what setting/context, if they had to pay (or were paid) or not, whether they had to be a member of specific group. If known, describe how participants obtained "access to the platform and Internet" [1]. To

ensure access for editors/reviewers/readers, consider to provide a “backdoor” login account or demo mode for reviewers/readers to explore the application (also important for archiving purposes, see vi).

subitem not at all important

1
2
3
4
5

essential

Does your paper address subitem 5-vii? *

Copy and paste relevant sections from the manuscript (include quotes in quotation marks "like this" to indicate direct quotes from your manuscript), or elaborate on this item by providing additional information not in the ms, or briefly explain why the item is not applicable/relevant for your study

Your answer

“"We sent participants a unique link to their materials upon completion of baseline survey and randomization.”

5-viii) Mode of delivery, features/functionalities/components of the intervention and comparator, and the theoretical framework

Describe mode of delivery, features/functionalities/components of the intervention and comparator, and the theoretical framework [6] used to design them (instructional strategy [1], behaviour change techniques, persuasive features, etc., see e.g., [7, 8] for terminology). This includes an in-depth description of the content (including where it is coming from and who developed it) [1],” whether [and how] it is tailored to individual circumstances and allows users to track their progress and receive feedback” [6]. This also includes a description of communication delivery channels and – if computer-mediated communication is a component – whether communication was synchronous or asynchronous [6]. It also includes information on presentation strategies [1], including page design principles, average amount of text on pages, presence of hyperlinks to other resources, etc. [1].

subitem not at all important

1
2
3
4
5

essential

Does your paper address subitem 5-viii? *

Copy and paste relevant sections from the manuscript (include quotes in quotation marks "like this" to indicate direct quotes from your manuscript), or elaborate on this item by providing additional information not in the ms, or briefly explain why the item is not applicable/relevant for your study

Your answer

See under Risk Reframing Intervention and Comparison Condition under Methods.

5-ix) Describe use parameters
Describe use parameters (e.g., intended “doses” and optimal timing for use). Clarify what instructions or recommendations were given to the user, e.g., regarding timing, frequency, heaviness of use, if any, or was the intervention used ad libitum.

subitem not at all important

1
2
3
4
5

essential

Does your paper address subitem 5-ix?
Copy and paste relevant sections from the manuscript (include quotes in quotation marks "like this" to indicate direct quotes from your manuscript), or elaborate on this item by providing additional information not in the ms, or briefly explain why the item is not applicable/relevant for your study
Your answer
" The OutsidePlay intervention consists of three chapters, which is guided by the animated character of MB and could be completed in up to 100 minutes, depending on participants’ movements through each chapter. Participants could also come back to the intervention at their convenience, picking up from where they left off previously."

5-x) Clarify the level of human involvement
Clarify the level of human involvement (care providers or health professionals, also technical assistance) in the e-intervention or as co-intervention (detail number and expertise of professionals involved, if any, as well as “type of assistance offered, the timing and frequency of the support, how it is initiated, and the medium by which the assistance is delivered”. It may be necessary to distinguish between the level of human involvement required for the trial, and the level of human involvement required for a routine application outside of a RCT setting (discuss under item 21 – generalizability).

subitem not at all important

1
2
3
4
5

essential

Does your paper address subitem 5-x?

Copy and paste relevant sections from the manuscript (include quotes in quotation marks "like this" to indicate direct quotes from your manuscript), or elaborate on this item by providing additional information not in the ms, or briefly explain why the item is not applicable/relevant for your study

Your answer

“As aforementioned, given that the RCT (including the intervention) was conducted entirely online, there was no human involvement except when participants had inquiries and/or reported technical issues via email. This limited human involvement enabled that only identifiable information we have collected from participants was their email address.”

5-xi) Report any prompts/reminders used

Report any prompts/reminders used: Clarify if there were prompts (letters, emails, phone calls, SMS) to use the application, what triggered them, frequency etc. It may be necessary to distinguish between the level of prompts/reminders required for the trial, and the level of prompts/reminders for a routine application outside of a RCT setting (discuss under item 21 – generalizability).

subitem not at all important

1
2
3
4
5

essential

Does your paper address subitem 5-xi? *

Copy and paste relevant sections from the manuscript (include quotes in quotation marks "like this" to indicate direct quotes from your manuscript), or elaborate on this item by providing additional information not in the ms, or briefly explain why the item is not applicable/relevant for your study

Your answer

" We conducted the RCT (including the intervention) entirely online, thus, there was no human involvement except when participants had inquiries and/or reported technical issues via email..”

And,

" We sent out participants in the intervention up to three sets of automated reminders via email per time point within 24-, 48-, and 60 hours of completion of the baseline survey through the REDCap. In other words, the REDCap activated and sent out the reminders once the baseline survey measure was completed for each participant. Likewise, we sent out automatic reminders within 24-, 48-, and 60-hour when 1-week and 3-month post-intervention follow-up measures were deployed by the REDCap, if they did not finish the measure at any given follow-up time points (i.e., 1-week and 3-months post-intervention)."

5-xii) Describe any co-interventions (incl. training/support)

Describe any co-interventions (incl. training/support): Clearly state any interventions that are provided in addition to the targeted eHealth intervention, as ehealth intervention may

not be designed as stand-alone intervention. This includes training sessions and support [1]. It may be necessary to distinguish between the level of training required for the trial, and the level of training for a routine application outside of a RCT setting (discuss under item 21 – generalizability.

subitem not at all important

1
2
3
4
5

essential

Does your paper address subitem 5-xii? *

Copy and paste relevant sections from the manuscript (include quotes in quotation marks "like this" to indicate direct quotes from your manuscript), or elaborate on this item by providing additional information not in the ms, or briefly explain why the item is not applicable/relevant for your study

Your answer

There was no co-intervention. There are only intervention and control groups.

6a) Completely defined pre-specified primary and secondary outcome measures, including how and when they were assessed

Does your paper address CONSORT subitem 6a? *

Copy and paste relevant sections from the manuscript (include quotes in quotation marks "like this" to indicate direct quotes from your manuscript), or elaborate on this item by providing additional information not in the ms, or briefly explain why the item is not applicable/relevant for your study

Your answer

See Outcome Measures under Methods section.

6a-i) Online questionnaires: describe if they were validated for online use and apply CHERRIES items to describe how the questionnaires were designed/deployed

If outcomes were obtained through online questionnaires, describe if they were validated for online use and apply CHERRIES items to describe how the questionnaires were designed/deployed [9].

subitem not at all important

1
2
3
4
5

essential

Does your paper address subitem 6a-i?

Copy and paste relevant sections from manuscript text
Your answer
“Our primary outcome measure was increase in the total score on the Teacher Tolerance of Risk in Play Scale (T-TRiPS), a validated, reliable 26-item measure with dichotomous yes/no responses on items that reflect Sandseter’s 34 six categories of risky play (great heights, high speed, dangerous tools, dangerous elements, rough-and-tumble, disappear/get lost) 36. The T-TRiPS is a modified version of the TRiPS for parents 37 measuring teachers’ perception of risk.”

6a-ii) Describe whether and how “use” (including intensity of use/dosage) was defined/measured/monitored
Describe whether and how “use” (including intensity of use/dosage) was defined/measured/monitored (logins, logfile analysis, etc.). Use/adoption metrics are important process outcomes that should be reported in any ehealth trial.
subitem not at all important
1
2
3
4
5
essential
Does your paper address subitem 6a-ii?
Copy and paste relevant sections from manuscript text

Your answer
" We sent out participants in the intervention up to three sets of automated reminders via email per time point within 24-, 48-, and 60 hours of completion of the baseline survey through the REDCap."
And,
“We did not send out automated reminders to participants in the control condition because once they opened the Position Statement on Active Outdoor Play, and closed their survey at that point, we considered they completed the baseline requirement. However, we reminded them up to three times (24-, 48- and 72-hours) if they did not finish the survey measures at any follow-up time point (i.e., 1-week and 3-months post-intervention).”

6a-iii) Describe whether, how, and when qualitative feedback from participants was obtained
Describe whether, how, and when qualitative feedback from participants was obtained (e.g., through emails, feedback forms, interviews, focus groups).
subitem not at all important
1
2
3

4
5
essential

Does your paper address subitem 6a-iii?
Copy and paste relevant sections from manuscript text
Your answer
As aforementioned, given that the RCT (including the intervention) was conducted entirely online, there was no human involvement except when participants had inquiries and/or reported technical issues via email. We didn't ask participants for any qualitative feedback.

6b) Any changes to trial outcomes after the trial commenced, with reasons

Does your paper address CONSORT subitem 6b? *
Copy and paste relevant sections from the manuscript (include quotes in quotation marks "like this" to indicate direct quotes from your manuscript), or elaborate on this item by providing additional information not in the ms, or briefly explain why the item is not applicable/relevant for your study
Your answer
There was no change to trial outcomes after the trial commenced.

7a) How sample size was determined
NPT: When applicable, details of whether and how the clustering by care provides or centers was addressed

7a-i) Describe whether and how expected attrition was taken into account when calculating the sample size
Describe whether and how expected attrition was taken into account when calculating the sample size.
subitem not at all important
1
2
3
4
5
essential

Does your paper address subitem 7a-i?
Copy and paste relevant sections from manuscript title (include quotes in quotation marks "like this" to indicate direct quotes from your manuscript), or elaborate on this item by providing additional information not in the ms, or briefly explain why the item is not applicable/relevant for your study
Your answer
" For a sample size of 206 ECEs and ELCC administrators in total, a linear mixed model examining the impact of intervention relative to control including an interaction with time will have 80% power at a 0.05 level of significance to detect a difference of 0.75 between intervention and control conditions when the standard deviation is 1.82 and the correlation between repeated observations is 0.75. From our previous work 20,40, we anticipate requiring

324 complete baseline requirement among ECEs and ELCC administrators who would then be randomized into the two conditions. We assumed a 75% retention rate at our 1-week post-intervention follow-up time point (n = 242) and an 85% retention rate at our 3-month post-intervention follow-up time point, which would result in a final sample of 206 ECEs, corresponding to 103 in each condition."

7b) When applicable, explanation of any interim analyses and stopping guidelines

Does your paper address CONSORT subitem 7b? *
Copy and paste relevant sections from the manuscript (include quotes in quotation marks "like this" to indicate direct quotes from your manuscript), or elaborate on this item by providing additional information not in the ms, or briefly explain why the item is not applicable/relevant for your study
Your answer
There was no interim analysis conducted.

8a) Method used to generate the random allocation sequence
NPT: When applicable, how care providers were allocated to each trial group

Does your paper address CONSORT subitem 8a? *
Copy and paste relevant sections from the manuscript (include quotes in quotation marks "like this" to indicate direct quotes from your manuscript), or elaborate on this item by providing additional information not in the ms, or briefly explain why the item is not applicable/relevant for your study
Your answer
See Randomization and Blinding under Methods section.

8b) Type of randomisation; details of any restriction (such as blocking and block size)

Does your paper address CONSORT subitem 8b? *
Copy and paste relevant sections from the manuscript (include quotes in quotation marks "like this" to indicate direct quotes from your manuscript), or elaborate on this item by providing additional information not in the ms, or briefly explain why the item is not applicable/relevant for your study
Your answer
“We automatically randomized enrolled participants who completed the baseline survey in the REDCap to one of the two conditions: intervention and control. Participants had equal (50%) likelihood of being assigned to each condition. We generated the randomization schedule beforehand via sealedenvelope.com using randomized permuted blocks of size 4, 6, and 8. We then transferred the list to the REDCap. We concealed allocations to the researchers at participant assignment and during data analysis. We sent participants a unique link to their materials upon completion of baseline survey and randomization. The nature of the intervention did not permit participant blinding. They may have intuited which condition they were allocated to based on the details of the two conditions provided in the consent form: intervention (e.g., web-based intervention) and control (i.e., a portable document format document).”

9) Mechanism used to implement the random allocation sequence (such as sequentially numbered containers), describing any steps taken to conceal the sequence until interventions were assigned

Does your paper address CONSORT subitem 9? *

Copy and paste relevant sections from the manuscript (include quotes in quotation marks "like this" to indicate direct quotes from your manuscript), or elaborate on this item by providing additional information not in the ms, or briefly explain why the item is not applicable/relevant for your study

Your answer

See our answer to 8b.

10) Who generated the random allocation sequence, who enrolled participants, and who assigned participants to interventions

Does your paper address CONSORT subitem 10? *

Copy and paste relevant sections from the manuscript (include quotes in quotation marks "like this" to indicate direct quotes from your manuscript), or elaborate on this item by providing additional information not in the ms, or briefly explain why the item is not applicable/relevant for your study

Your answer

See our answer to 8b.

11a) If done, who was blinded after assignment to interventions (for example, participants, care providers, those assessing outcomes) and how

NPT: Whether or not administering co-interventions were blinded to group assignment

11a-i) Specify who was blinded, and who wasn't

Specify who was blinded, and who wasn't. Usually, in web-based trials it is not possible to blind the participants [1, 3] (this should be clearly acknowledged), but it may be possible to blind outcome assessors, those doing data analysis or those administering co-interventions (if any).

subitem not at all important

1

2

3

4

5

essential

Does your paper address subitem 11a-i? *

Copy and paste relevant sections from the manuscript (include quotes in quotation marks "like this" to indicate direct quotes from your manuscript), or elaborate on this item by providing additional information not in the ms, or briefly explain why the item is not applicable/relevant for your study

Your answer
See our answer to 8b.

11a-ii) Discuss e.g., whether participants knew which intervention was the "intervention of interest" and which one was the "comparator"
Informed consent procedures (4a-ii) can create biases and certain expectations - discuss e.g., whether participants knew which intervention was the "intervention of interest" and which one was the "comparator".
subitem not at all important
1
2
3
4
5
essential
Does your paper address subitem 11a-ii?
Copy and paste relevant sections from the manuscript (include quotes in quotation marks "like this" to indicate direct quotes from your manuscript), or elaborate on this item by providing additional information not in the ms, or briefly explain why the item is not applicable/relevant for your study
Your answer
See our answer to 8b.

11b) If relevant, description of the similarity of interventions
(this item is usually not relevant for ehealth trials as it refers to similarity of a placebo or sham intervention to a active medication/intervention)
Does your paper address CONSORT subitem 11b? *
Copy and paste relevant sections from the manuscript (include quotes in quotation marks "like this" to indicate direct quotes from your manuscript), or elaborate on this item by providing additional information not in the ms, or briefly explain why the item is not applicable/relevant for your study

Your answer
"We asked participants in the control condition to review a portable document format of the Position Statement on Active Outdoor Play – a four page document with information on research and recommendations for action in addressing barriers to outdoor play 4,35. We estimated participants take 15-20 minutes to read through the document."

12a) Statistical methods used to compare groups for primary and secondary outcomes
NPT: When applicable, details of whether and how the clustering by care providers or centers was addressed

Does your paper address CONSORT subitem 12a? *
Copy and paste relevant sections from the manuscript (include quotes in quotation marks "like this" to indicate direct quotes from your manuscript), or elaborate on this item by providing additional information not in the ms, or briefly explain why the item is not applicable/relevant for your study
Your answer
See Outcome Measures under Methods section.

12a-i) Imputation techniques to deal with attrition / missing values
Imputation techniques to deal with attrition / missing values: Not all participants will use the intervention/comparator as intended and attrition is typically high in ehealth trials. Specify how participants who did not use the application or dropped out from the trial were treated in the statistical analysis (a complete case analysis is strongly discouraged, and simple imputation techniques such as LOCF may also be problematic [4]).
subitem not at all important
1
2
3
4
5
essential
Does your paper address subitem 12a-i? *
Copy and paste relevant sections from the manuscript (include quotes in quotation marks "like this" to indicate direct quotes from your manuscript), or elaborate on this item by providing additional information not in the ms, or briefly explain why the item is not applicable/relevant for your study
Your answer
"We employed intent-to-treat analysis of T-TRiPS scores that used last-observation-carried forward (LOCF) as the method of imputation, because missing data due to loss-to-follow-up were primarily in the intervention condition (n=73 at 1-week, as compared to n=11 at the control condition)."

12b) Methods for additional analyses, such as subgroup analyses and adjusted analyses
Does your paper address CONSORT subitem 12b? *
Copy and paste relevant sections from the manuscript (include quotes in quotation marks "like this" to indicate direct quotes from your manuscript), or elaborate on this item by providing additional information not in the ms, or briefly explain why the item is not applicable/relevant for your study
Your answer
We have not done any additional analyses such as subgroup or adjusted analyses.

X26) REB/IRB Approval and Ethical Considerations [recommended as subheading under "Methods"] (not a CONSORT item)

X26-i) Comment on ethics committee approval

subitem not at all important

1
2
3
4
5

essential

Does your paper address subitem X26-i?

Copy and paste relevant sections from the manuscript (include quotes in quotation marks "like this" to indicate direct quotes from your manuscript), or elaborate on this item by providing additional information not in the ms, or briefly explain why the item is not applicable/relevant for your study

Your answer

" We registered our RCT with the United States National Institute of Health's Protocol Registration and Results System (registered #: NCT04624932). The RCT was approved by the University of British Columbia / Children's and Women's Health Center of British Columbia Research Ethics Board (H19-03644)."

x26-ii) Outline informed consent procedures

Outline informed consent procedures e.g., if consent was obtained offline or online (how? Checkbox, etc.?), and what information was provided (see 4a-ii). See [6] for some items to be included in informed consent documents.

subitem not at all important

1
2
3
4
5

essential

Does your paper address subitem X26-ii?

Copy and paste relevant sections from the manuscript (include quotes in quotation marks "like this" to indicate direct quotes from your manuscript), or elaborate on this item by providing additional information not in the ms, or briefly explain why the item is not applicable/relevant for your study

Your answer

" We included a complete description and procedure of the study on the online survey allowing participants to self-assess their eligibility, and to consent online with the capability of downloading the consent form, if they decided to participate in our study. "

“Eligible and interested participants provided consent by downloading the consent form for their review and selecting a checkbox to participate..”

X26-iii) Safety and security procedures
Safety and security procedures, incl. privacy considerations, and any steps taken to reduce the likelihood or detection of harm (e.g., education and training, availability of a hotline)

subitem not at all important

1
2
3
4
5

essential

Does your paper address subitem X26-iii?
Copy and paste relevant sections from the manuscript (include quotes in quotation marks "like this" to indicate direct quotes from your manuscript), or elaborate on this item by providing additional information not in the ms, or briefly explain why the item is not applicable/relevant for your study
Your answer
“The sole identifiable information collected was participants’ email addresses, which were required for sending allocated study material, follow-up measures, and reminders. We did not export participants’ email addresses for data analysis. We assigned each participant a study number, which did not include any personal identifiable information.”

RESULTS

13a) For each group, the numbers of participants who were randomly assigned, received intended treatment, and were analysed for the primary outcome
NPT: The number of care providers or centers performing the intervention in each group and the number of patients treated by each care provider in each center

Does your paper address CONSORT subitem 13a? *
Copy and paste relevant sections from the manuscript (include quotes in quotation marks "like this" to indicate direct quotes from your manuscript), or elaborate on this item by providing additional information not in the ms, or briefly explain why the item is not applicable/relevant for your study
Your answer
“Figure 2 shows the study flow diagram. We randomly allocated a total of 563 ECEs automatically to one of the two intervention conditions using the REDCap. Of these, 420 completed the baseline requirement which includes completion of the baseline survey and the intervention, and setting-up their goal. While randomization produced roughly equal numbers of participants allocated to each condition, the intervention condition experienced

the most drop-outs (n=73) at 1-week post-intervention follow-up time point compared to baseline (n=10). The intervention condition involved more time commitment from the participants, since completing OutsidePlay-ECE intervention typically took up to 100 minutes compared to 15-20 minutes for the control condition. However, of the participants completing the intervention, we only lost 7 to follow-up at 3-months post-intervention, versus 14 in the control condition. We confirmed fidelity to the intervention through review of the participants' responses within each chapter of the intervention."

13b) For each group, losses and exclusions after randomisation, together with reasons

Does your paper address CONSORT subitem 13b? (NOTE: Preferably, this is shown in a CONSORT flow diagram) *

Copy and paste relevant sections from the manuscript (include quotes in quotation marks "like this" to indicate direct quotes from your manuscript), or elaborate on this item by providing additional information not in the ms, or briefly explain why the item is not applicable/relevant for your study

Your answer

See our response to 13a.

13b-i) Attrition diagram

Strongly recommended: An attrition diagram (e.g., proportion of participants still logging in or using the intervention/comparator in each group plotted over time, similar to a survival curve) or other figures or tables demonstrating usage/dose/engagement.

subitem not at all important

1
2
3
4
5

essential

Does your paper address subitem 13b-i?

Copy and paste relevant sections from the manuscript or cite the figure number if applicable (include quotes in quotation marks "like this" to indicate direct quotes from your manuscript), or elaborate on this item by providing additional information not in the ms, or briefly explain why the item is not applicable/relevant for your study

Your answer

Yes, it's included in our figure.

14a) Dates defining the periods of recruitment and follow-up

Does your paper address CONSORT subitem 14a? *

Copy and paste relevant sections from the manuscript (include quotes in quotation marks "like this" to indicate direct quotes from your manuscript), or elaborate on this item by

providing additional information not in the ms, or briefly explain why the item is not applicable/relevant for your study
Your answer

14a-i) Indicate if critical “secular events” fell into the study period
Indicate if critical “secular events” fell into the study period, e.g., significant changes in Internet resources available or “changes in computer hardware or Internet delivery resources”

subitem not at all important

1
2
3
4
5

essential

Does your paper address subitem 14a-i?
Copy and paste relevant sections from the manuscript (include quotes in quotation marks "like this" to indicate direct quotes from your manuscript), or elaborate on this item by providing additional information not in the ms, or briefly explain why the item is not applicable/relevant for your study
Your answer
There was no secular events fell into our study period. Only relevant event to report in our study was the interruption mentioned in Question 5-iii.

14b) Why the trial ended or was stopped (early)

Does your paper address CONSORT subitem 14b? *
Copy and paste relevant sections from the manuscript (include quotes in quotation marks "like this" to indicate direct quotes from your manuscript), or elaborate on this item by providing additional information not in the ms, or briefly explain why the item is not applicable/relevant for your study
Your answer
n/a

15) A table showing baseline demographic and clinical characteristics for each group
NPT: When applicable, a description of care providers (case volume, qualification, expertise, etc.) and centers (volume) in each group

Does your paper address CONSORT subitem 15? *
Copy and paste relevant sections from the manuscript (include quotes in quotation marks "like this" to indicate direct quotes from your manuscript), or elaborate on this item by providing additional information not in the ms, or briefly explain why the item is not applicable/relevant for your study
Your answer
See table 1.

15-i) Report demographics associated with digital divide issues
In ehealth trials it is particularly important to report demographics associated with digital divide issues, such as age, education, gender, social-economic status, computer/Internet/ehealth literacy of the participants, if known.

subitem not at all important

1
2
3
4
5

essential

Does your paper address subitem 15-i? *
Copy and paste relevant sections from the manuscript (include quotes in quotation marks "like this" to indicate direct quotes from your manuscript), or elaborate on this item by providing additional information not in the ms, or briefly explain why the item is not applicable/relevant for your study
Your answer
See table 1. It includes demographic categories such as sex, age, language, role, years working in the field and so on.

16) For each group, number of participants (denominator) included in each analysis and whether the analysis was by original assigned groups

16-i) Report multiple “denominators” and provide definitions
Report multiple “denominators” and provide definitions: Report N’s (and effect sizes) “across a range of study participation [and use] thresholds” [1], e.g., N exposed, N consented, N used more than x times, N used more than y weeks, N participants “used” the intervention/comparator at specific pre-defined time points of interest (in absolute and relative numbers per group). Always clearly define “use” of the intervention.

subitem not at all important

1
2
3
4
5

essential

Does your paper address subitem 16-i? *
Copy and paste relevant sections from the manuscript (include quotes in quotation marks "like this" to indicate direct quotes from your manuscript), or elaborate on this item by providing additional information not in the ms, or briefly explain why the item is not applicable/relevant for your study
Your answer
Yes.

(1) Participant characteristics (**Table 1**): N=563, consented, provided socio-demographics at baseline survey, provided an email address, and was randomized to a condition. (So, here, we do have some socio-demo information for those who did not provide an email address, n=56. See the second paragraph of the "participant characteristic" under "results" section in the manuscript –there are differences.)
(2) PRIMARY outcome (TRIPS), description: **Table 2**, for those who were randomized and reported TRIPS score at each different evaluation period.
(3) PRIMARY outcome (TRIPS), Intervention effect sizes (**Table 3**): N=377, for those who were randomized to a condition, completed the intervention, completed baseline sociodemographic and completed baseline T-TRiPS measure
(4) PRIMARY outcome (TRIPS), Intervention effect sizes, intention-to-treat analysis (**Table 3**): N=481, for those who were randomized to a condition, and completed baseline sociodemographic and completed baseline T-TRiPS measure
(5) SECONDARY outcome (goal attainment): **Table 4**, for those who were randomized and reported goal information at each different evaluation period.
(6) SECONDARY outcome (goal attainment), Intervention effect sizes (**Table 5**): N=299, who were randomized to a condition, and completed baseline sociodemographic, set a goal at baseline, completed goal attainment measures at follow-up.)

16-ii) Primary analysis should be intent-to-treat
Primary analysis should be intent-to-treat, secondary analyses could include comparing only "users", with the appropriate caveats that this is no longer a randomized sample (see 18-i).

subitem not at all important

1
2
3
4
5

essential

Does your paper address subitem 16-ii?
Copy and paste relevant sections from the manuscript (include quotes in quotation marks "like this" to indicate direct quotes from your manuscript), or elaborate on this item by providing additional information not in the ms, or briefly explain why the item is not applicable/relevant for your study
Your answer
Yes. Table 2 provides both analysis

17a) For each primary and secondary outcome, results for each group, and the estimated effect size and its precision (such as 95% confidence interval)

Does your paper address CONSORT subitem 17a? *
Copy and paste relevant sections from the manuscript (include quotes in quotation marks "like this" to indicate direct quotes from your manuscript), or elaborate on this item by providing additional information not in the ms, or briefly explain why the item is not applicable/relevant for your study
Your answer

Yes, all tables reporting effect sizes come with 95% Cis: table 3 & 5

17a-i) Presentation of process outcomes such as metrics of use and intensity of use
In addition to primary/secondary (clinical) outcomes, the presentation of process outcomes such as metrics of use and intensity of use (dose, exposure) and their operational definitions is critical. This does not only refer to metrics of attrition (13-b) (often a binary variable), but also to more continuous exposure metrics such as “average session length”. These must be accompanied by a technical description how a metric like a “session” is defined (e.g., timeout after idle time) [1] (report under item 6a).

subitem not at all important

1
2
3
4
5

essential

Does your paper address subitem 17a-i?
Copy and paste relevant sections from the manuscript (include quotes in quotation marks "like this" to indicate direct quotes from your manuscript), or elaborate on this item by providing additional information not in the ms, or briefly explain why the item is not applicable/relevant for your study
Your answer
Not applicable

17b) For binary outcomes, presentation of both absolute and relative effect sizes is recommended

Does your paper address CONSORT subitem 17b? *
Copy and paste relevant sections from the manuscript (include quotes in quotation marks "like this" to indicate direct quotes from your manuscript), or elaborate on this item by providing additional information not in the ms, or briefly explain why the item is not applicable/relevant for your study
Your answer
We reported both relative and absolute effect sizes in Table 5, in the format of Odds Ratios, risk differences, and their 95% Cis, respectively.

18) Results of any other analyses performed, including subgroup analyses and adjusted analyses, distinguishing pre-specified from exploratory

Does your paper address CONSORT subitem 18? *
Copy and paste relevant sections from the manuscript (include quotes in quotation marks "like this" to indicate direct quotes from your manuscript), or elaborate on this item by providing additional information not in the ms, or briefly explain why the item is not applicable/relevant for your study
Your answer
NA

18-i) Subgroup analysis of comparing only users

A subgroup analysis of comparing only users is not uncommon in ehealth trials, but if done, it must be stressed that this is a self-selected sample and no longer an unbiased sample from a randomized trial (see 16-iii).

subitem not at all important

1
2
3
4
5

essential

Does your paper address subitem 18-i?

Copy and paste relevant sections from the manuscript (include quotes in quotation marks "like this" to indicate direct quotes from your manuscript), or elaborate on this item by providing additional information not in the ms, or briefly explain why the item is not applicable/relevant for your study

Your answer

Not applicable. We are not doing sub-group analysis.

19) All important harms or unintended effects in each group
(for specific guidance see CONSORT for harms)

Does your paper address CONSORT subitem 19? *

Copy and paste relevant sections from the manuscript (include quotes in quotation marks "like this" to indicate direct quotes from your manuscript), or elaborate on this item by providing additional information not in the ms, or briefly explain why the item is not applicable/relevant for your study

Your answer

No harms nor unintended effects were found.

19-i) Include privacy breaches, technical problems

Include privacy breaches, technical problems. This does not only include physical “harm” to participants, but also incidents such as perceived or real privacy breaches [1], technical problems, and other unexpected/unintended incidents. “Unintended effects” also includes unintended positive effects [2].

subitem not at all important

1
2
3
4
5

essential

Does your paper address subitem 19-i?

Copy and paste relevant sections from the manuscript (include quotes in quotation marks "like this" to indicate direct quotes from your manuscript), or elaborate on this item by

providing additional information not in the ms, or briefly explain why the item is not applicable/relevant for your study
Your answer
NA

19-ii) Include qualitative feedback from participants or observations from staff/researchers
Include qualitative feedback from participants or observations from staff/researchers, if available, on strengths and shortcomings of the application, especially if they point to unintended/unexpected effects or uses. This includes (if available) reasons for why people did or did not use the application as intended by the developers.

subitem not at all important

1
2
3
4
5

essential

Does your paper address subitem 19-ii?
Copy and paste relevant sections from the manuscript (include quotes in quotation marks "like this" to indicate direct quotes from your manuscript), or elaborate on this item by providing additional information not in the ms, or briefly explain why the item is not applicable/relevant for your study
Your answer
We did not receive qualitative feedback from participants. However we have indicated our ‘researcher’ observations on strengths and shortcomings of the application and the RCT in Strengths and Limitations.

DISCUSSION

22) Interpretation consistent with results, balancing benefits and harms, and considering other relevant evidence
NPT: In addition, take into account the choice of the comparator, lack of or partial blinding, and unequal expertise of care providers or centers in each group

22-i) Restate study questions and summarize the answers suggested by the data, starting with primary outcomes and process outcomes (use)
Restate study questions and summarize the answers suggested by the data, starting with primary outcomes and process outcomes (use).

subitem not at all important

1
2
3
4
5

essential

Does your paper address subitem 22-i? *

Copy and paste relevant sections from the manuscript (include quotes in quotation marks "like this" to indicate direct quotes from your manuscript), or elaborate on this item by providing additional information not in the ms, or briefly explain why the item is not applicable/relevant for your study
Your answer
Our RCT tested the efficacy of the web-based OutsidePlay-ECE intervention in changing ECEs and ELCC administrators' tolerance of risk in play and the attainment of their personalized goals for change to support children's outdoor play within the ELCC. The RCT results partially support our hypotheses. ECEs and ELCC administrators receiving the intervention reported significantly higher increases in their tolerance of risk in play at 1-week post-intervention than participants in the control condition. These differences remained significant at 3-month post-intervention. There were no significant differences in goal attainment. These results are consistent with findings of a previous RCT testing the OutsidePlay-Parent intervention, which also found significantly greater increases of tolerance of risk in play for intervention versus control participants at 1-week and 3-month post-intervention 20.

22-ii) Highlight unanswered new questions, suggest future research
Highlight unanswered new questions, suggest future research.
subitem not at all important
1
2
3
4
5
essential
Does your paper address subitem 22-ii?
Copy and paste relevant sections from the manuscript (include quotes in quotation marks "like this" to indicate direct quotes from your manuscript), or elaborate on this item by providing additional information not in the ms, or briefly explain why the item is not applicable/relevant for your study
Your answer
"Our RCT results demonstrate that the OutsidePlay-ECE web-based intervention is effective in increasing ECEs and ELCC administrators' risk tolerance in children's outdoor play. As an easily accessible and free resource, the OutsidePlay-ECE has great potential to support early childhood education practice. For example, it can be integrated into ECE professional development, provided as a stand-alone ECE program, and revisited over time to help ECEs deepen their understanding and expand their practice related to outdoor play provision."
And,
"Perhaps future studies can test the efficiency of similar web-based risk-reframing tool under the non-pandemic normal situations."

20) Trial limitations, addressing sources of potential bias, imprecision, and, if relevant, multiplicity of analyses

20-i) Typical limitations in ehealth trials
Typical limitations in ehealth trials: Participants in ehealth trials are rarely blinded. Ehealth trials often look at a multiplicity of outcomes, increasing risk for a Type I error. Discuss biases due to non-use of the intervention/usability issues, biases through informed consent procedures, unexpected events.

subitem not at all important

1
2
3
4
5

essential

Does your paper address subitem 20-i? *
Copy and paste relevant sections from the manuscript (include quotes in quotation marks "like this" to indicate direct quotes from your manuscript), or elaborate on this item by providing additional information not in the ms, or briefly explain why the item is not applicable/relevant for your study
Your answer
See Strengths and Limitation section.

21) Generalisability (external validity, applicability) of the trial findings
NPT: External validity of the trial findings according to the intervention, comparators, patients, and care providers or centers involved in the trial

21-i) Generalizability to other populations
Generalizability to other populations: In particular, discuss generalizability to a general Internet population, outside of a RCT setting, and general patient population, including applicability of the study results for other organizations

subitem not at all important

1
2
3
4
5

essential

Does your paper address subitem 21-i?
Copy and paste relevant sections from the manuscript (include quotes in quotation marks "like this" to indicate direct quotes from your manuscript), or elaborate on this item by providing additional information not in the ms, or briefly explain why the item is not applicable/relevant for your study

Your answer
“Third, we conducted the study during the COVID-19 pandemic which included initial closures of ELCCs. The data collection period occurred after most ELCCs had resumed operations. Yet, practices remained in flux as Canadian ELCCs were receiving rapidly evolving provincial and federal guidance regarding recommended procedures while understanding and implementation of the guidance were challenging and varied between centers. While this may have provided novel insights on ECEs and ELCC administrators’ perception of children’s outdoor play in the specific context of the pandemic, the findings may have differed in other conditions..”

21-ii) Discuss if there were elements in the RCT that would be different in a routine application setting

Discuss if there were elements in the RCT that would be different in a routine application setting (e.g., prompts/reminders, more human involvement, training sessions or other co-interventions) and what impact the omission of these elements could have on use, adoption, or outcomes if the intervention is applied outside of a RCT setting.

subitem not at all important

1
2
3
4
5

essential

Does your paper address subitem 21-ii?

Copy and paste relevant sections from the manuscript (include quotes in quotation marks "like this" to indicate direct quotes from your manuscript), or elaborate on this item by providing additional information not in the ms, or briefly explain why the item is not applicable/relevant for your study

Your answer
Non-trial application setting for the OutsidePlay intervention will be different than the protocol taken in this trial. While users will be provided with a professional development certificate for 100 minutes, they will not be remunerated for taking the intervention.

OTHER INFORMATION

23) Registration number and name of trial registry

Does your paper address CONSORT subitem 23? *

Copy and paste relevant sections from the manuscript (include quotes in quotation marks "like this" to indicate direct quotes from your manuscript), or elaborate on this item by providing additional information not in the ms, or briefly explain why the item is not applicable/relevant for your study

Your answer
ClinicalTrials.gov PRS – NCT04624932

24) Where the full trial protocol can be accessed, if available

Does your paper address CONSORT subitem 24? *
Cite a Multimedia Appendix, other reference, or copy and paste relevant sections from the manuscript (include quotes in quotation marks "like this" to indicate direct quotes from your manuscript), or elaborate on this item by providing additional information not in the ms, or briefly explain why the item is not applicable/relevant for your study
Your answer
https://www.researchprotocols.org/2021/11/e31041/

25) Sources of funding and other support (such as supply of drugs), role of funders

Does your paper address CONSORT subitem 25? *
Copy and paste relevant sections from the manuscript (include quotes in quotation marks "like this" to indicate direct quotes from your manuscript), or elaborate on this item by providing additional information not in the ms, or briefly explain why the item is not applicable/relevant for your study
Your answer
This RCT study was funded by the Lawson Foundation, Grant #GRT 2019-79.

X27) Conflicts of Interest (not a CONSORT item)

X27-i) State the relation of the study team towards the system being evaluated
In addition to the usual declaration of interests (financial or otherwise), also state the relation of the study team towards the system being evaluated, i.e., state if the authors/evaluators are distinct from or identical with the developers/sponsors of the intervention.

subitem not at all important

1
2
3
4
5

essential

Does your paper address subitem X27-i?
Copy and paste relevant sections from the manuscript (include quotes in quotation marks "like this" to indicate direct quotes from your manuscript), or elaborate on this item by providing additional information not in the ms, or briefly explain why the item is not applicable/relevant for your study
Your answer
There is no conflict of interest.

About the CONSORT EHEALTH checklist

As a result of using this checklist, did you make changes in your manuscript? *
yes, major changes
yes, minor changes
no

What were the most important changes you made as a result of using this checklist?
Your answer
On research methods and results sections.

How much time did you spend on going through the checklist INCLUDING making changes in your manuscript *
Your answer
10 hours.

As a result of using this checklist, do you think your manuscript has improved? *
Yes

Would you like to become involved in the CONSORT EHEALTH group?
This would involve for example becoming involved in participating in a workshop and writing an "Explanation and Elaboration" document
No

Any other comments or questions on CONSORT EHEALTH
Your answer
No

STOP - Save this form as PDF before you click submit
To generate a record that you filled in this form, we recommend to generate a PDF of this page (on a Mac, simply select "print" and then select "print as PDF") before you submit it.

When you submit your (revised) paper to JMIR, please upload the PDF as supplementary file.

Don't worry if some text in the textboxes is cut off, as we still have the complete information in our database. Thank you!

Final step: Click submit !
Click submit so we have your answers in our database!

Submit

Never submit passwords through Google Forms.
This content is neither created nor endorsed by Google. Report Abuse - Terms of Service - Privacy Policy

Forms
