# Supplementary material for: Evaluation of the Web-Based OutsidePlay-ECE Intervention to Influence Early Childhood Educators’ Attitudes and Supportive Behaviors Toward Outdoor Play: Randomized Controlled Trial
Source: J Med Internet Res. 2022 Jun 10;24(6):e36826. doi: 10.2196/36826 (PMC9233267; doi:10.2196/36826)
Supplement: Multimedia Appendix 2 [file jmir_v24i6e36826_app2.pdf]

Chapter 1:

Reflection

Welcome to the start of your 3-part journey! You'll be thinking about how you support outdoor play now and some of the ways you would want to change or add to your practice. Your journey will be unique to you and based on your responses to the questions. When you finish, you will:

- understand why outdoor play is important
- know strategies for dealing with common challenges
- develop your own plan for supporting outdoor play at your centre
- access more resources to help you on your journey

CONTINUE

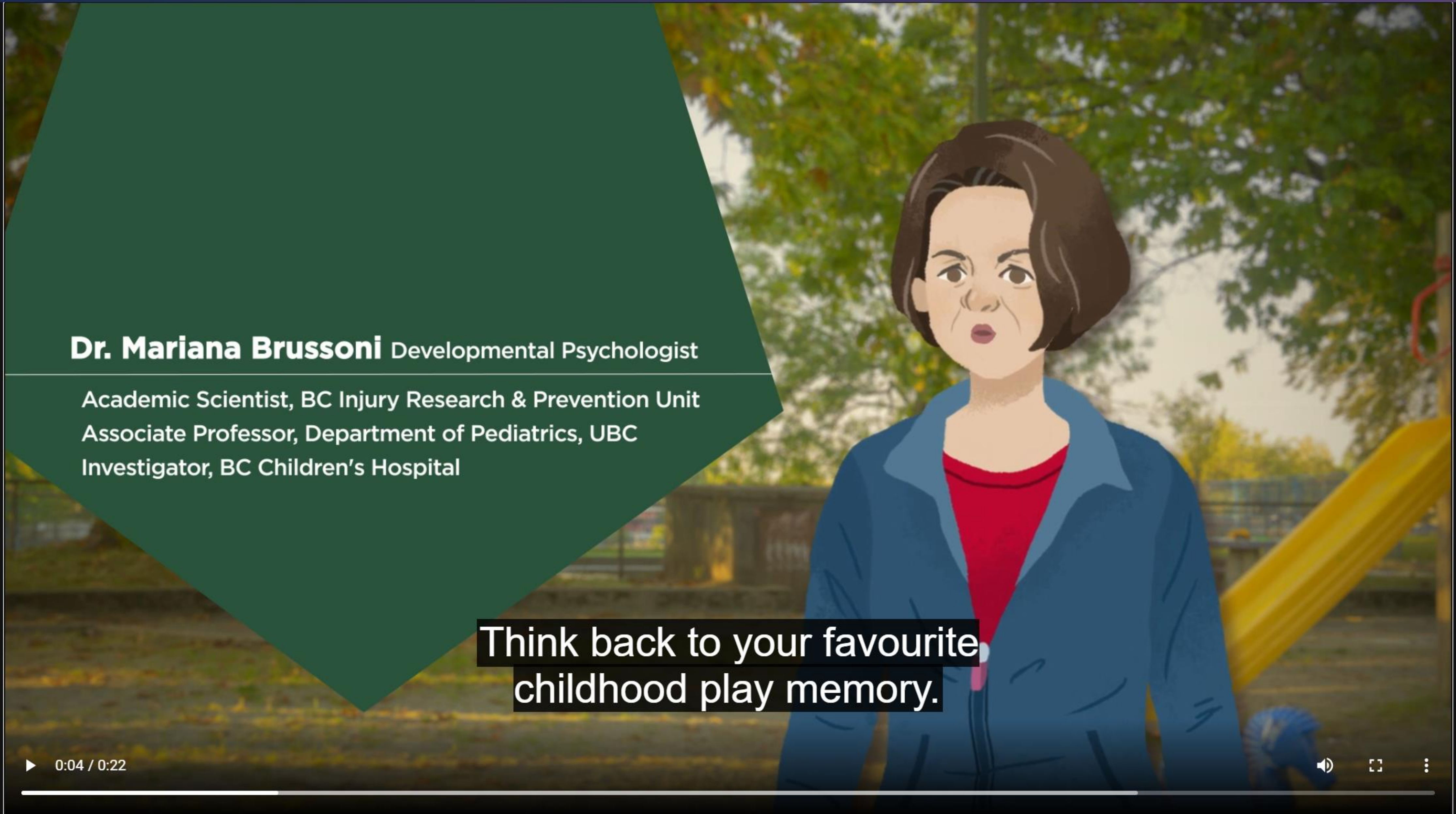
Dr. Mariana Brussoni Developmental Psychologist
Academic Scientist, BC Injury Research & Prevention Unit
Associate Professor, Department of Pediatrics, UBC
Investigator, BC Children's Hospital
Think back to your favourite childhood play memory.
0:04 / 0:22

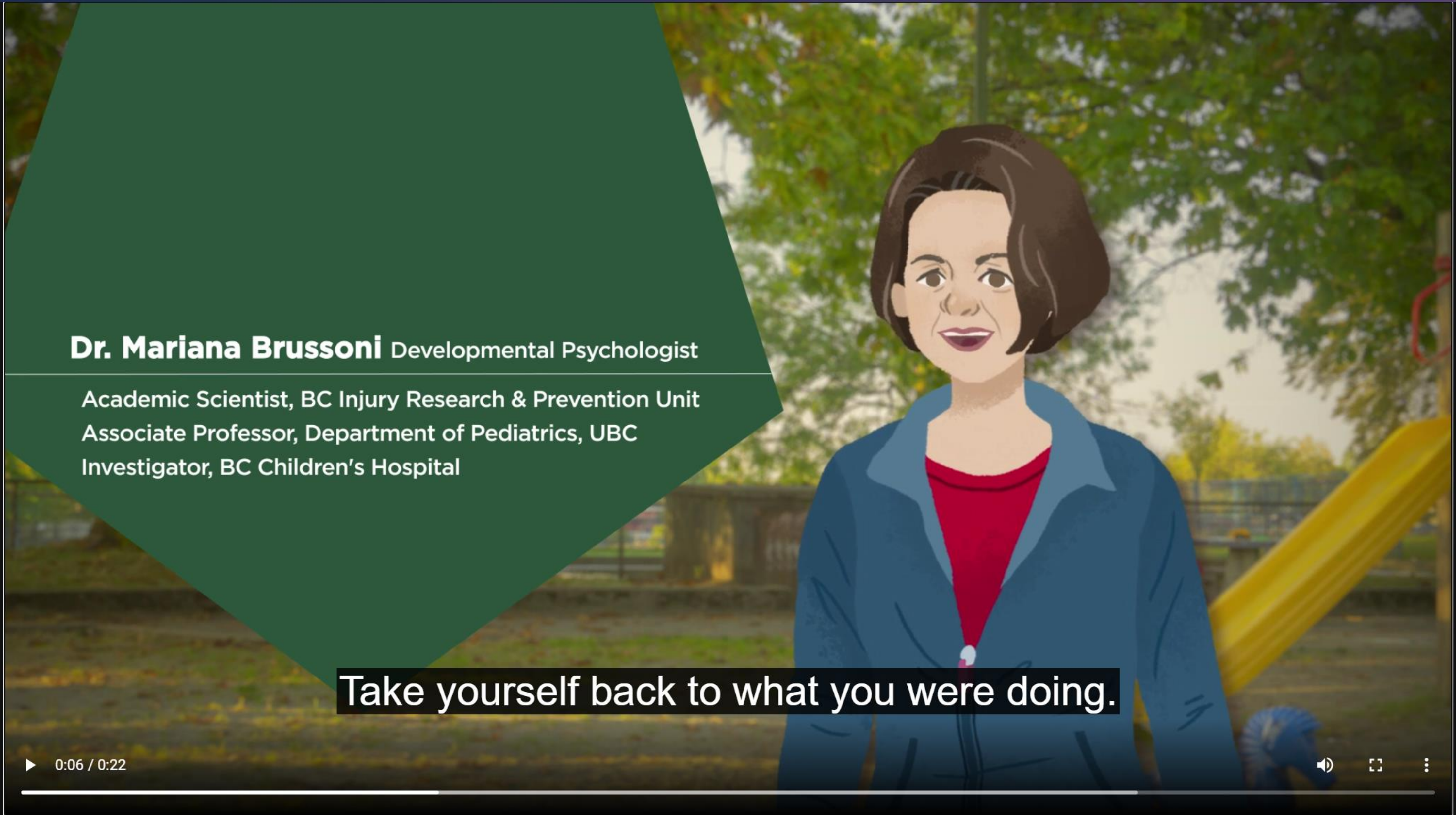
Dr. Mariana Brussoni Developmental Psychologist
Academic Scientist, BC Injury Research & Prevention Unit
Associate Professor, Department of Pediatrics, UBC
Investigator, BC Children's Hospital
Take yourself back to what you were doing.
0:06 / 0:22

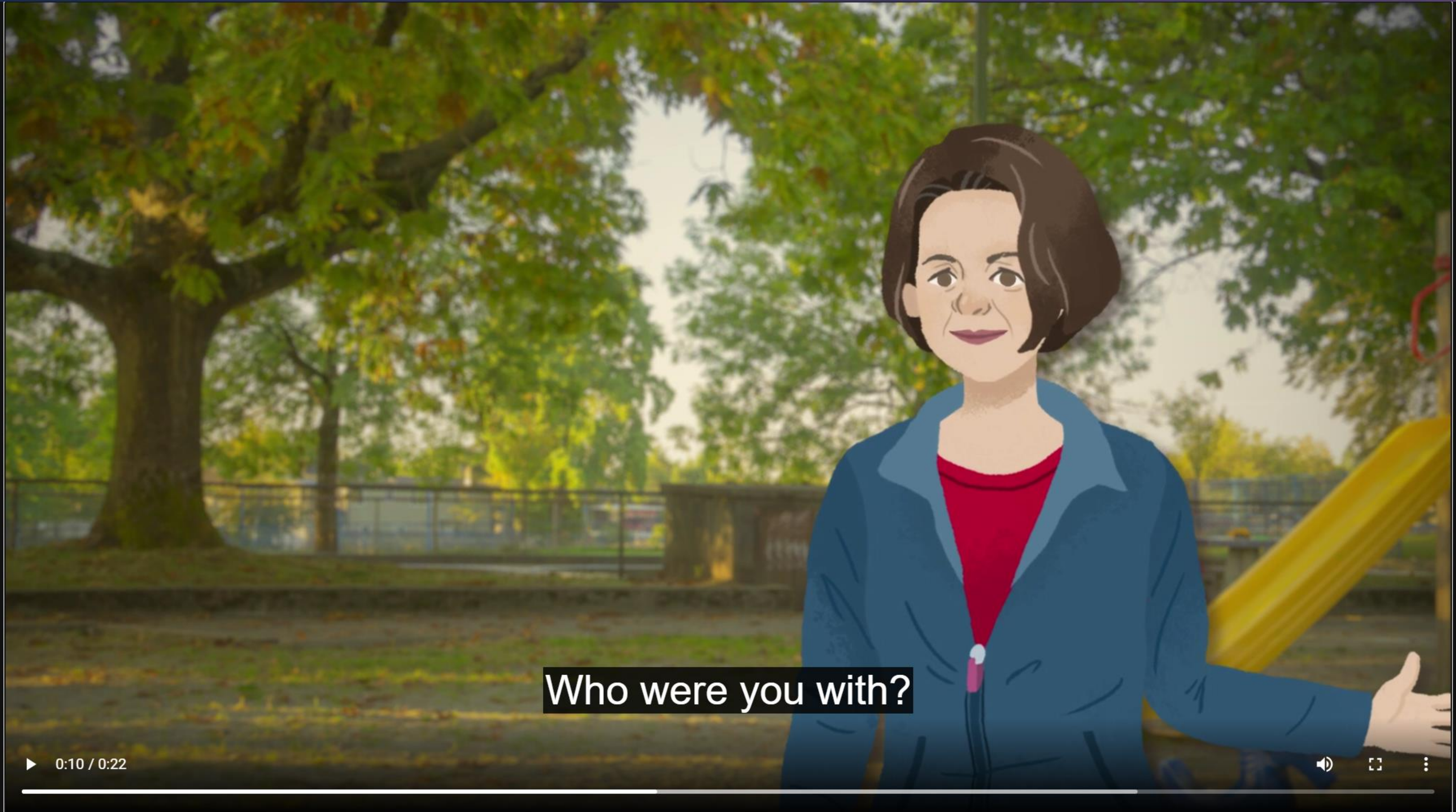
Who were you with?
0:10 / 0:22

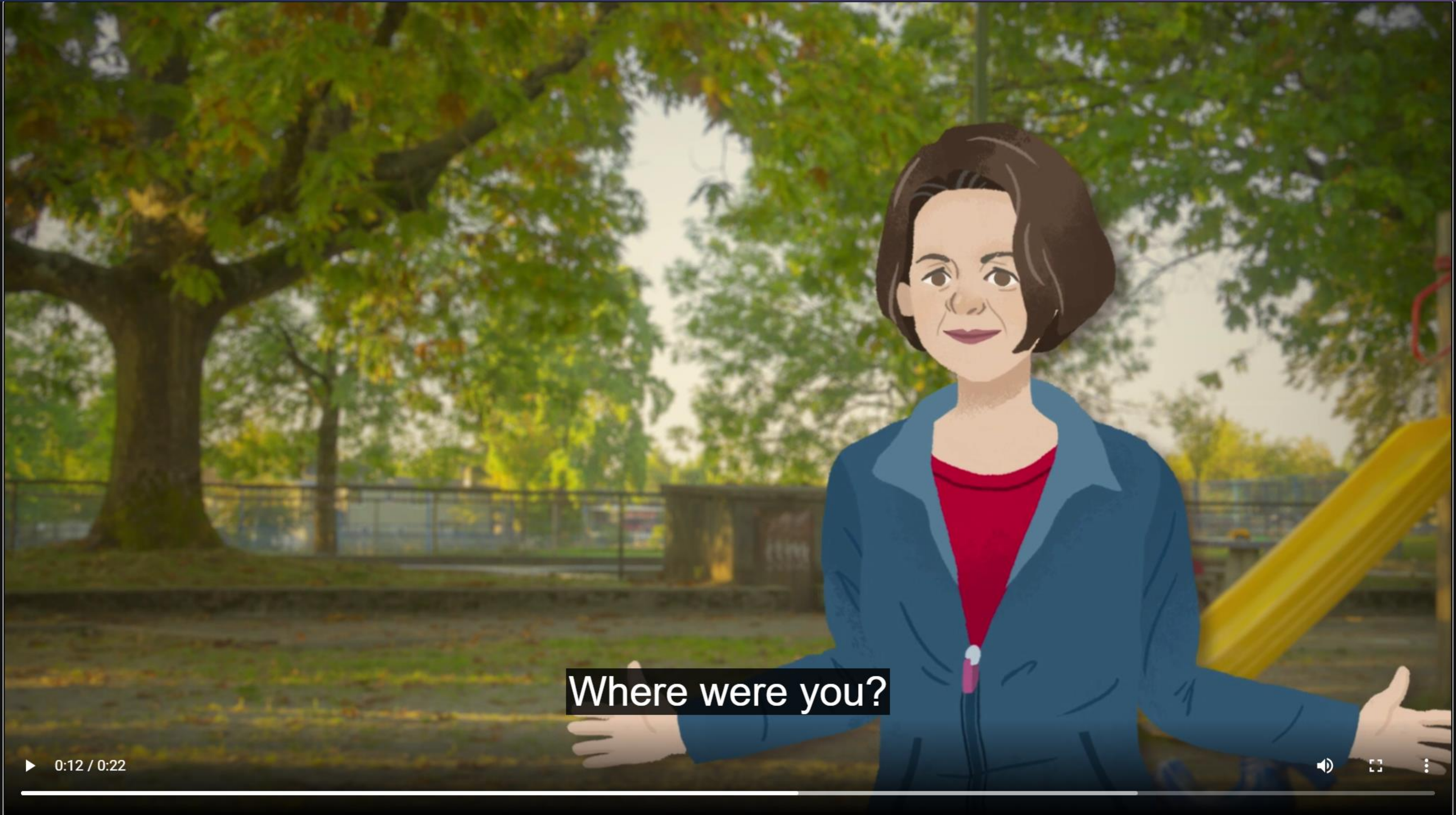
Where were you?
0:12 / 0:22

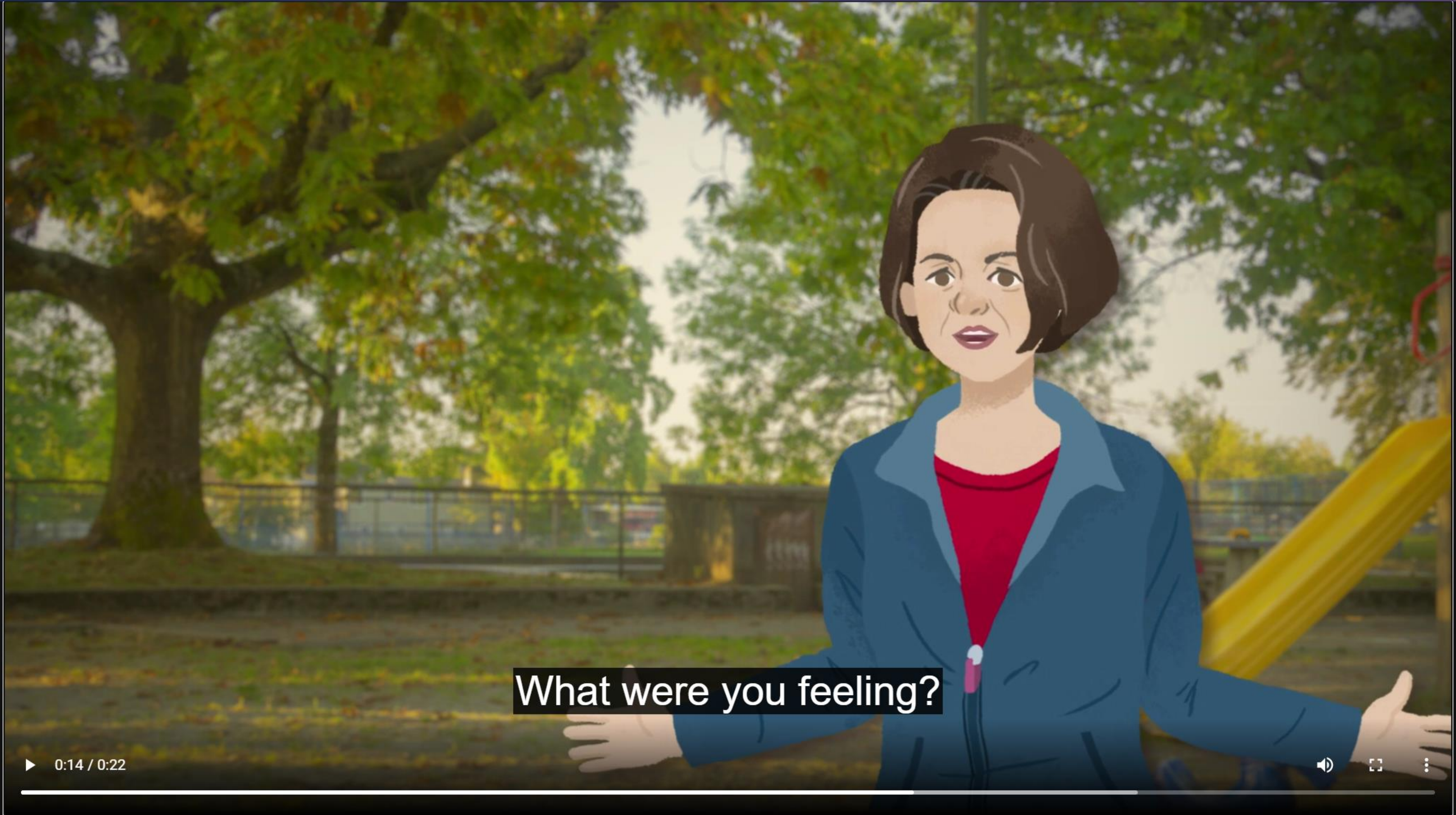
What were you feeling?
0:14 / 0:22

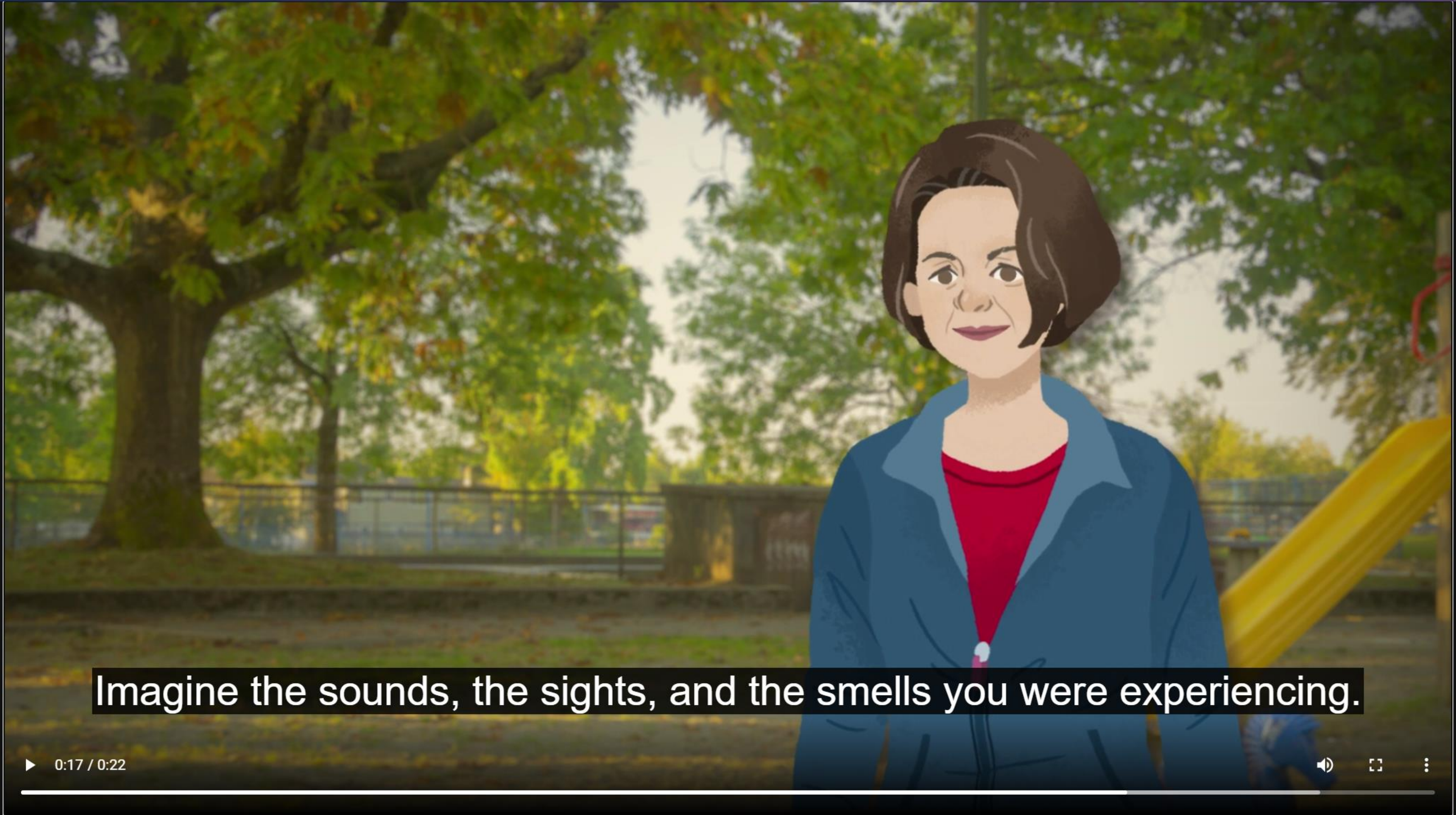
Imagine the sounds, the sights, and the smells you were experiencing.
0:17 / 0:22

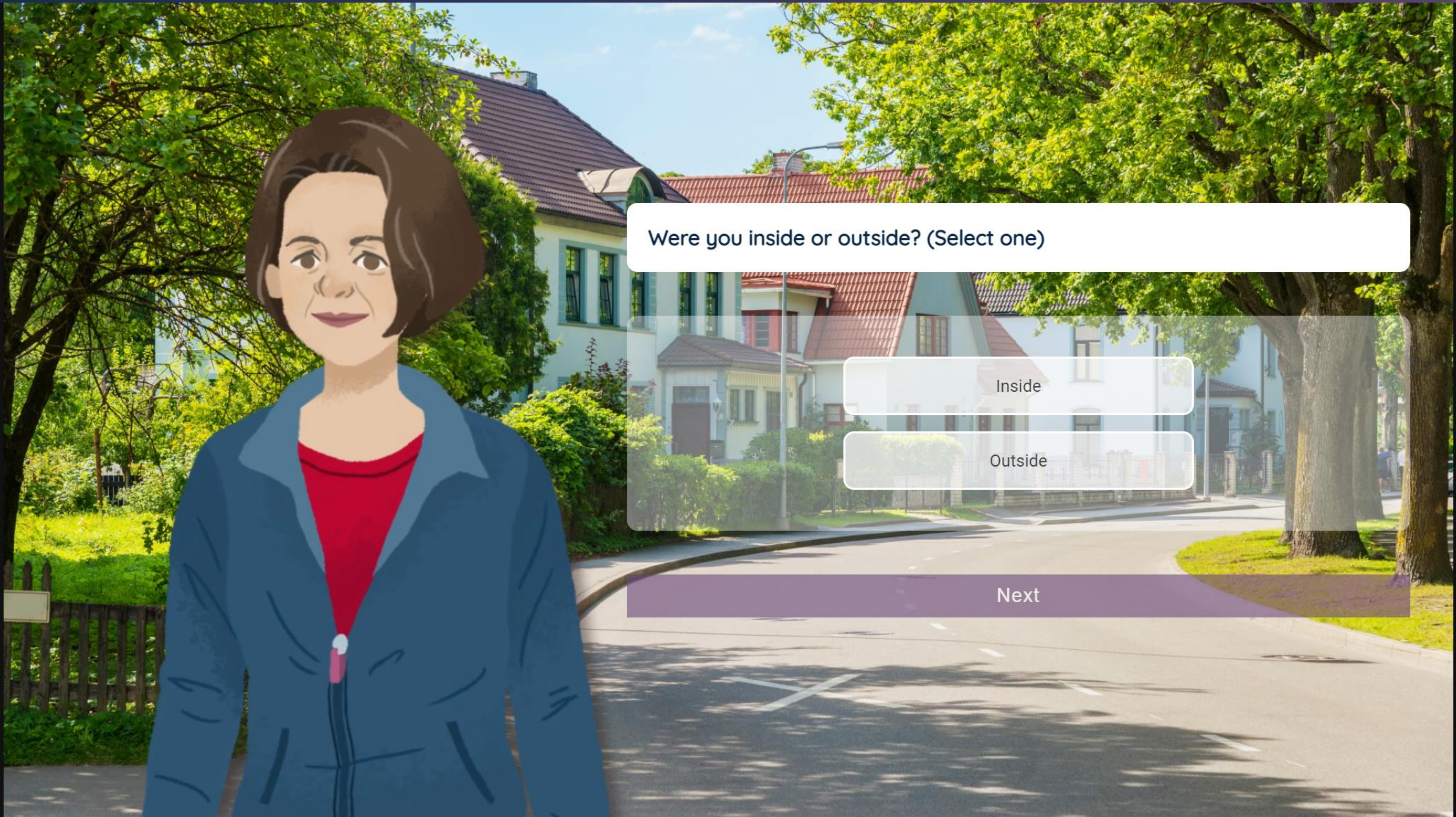
Were you inside or outside? (Select one)
Inside
Outside
Next

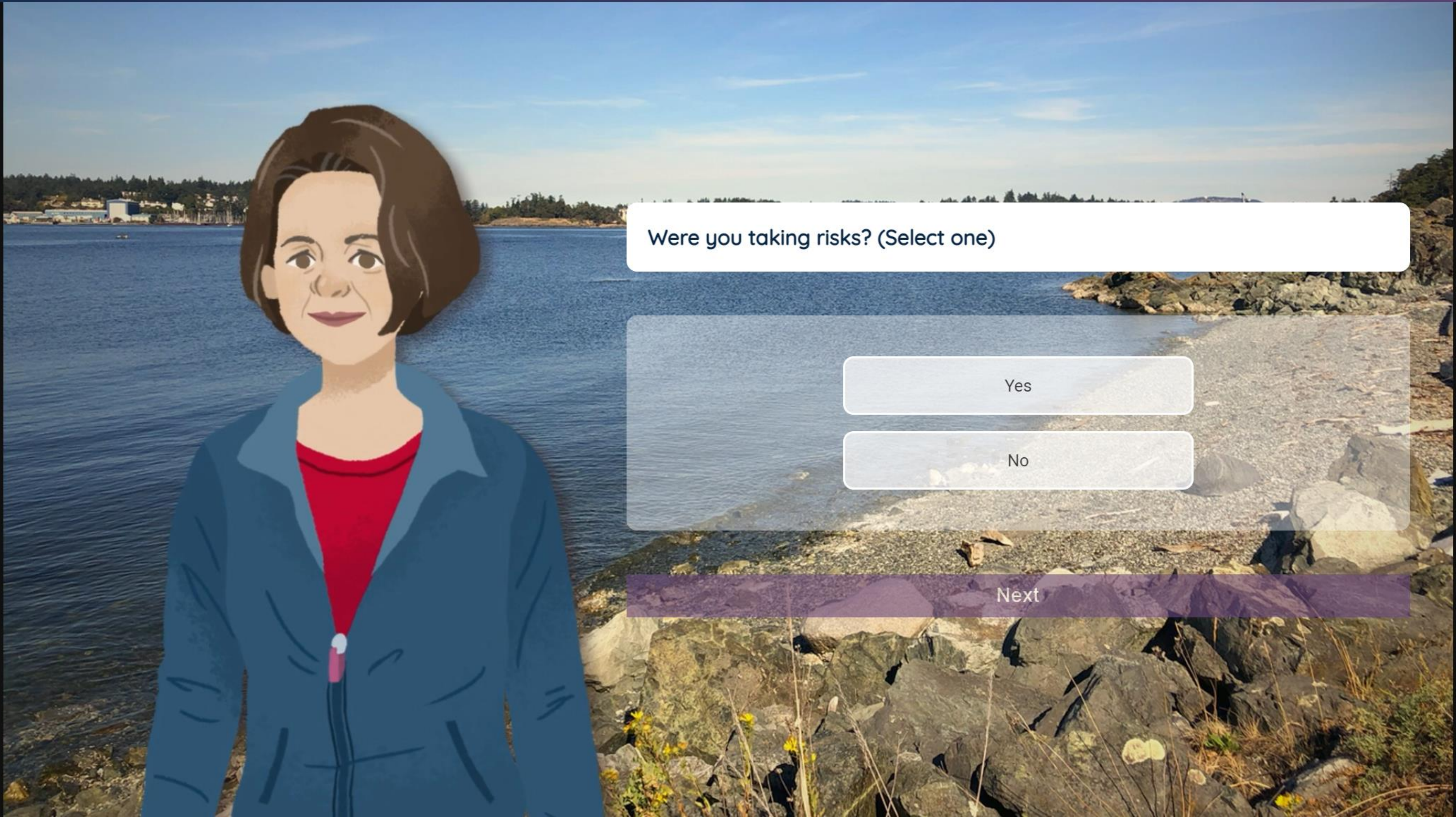

Were you taking risks? (Select one)

Yes

No

Next

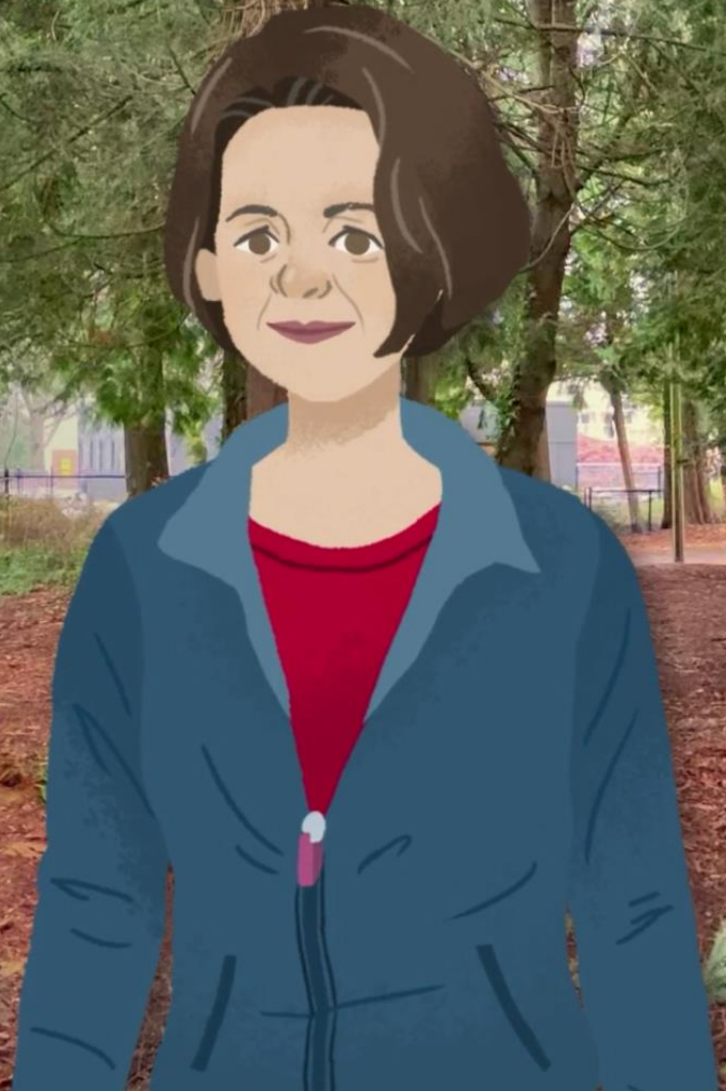

What was your favourite thing to do? (Select all that apply)

- Playing group games, such as tag or hide-and-seek
- Cycling, skateboarding, or scootering
- Building things with loose parts and tools
- Climbing trees or structures
- Running
- Jumping
- Playing on playground equipment
- Playing with natural elements, such as sand, sticks, mud
- Exploring nature
- Imaginative play
- Daydreaming
- Reading
- Playing on screens, such as playing video games
- Other

Next

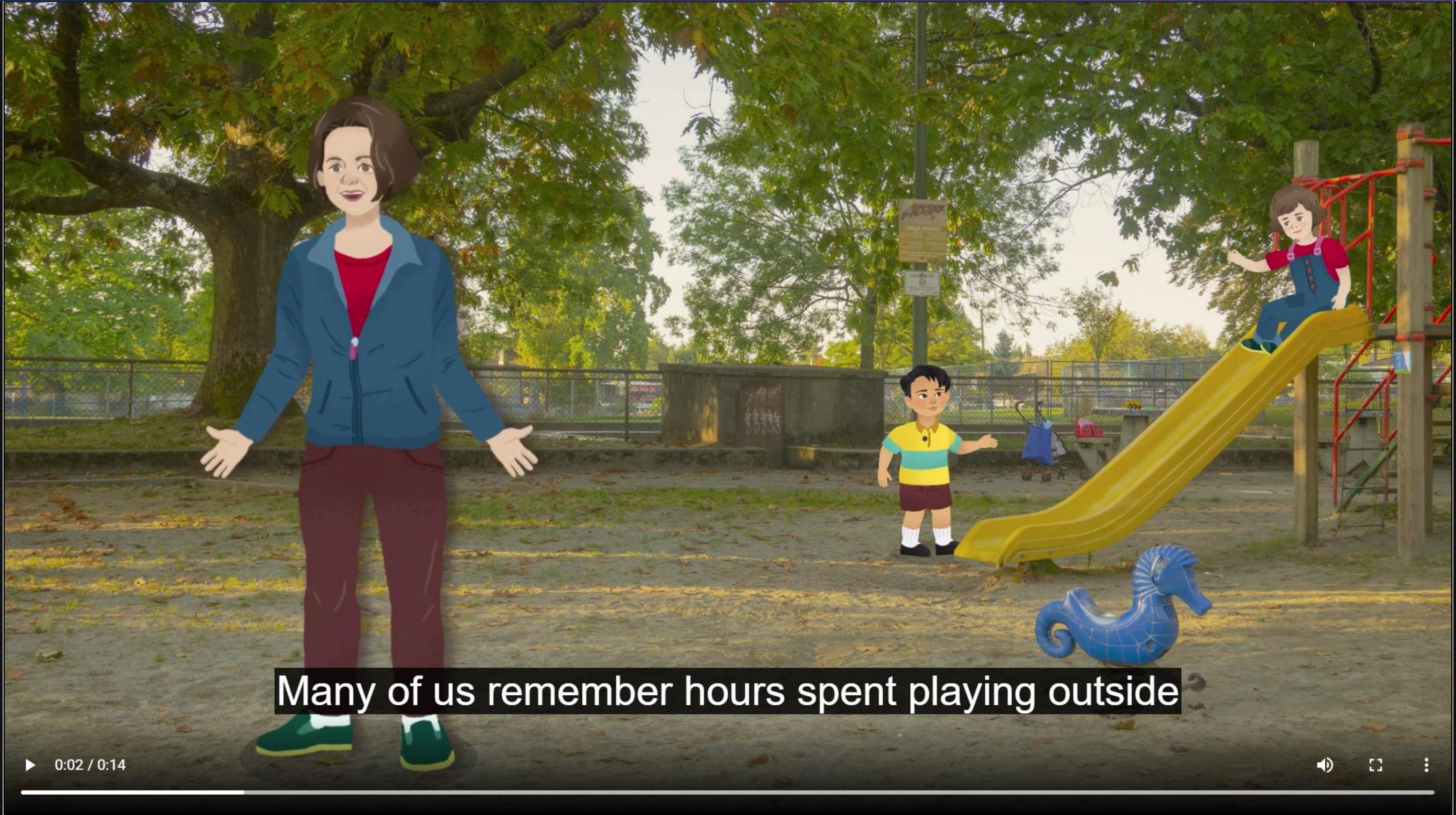
Many of us remember hours spent playing outside
0:02 / 0:14

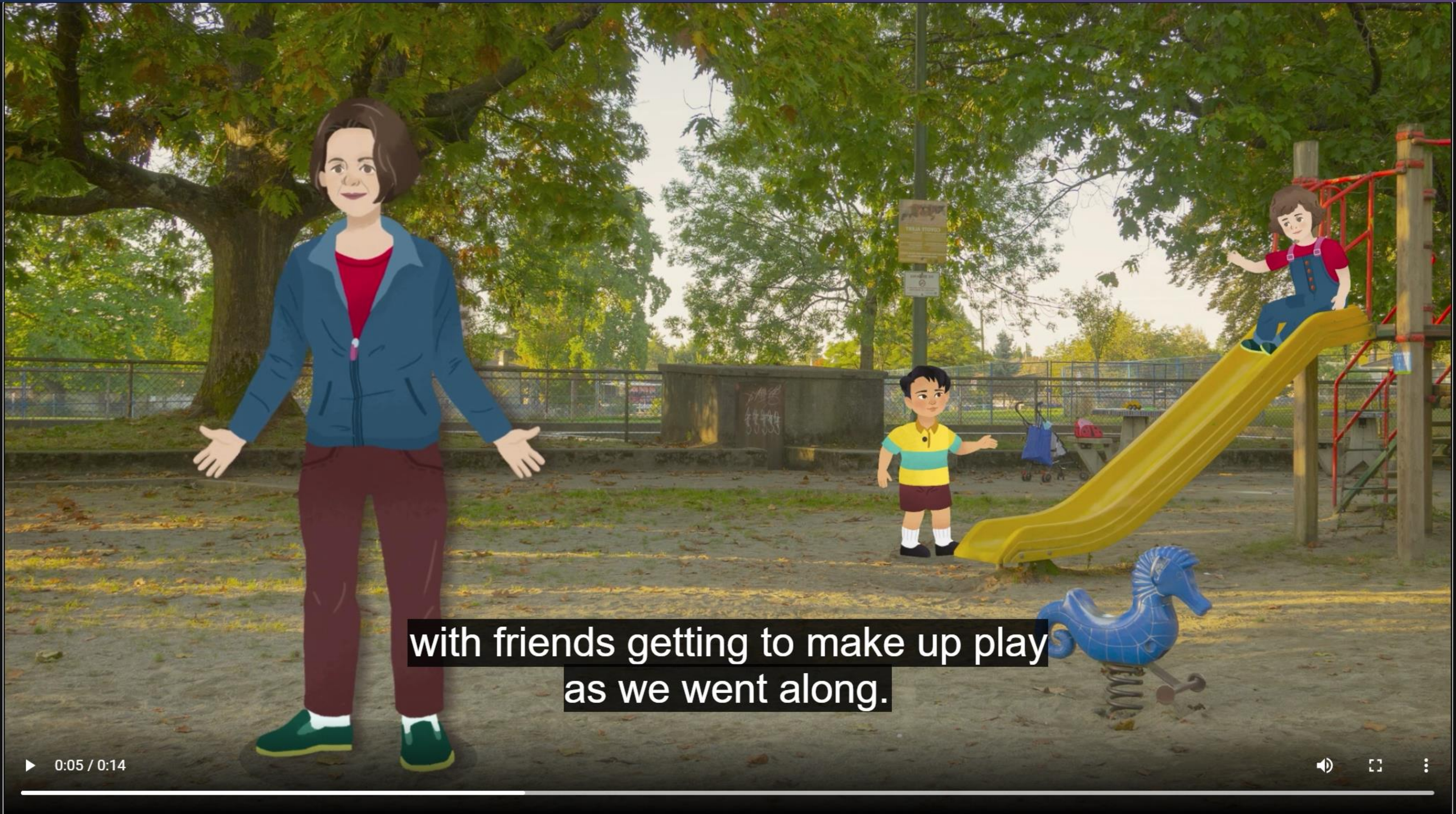
with friends getting to make up play
as we went along.
0:05 / 0:14

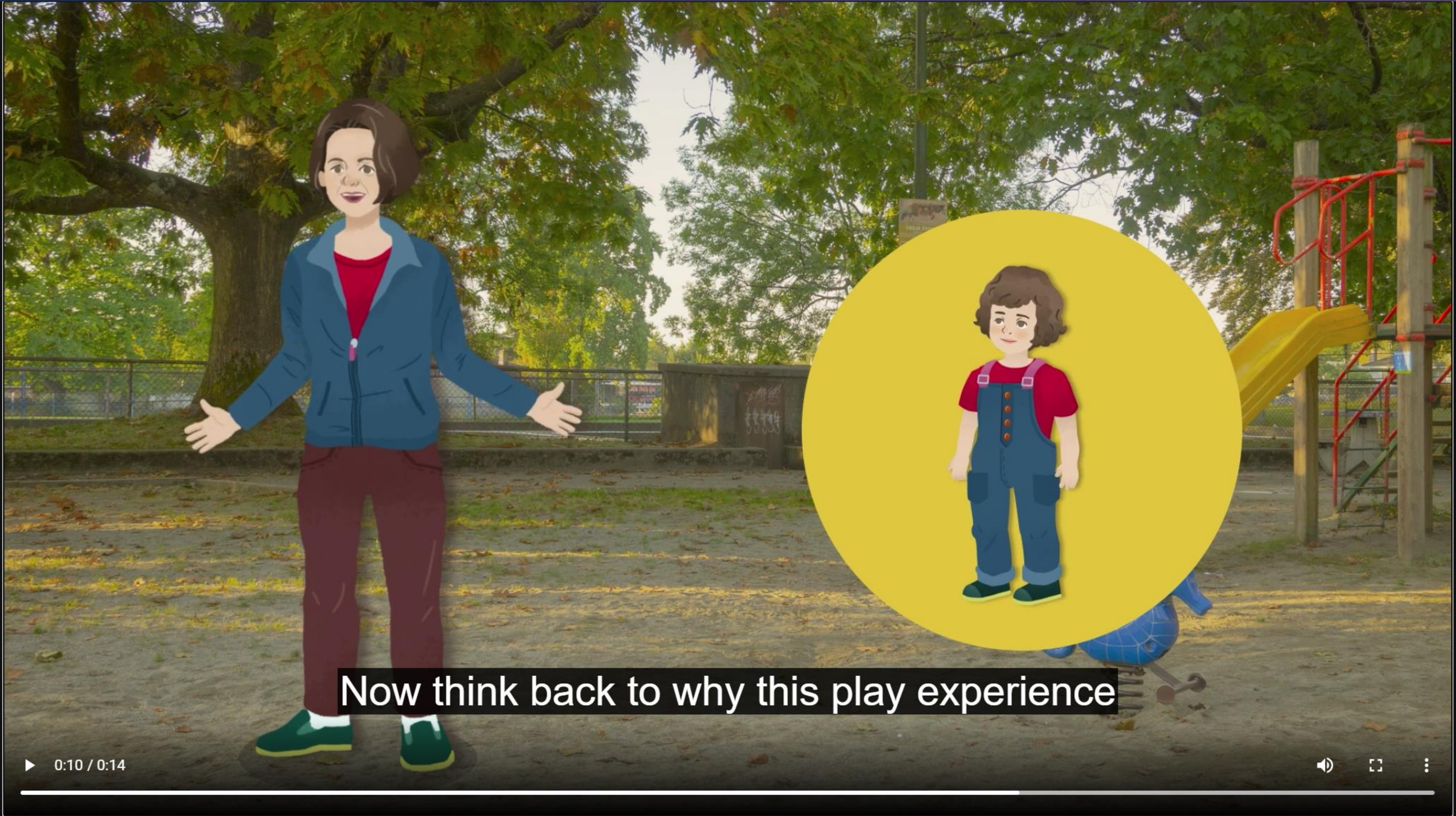
Now think back to why this play experience
0:10 / 0:14

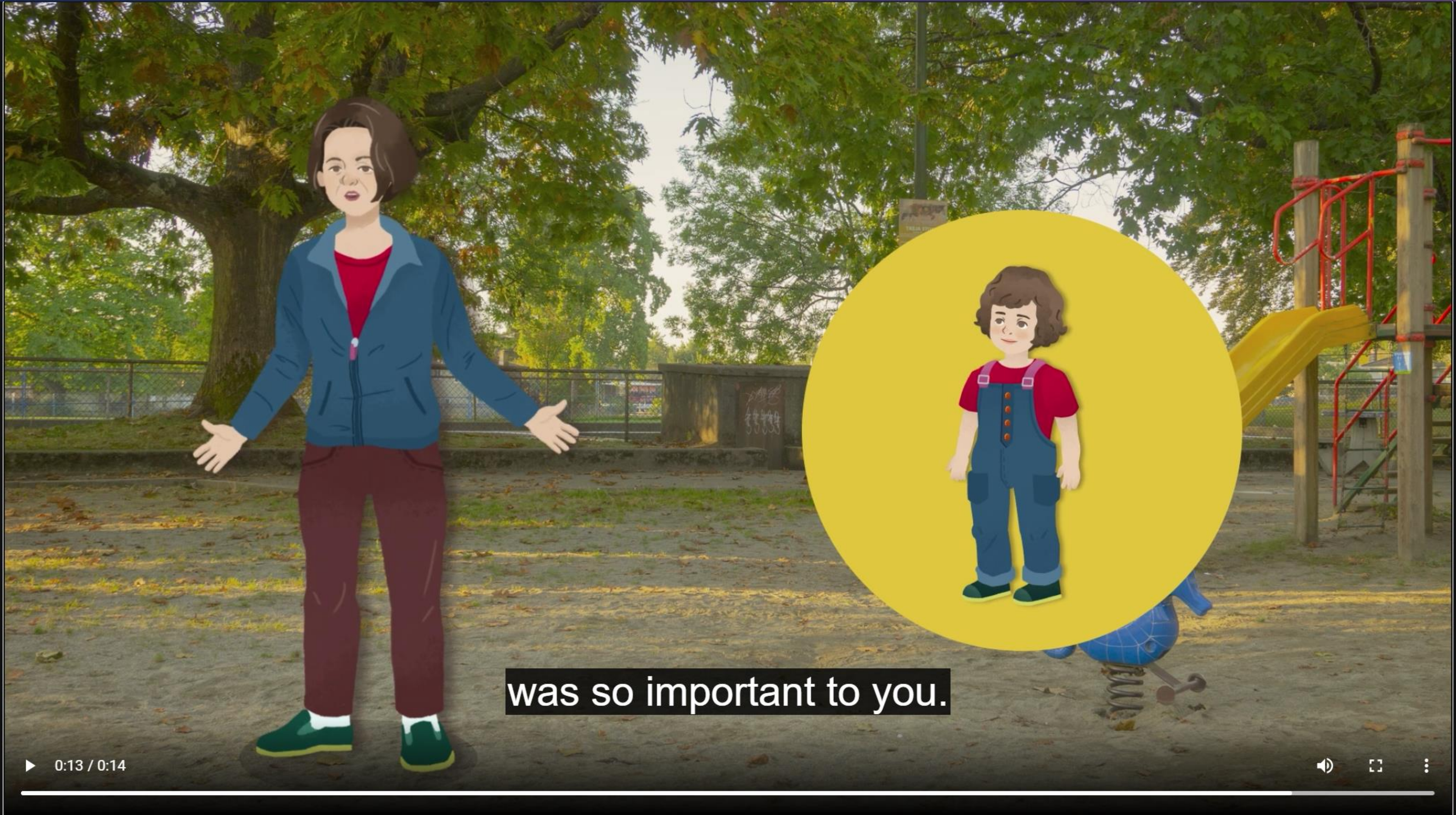
was so important to you.
0:13 / 0:14

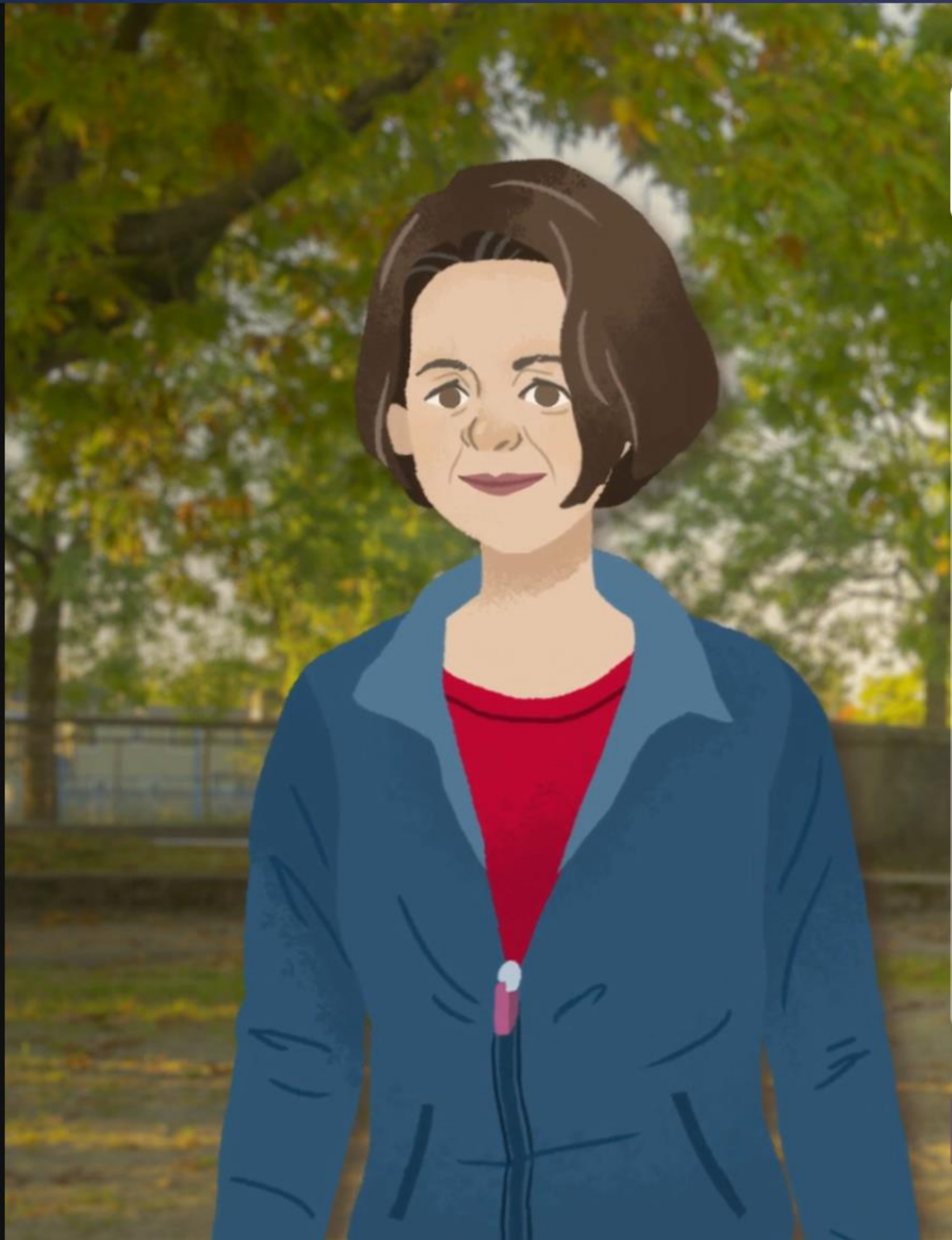

What did you get out of it? How did this experience influence you? (Select all that apply)

Confidence	Connection to the land	Courage
Creativity	Curiosity	Empathy
Independence	Joy	Leadership
Physical competence	Problem-solving	Resilience
Sense of belonging	Sense of freedom	Spirituality

Next

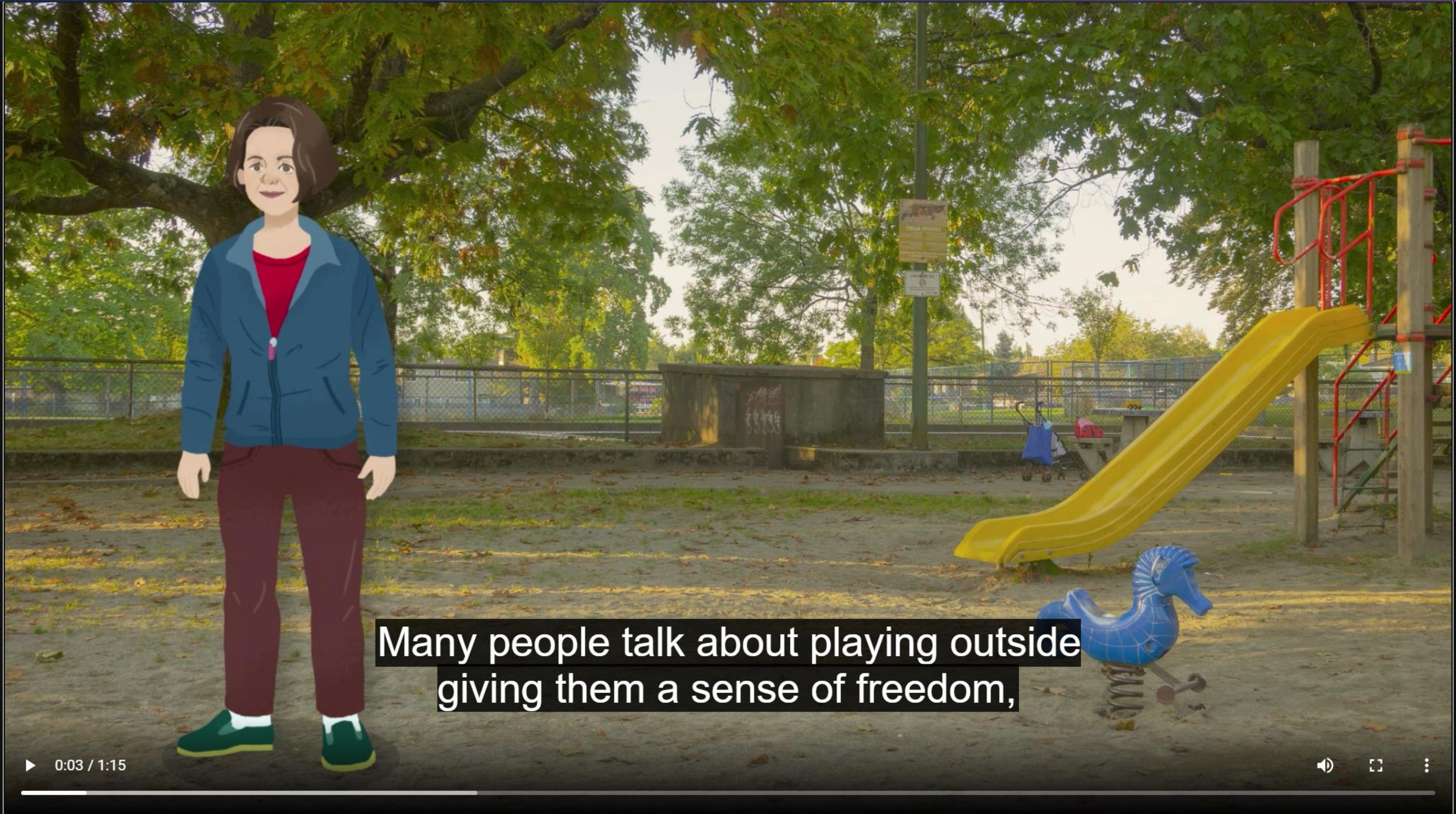
Many people talk about playing outside
giving them a sense of freedom,
0:03 / 1:15

Take a risk, go play outside!

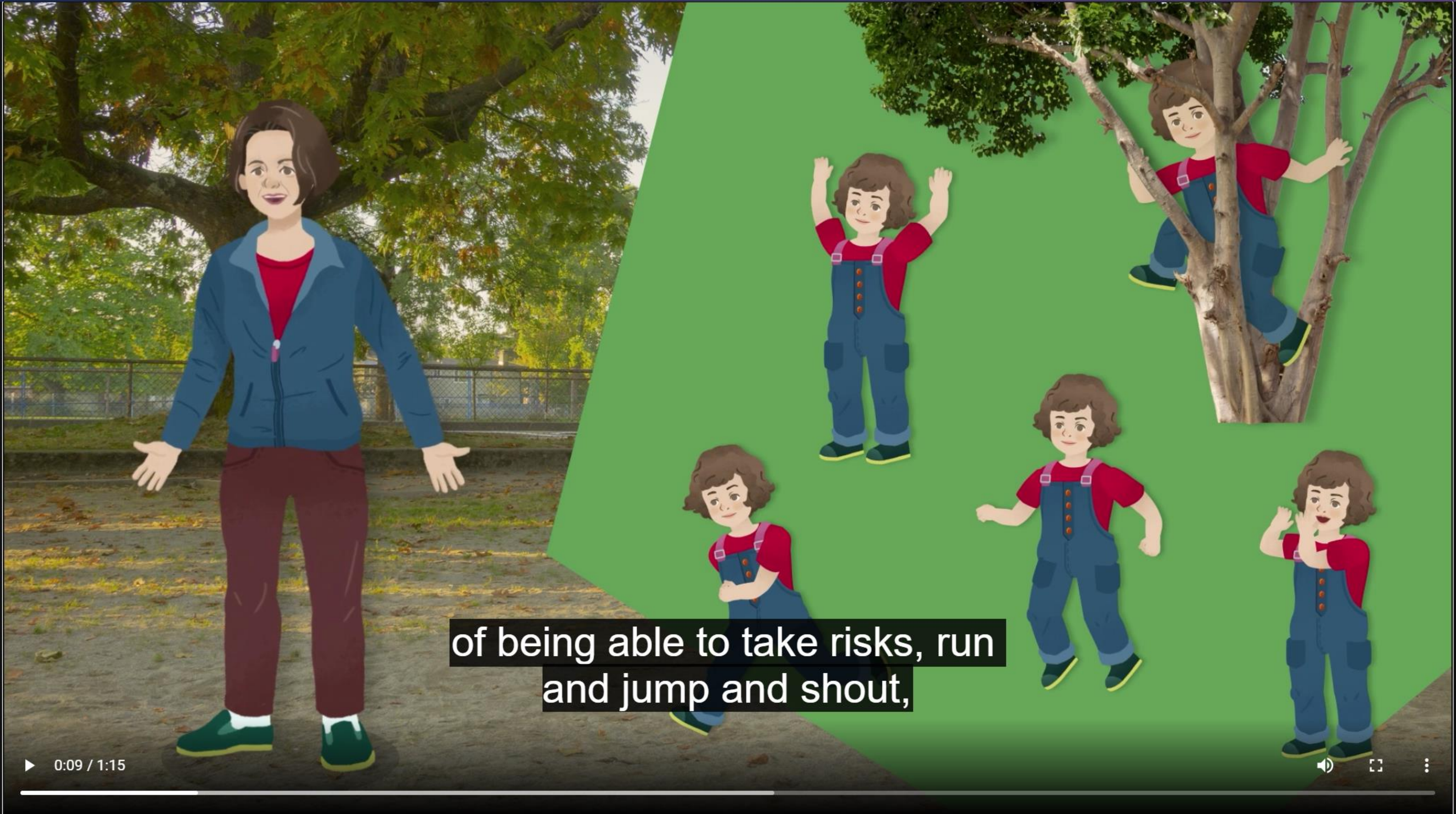

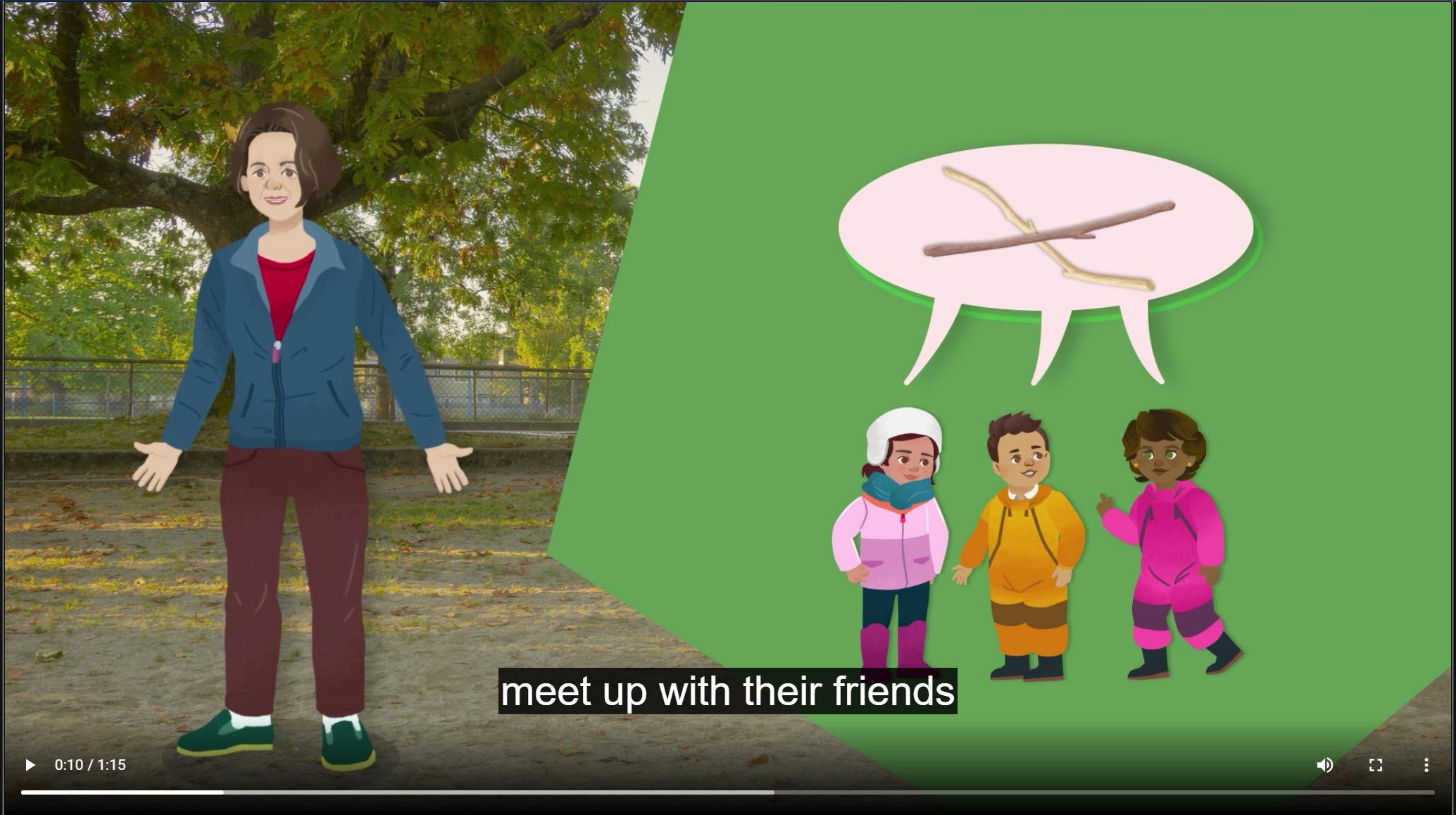
meet up with their friends
0:10 / 1:15

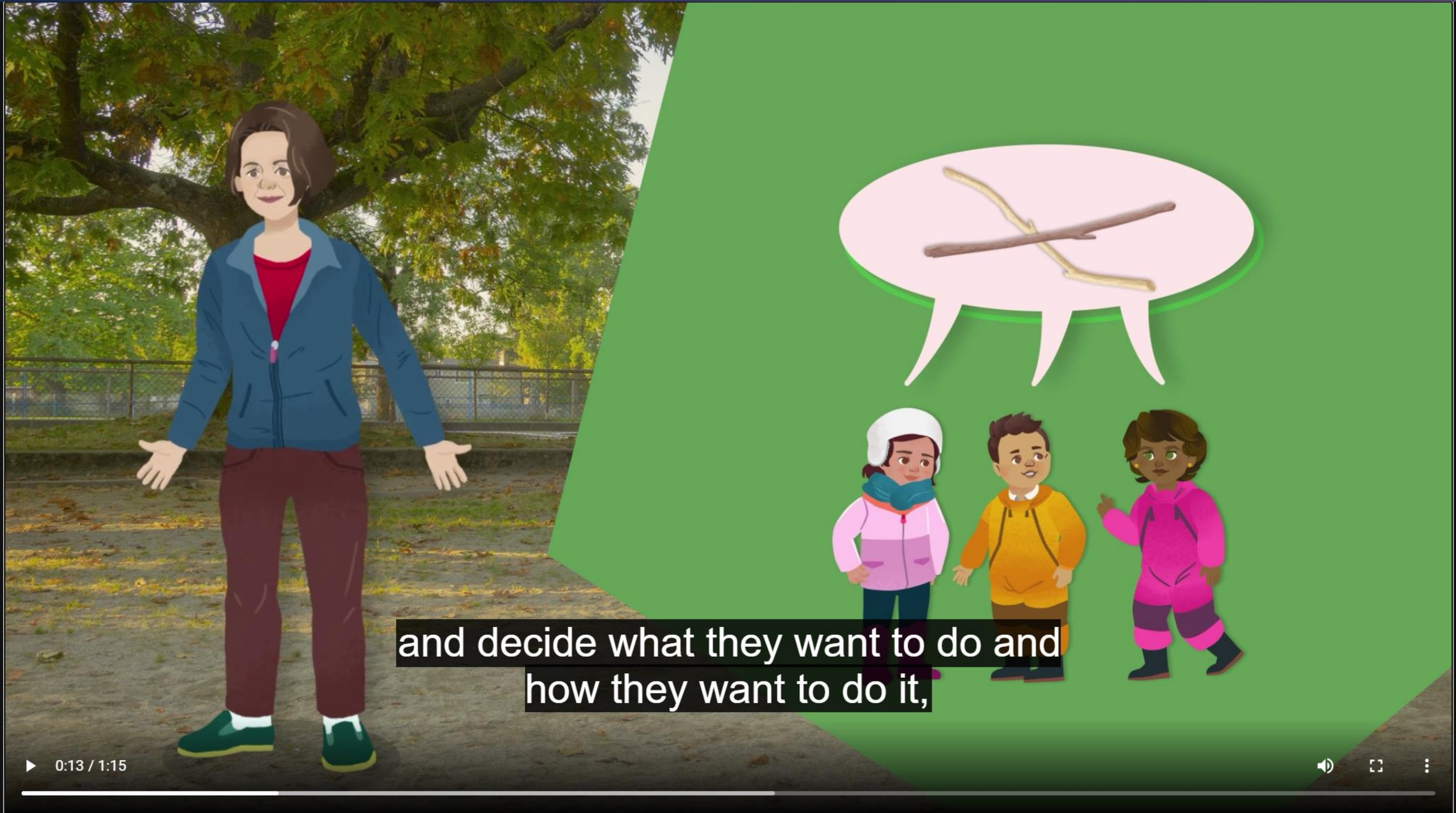
and decide what they want to do and
how they want to do it,
0:13 / 1:15

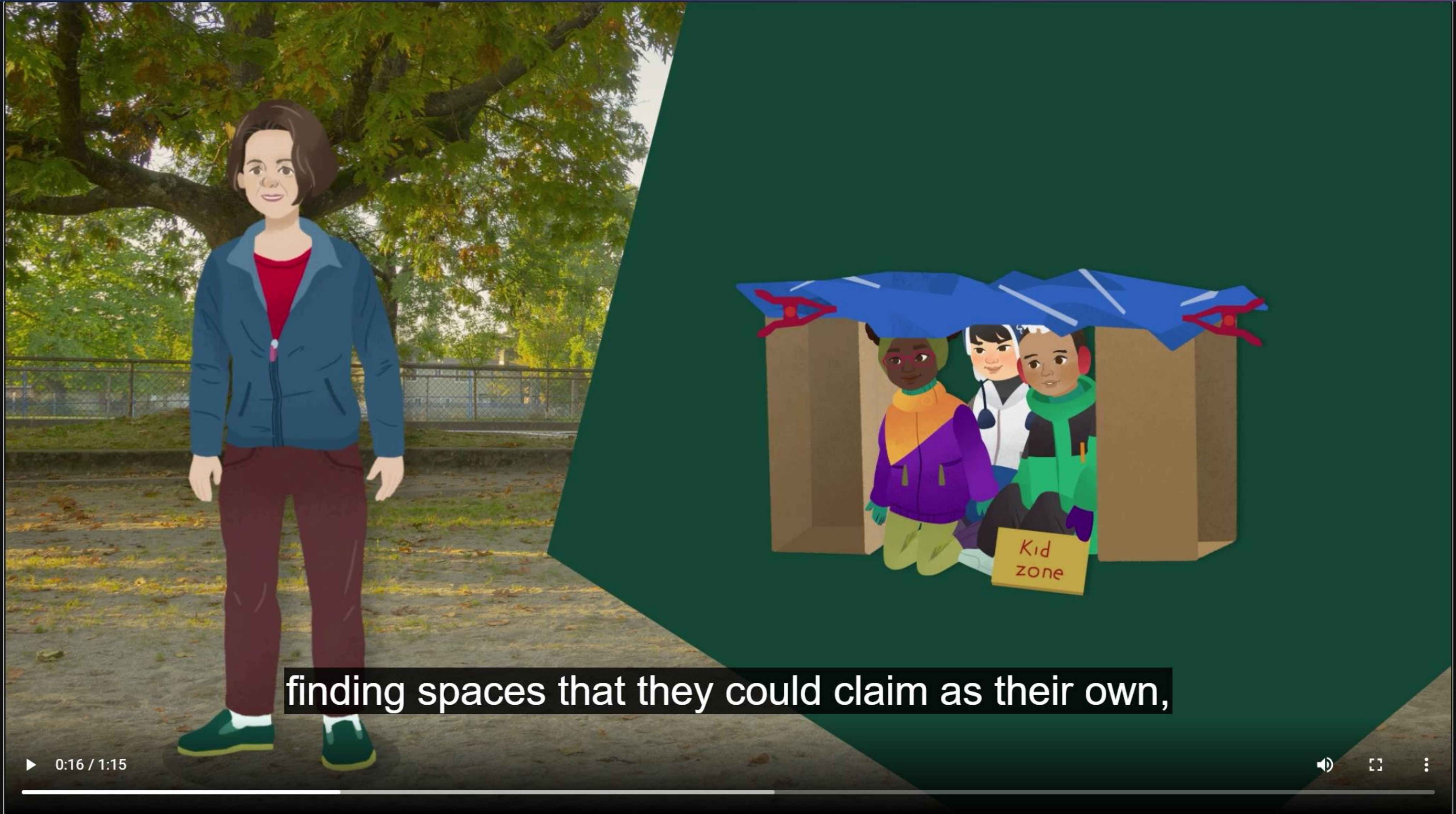
Kid zone
finding spaces that they could claim as their own,
0:16 / 1:15

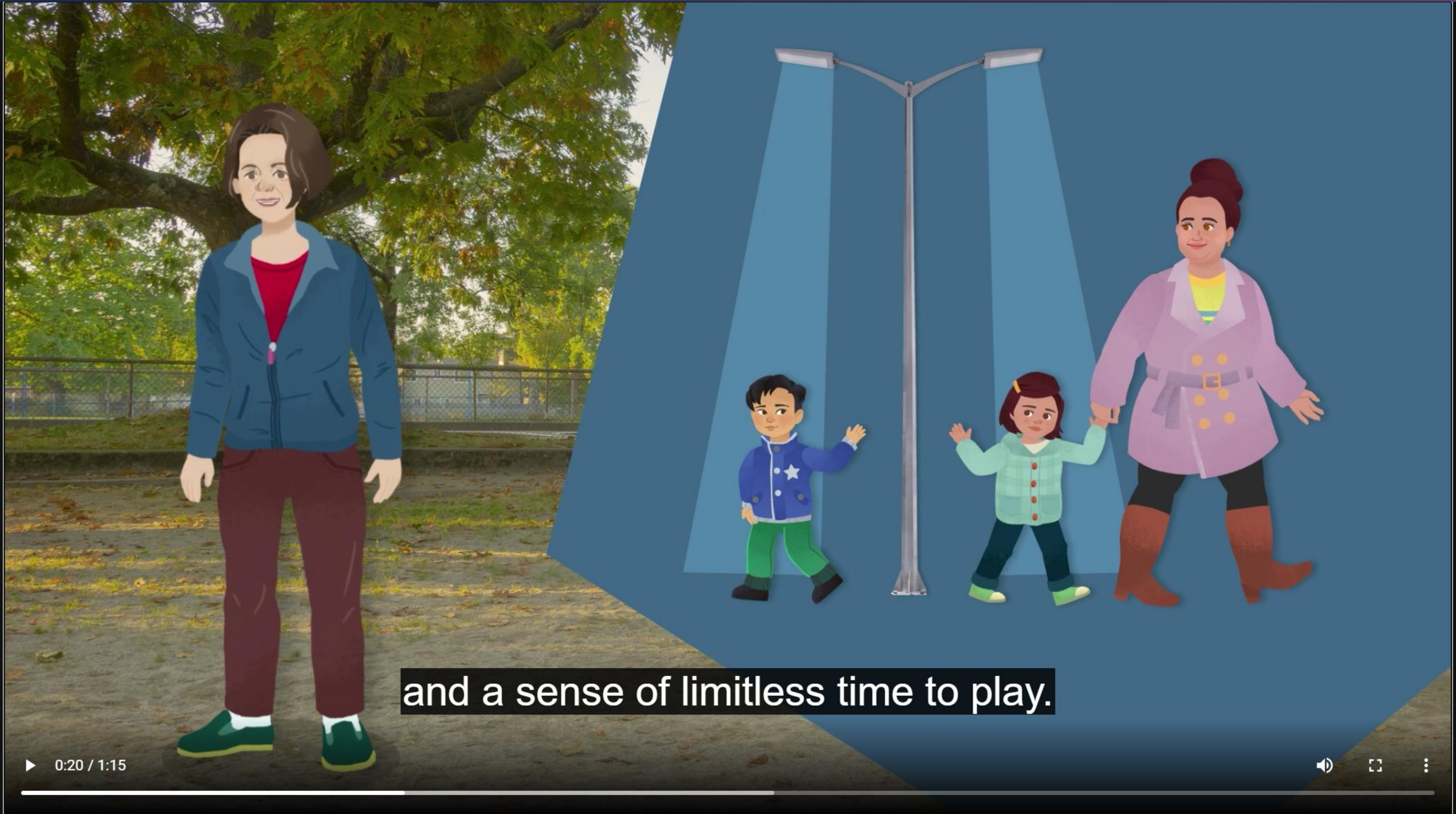
and a sense of limitless time to play.
0:20 / 1:15

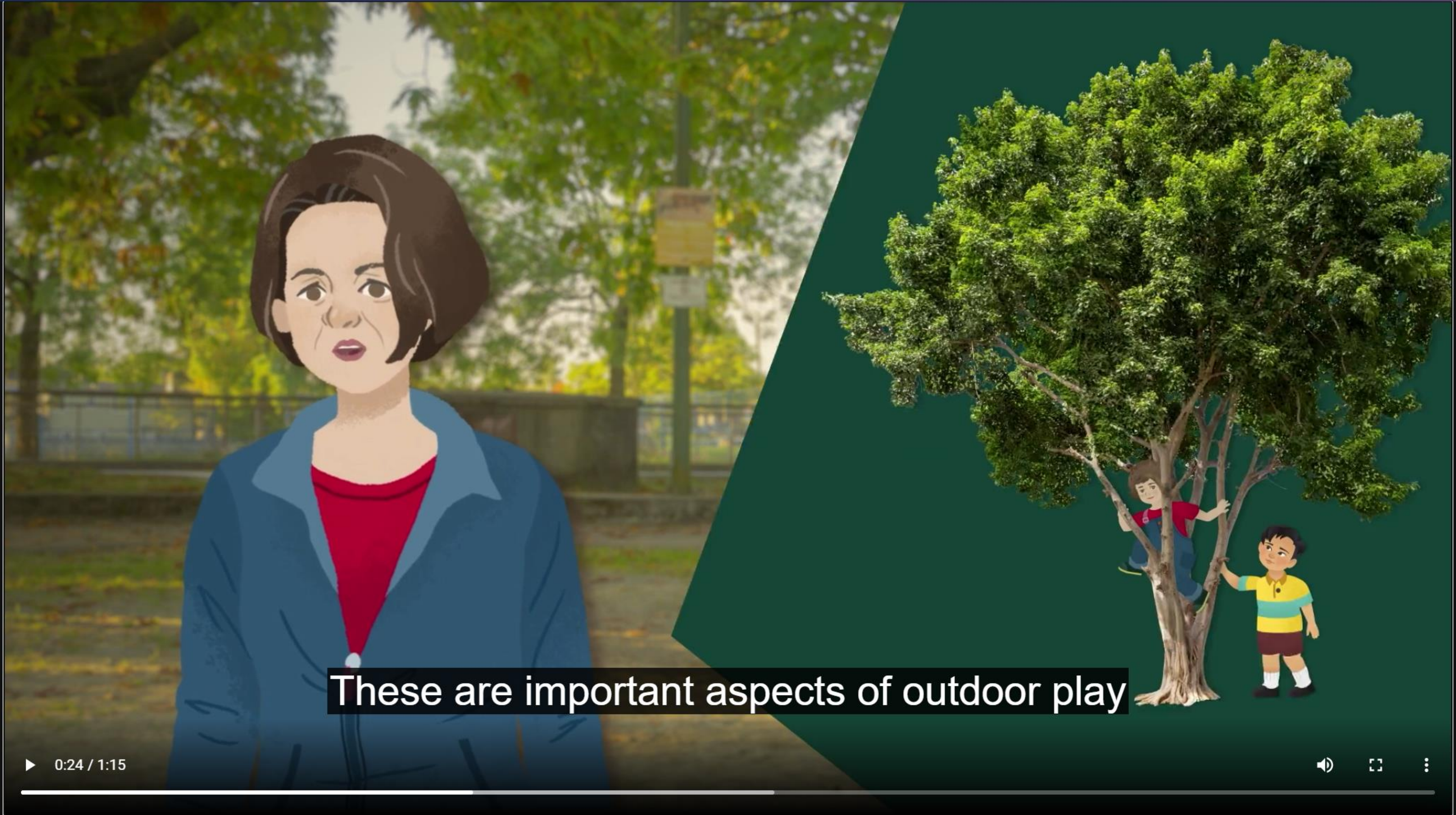
These are important aspects of outdoor play
0:24 / 1:15

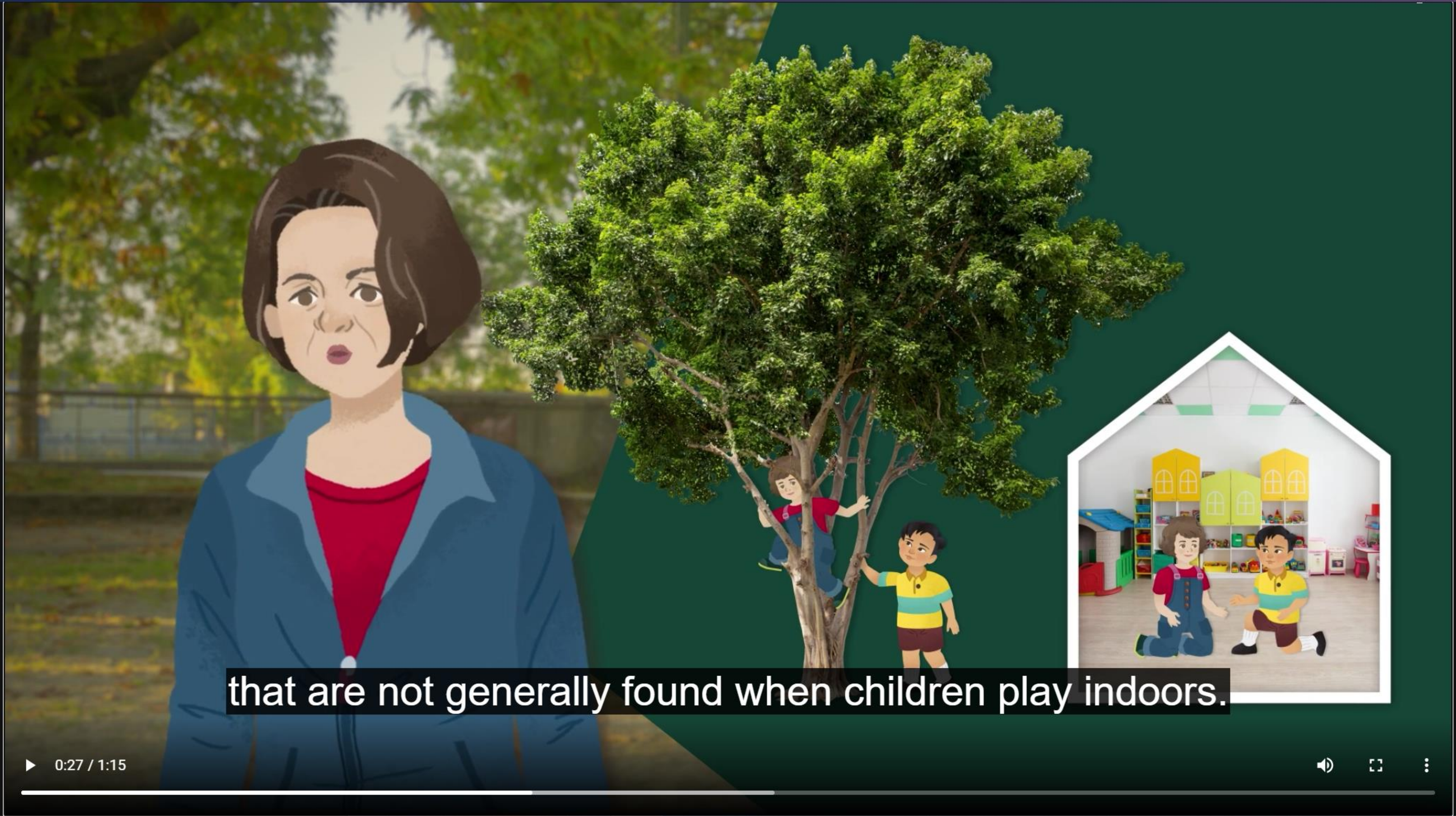
that are not generally found when children play indoors.
0:27 / 1:15

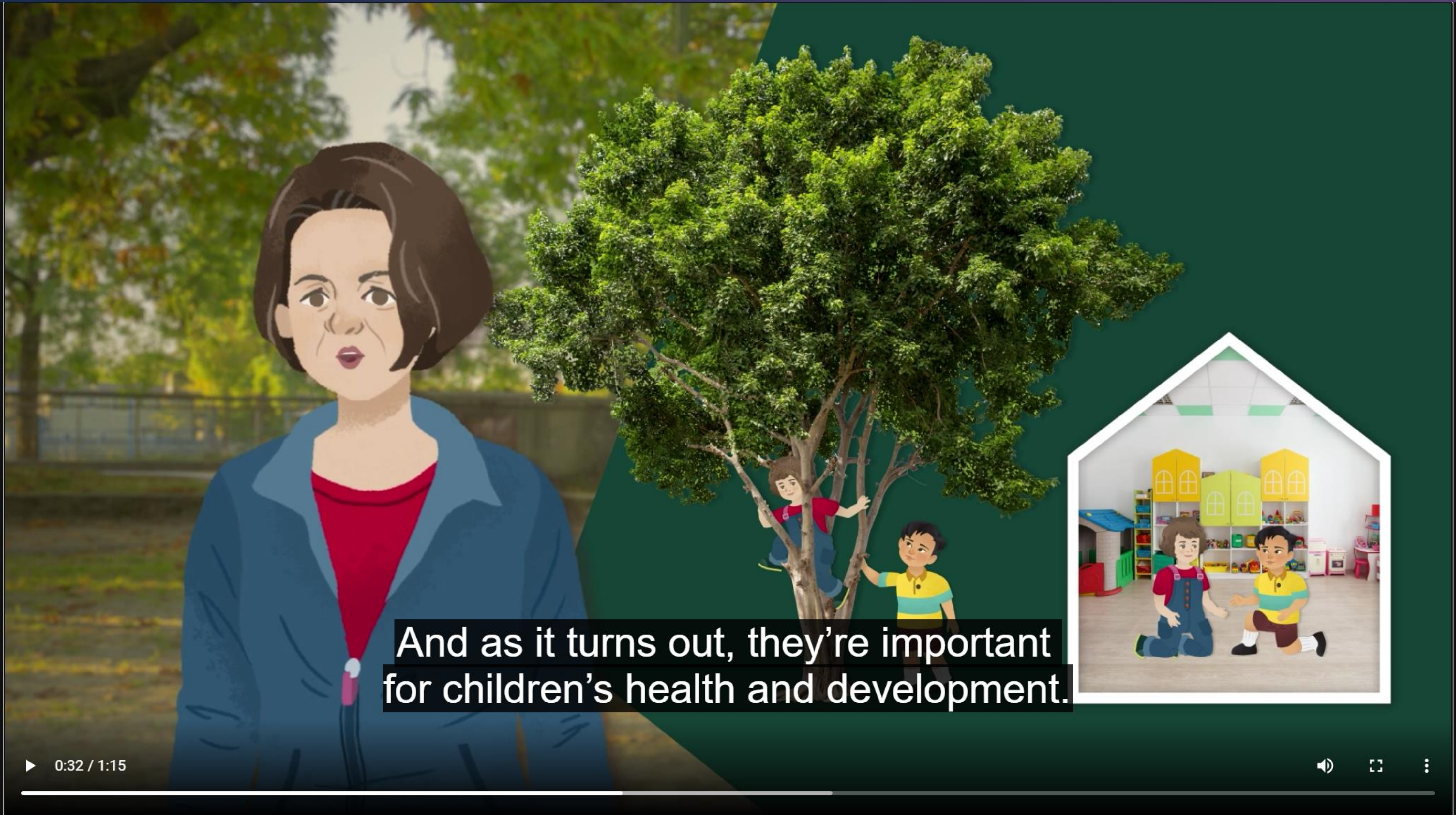
And as it turns out, they're important
for children's health and development.
0:32 / 1:15

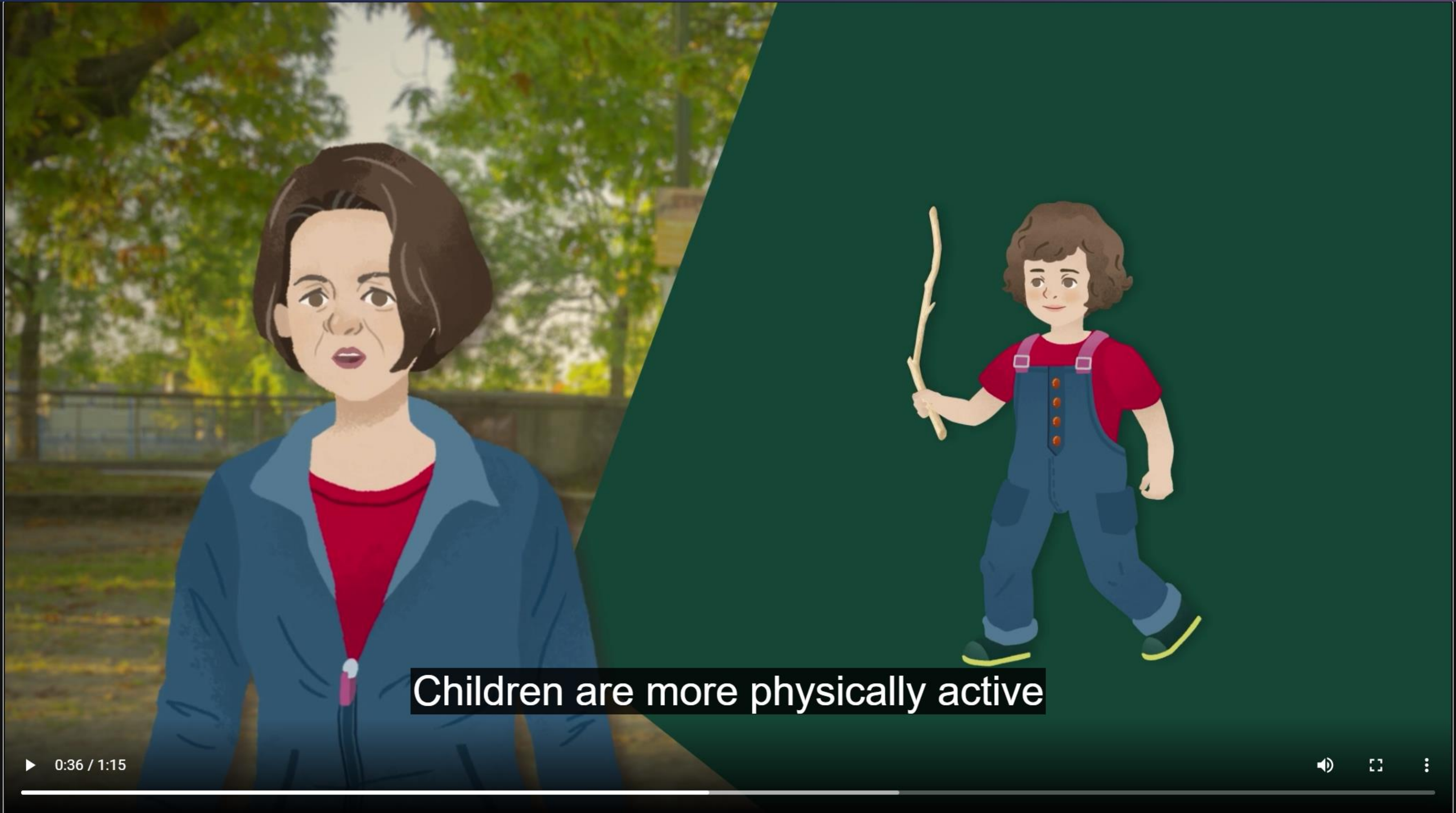
Children are more physically active
0:36 / 1:15

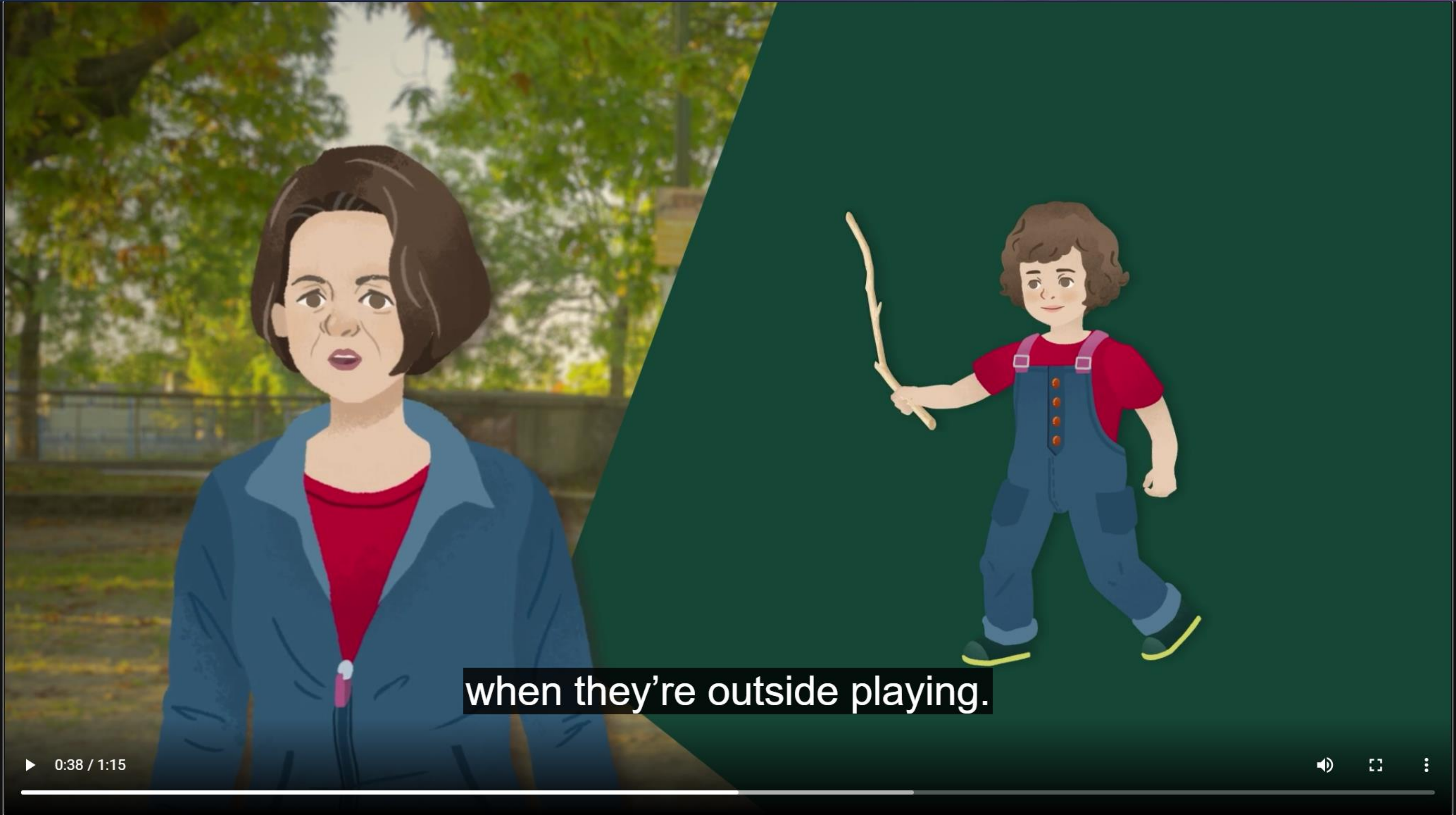
when they're outside playing.
0:38 / 1:15

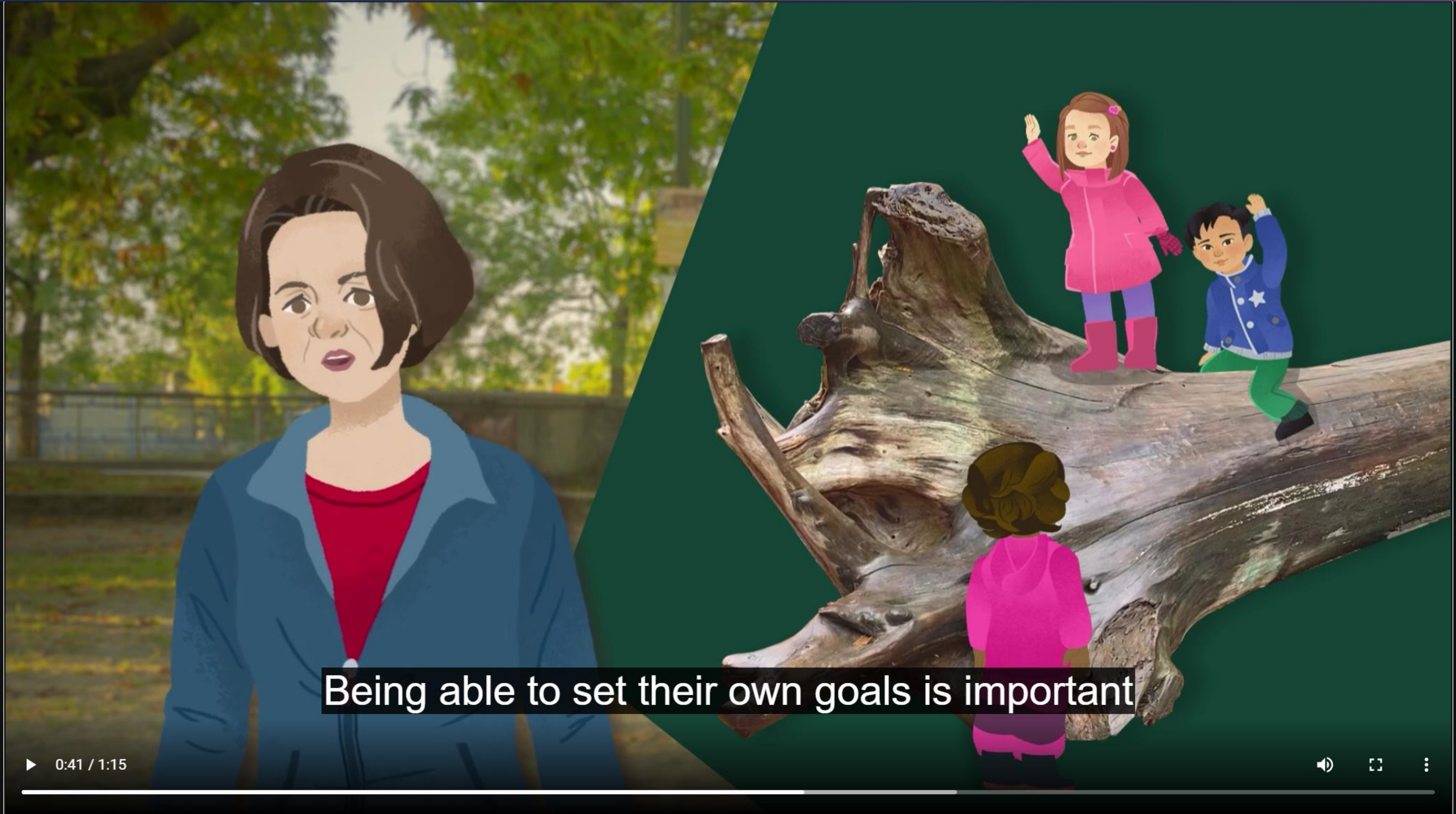
Being able to set their own goals is important
0:41 / 1:15

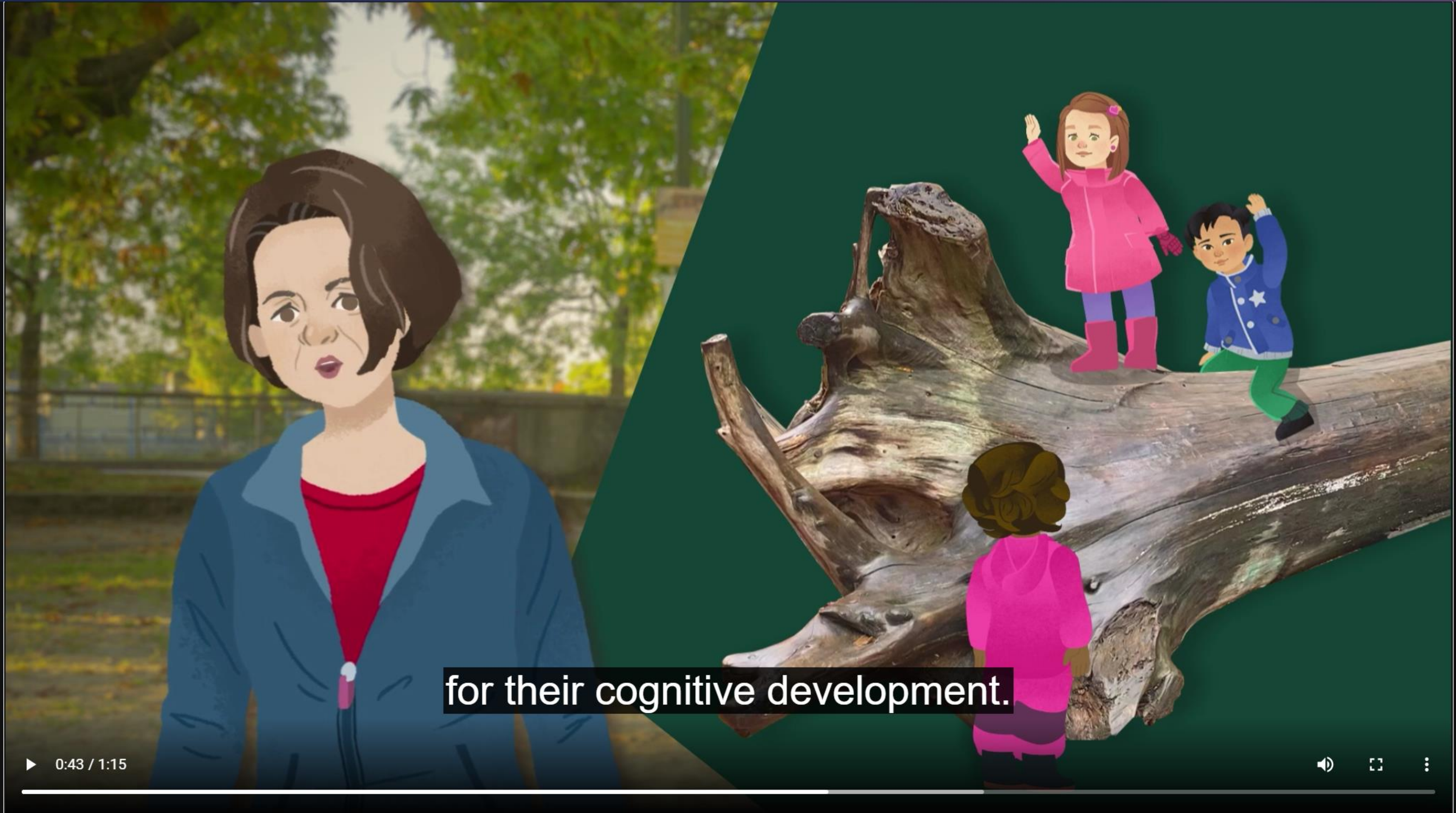
for their cognitive development.
0:43 / 1:15

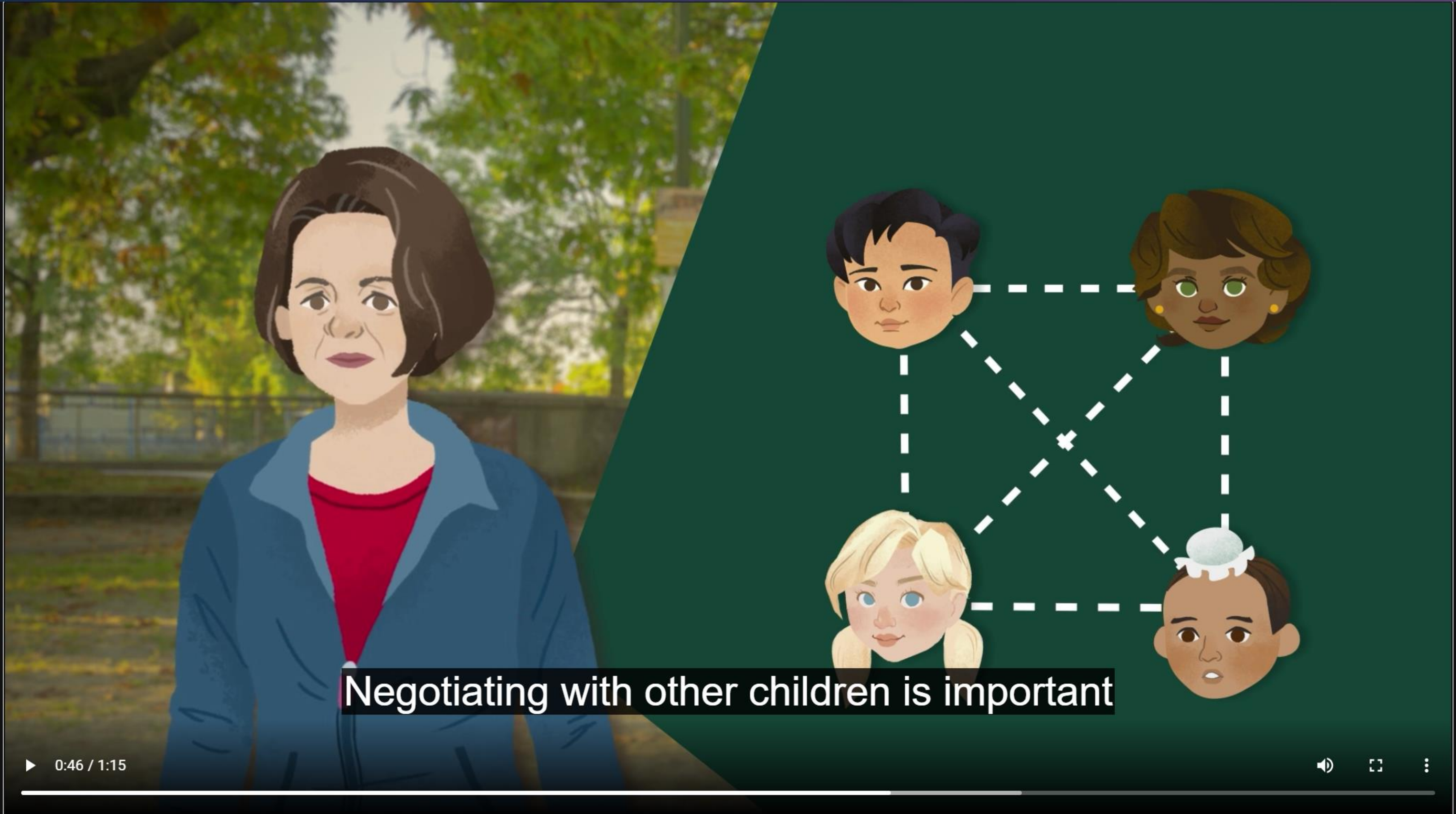
Negotiating with other children is important
0:46 / 1:15

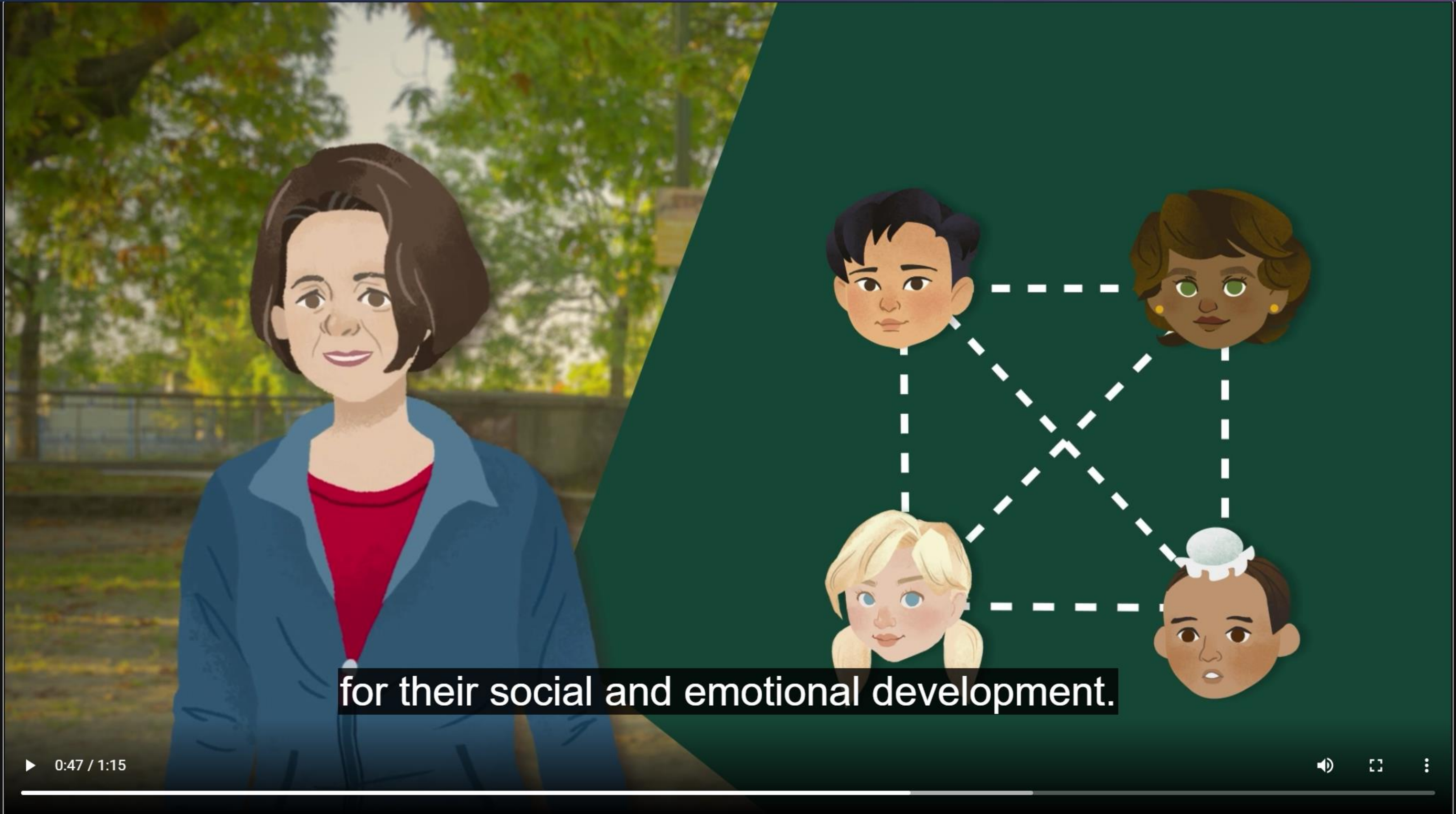
for their social and emotional development.
0:47 / 1:15

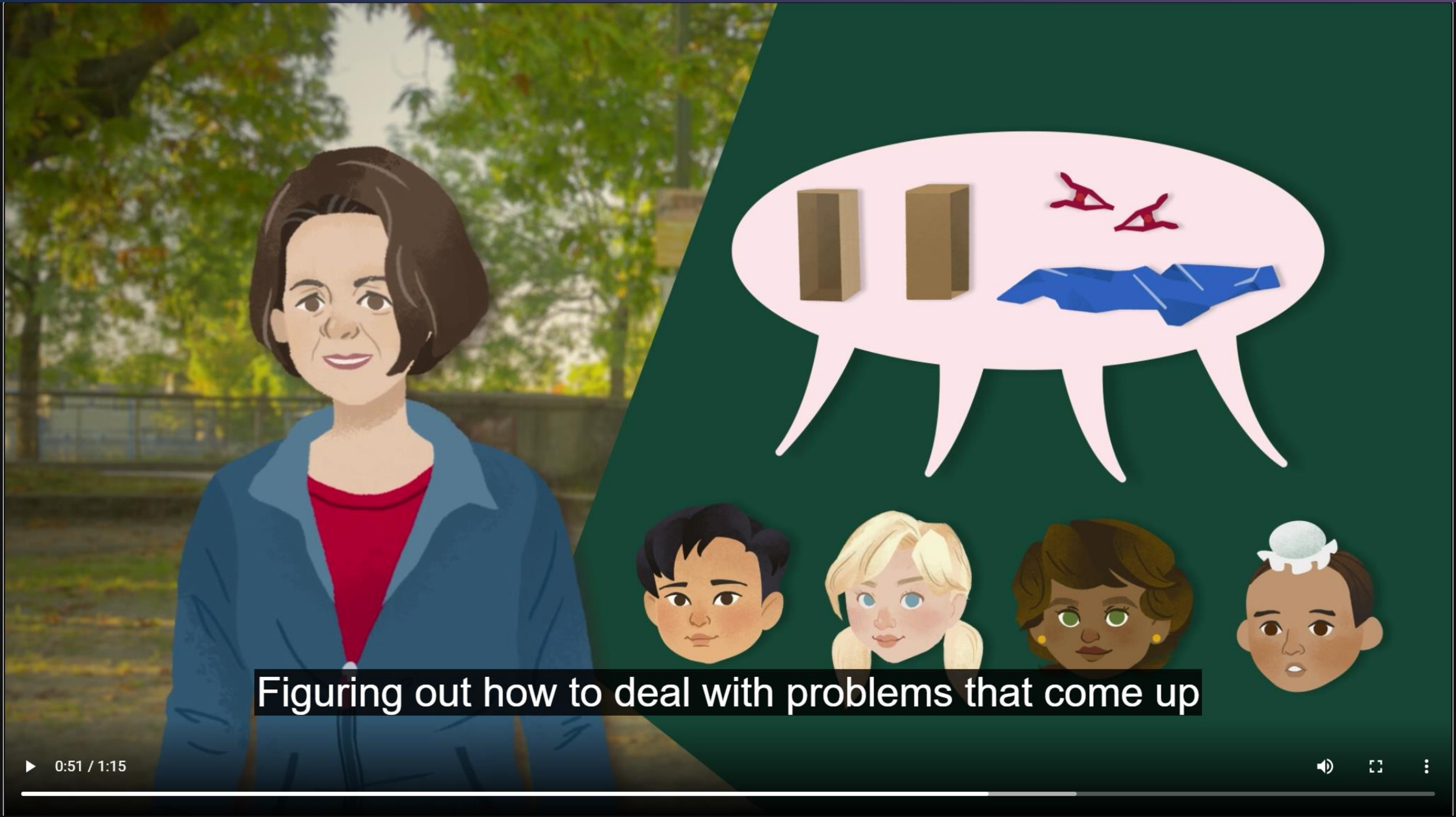
Figuring out how to deal with problems that come up
0:51 / 1:15

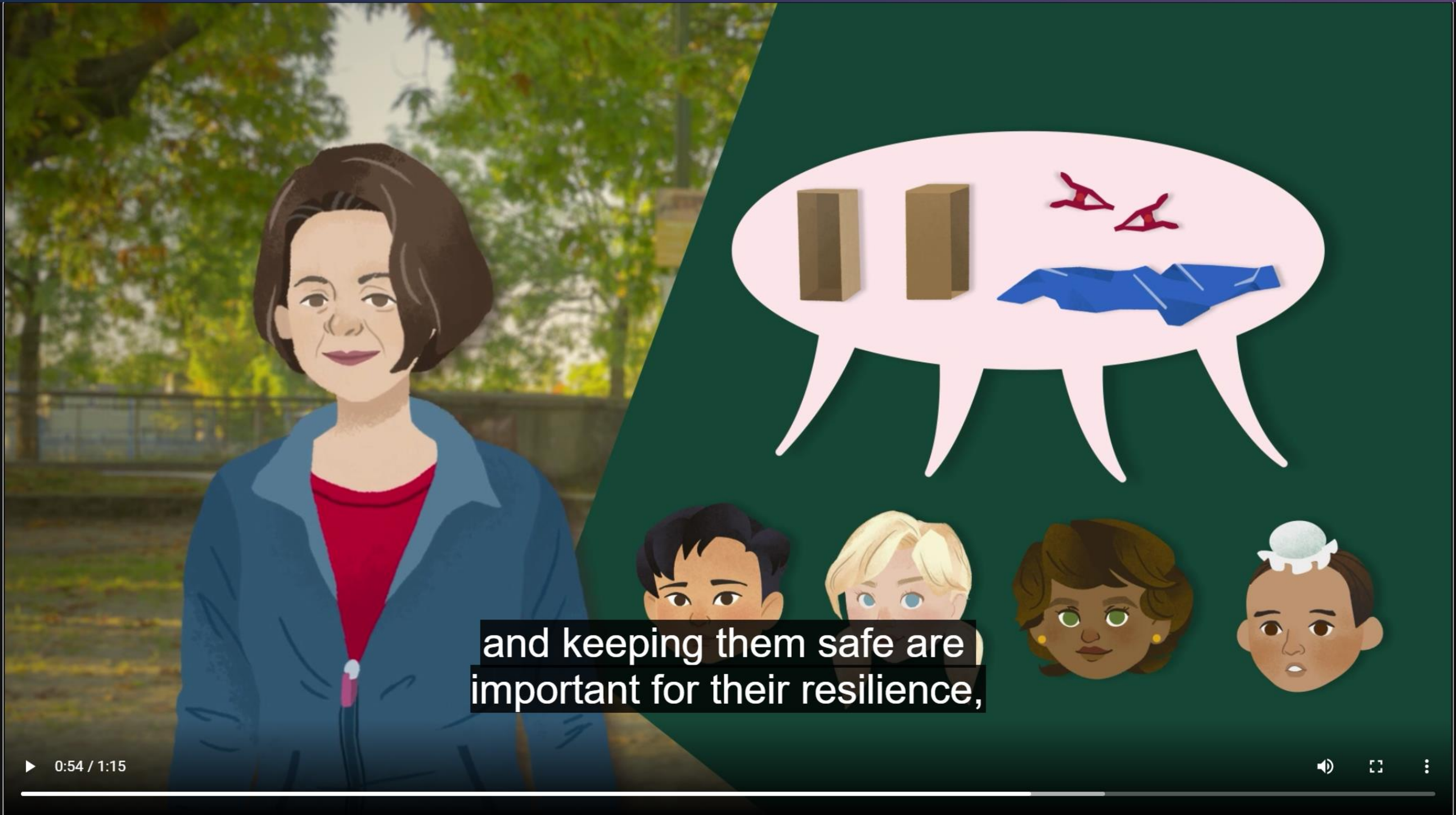
and keeping them safe are
important for their resilience,
0:54 / 1:15

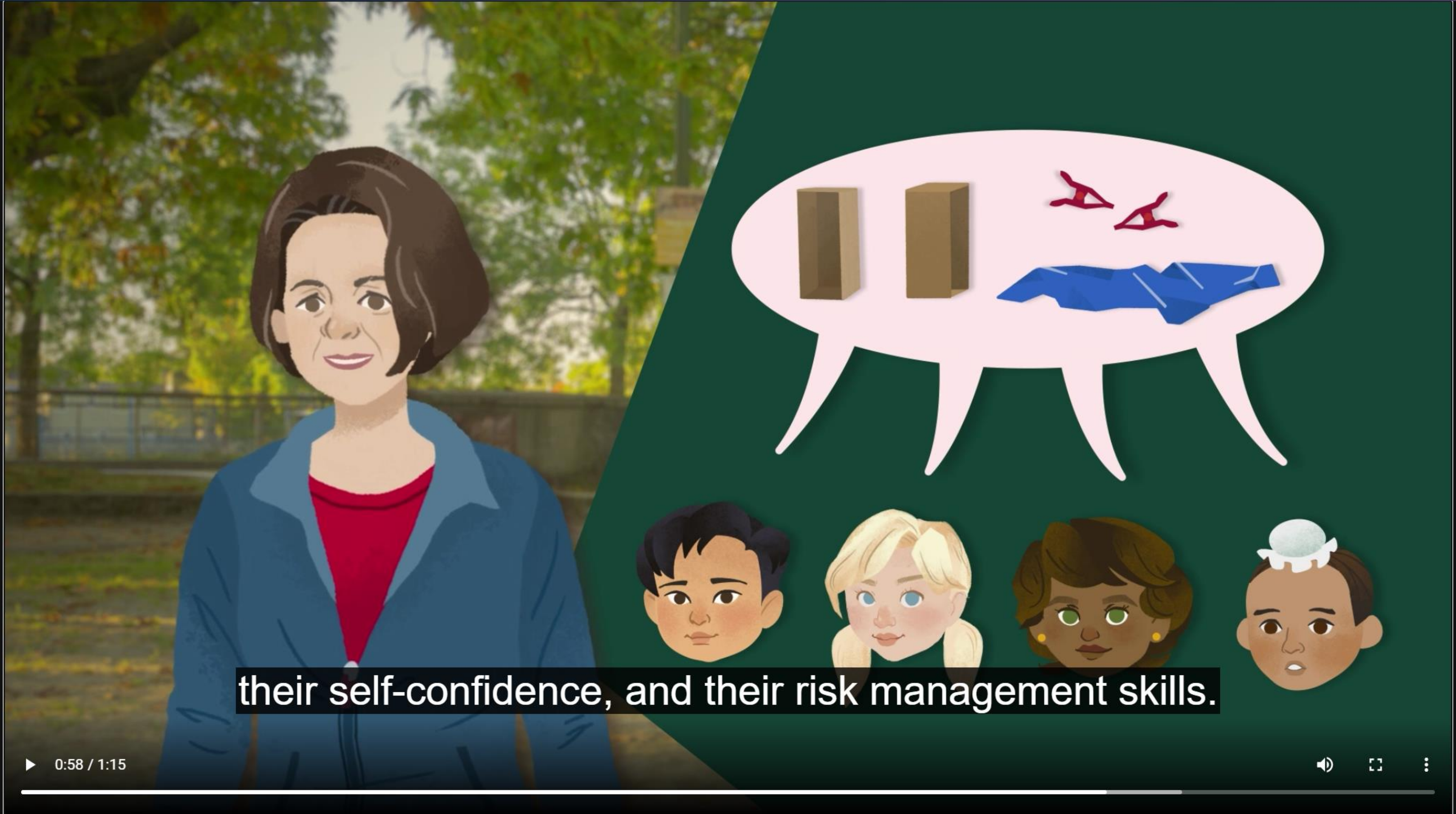
their self-confidence, and their risk management skills.
0:58 / 1:15

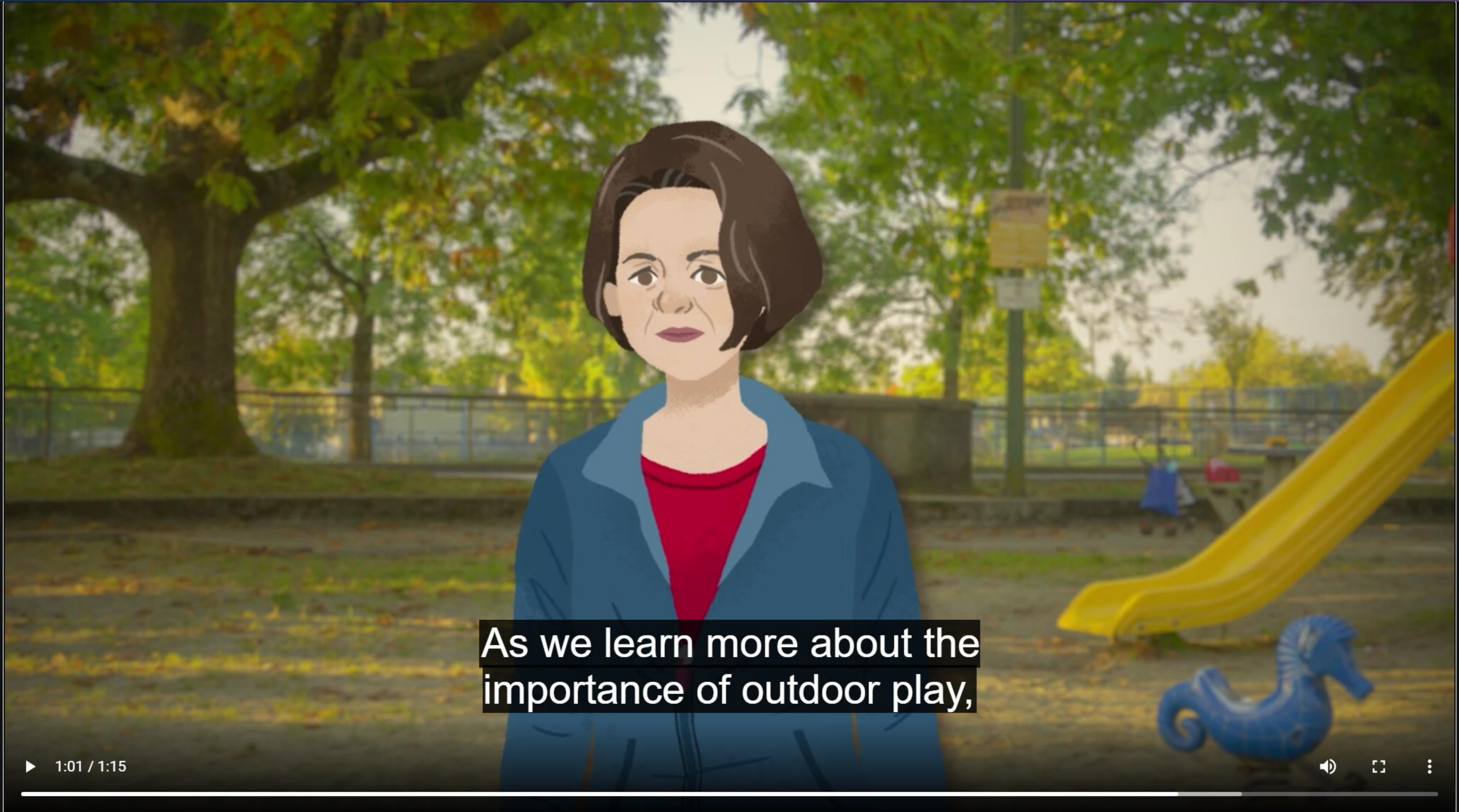
As we learn more about the
importance of outdoor play,
1:01 / 1:15

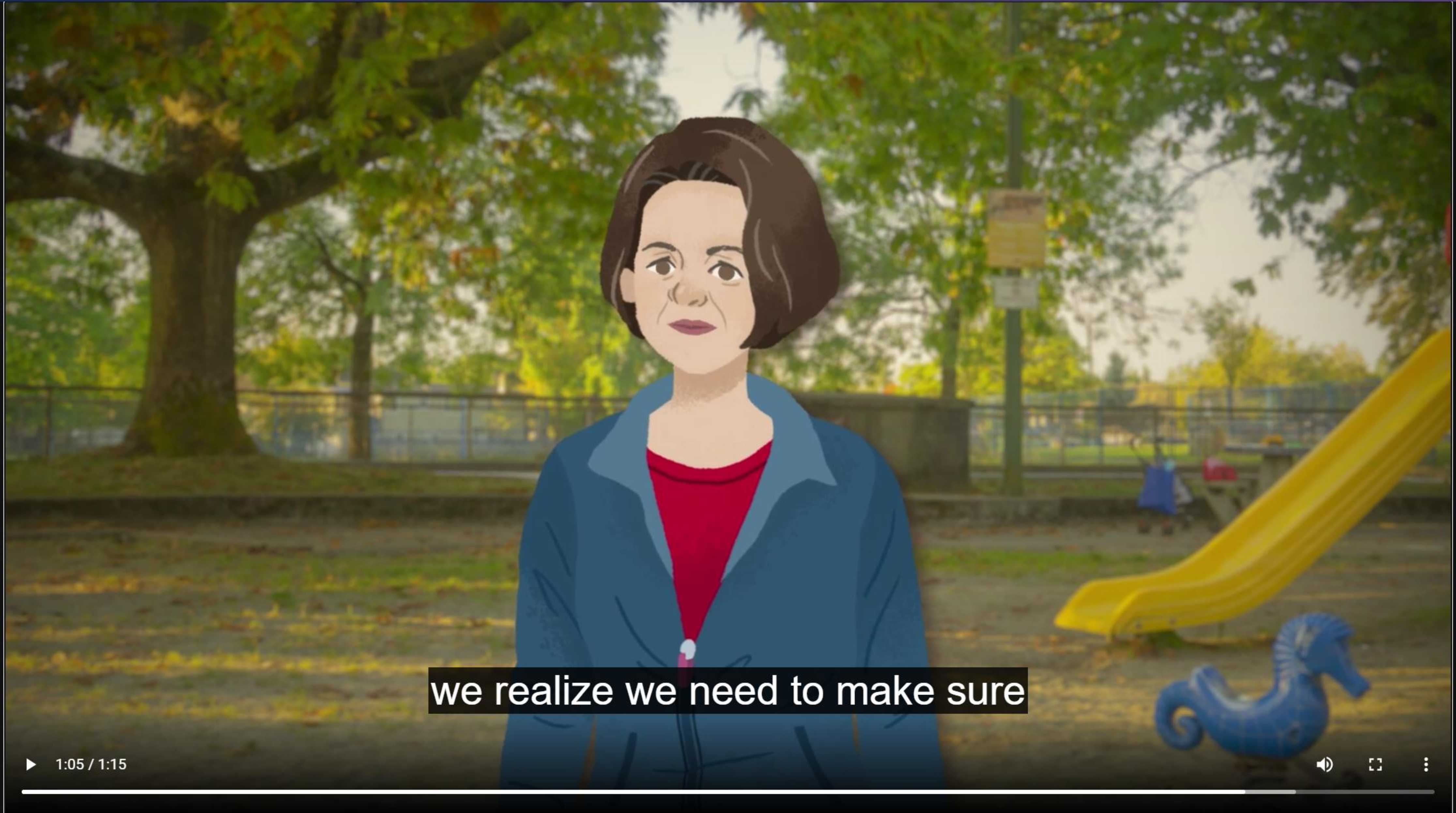
we realize we need to make sure
1:05 / 1:15

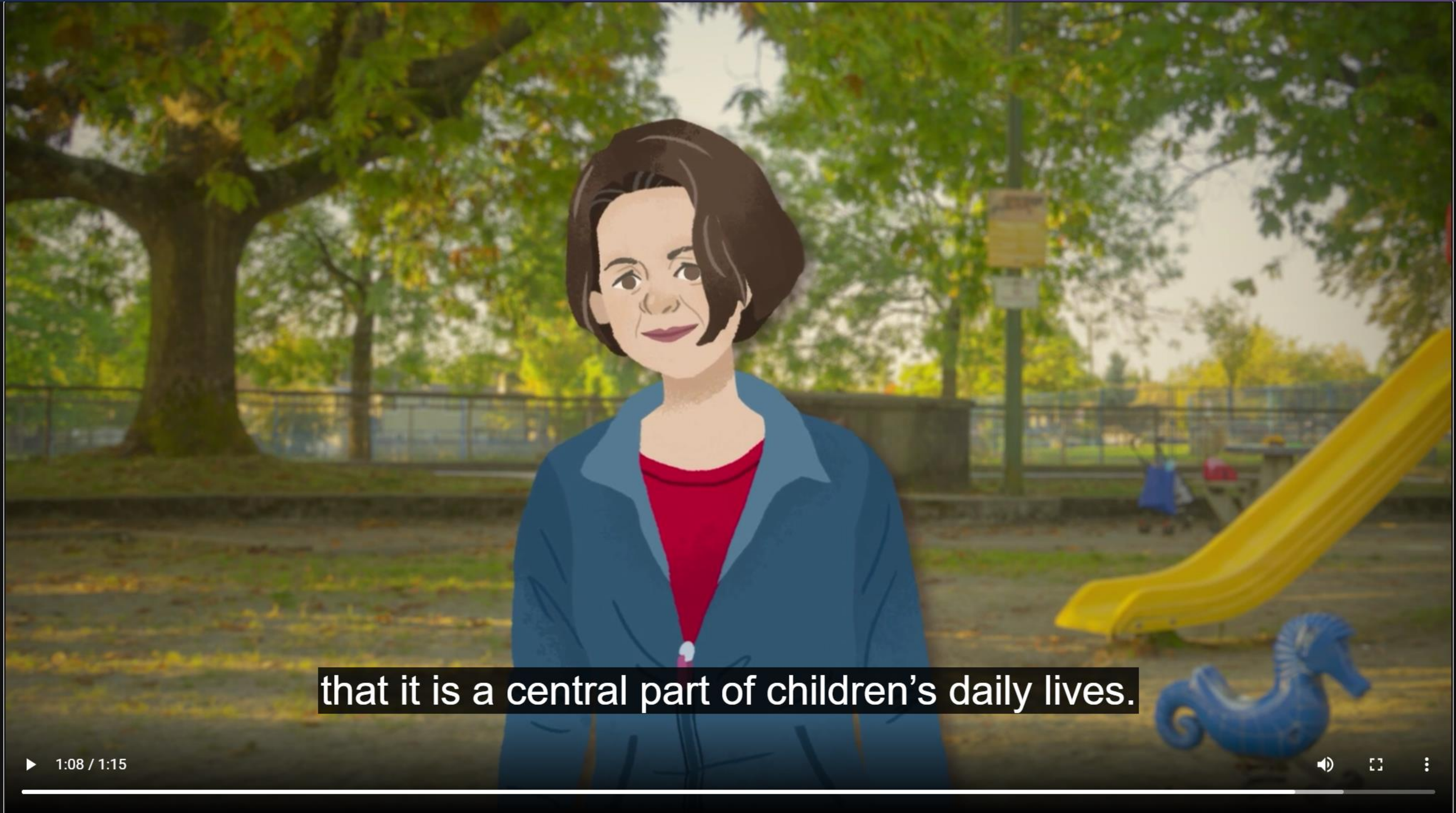
that it is a central part of children’s daily lives.
1:08 / 1:15

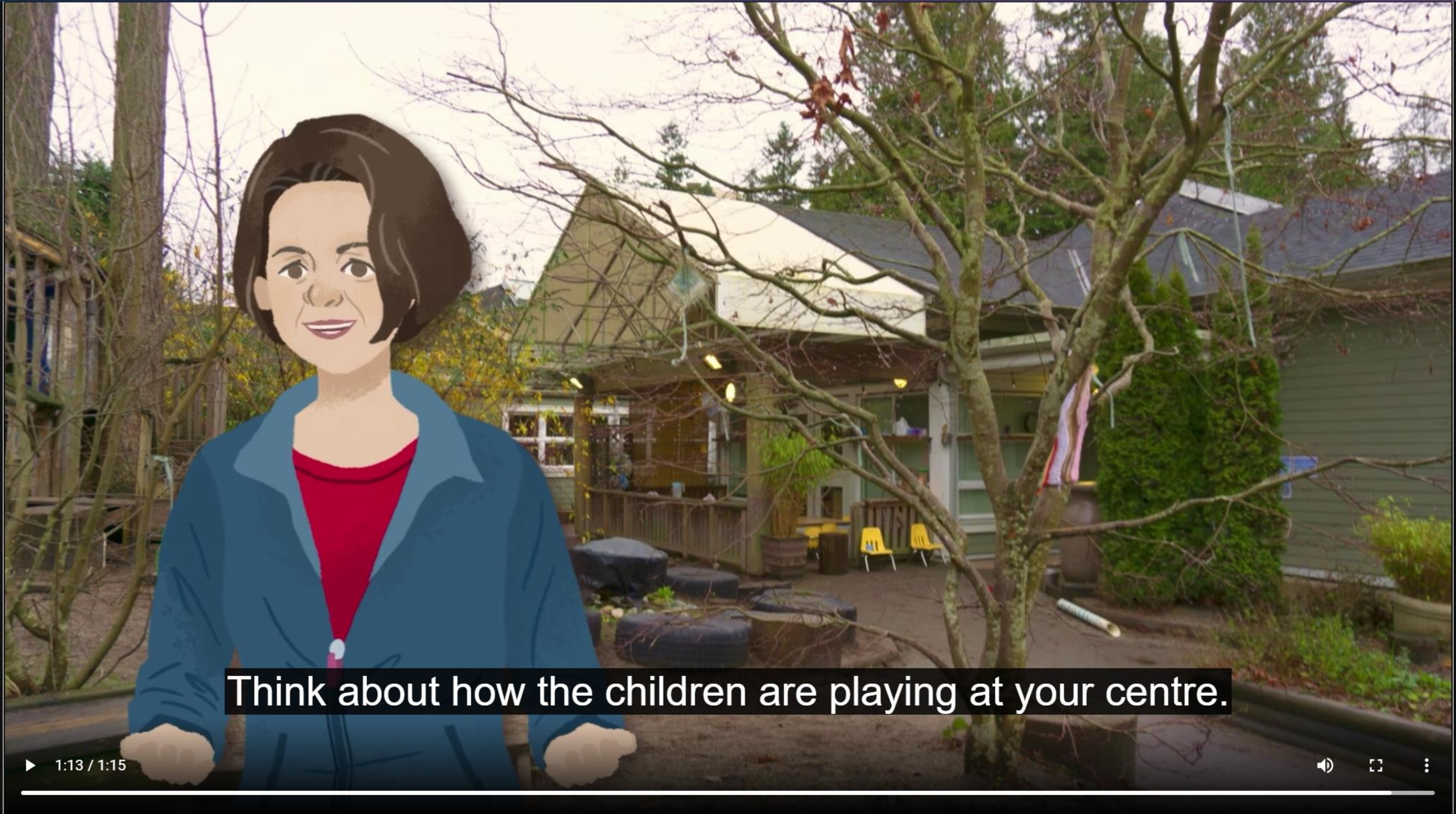
Think about how the children are playing at your centre.
1:13 / 1:15

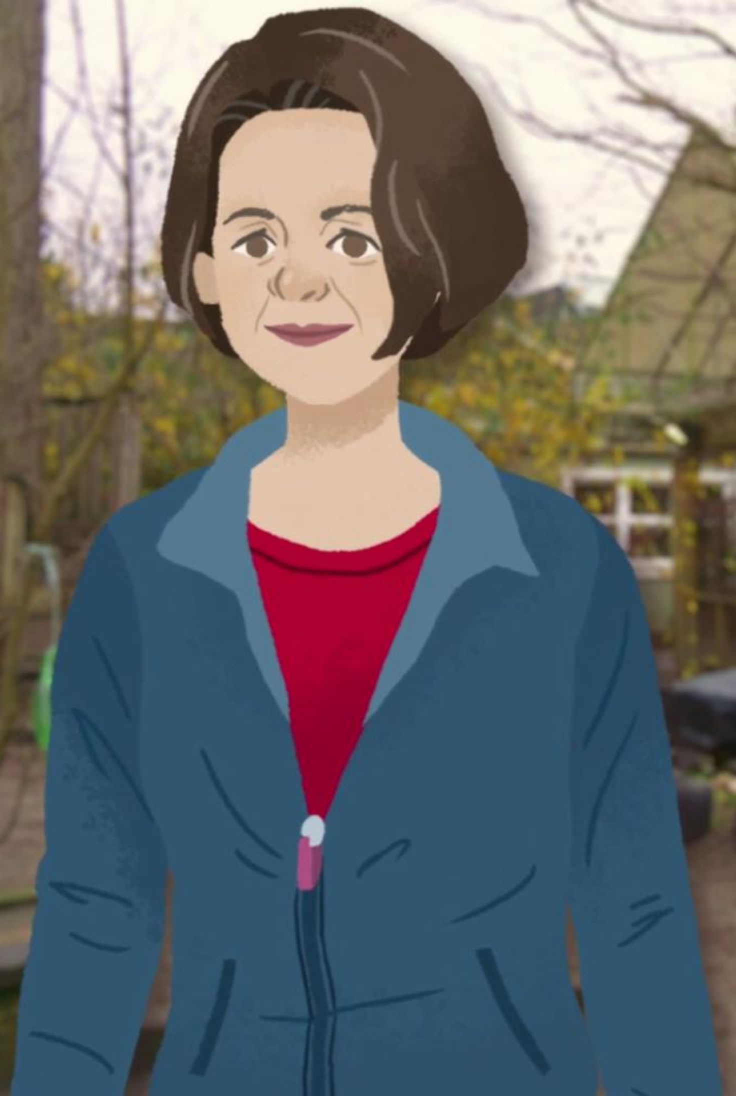

How do children currently play at your centre? (Select all that apply)

- Playing group games, such as tag or hide-and-seek
- Cycling, skateboarding, or scootering
- Building things with loose parts and tools
- Climbing trees or structures
- Running
- Jumping
- Playing on playground equipment
- Playing with natural elements, such as sand, sticks, mud
- Exploring nature
- Imaginative play
- Daydreaming
- Reading
- Playing on screens, such as playing video games
- Other

Next

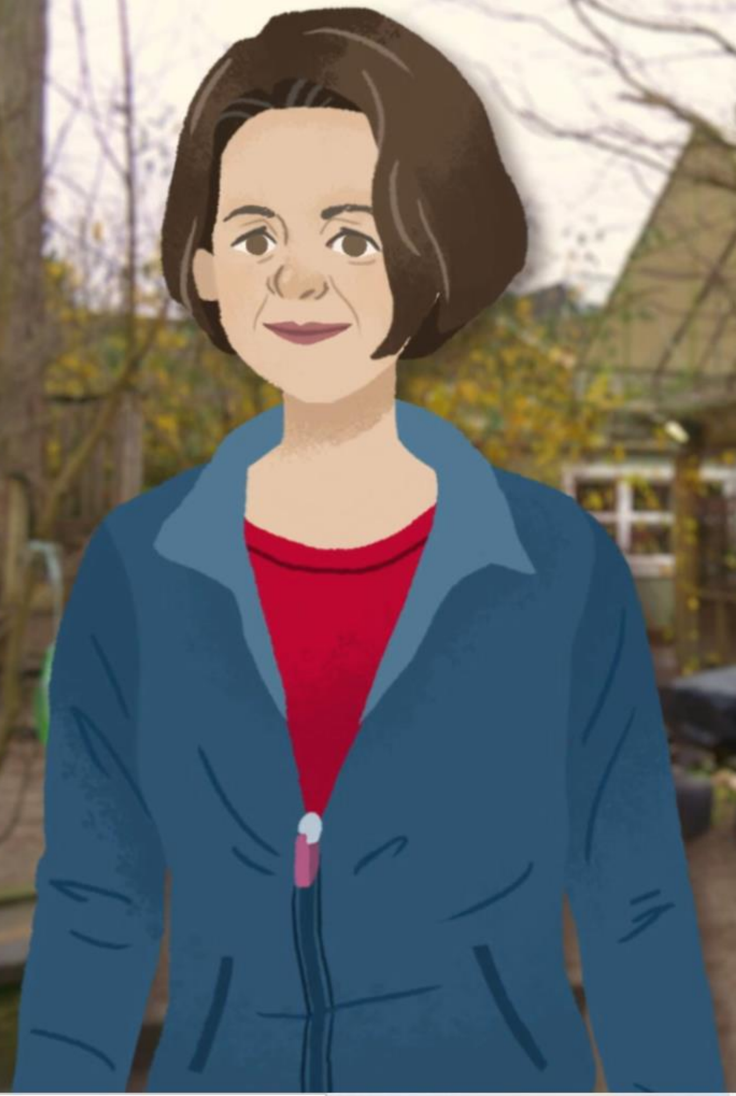

How would you like children to be able to play at your centre? (Select all that apply)

- Playing group games, such as tag or hide-and-seek
- Cycling, skateboarding, or scootering
- Building things with loose parts and tools
- Climbing trees or structures
- Running
- Jumping
- Playing on playground equipment
- Playing with natural elements, such as sand, sticks, mud
- Exploring nature
- Imaginative play
- Daydreaming
- Reading
- Playing on screens, such as playing video games
- Other

Next

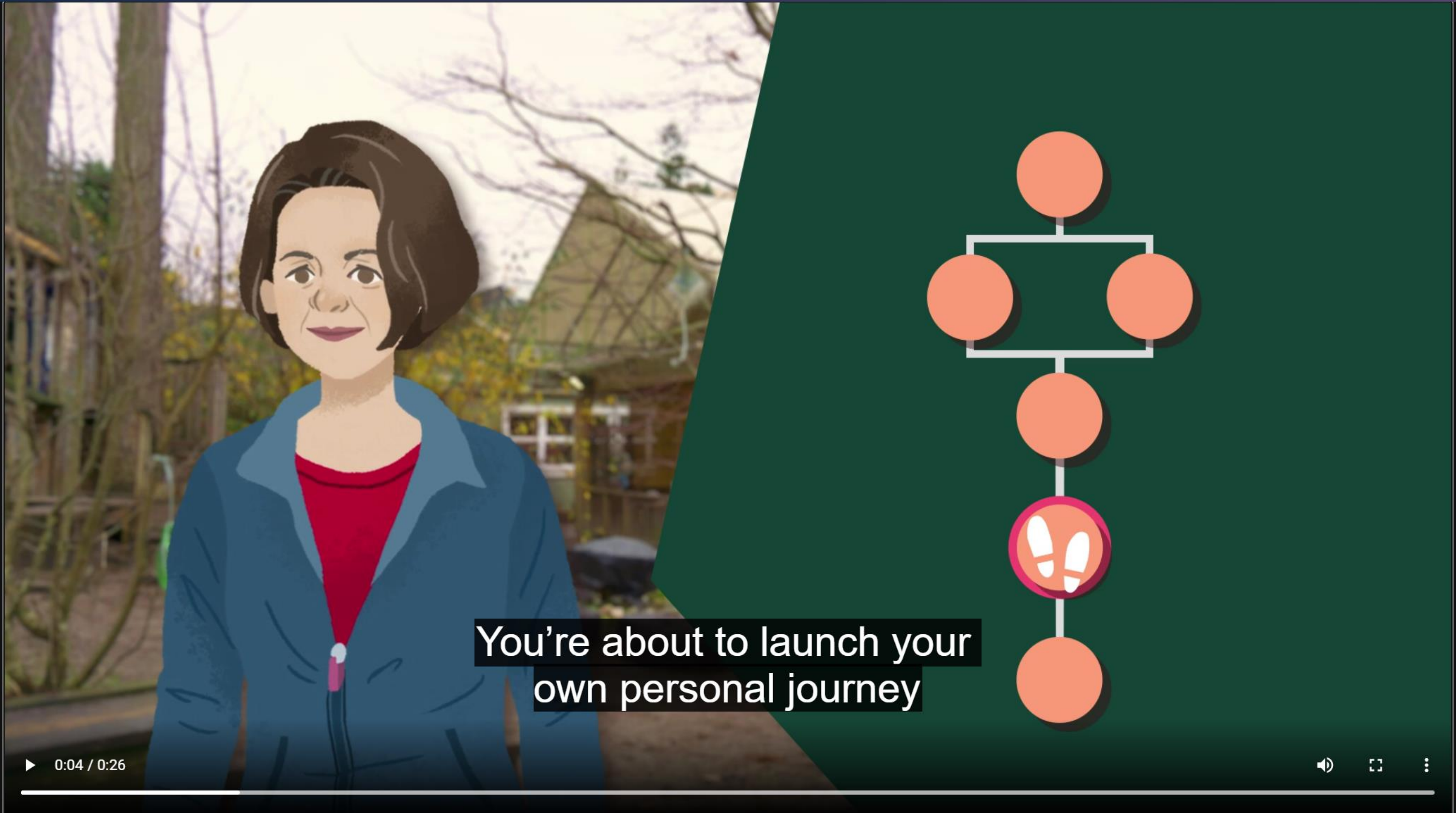
You're about to launch your own personal journey
0:04 / 0:26

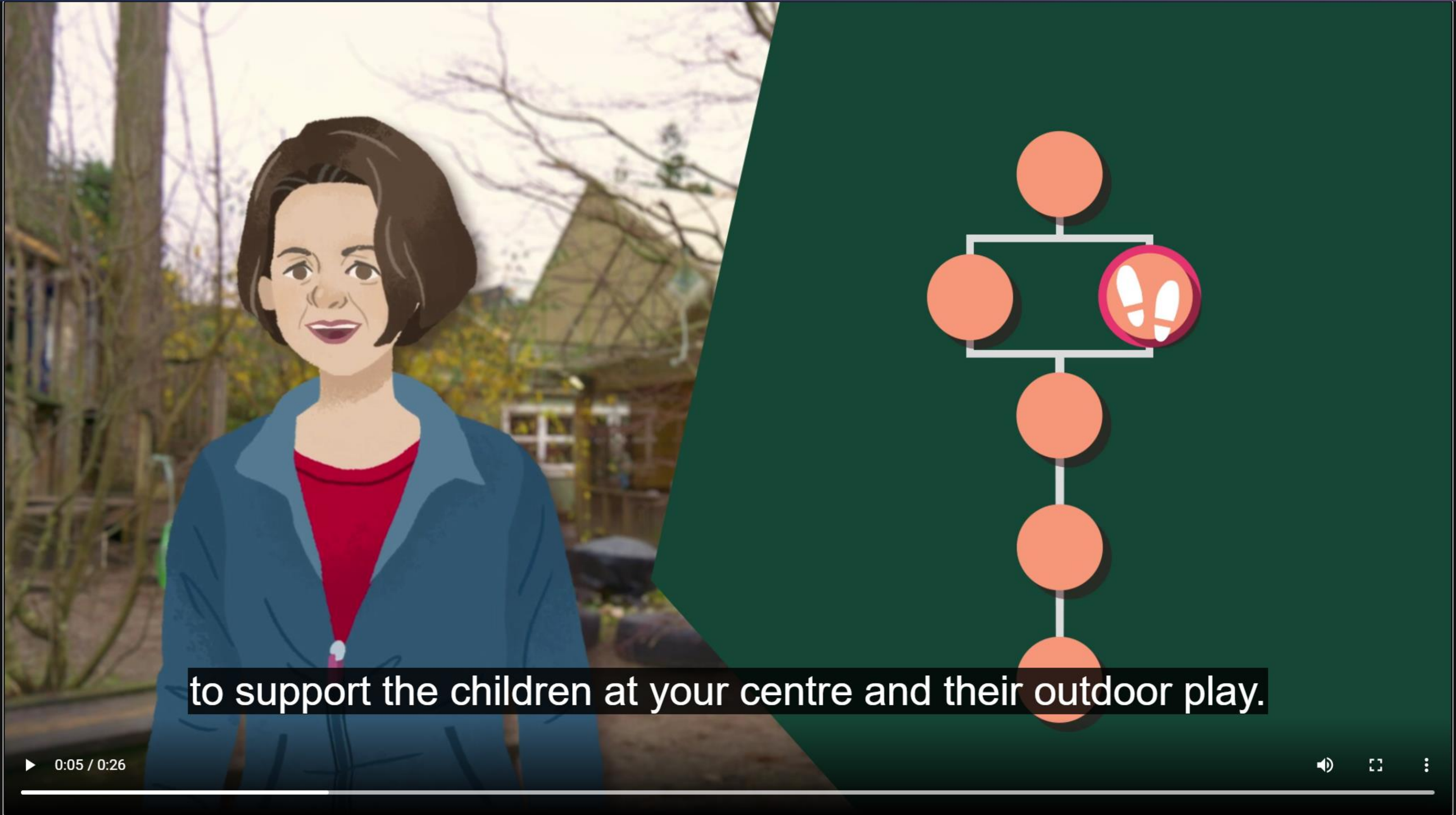
to support the children at your centre and their outdoor play.
0:05 / 0:26

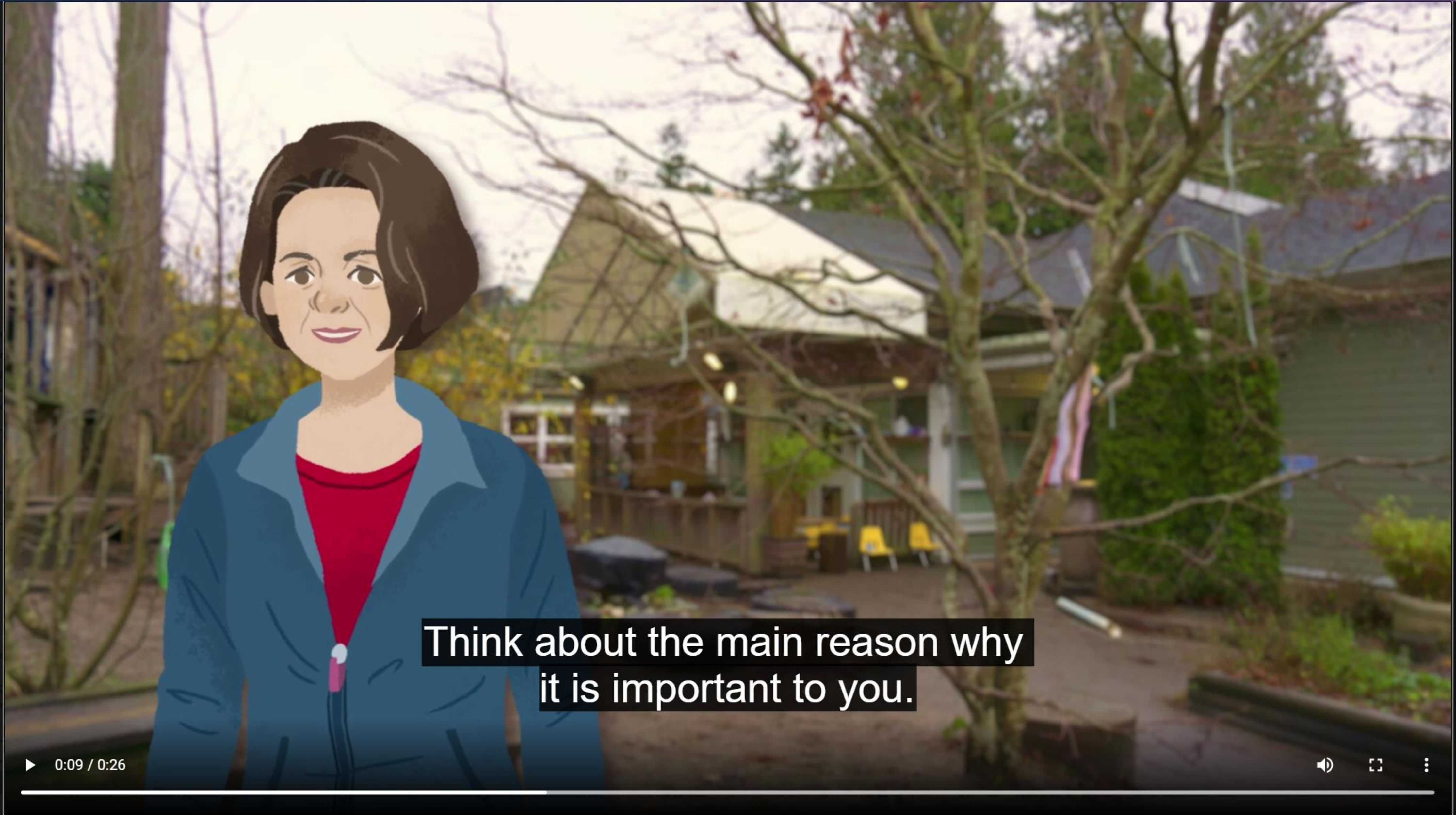
Think about the main reason why
it is important to you.
0:09 / 0:26

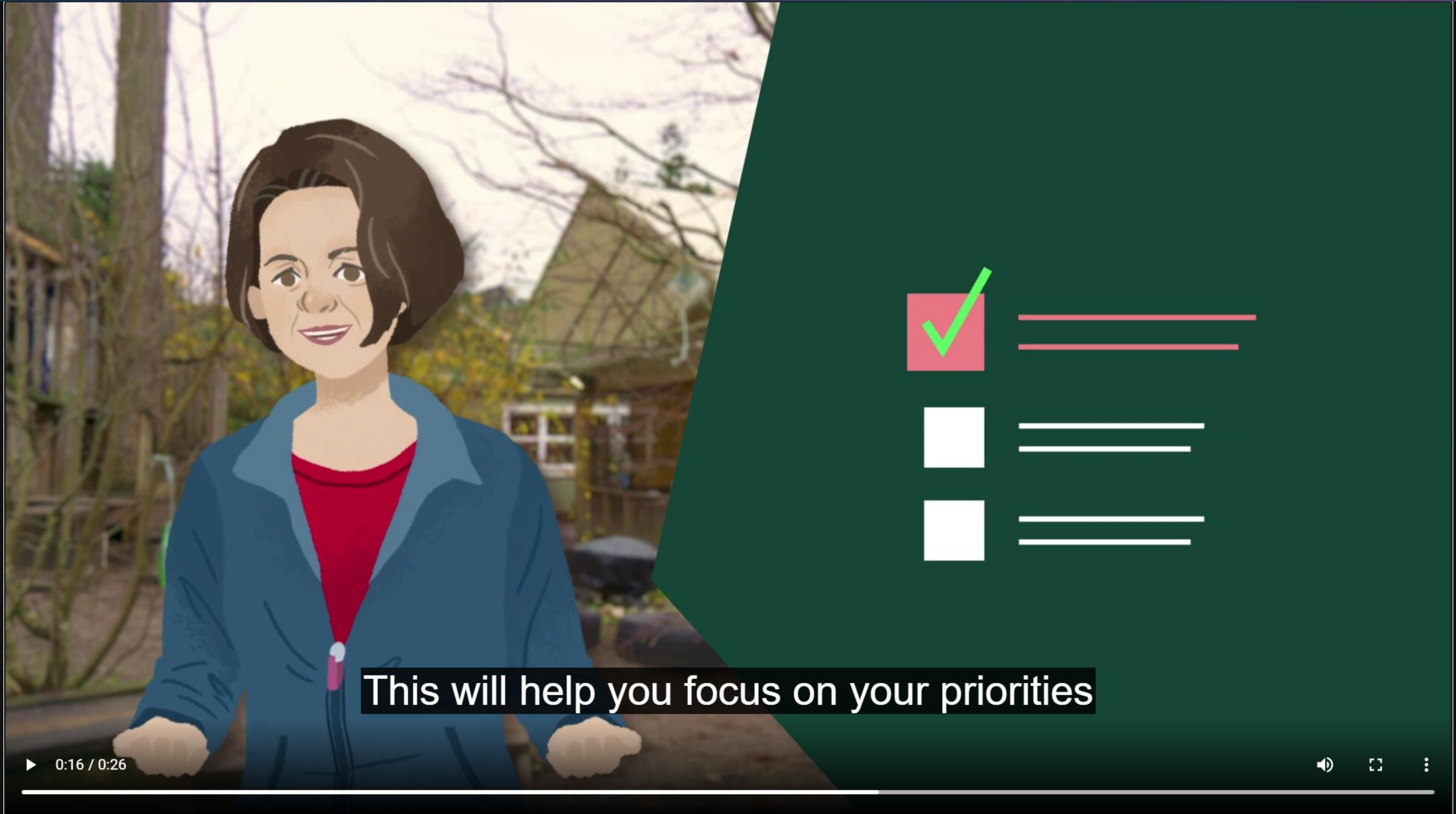
This will help you focus on your priorities
0:16 / 0:26

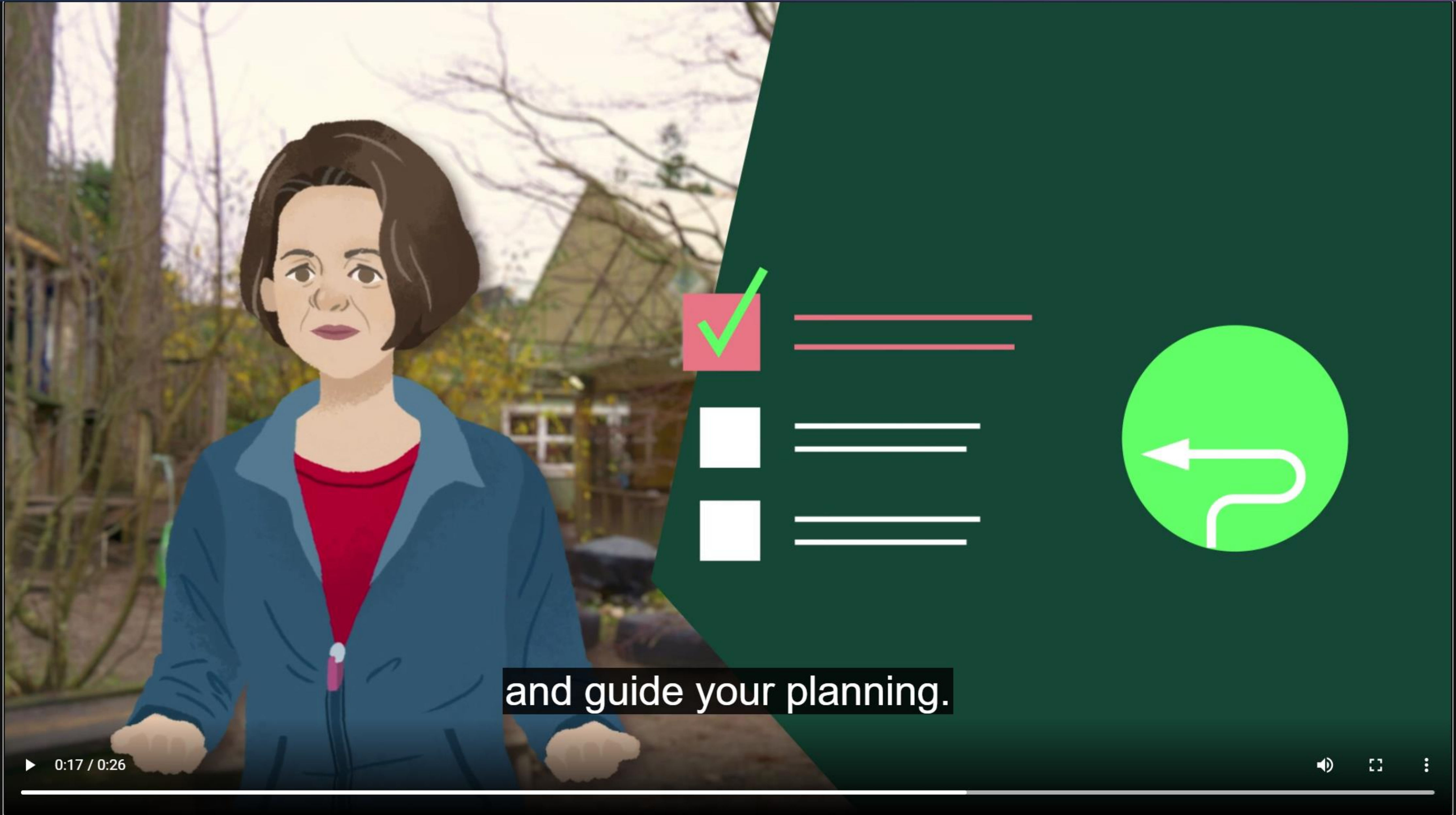
and guide your planning.
0:17 / 0:26

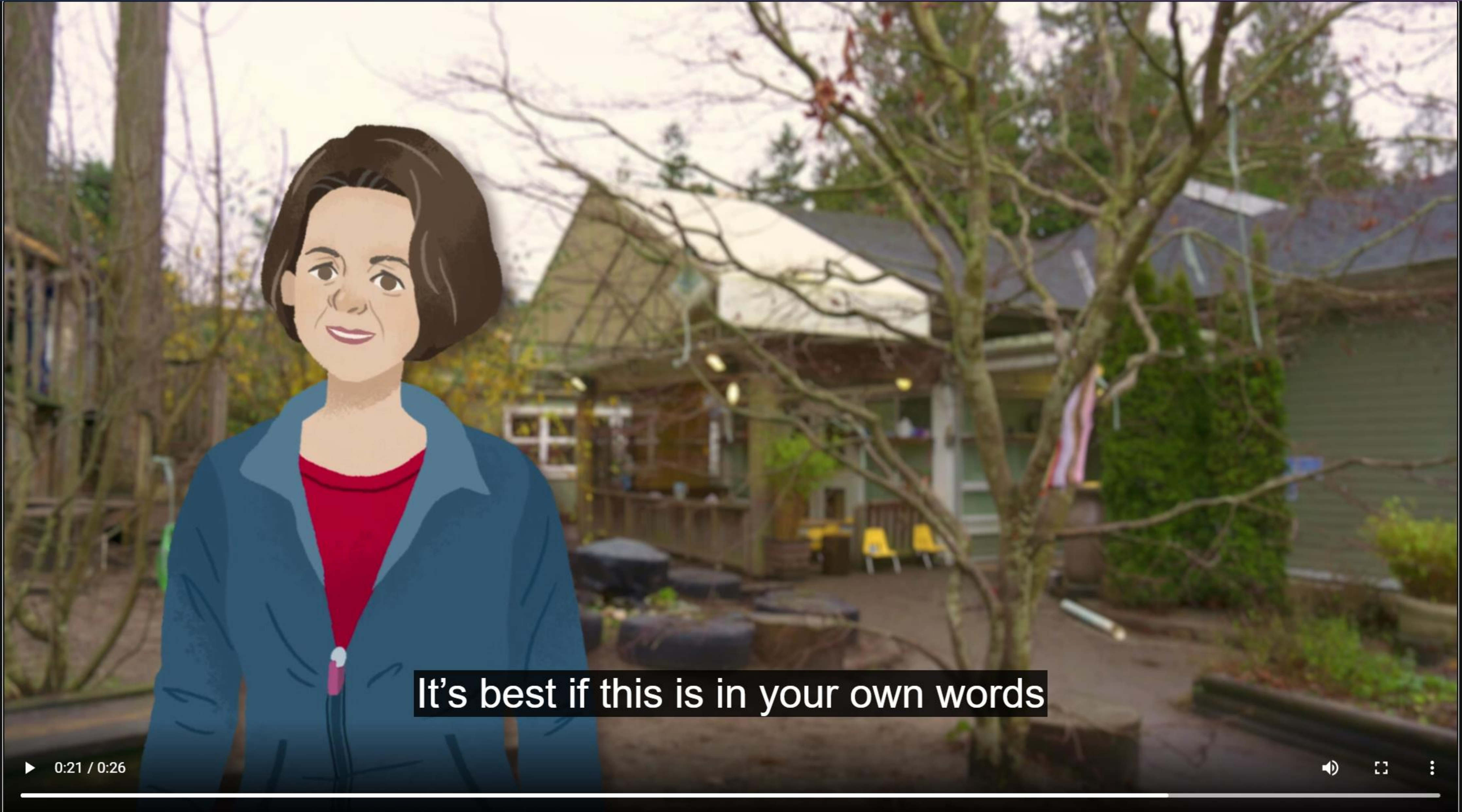
It’s best if this is in your own words
0:21 / 0:26

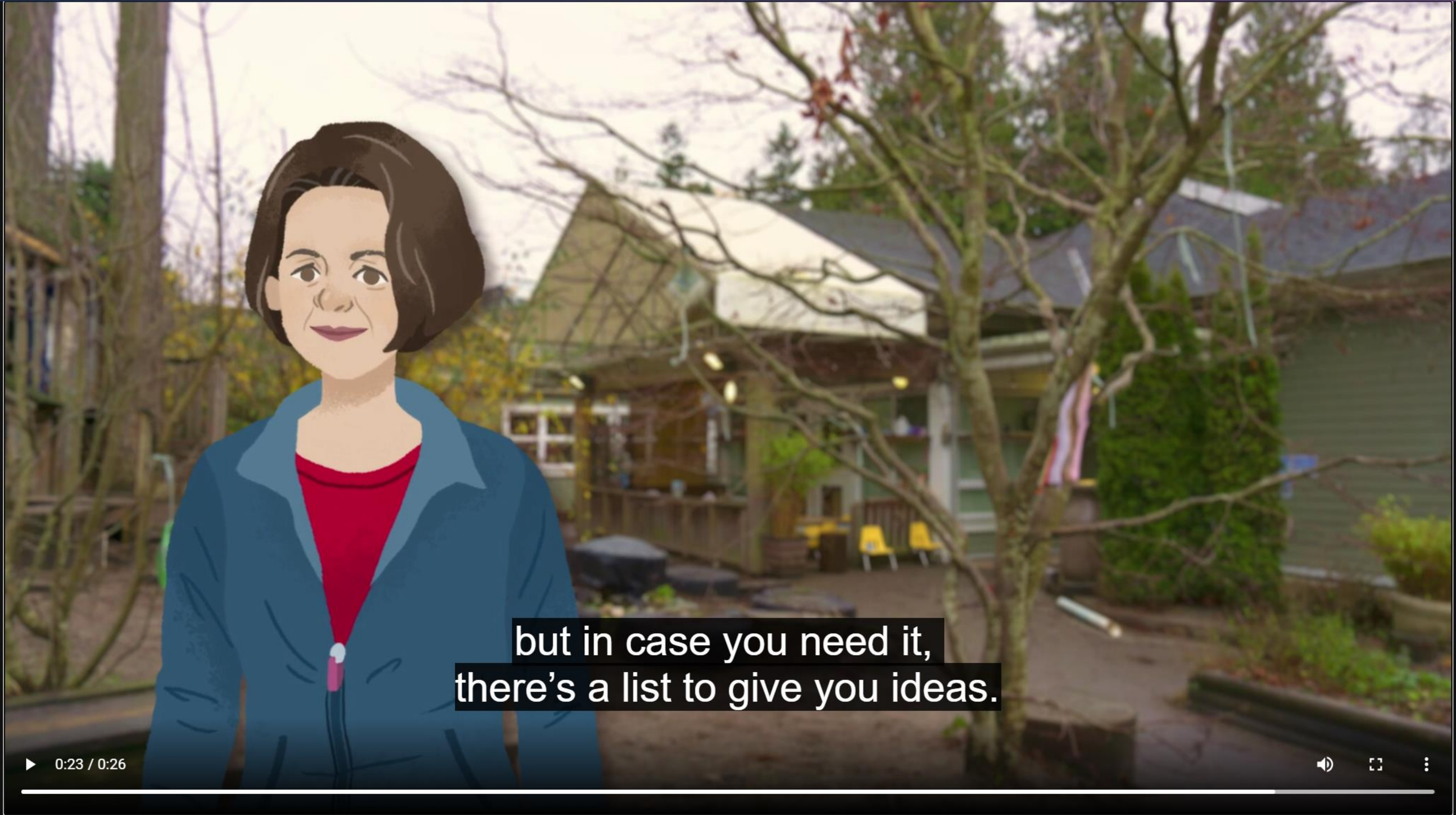
but in case you need it,
there's a list to give you ideas.
0:23 / 0:26

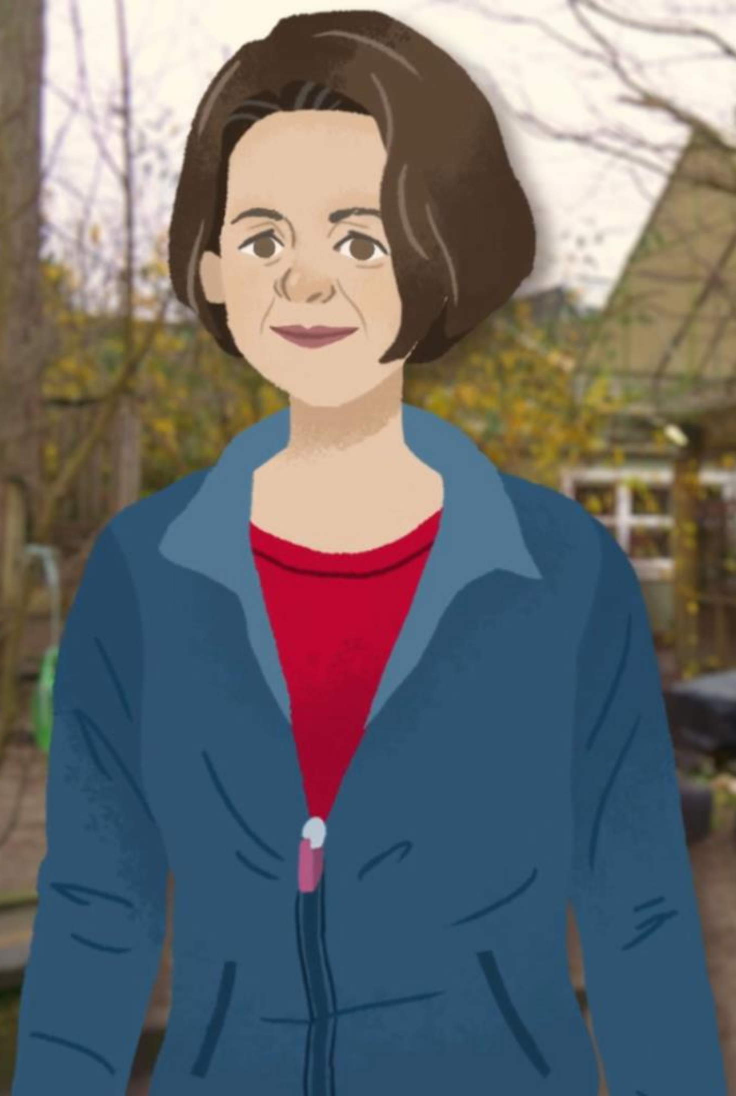

What is the one main reason why you want to support children's outdoor play opportunities?

My reason is...

Here are some examples if you get stuck

- To encourage joy and fun
- To build my relationship with children
- To help children build resilience and independence
- To promote physical activity and movement
- To expand my pedagogical practice
- To reduce exposure to communicable diseases

Next

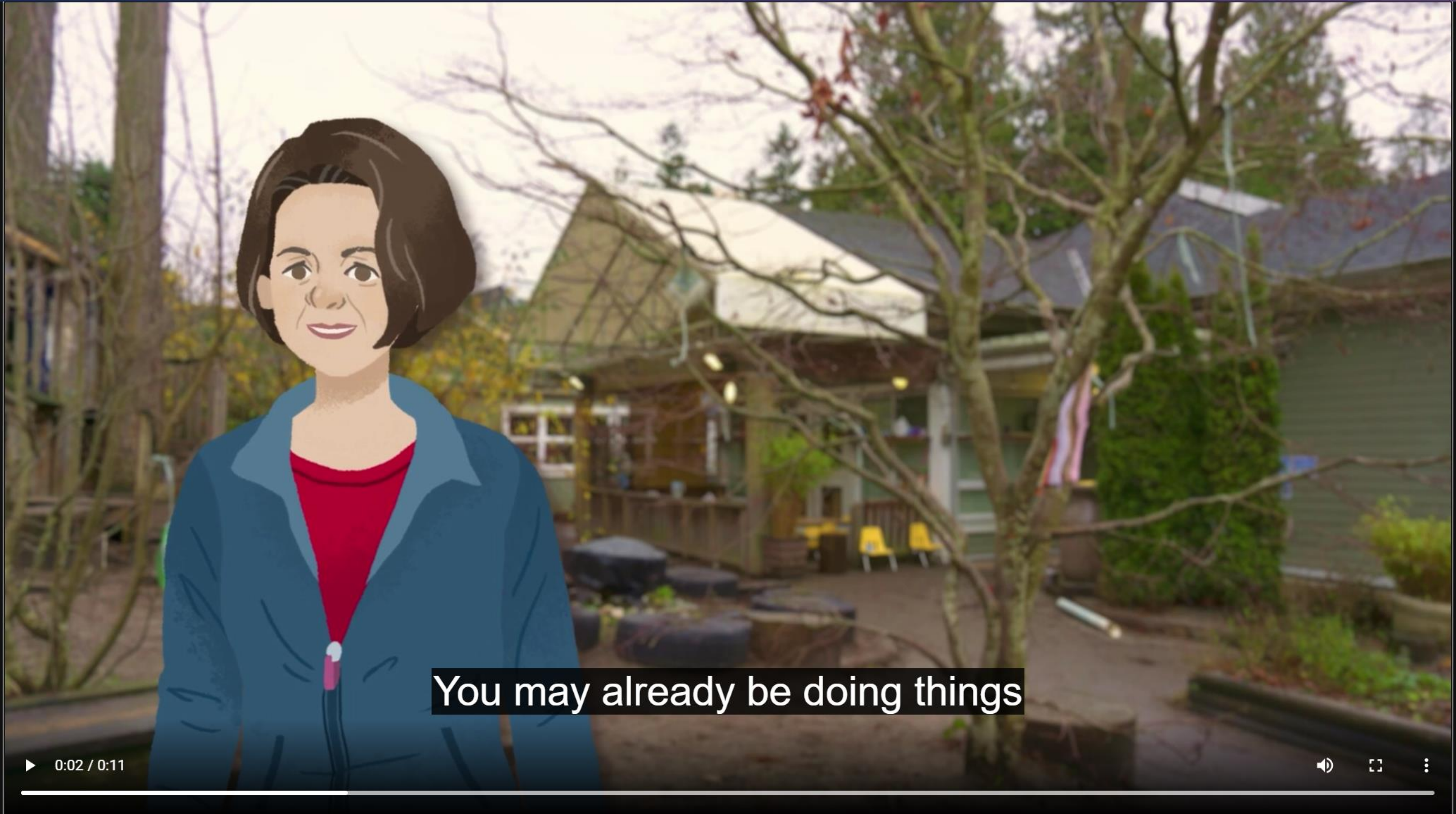
You may already be doing things
0:02 / 0:11

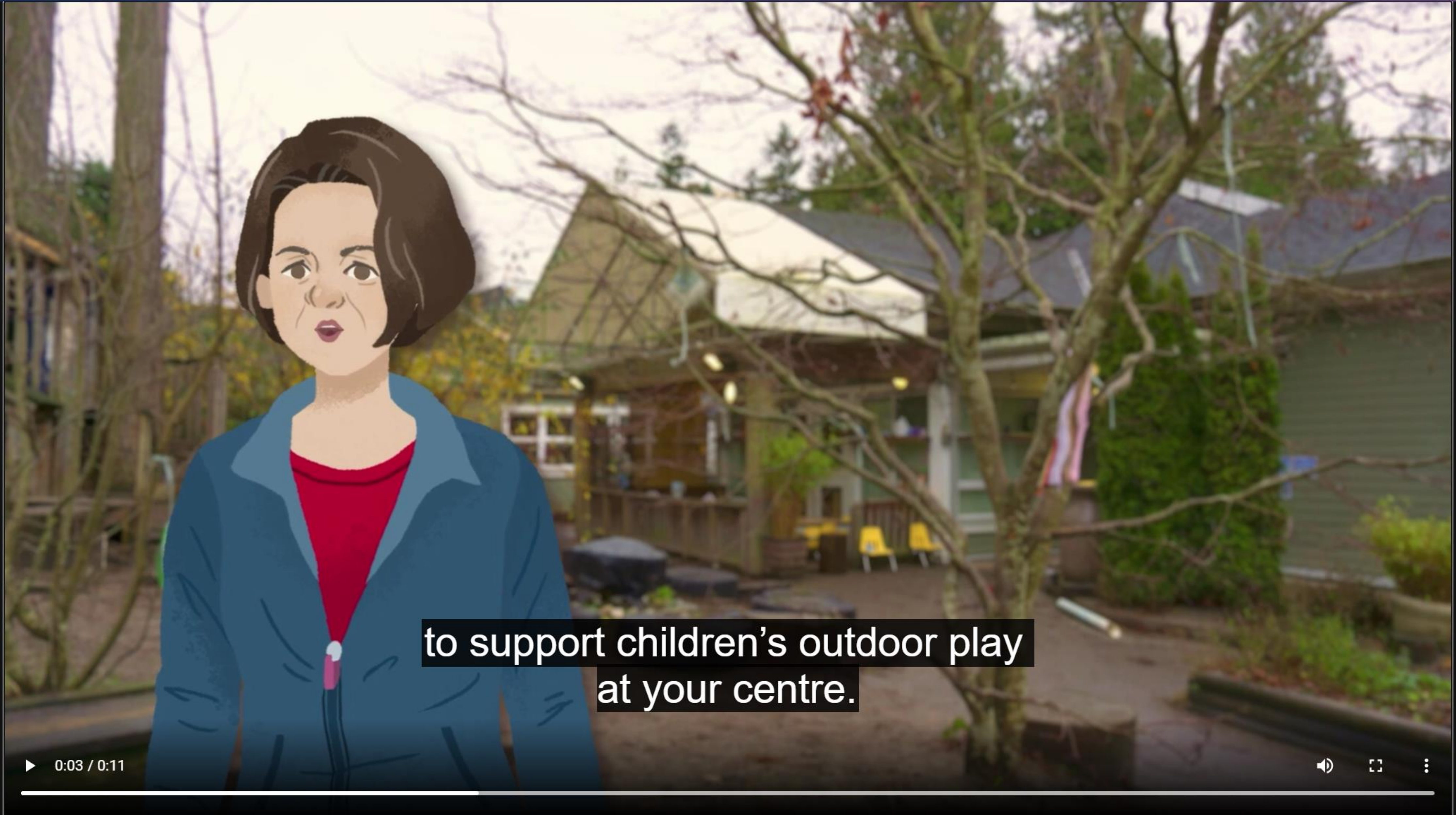
to support children’s outdoor play
at your centre.
0:03 / 0:11

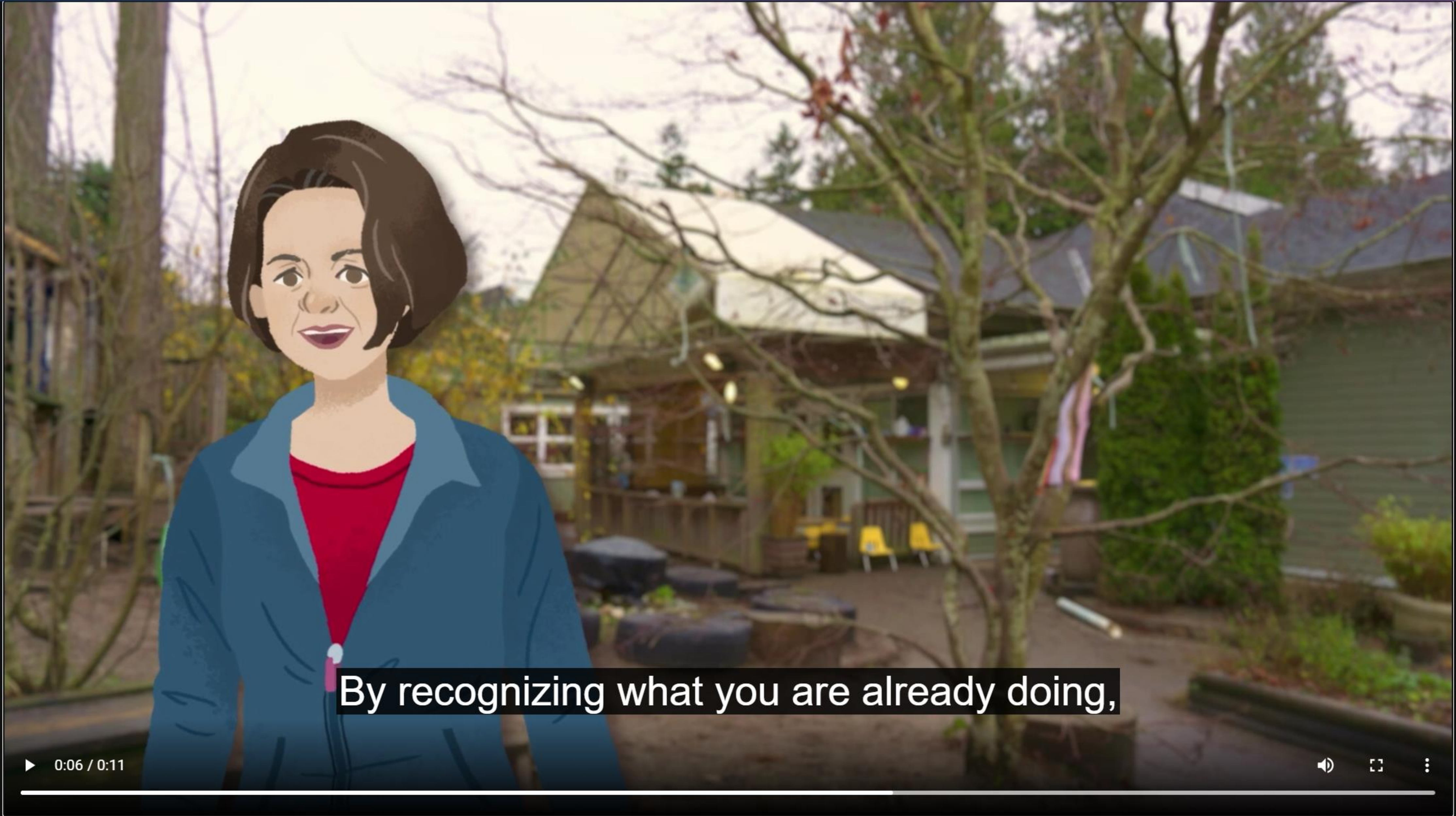
By recognizing what you are already doing,
0:06 / 0:11

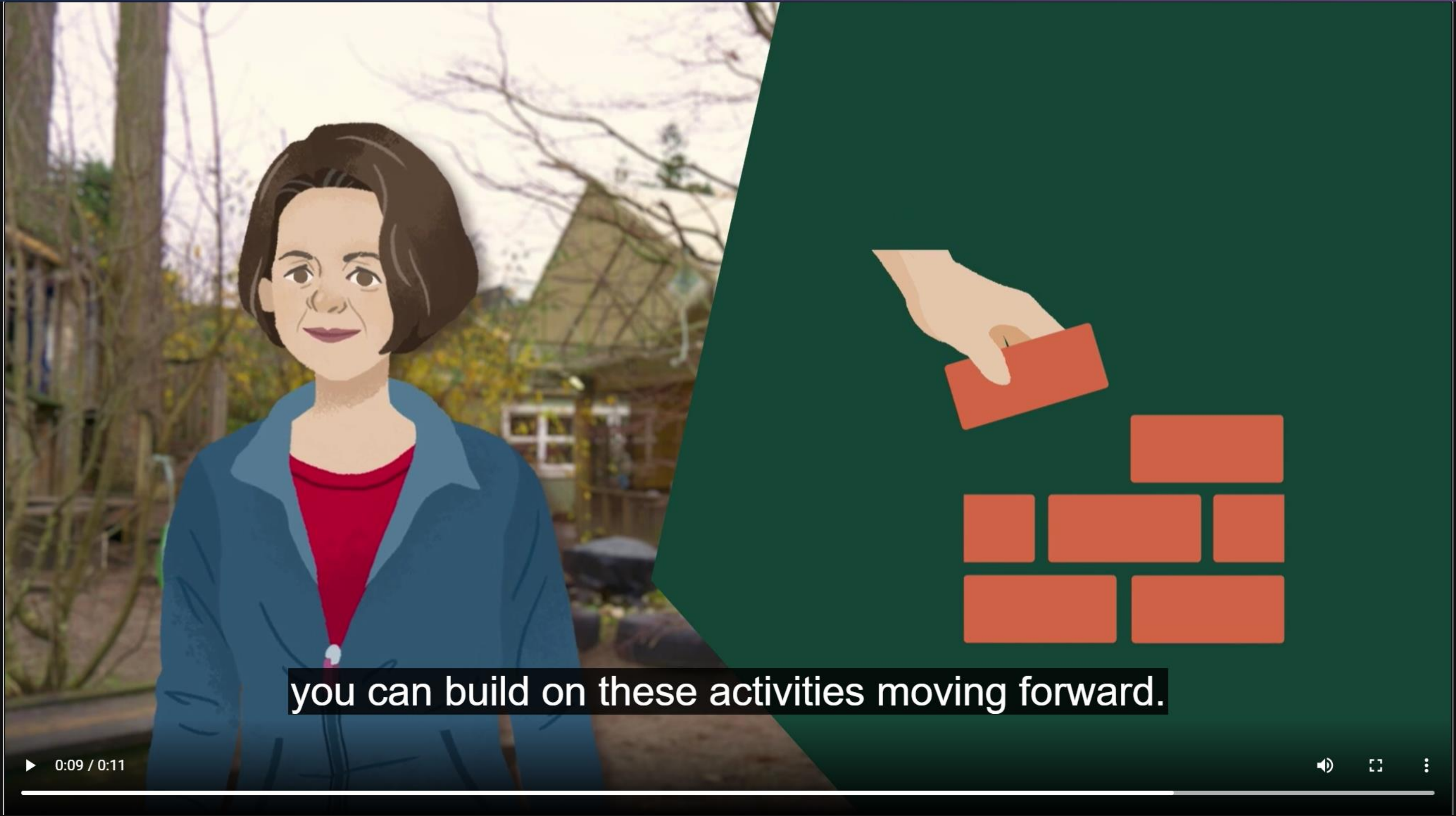
you can build on these activities moving forward.
0:09 / 0:11

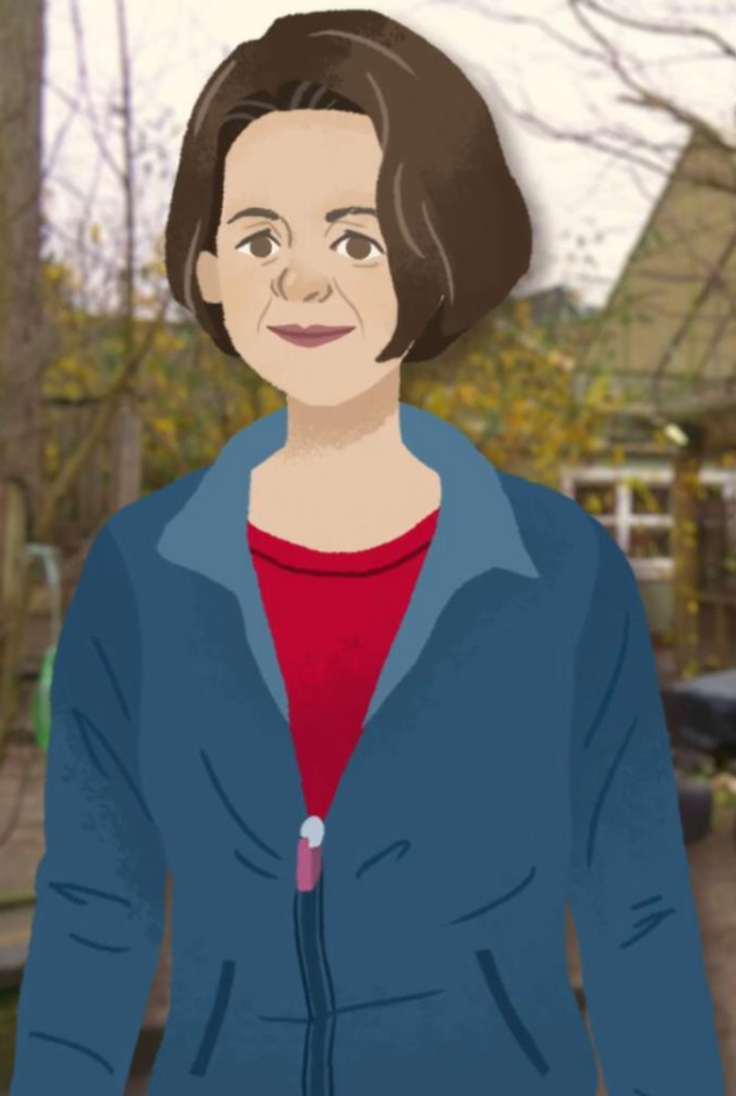

How do you currently support children's outdoor play at your centre? (Select all that apply)

- I make sure children get outside everyday
- I provide loose parts for play
- I make it easy for children to go outside when they want to
- I say 'yes' as much as possible to children's play ideas
- I support children's risk taking
- I conduct risk-benefit assessments to support risk taking while managing hazards
- I provide appropriate supervision
- I join children's play in a non-intrusive way
- I document children's learning and make it visible
- I reflect on my learning [illegible]
- I communicate with other people about the [illegible]
- I seek learning [illegible]

Next

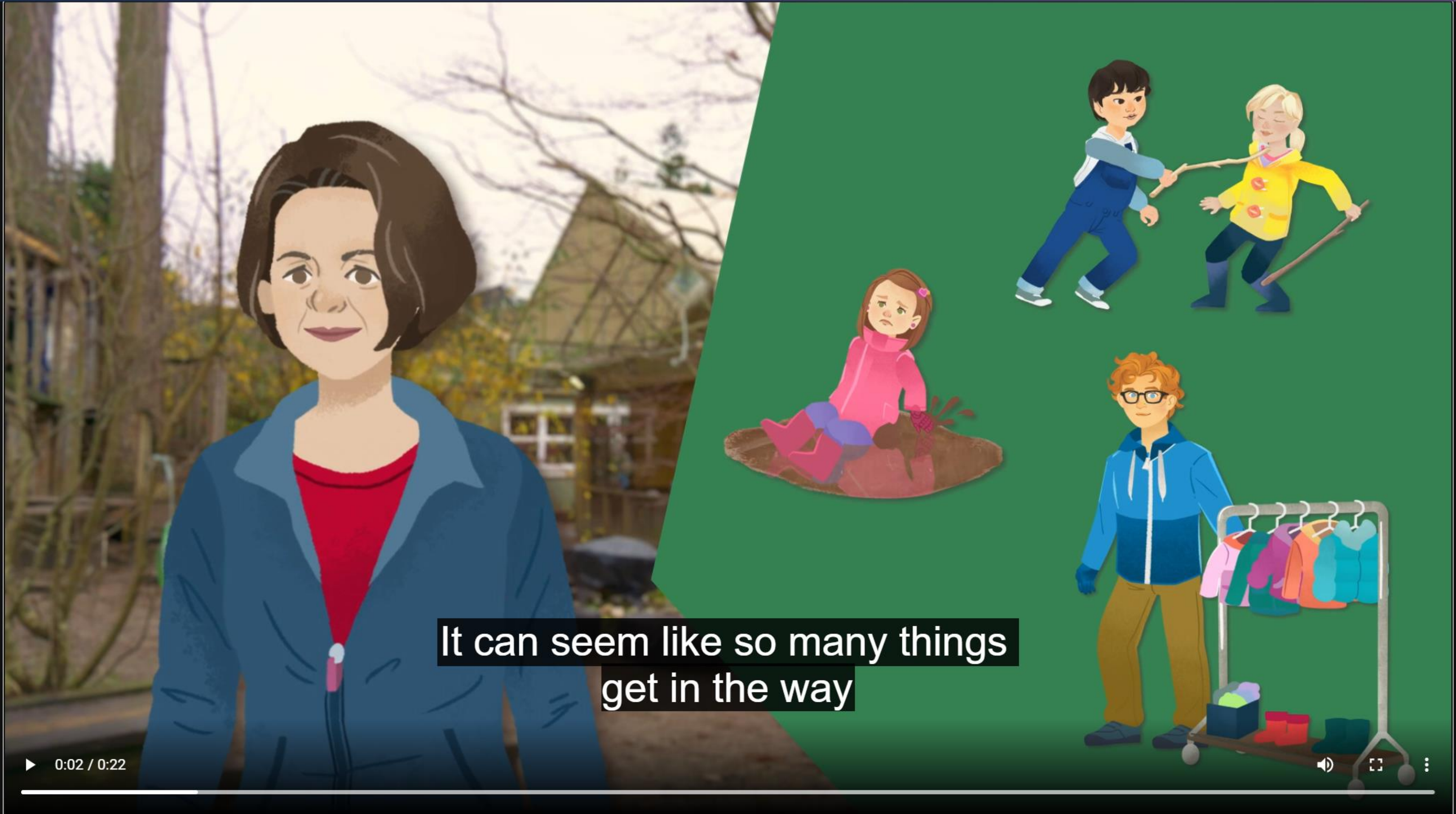
It can seem like so many things
get in the way
0:02 / 0:22

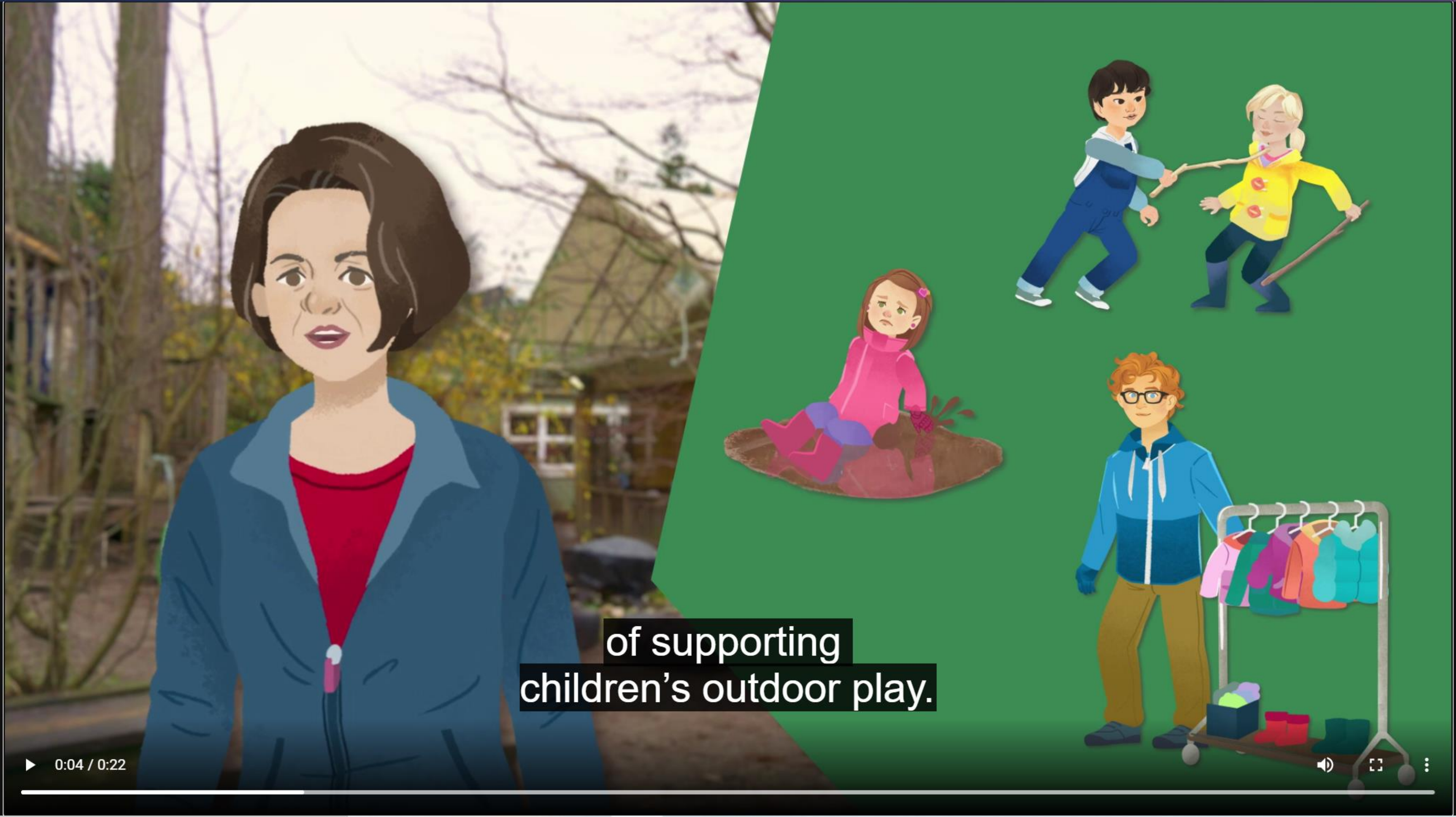
of supporting
children's outdoor play.
0:04 / 0:22

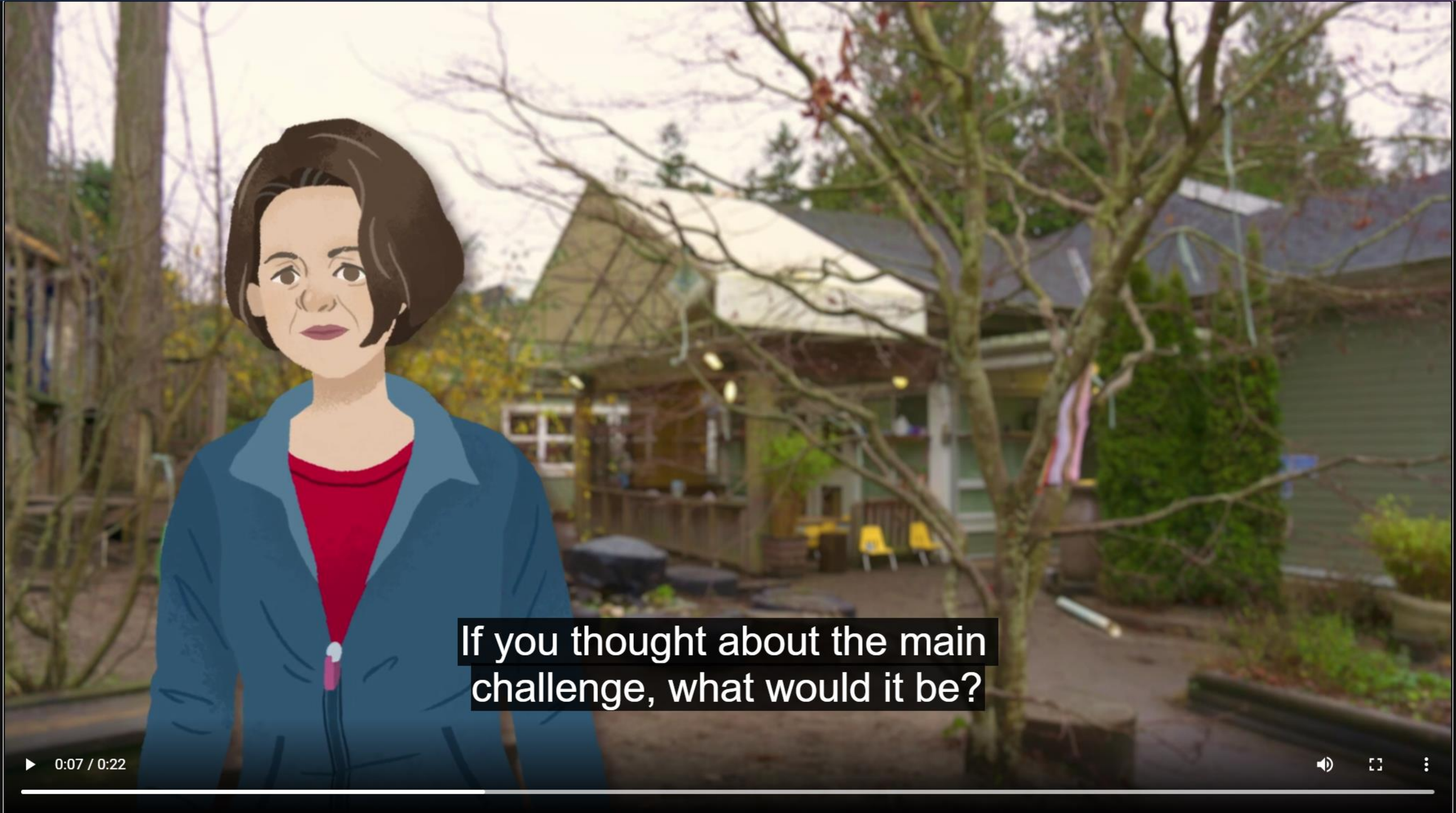
If you thought about the main
challenge, what would it be?
0:07 / 0:22

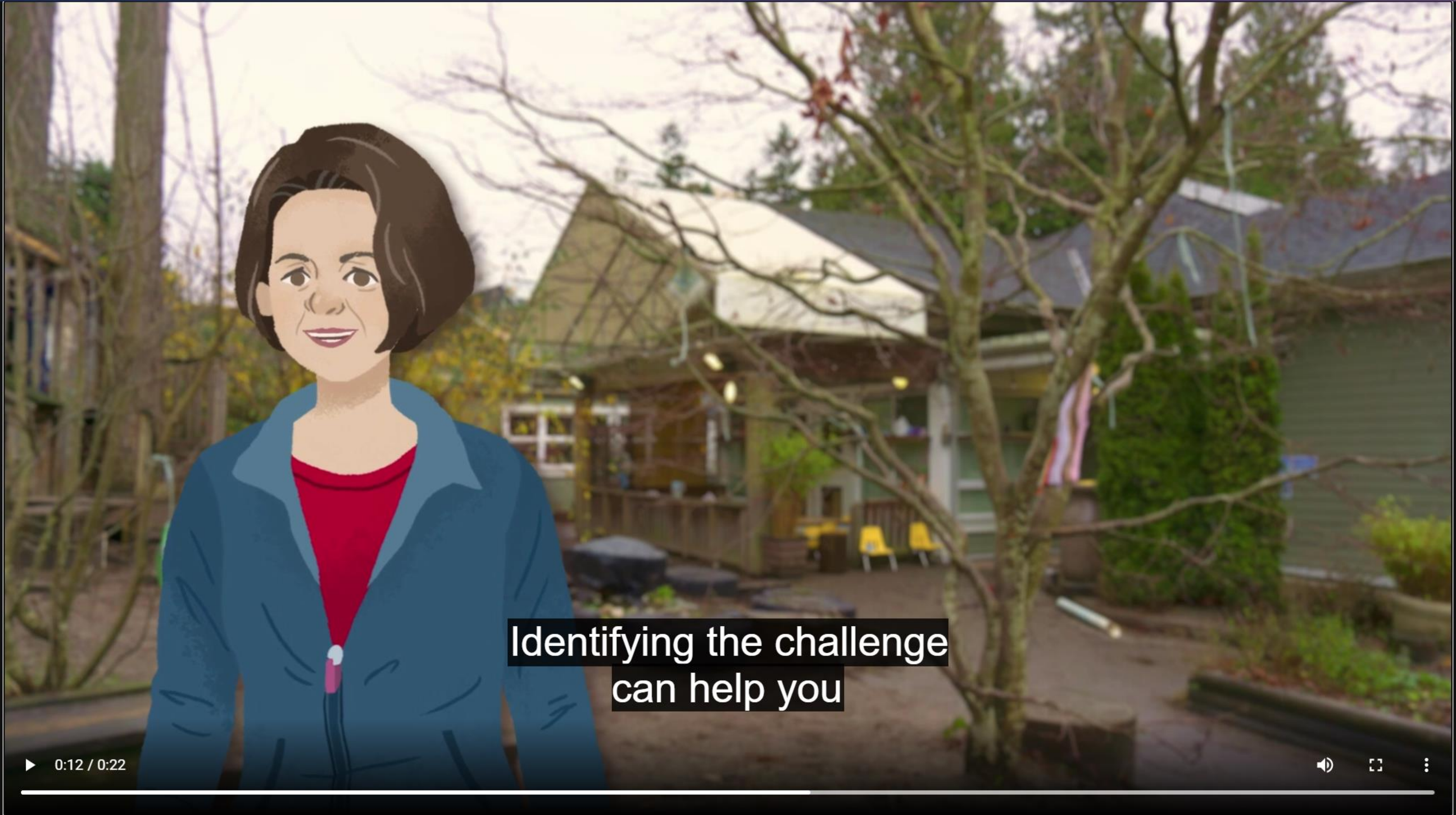
Identifying the challenge
can help you
0:12 / 0:22

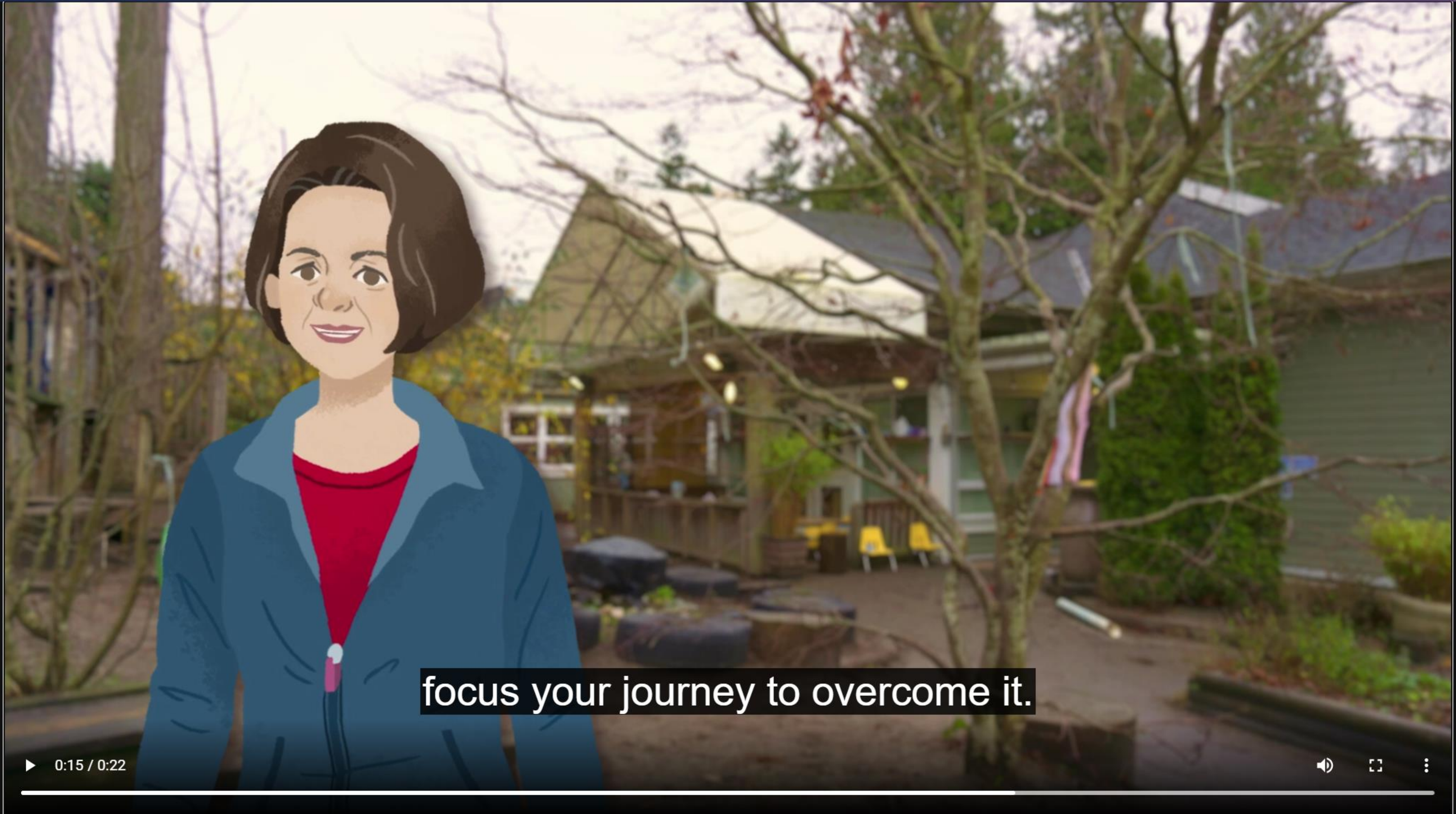
focus your journey to overcome it.
0:15 / 0:22

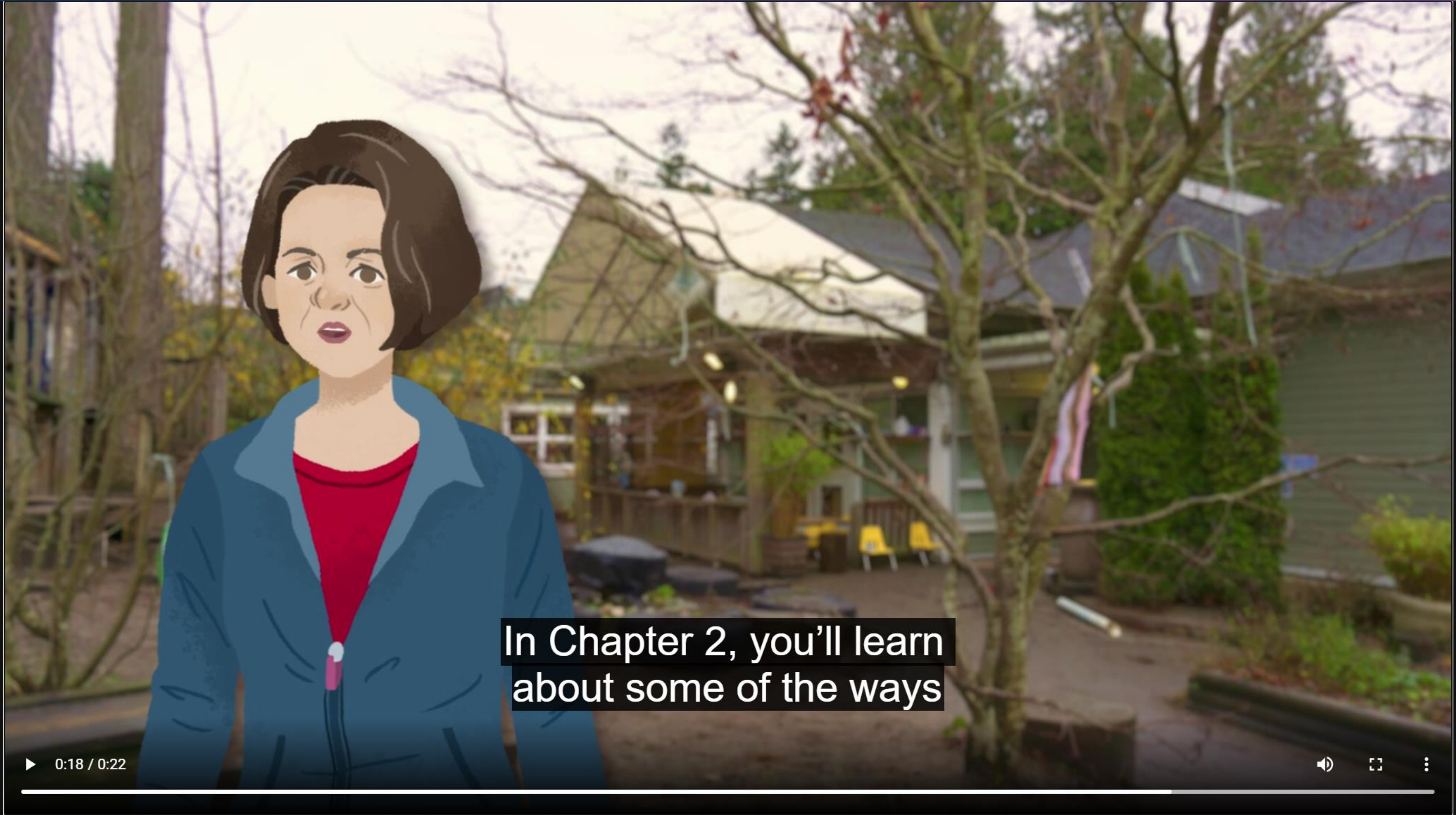
In Chapter 2, you'll learn
about some of the ways
0:18 / 0:22

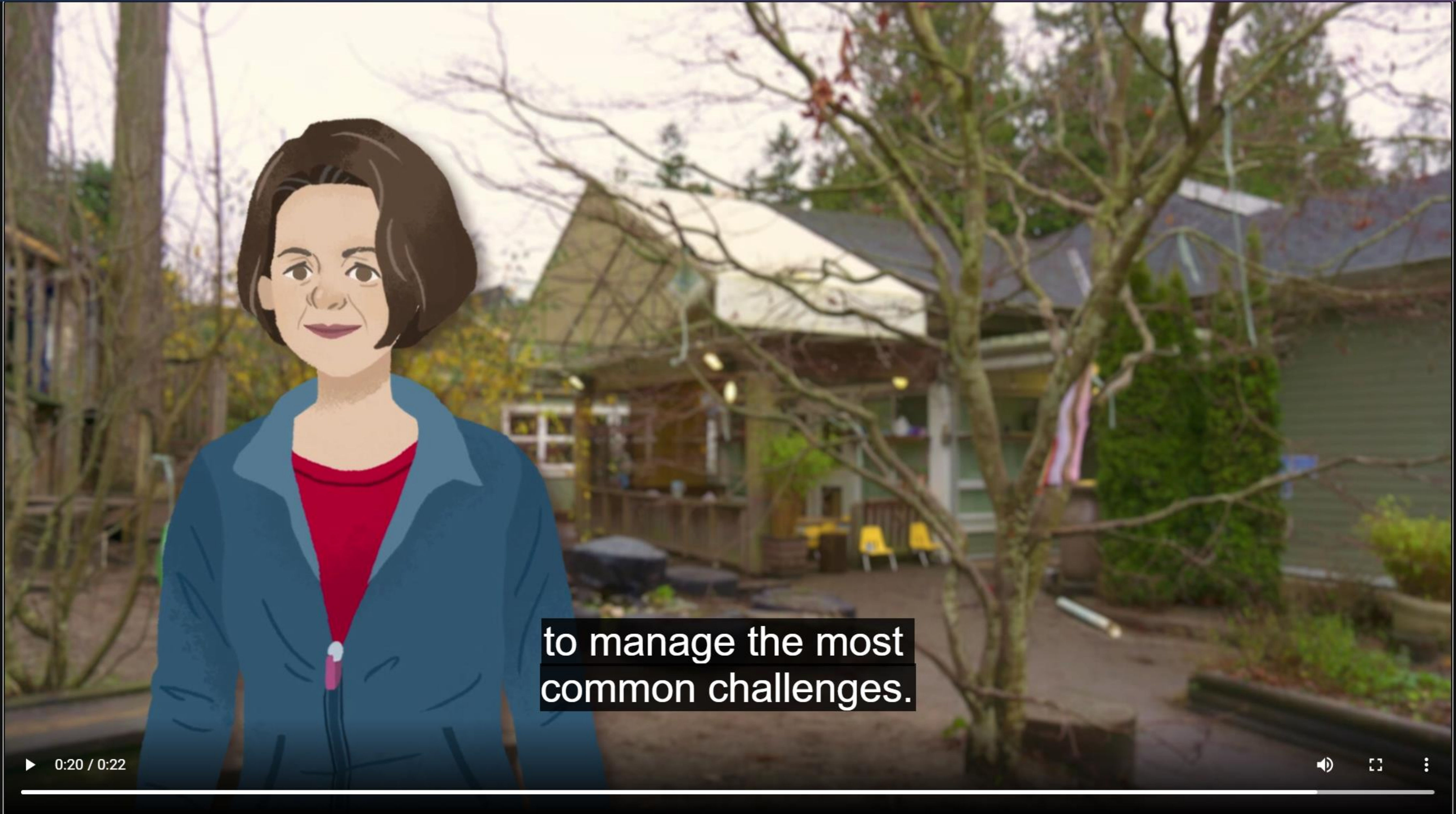
to manage the most
common challenges.
0:20 / 0:22

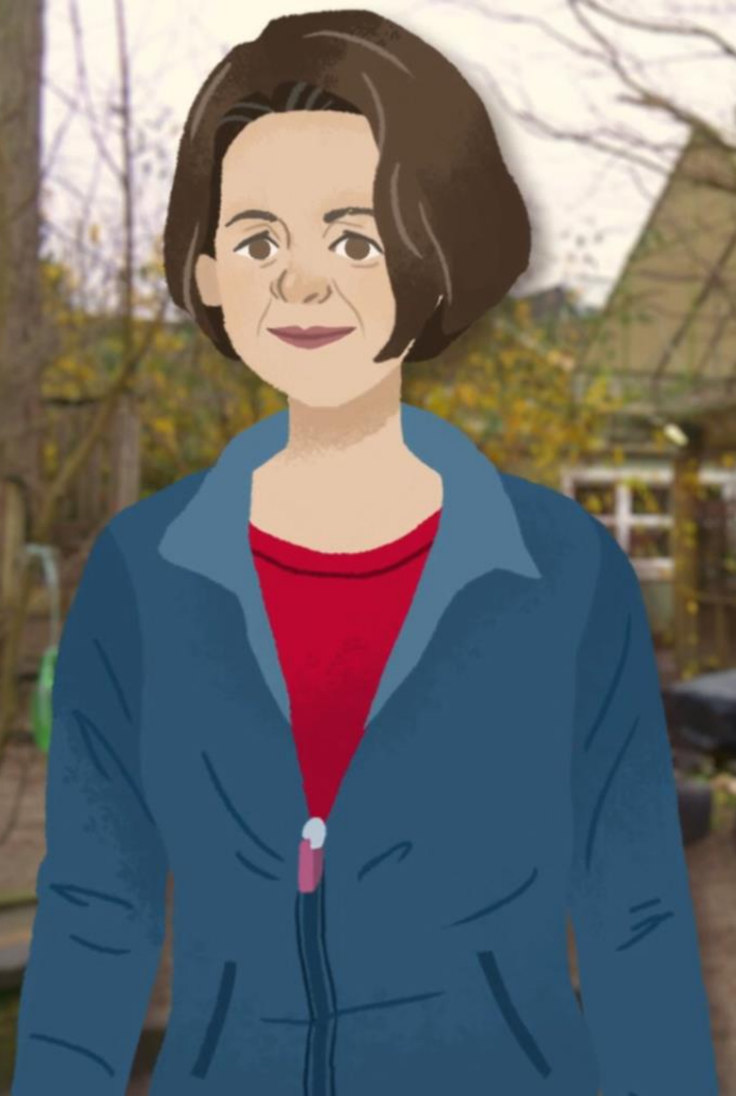

What gets in your way the most in supporting children's outdoor play? (Select one)

I worry about injury or liability	I worry that parents, colleagues or licensing officers don't support outdoor play	My centre's space is not good enough
I don't trust my ability to manage children's risk taking	Children don't know how to keep themselves safe	Our centre doesn't have the right clothing/gear for being outside
I don't like being outside, especially in bad weather	We don't have enough time for outdoor play	Other

Next

Chapter 1 Summary

Were you inside or outside?

Outside

Were you taking risks?

Yes

What was your favourite thing to do?

- Playing group games, such as tag or hide-and-seek
- Cycling, skateboarding, or scootering
- Climbing trees or structures
- Playing on playground equipment
- Imaginative play

What did you get out of it? How did this experience influence you?

- Courage
- Curiosity
- Creativity
- Independence
- Physical competence

Next

Chapter 2:

Scenarios

Imagine yourself as the educator in each interactive video scenario. Think about what you would do in these situations. Your choices will impact what happens in the story and the outcomes. Think about what influenced your decisions and hear from other educators as they share their perspectives on each situation.

CONTINUE

Chapter 2:

Scenarios

Click on a scenario to start the experience!

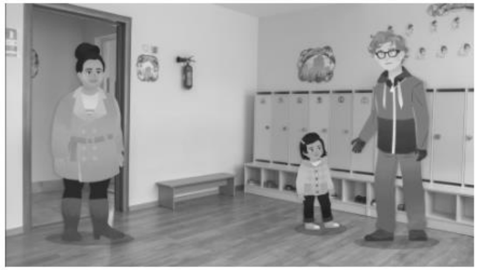

Communicating with Parents/Caregivers

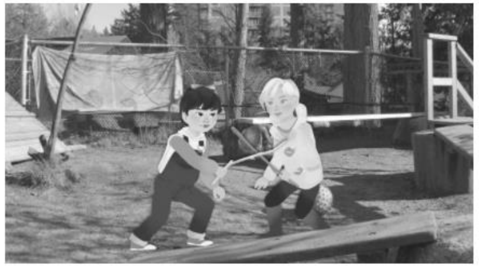

Rough and Tumble Play

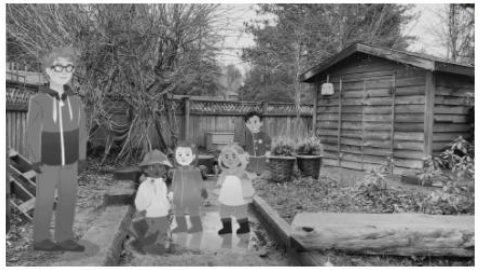

Play at Speed

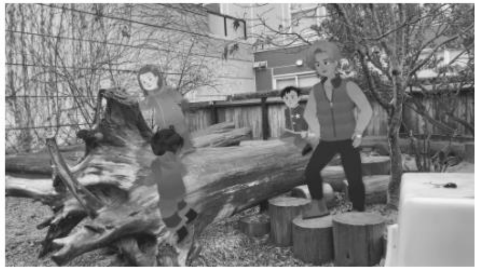

Play at Heights

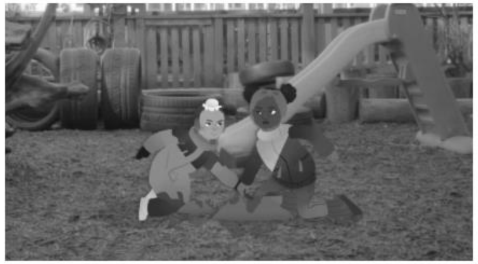

Conflict Resolution

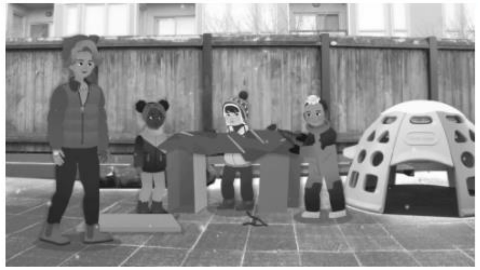

Play with Loose Parts

Scenarios

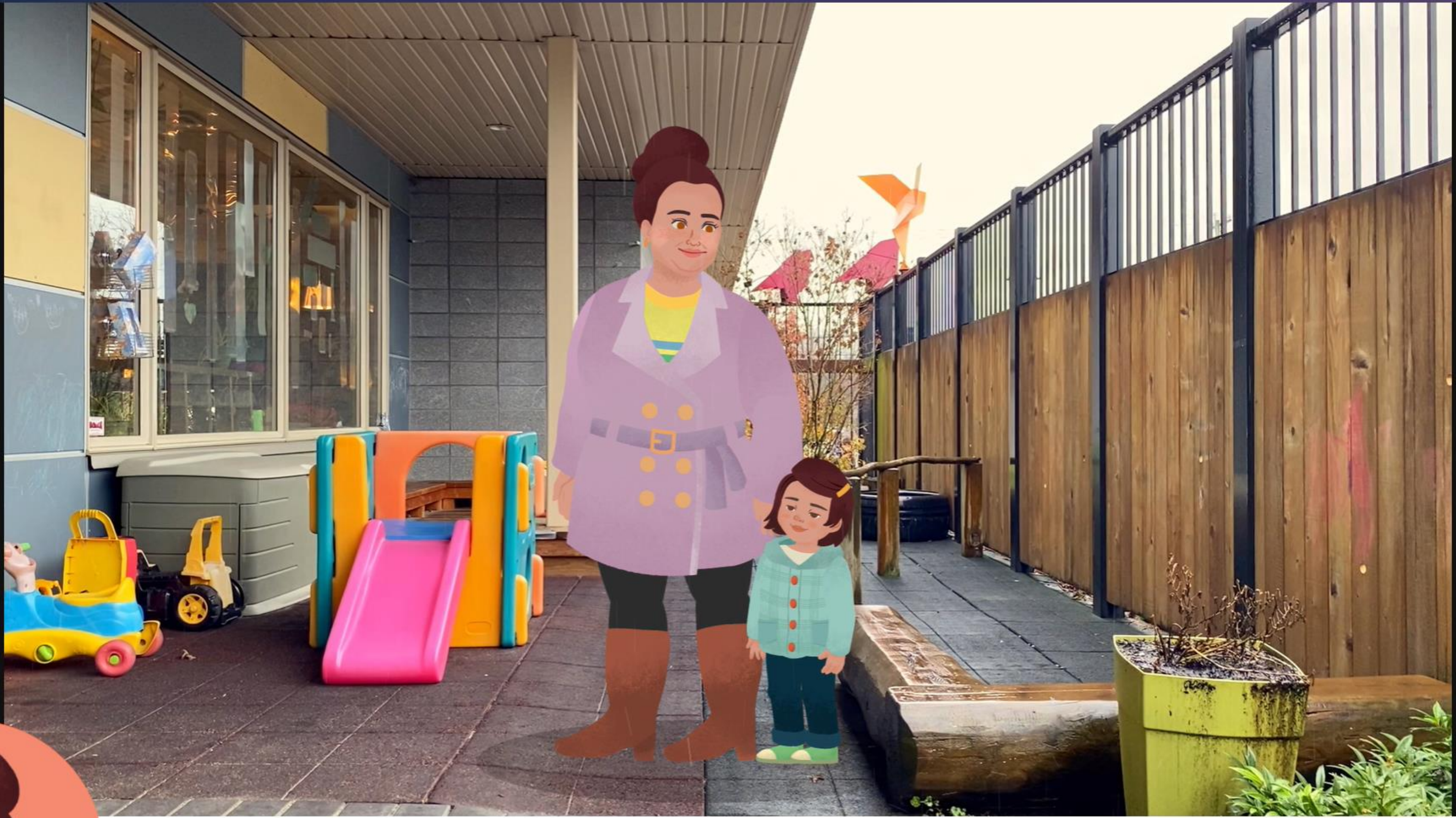

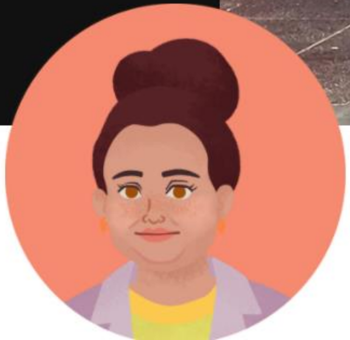

"Are you excited to see your friends?"

NEXT ▶

Take a risk, go play outside!

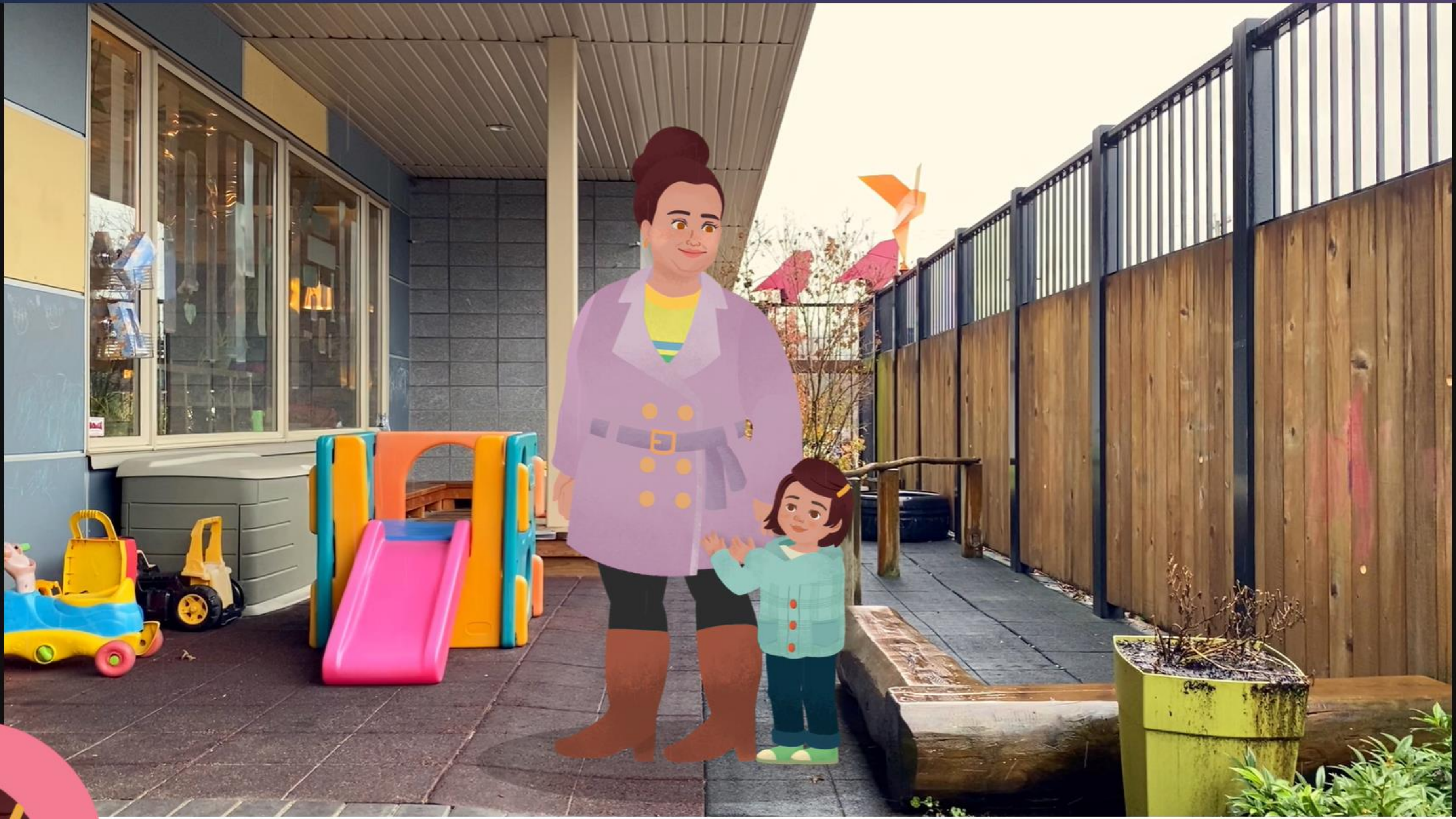

Scenarios

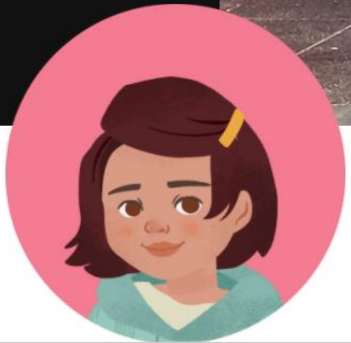

"Yes!"

NEXT

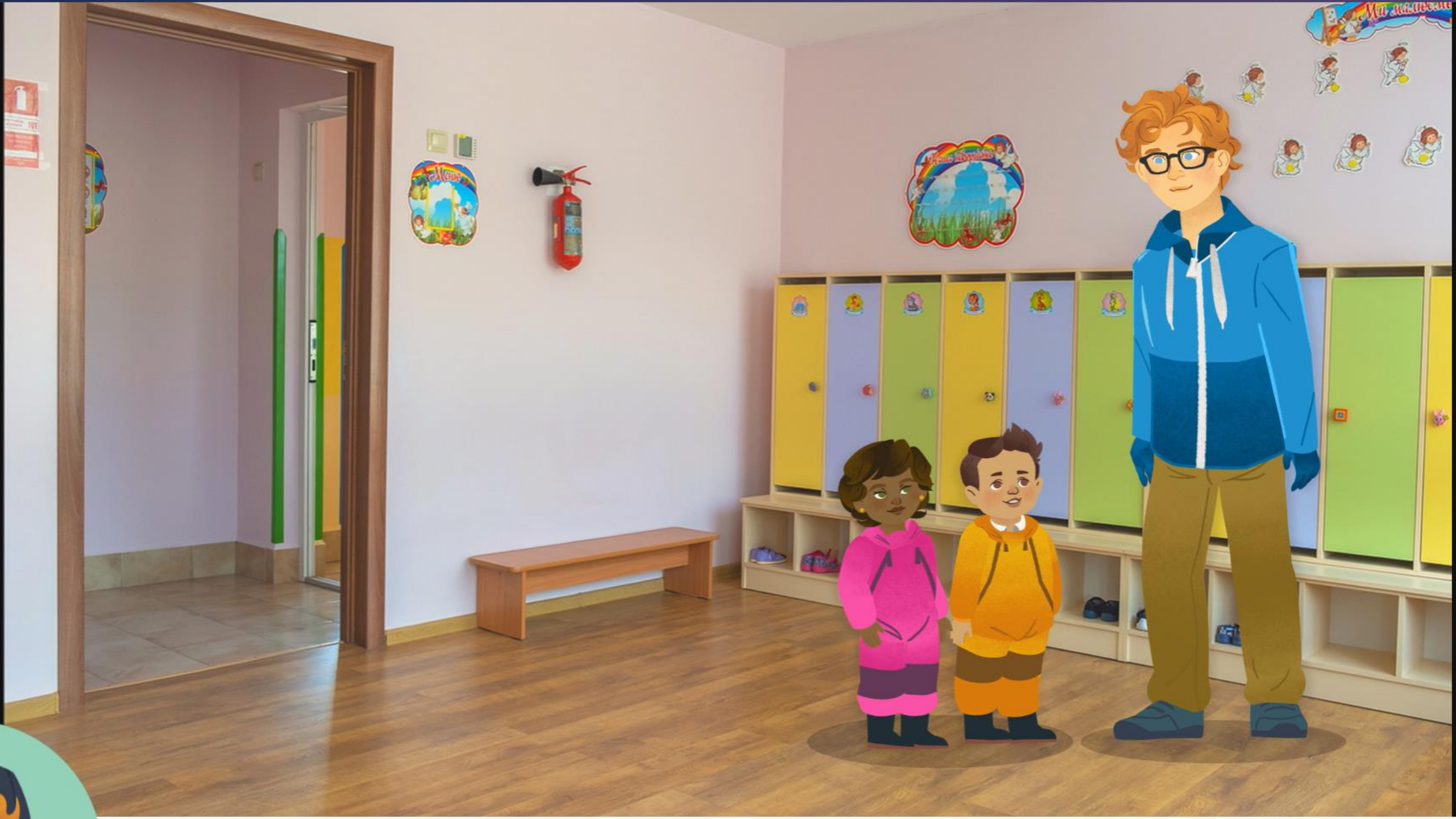

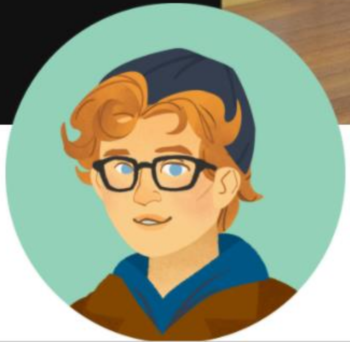

"It rained last night and we have lots of puddles to play with today!"

NEXT

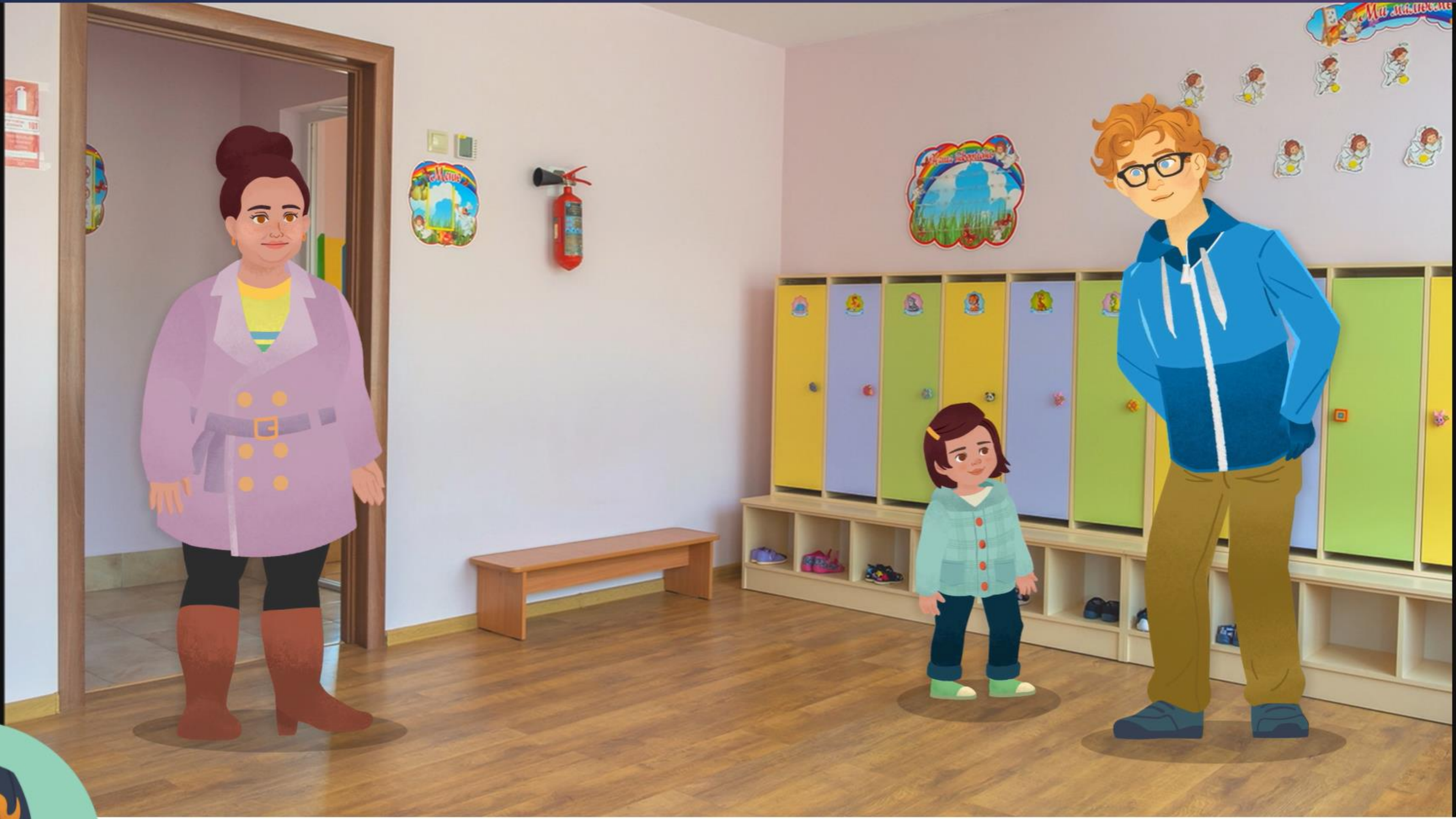

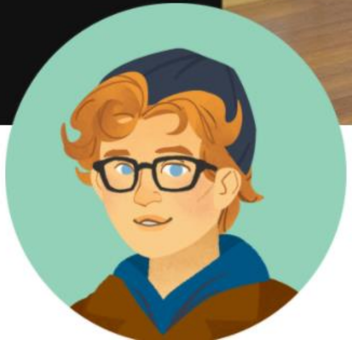

"Good morning! Are you excited about playing out in the puddles today, Kate?"

NEXT

Scenarios

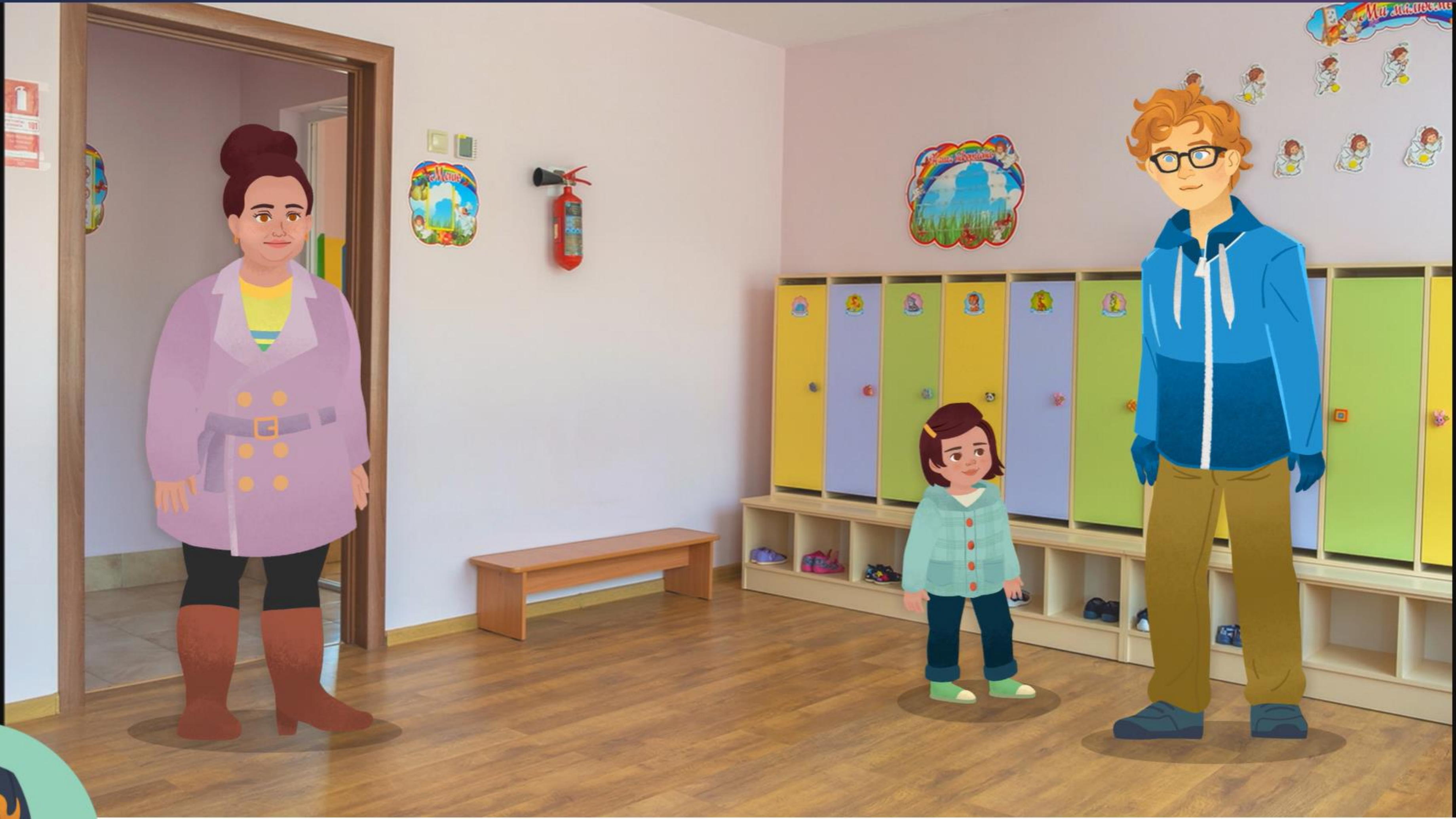

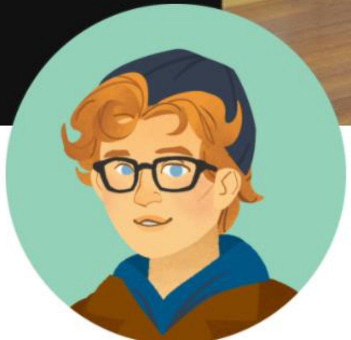

"We're going to head straight outside this morning. Is your raingear in your cubby?"

NEXT ▶

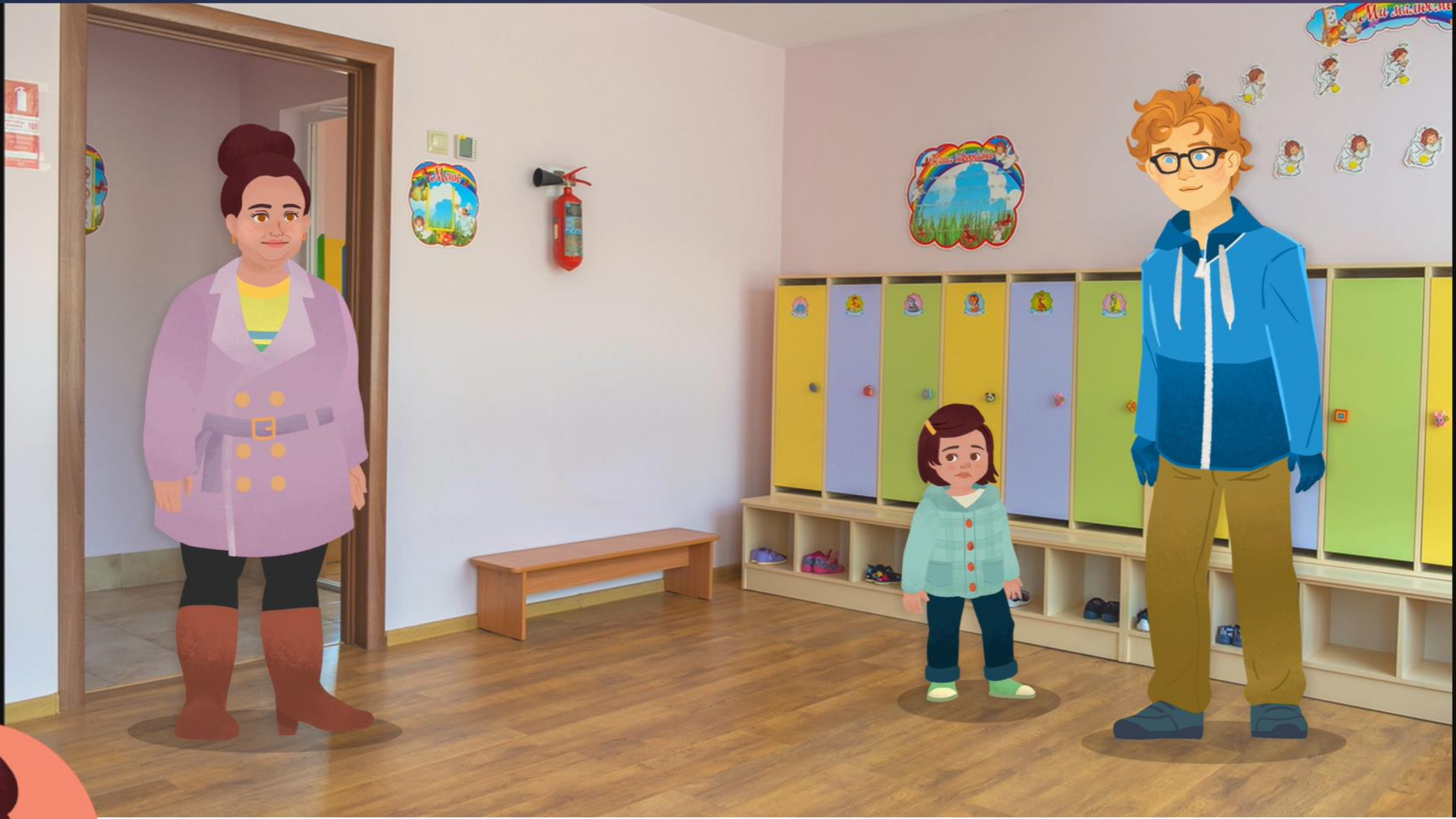

Scenarios

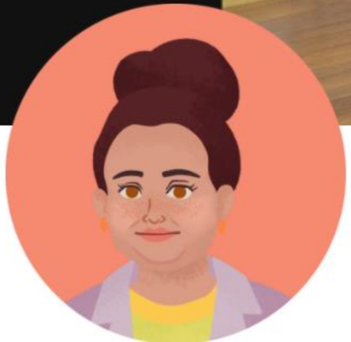

"It's such a cold and rainy day, I don't think it's a good idea for Kate to be outside today. I'd like her to stay inside where it's warm and dry and she won't get sick or dirty."

NEXT

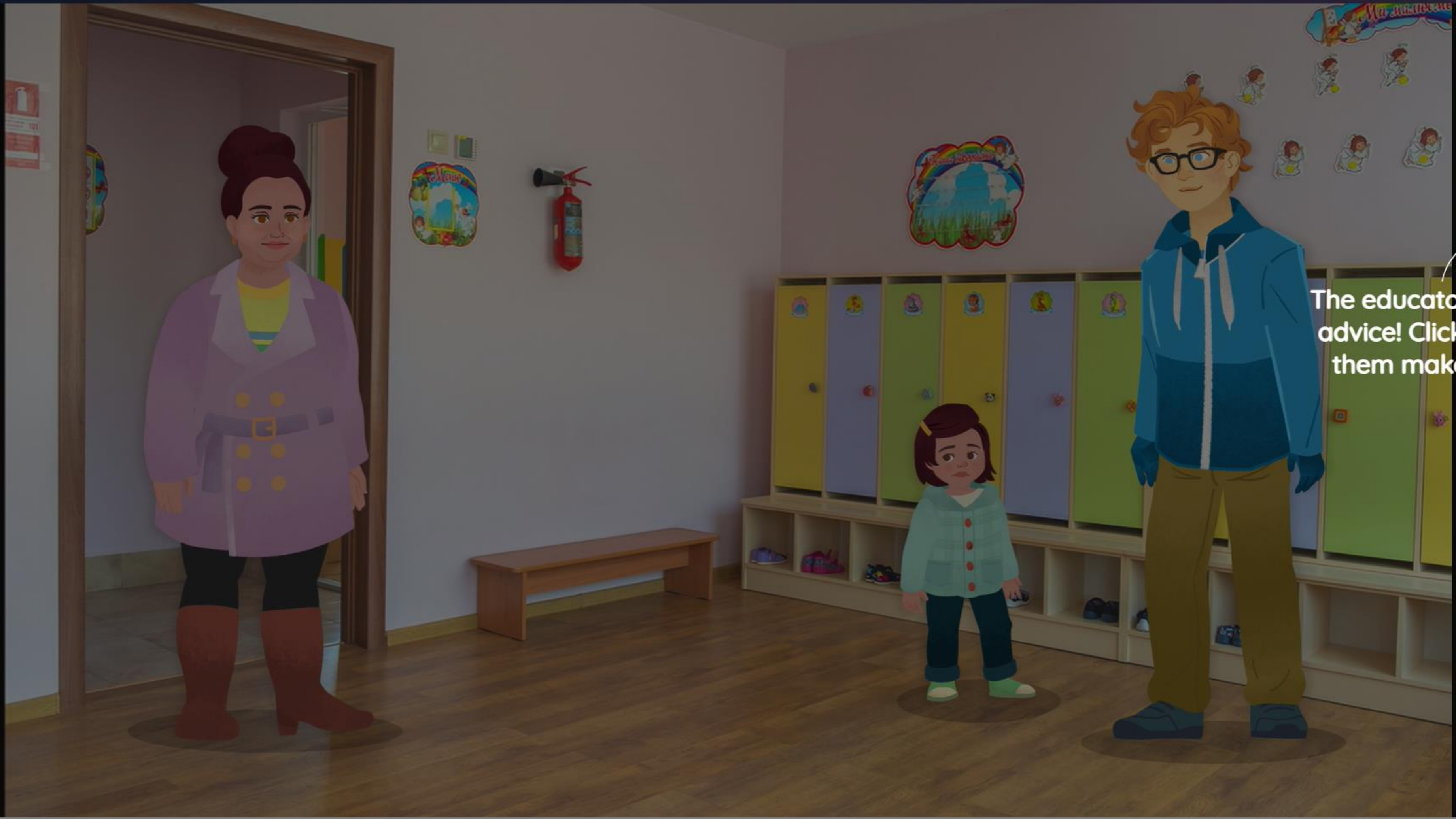

Scenarios

The educator needs some advice! Click here to help them make a decision.

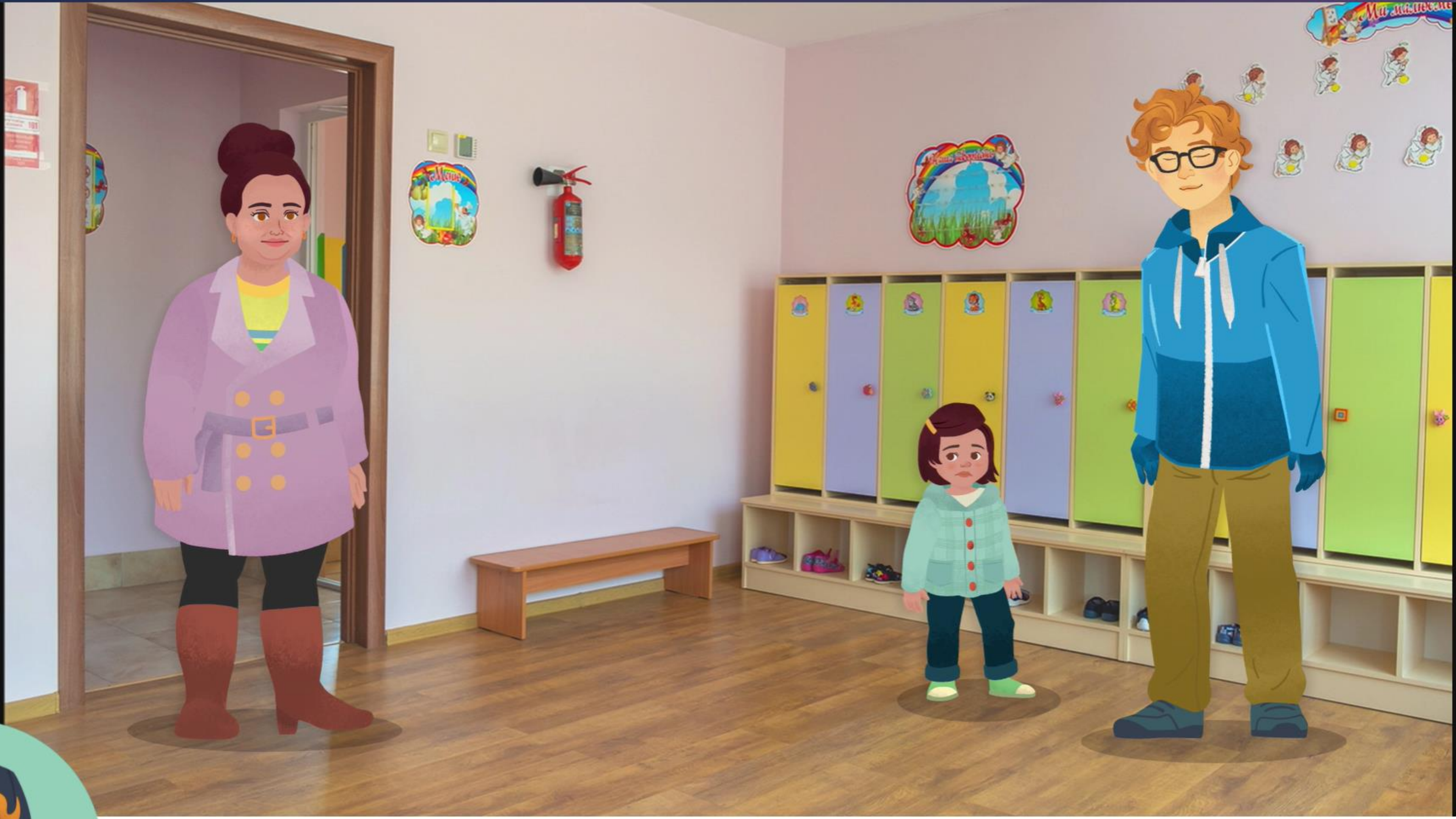

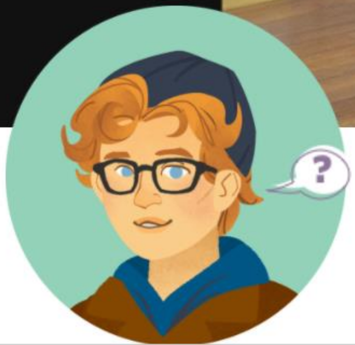

"What should I do?"

▶ Keep Kate warm and dry with indoor activities

▶ Get Kate kitted up from the gear library

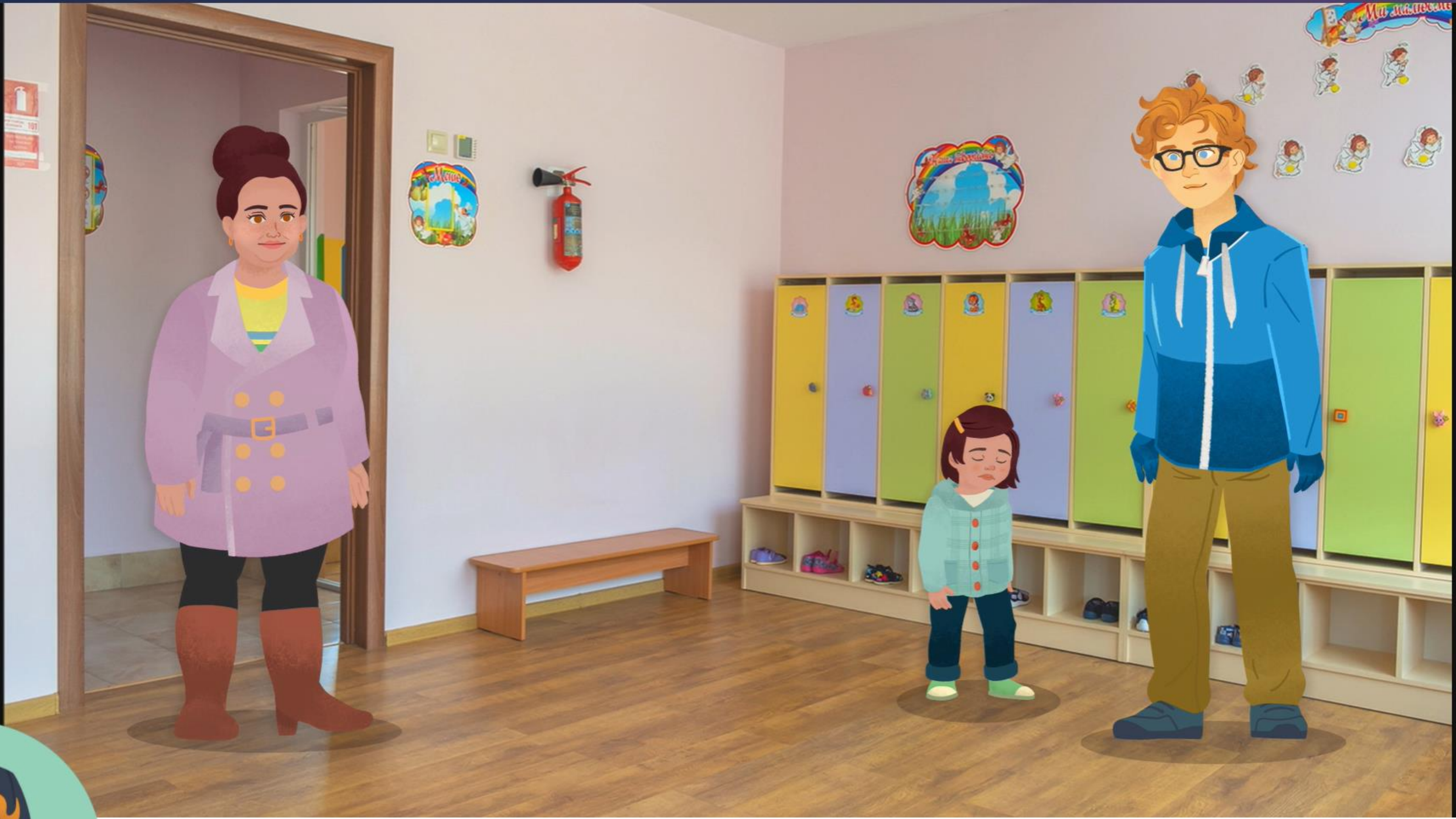

Scenarios

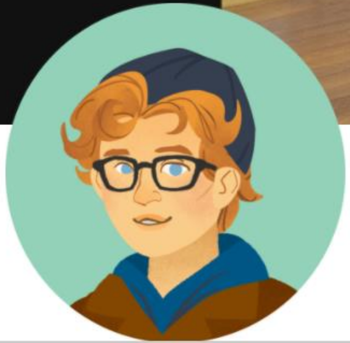

"Okay, we'll make sure Kate has plenty to do indoors today."

NEXT

Scenarios

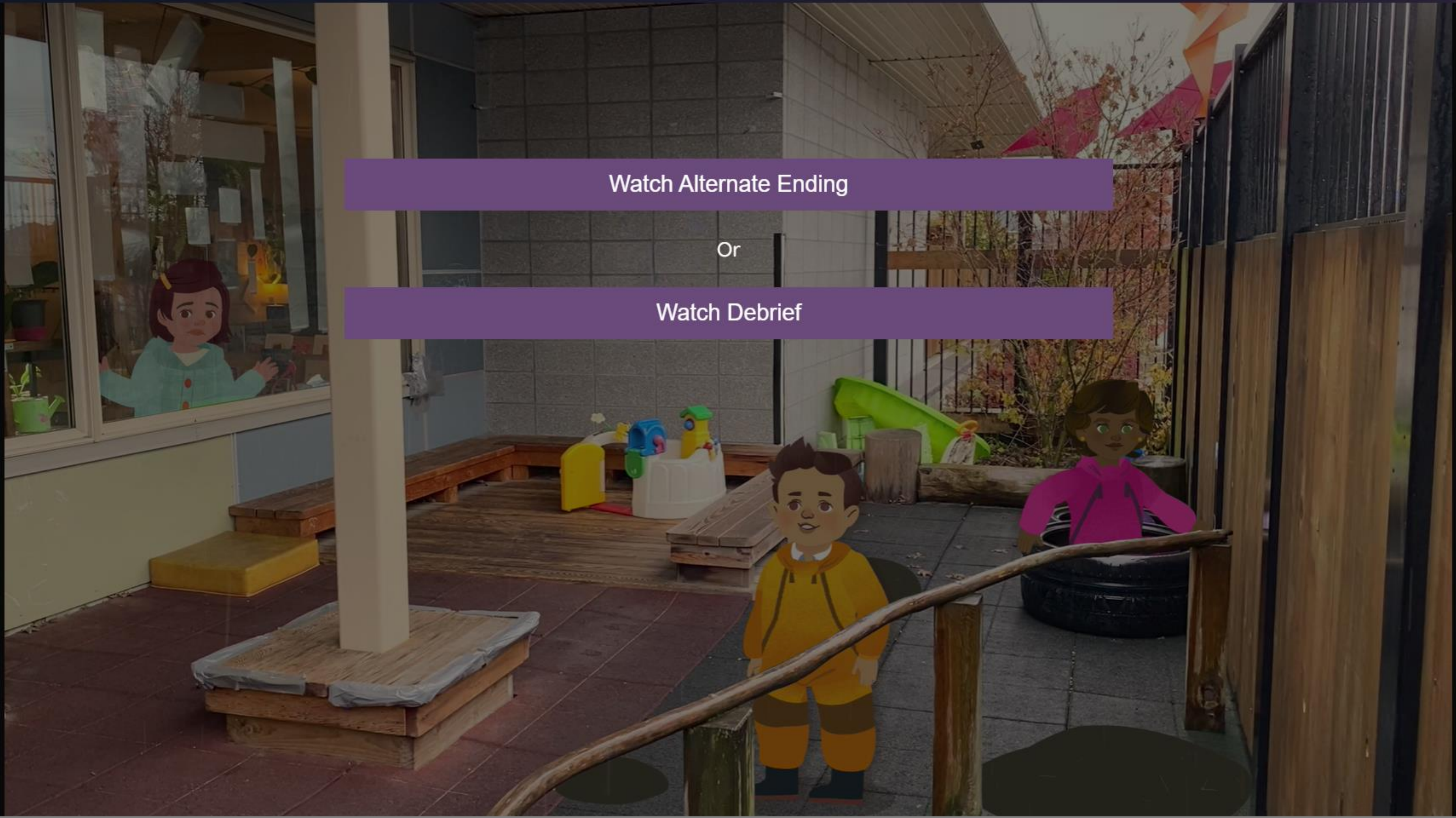

SKIP ▶

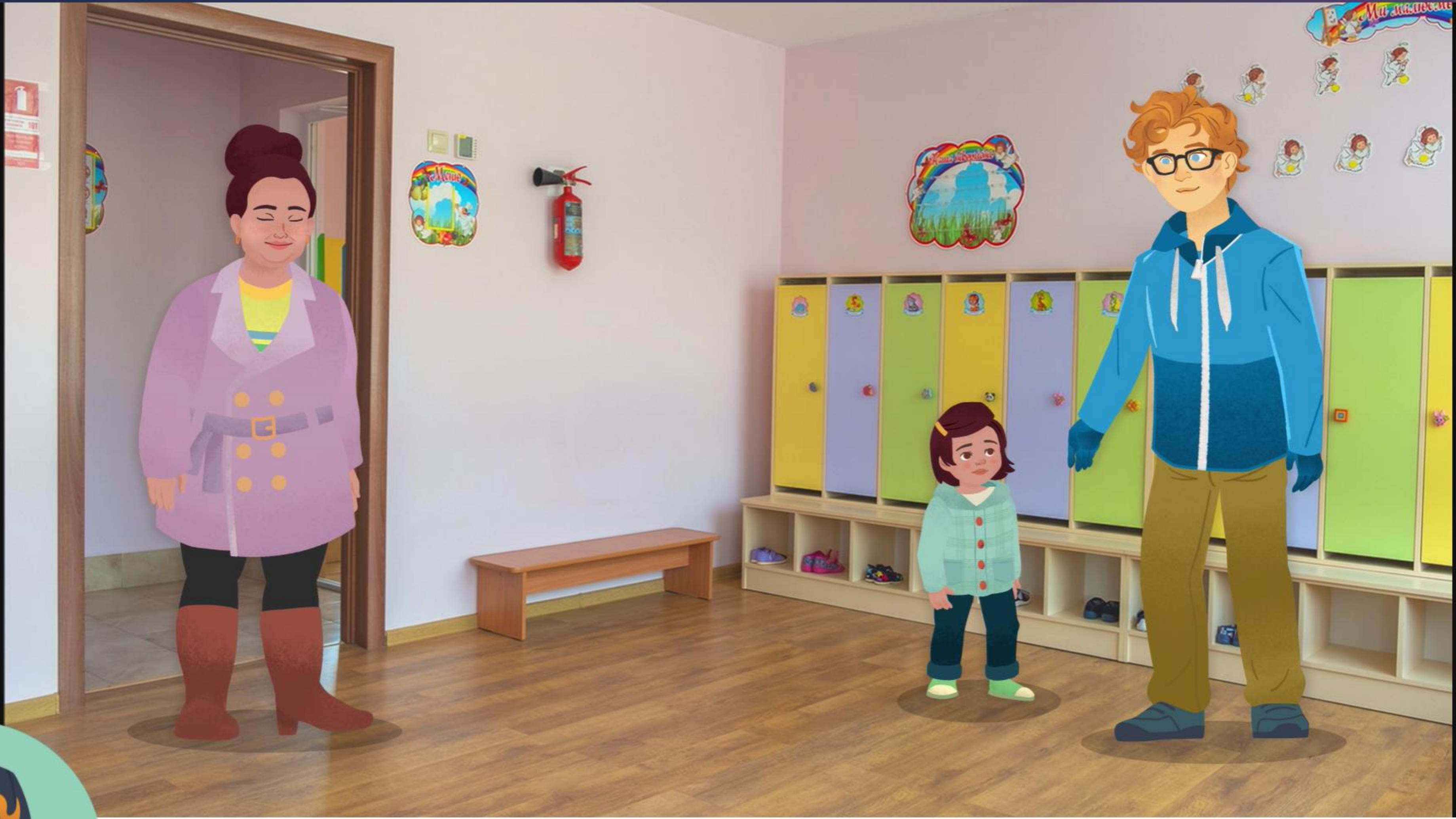

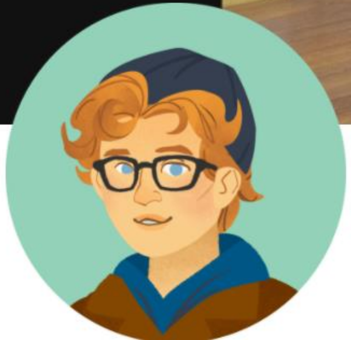

"I understand that you want Kate to be healthy and safe and that it could be hard to feel that she will be ok outside."

NEXT

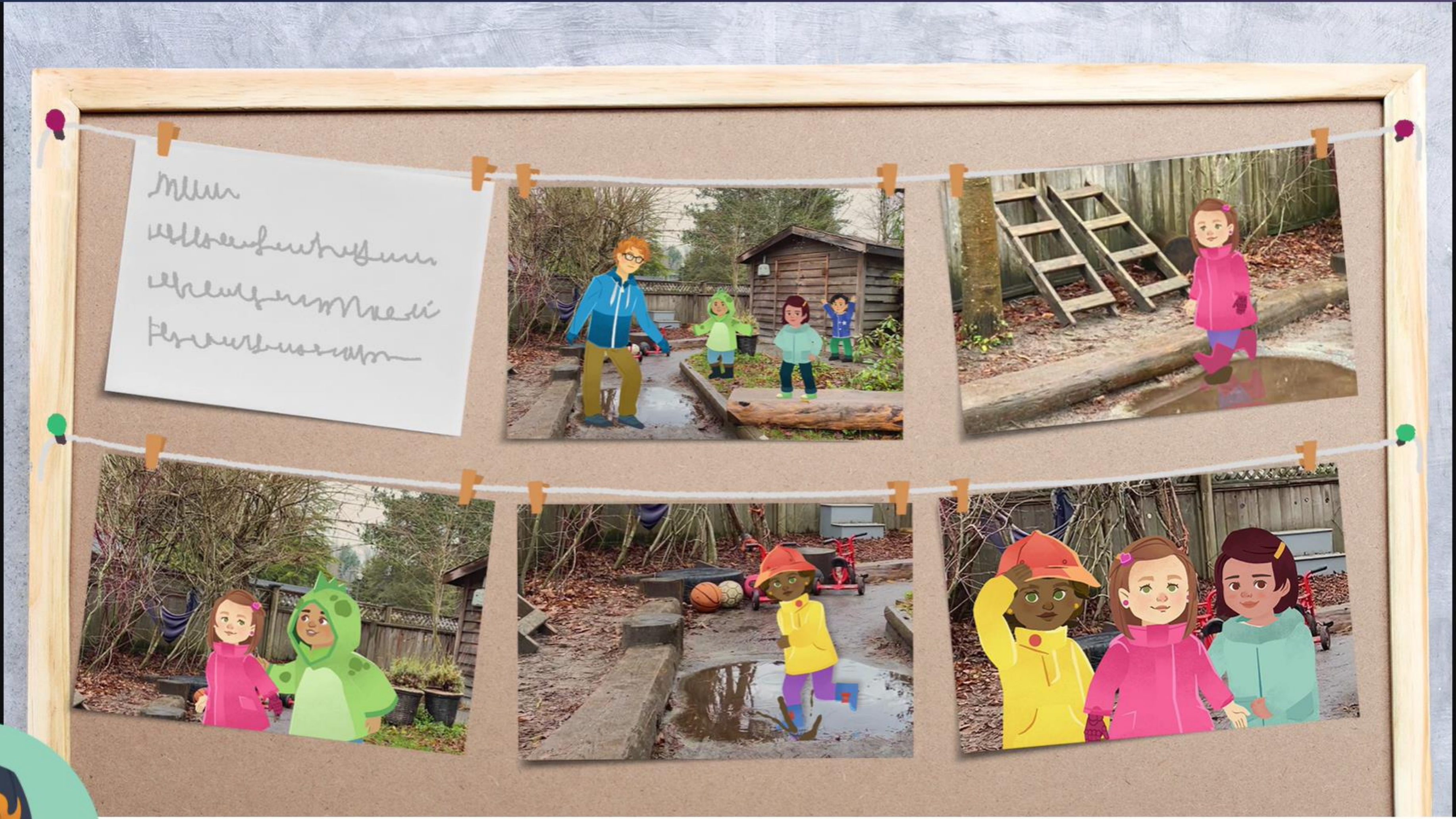

Scenarios

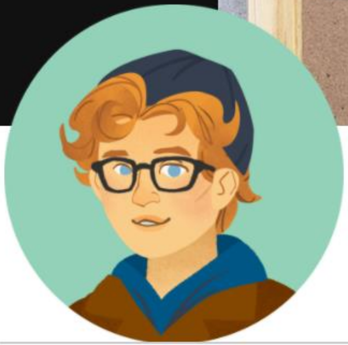

"At this centre, we really want to make sure that the children spend a large portion of their time playing outdoors because it comes with a wide range of benefits that they can miss out on when they stay inside."

NEXT ▶

Take a risk, go play outside!

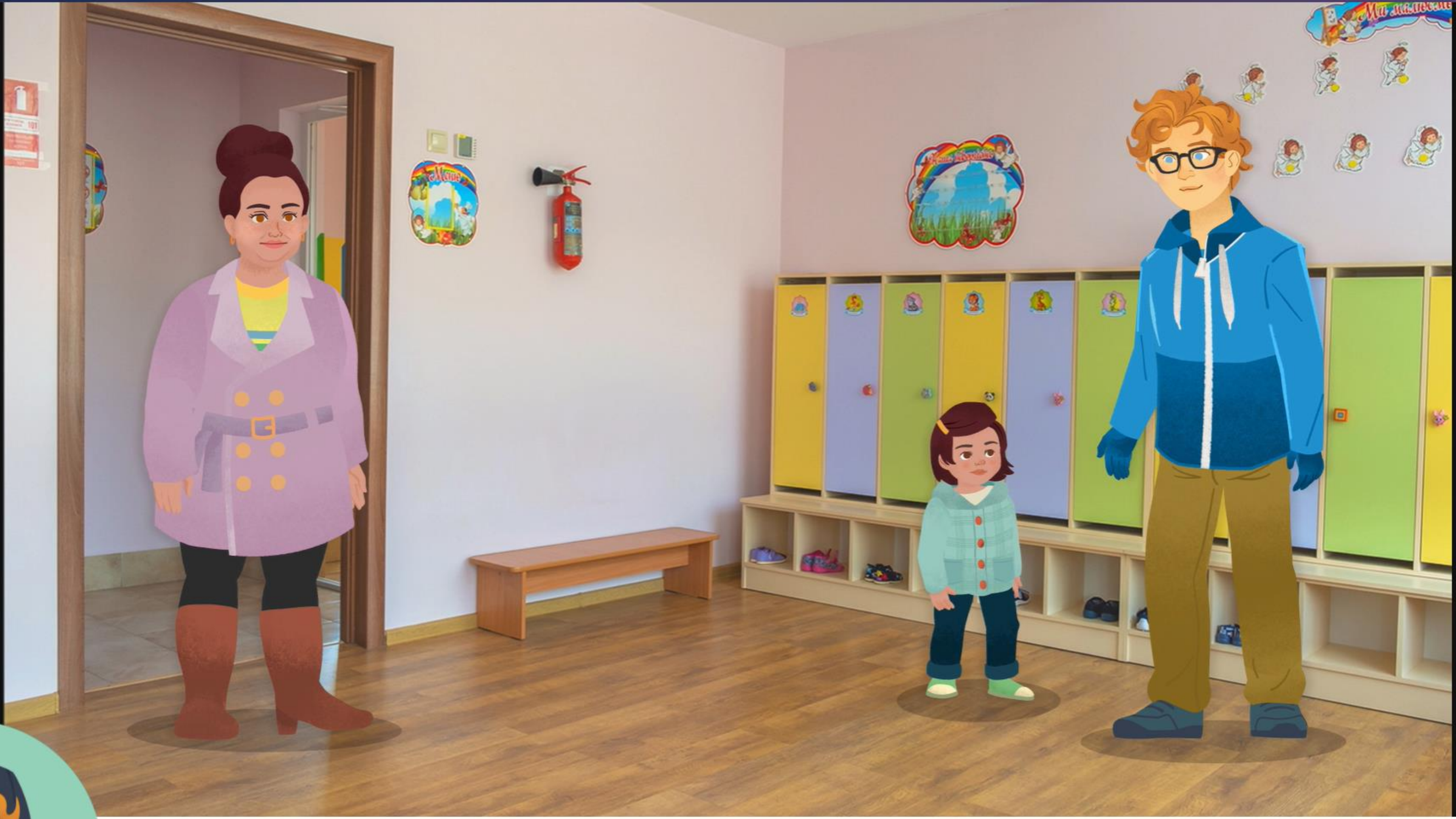

Scenarios

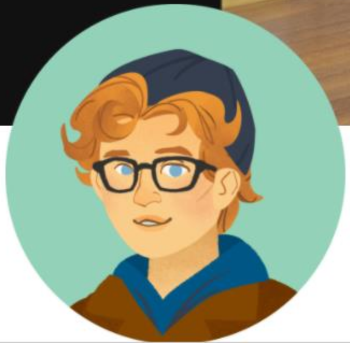

"How about we give it a try today and you and I talk again at the end of the day? I can share what Kate did today and what she learned and how she benefited."

NEXT

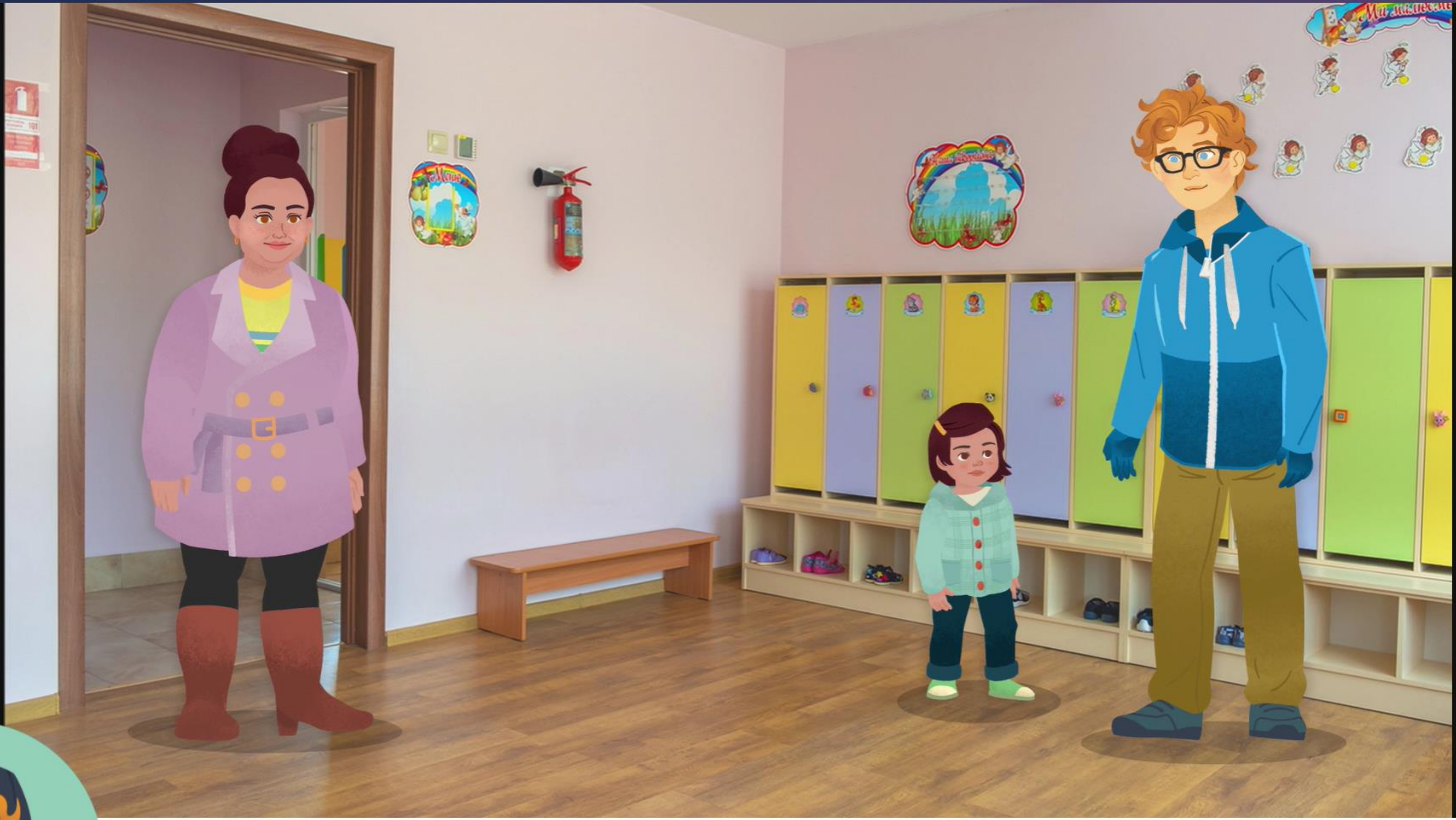

Scenarios

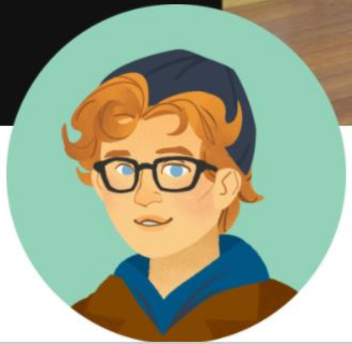

"That might make it easier to see how outdoor play could be important for Kate in particular. You are also welcome to come by anytime and see Kate playing and exploring outside. You can see how engaged she is playing, creating and learning outside."

NEXT

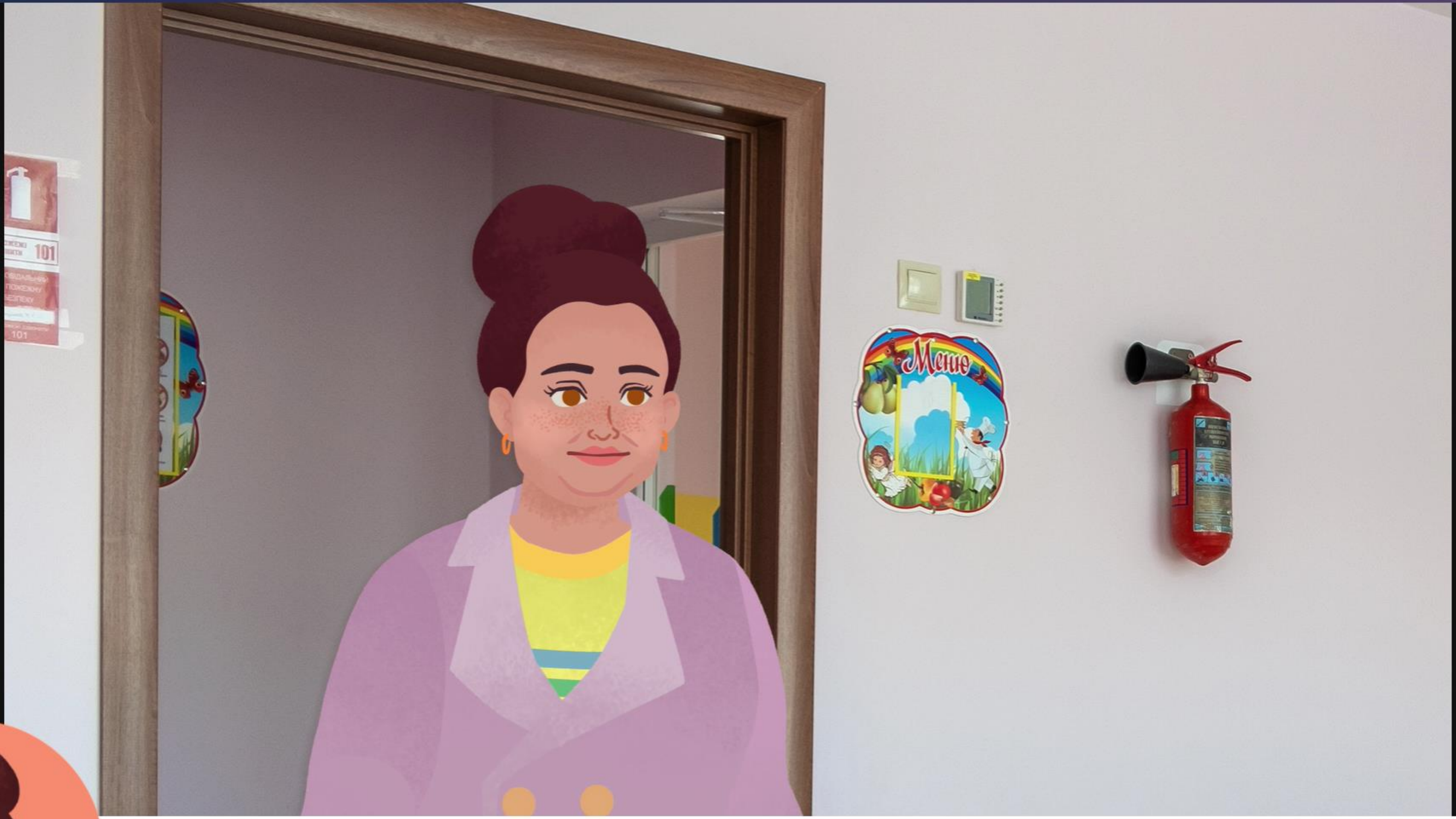

Scenarios

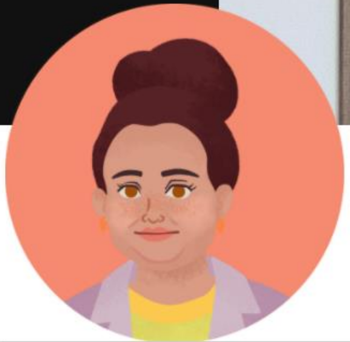

"Okay, let's see how today goes."

NEXT

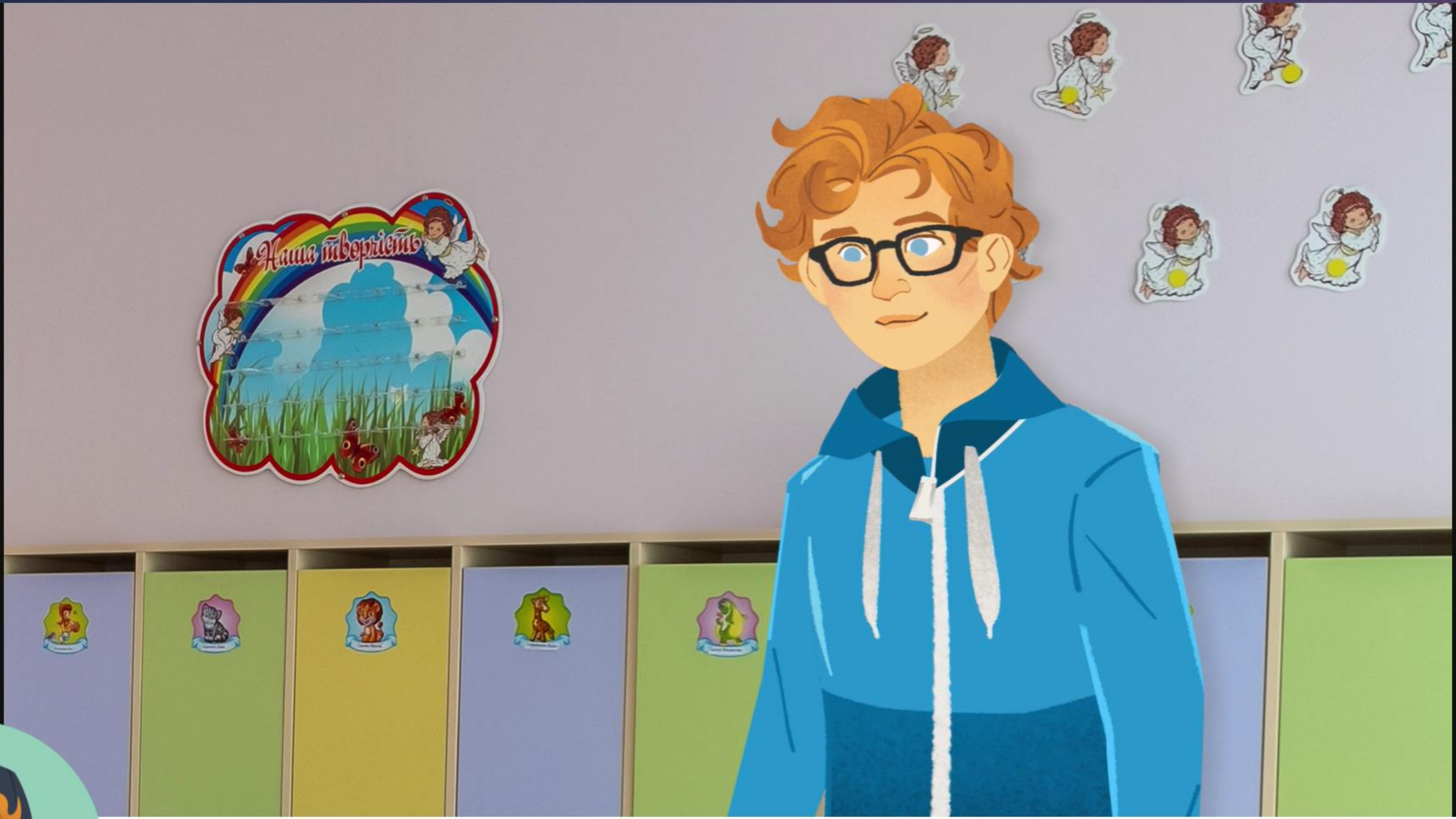

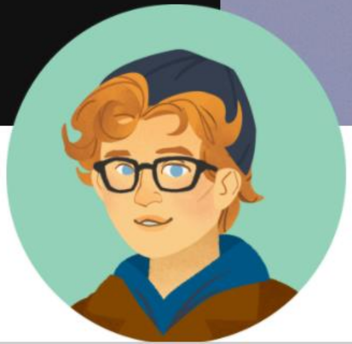

"Great! The first step is to make sure Kate is in the right gear for the weather so that she is comfortable and warm while outside."

NEXT

Scenarios

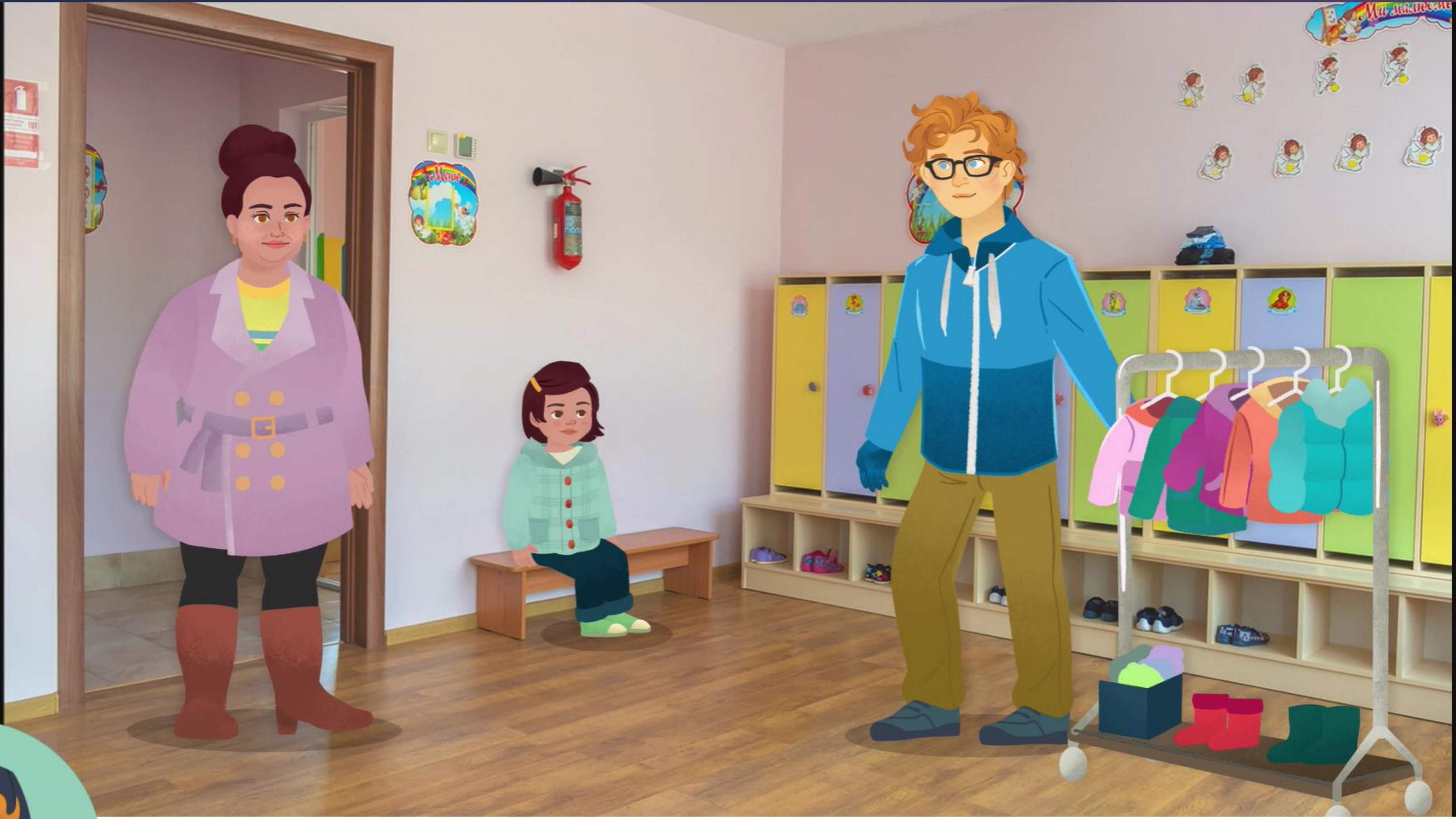

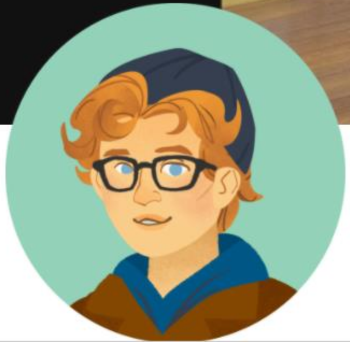

"This is our gear lending library. It’s full of gear of different sizes for different types of weather. Let’s find the right stuff together for Kate so she can have fun with the other children."

NEXT

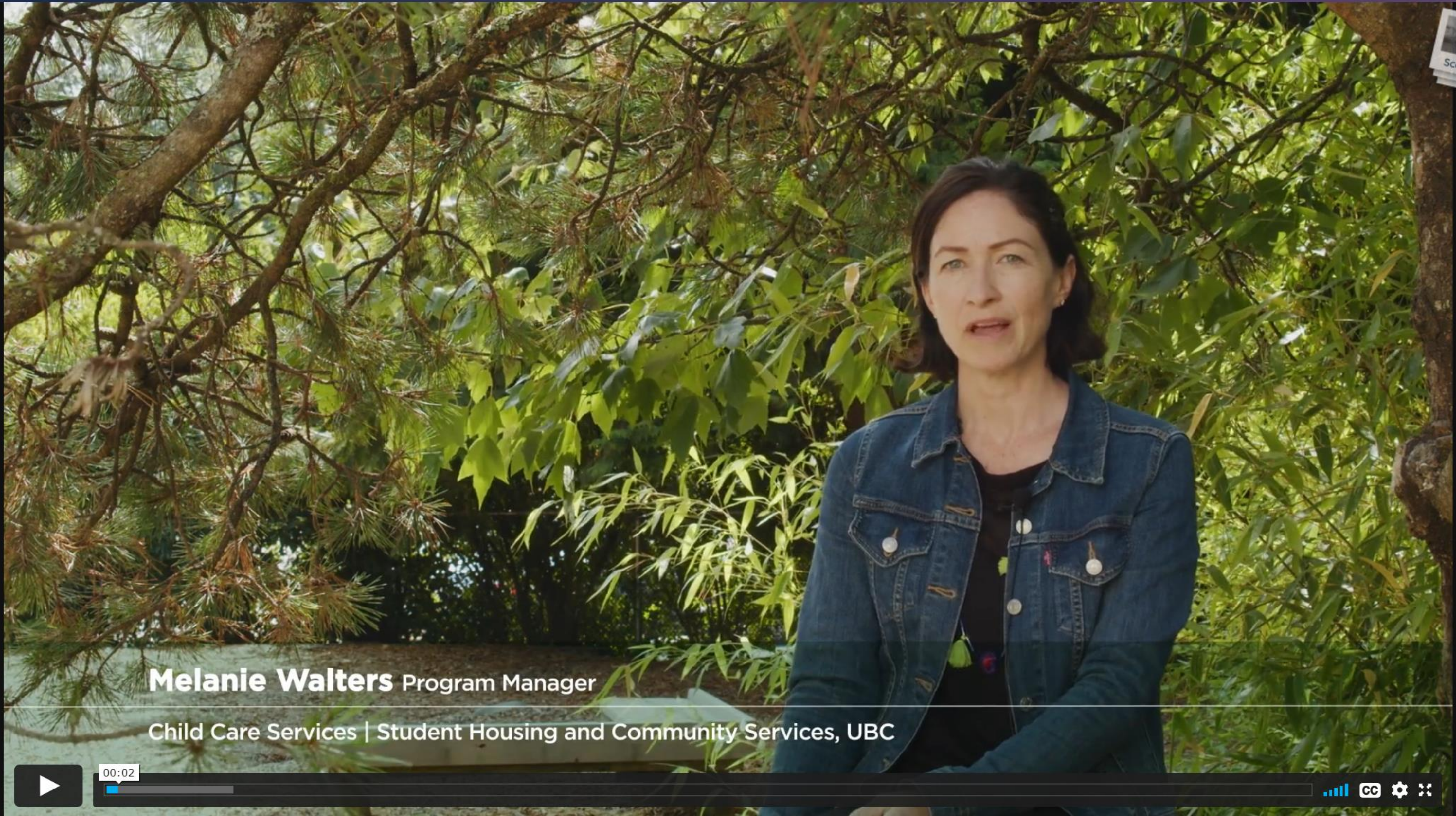
Scenarios
Melanie Walters Program Manager
Child Care Services | Student Housing and Community Services, UBC
00:02

What's the most important thing you learned?

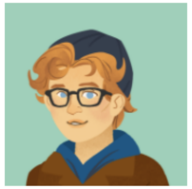

I learned...

Here are some examples if you get stuck

- Play outdoors opens up new possibilities that may not be available indoors and has unique benefits for children.
- High quality outdoor play can help children's learning, socio-emotional development, self-confidence, critical thinking skills, creativity, physical movement and many other benefits.
- Children need to be active for at least 180 minutes daily and outdoor play can help them meet this benchmark.
- People will have different comfort and familiarity with outdoor play. Introduce new ideas slowly and respectfully.
- Find repeated opportunities to connect with parents about outdoor play and how their child is benefiting.
- Pedagogical narration can be an important way to share learning with parents and children
- Outdoor play can occur in all weather, with the right clothing.
- Being outside in all kinds of weather helps build children's immune systems. It is harder to transmit some viruses outdoors.

Save and Continue

Chapter 2:

Scenarios

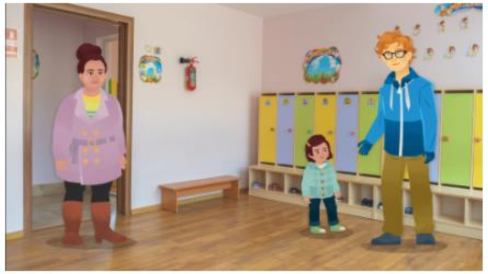
Communicating with Parents/Caregivers

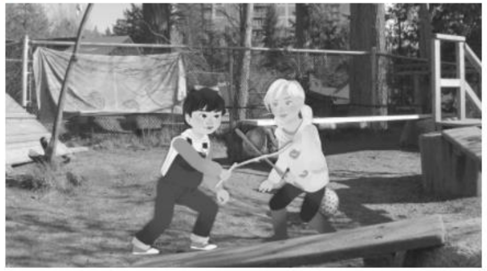
Rough and Tumble Play

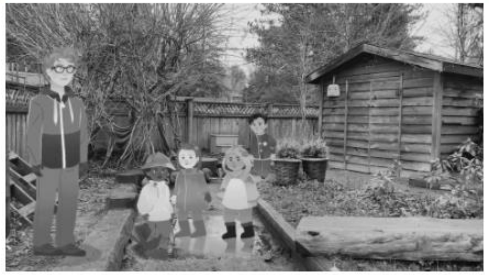
Play at Speed

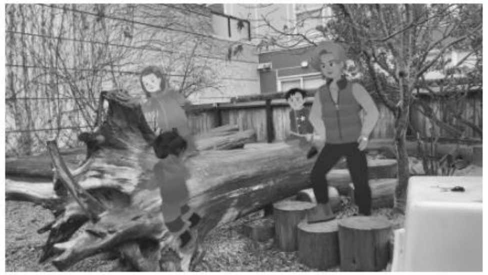
Play at Heights

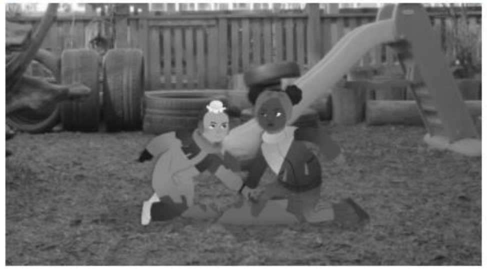
Conflict Resolution

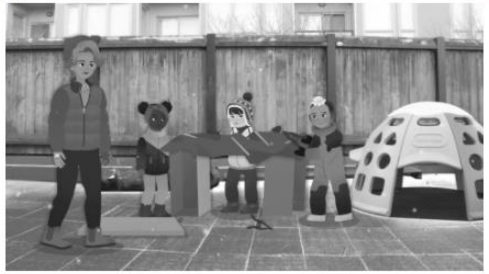
Play with Loose Parts

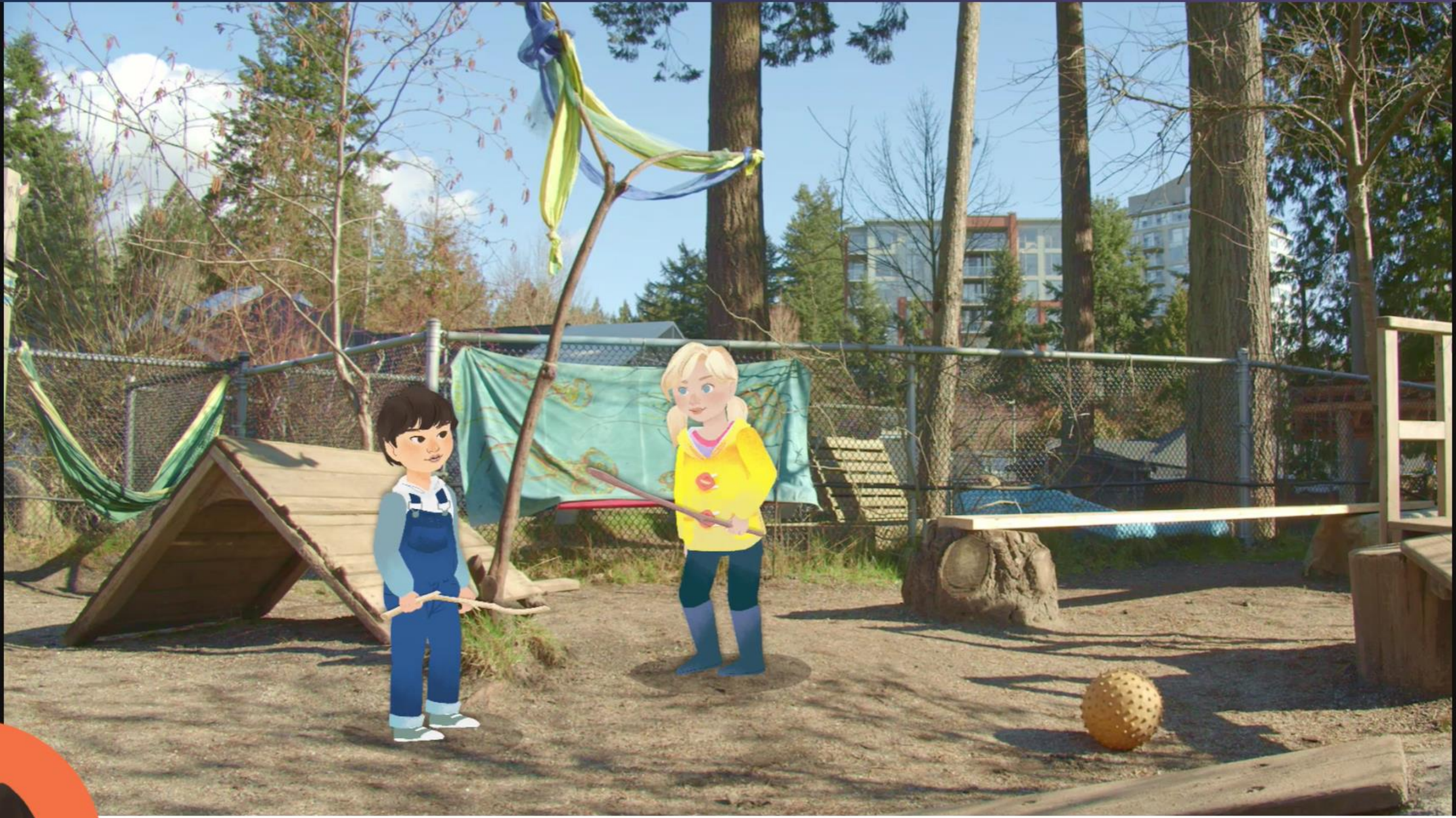

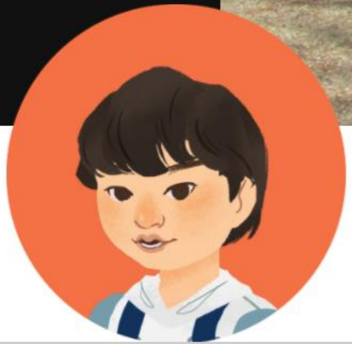

"Let's play swordfighting!"

NEXT

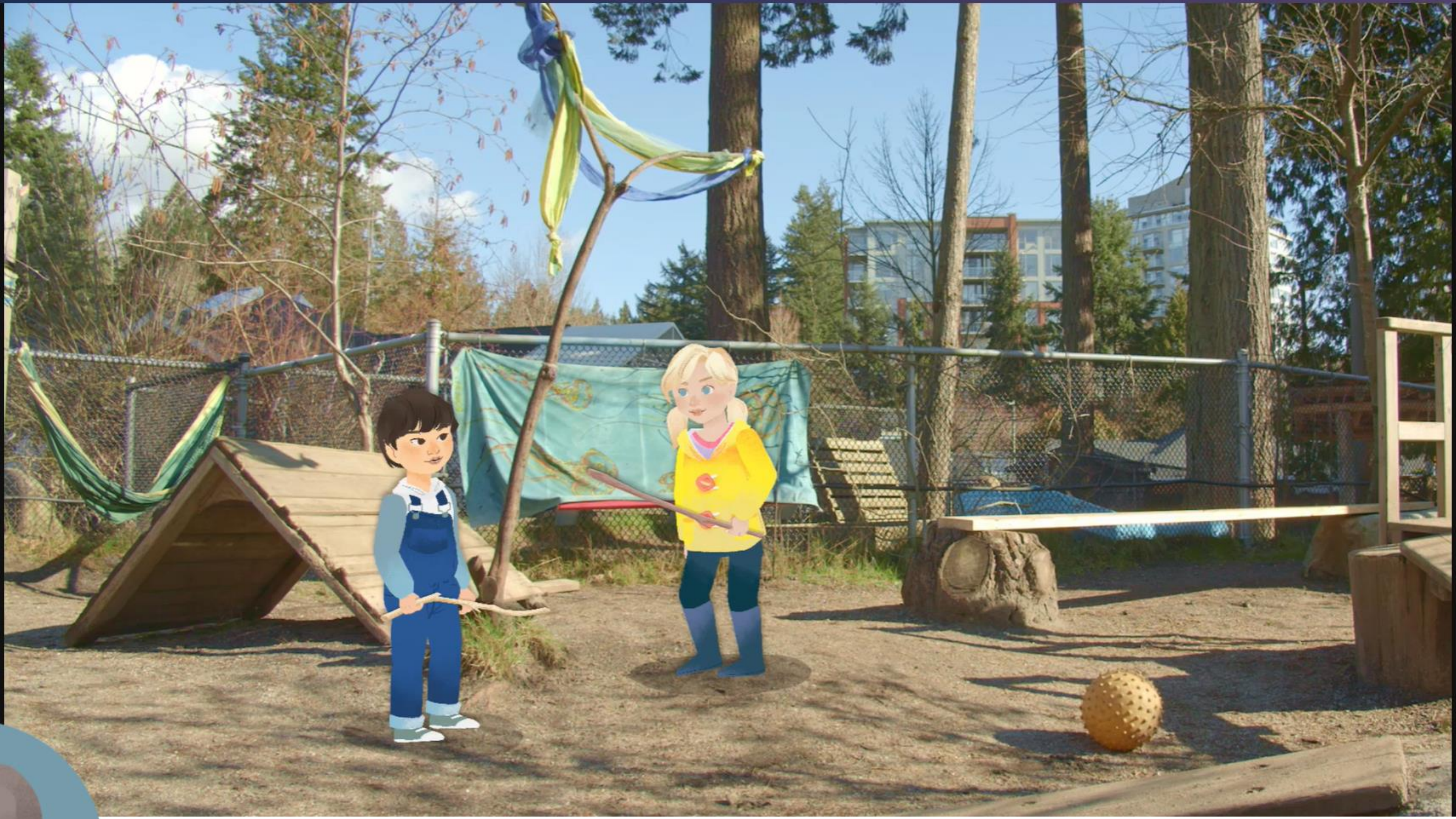

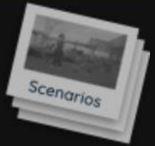

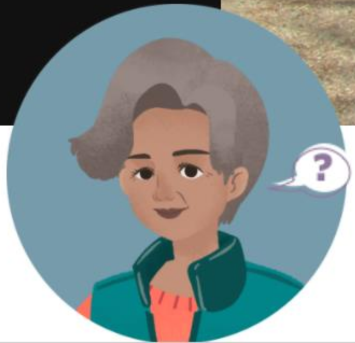

"What should I do?"

- Sticks are dangerous, stop the children!
- Talk to the children about consent and safety

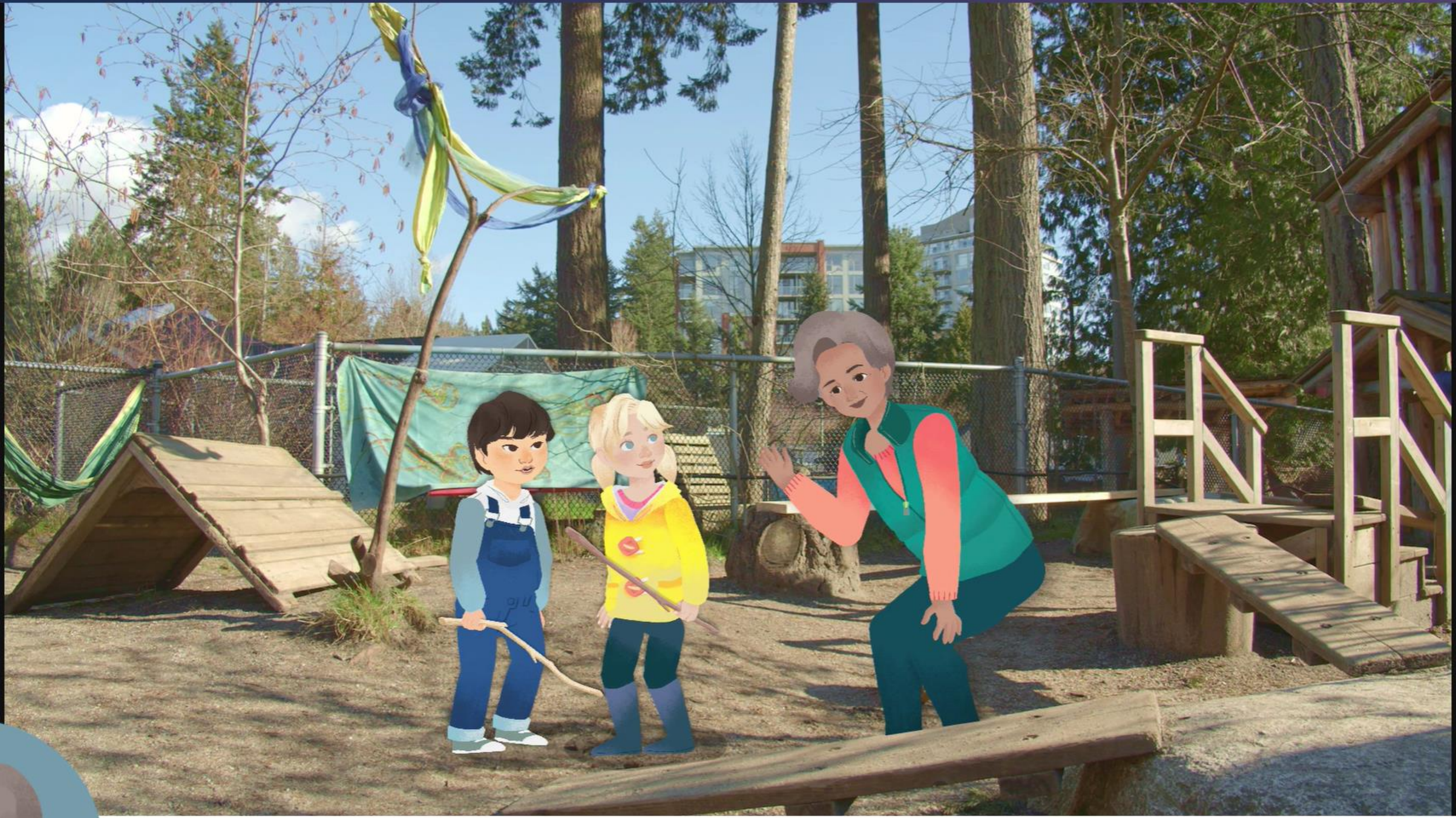

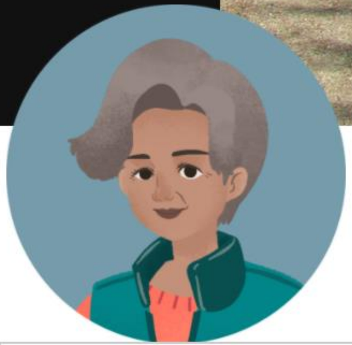

"This is not acceptable play. We don't play with sticks and weapons."

Scenarios

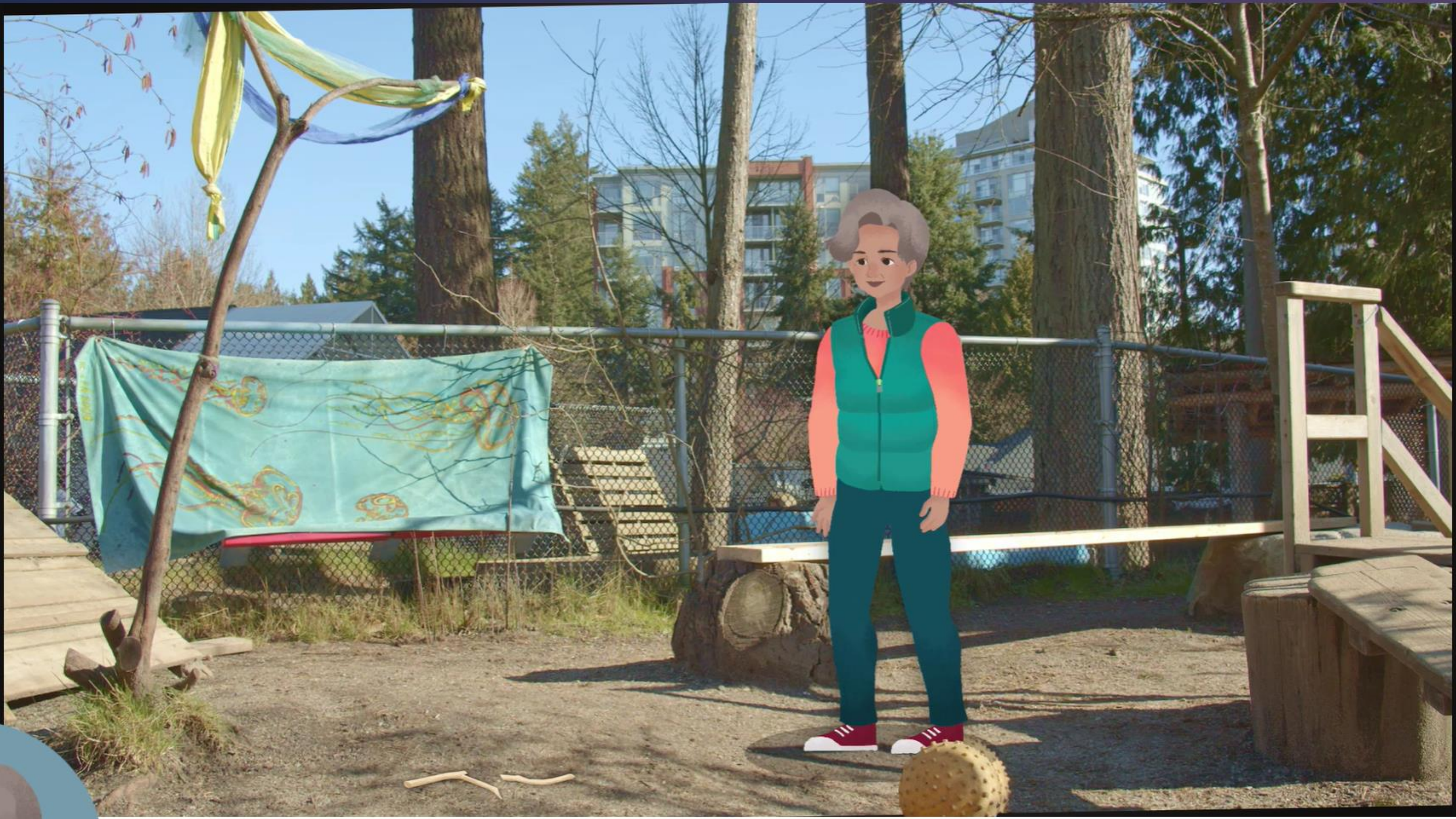

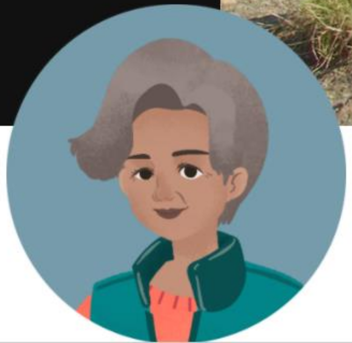

"Sticks can be dangerous!"

NEXT

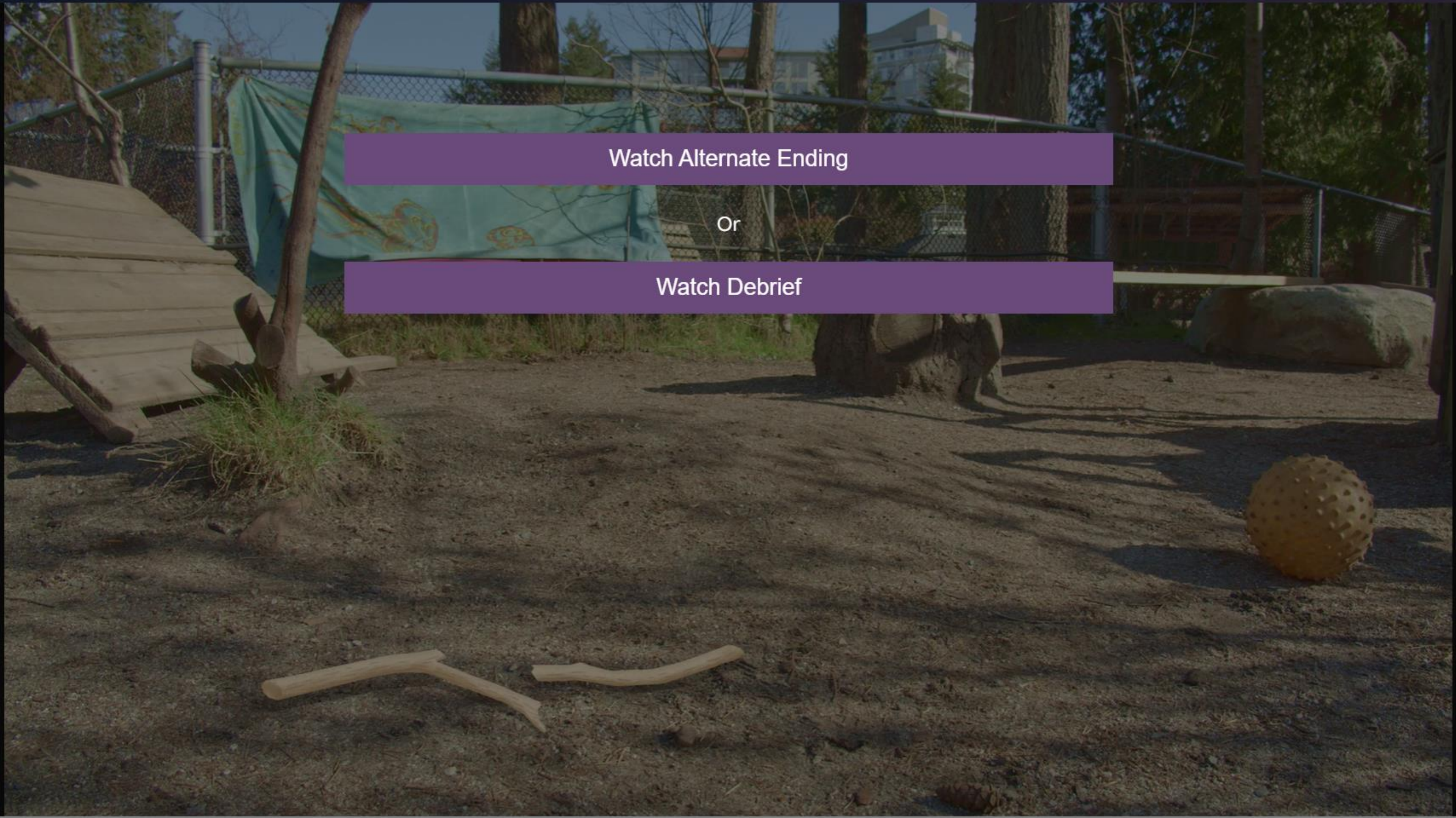

Watch Alternate Ending

Or

Watch Debrief

Scenarios

SKIP

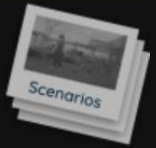

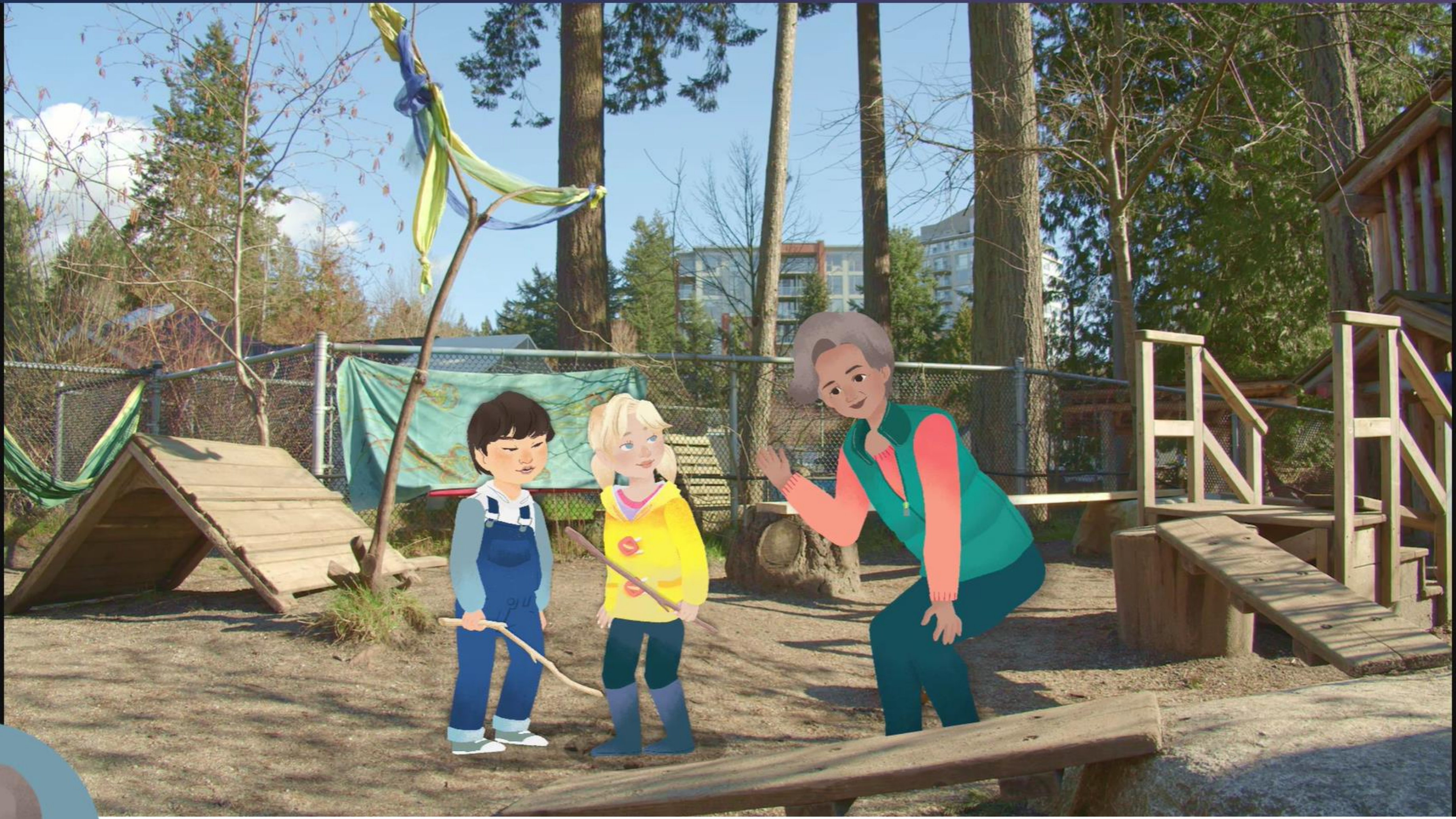

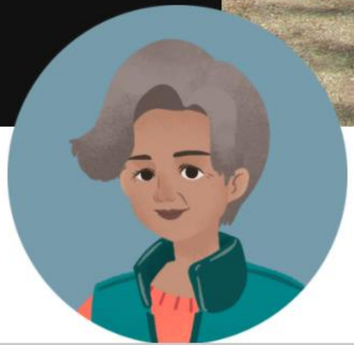

"When we play with sticks, we might get hit by sticks. Does everyone understand this and agree to be careful with each other's bodies?"

NEXT

Scenarios

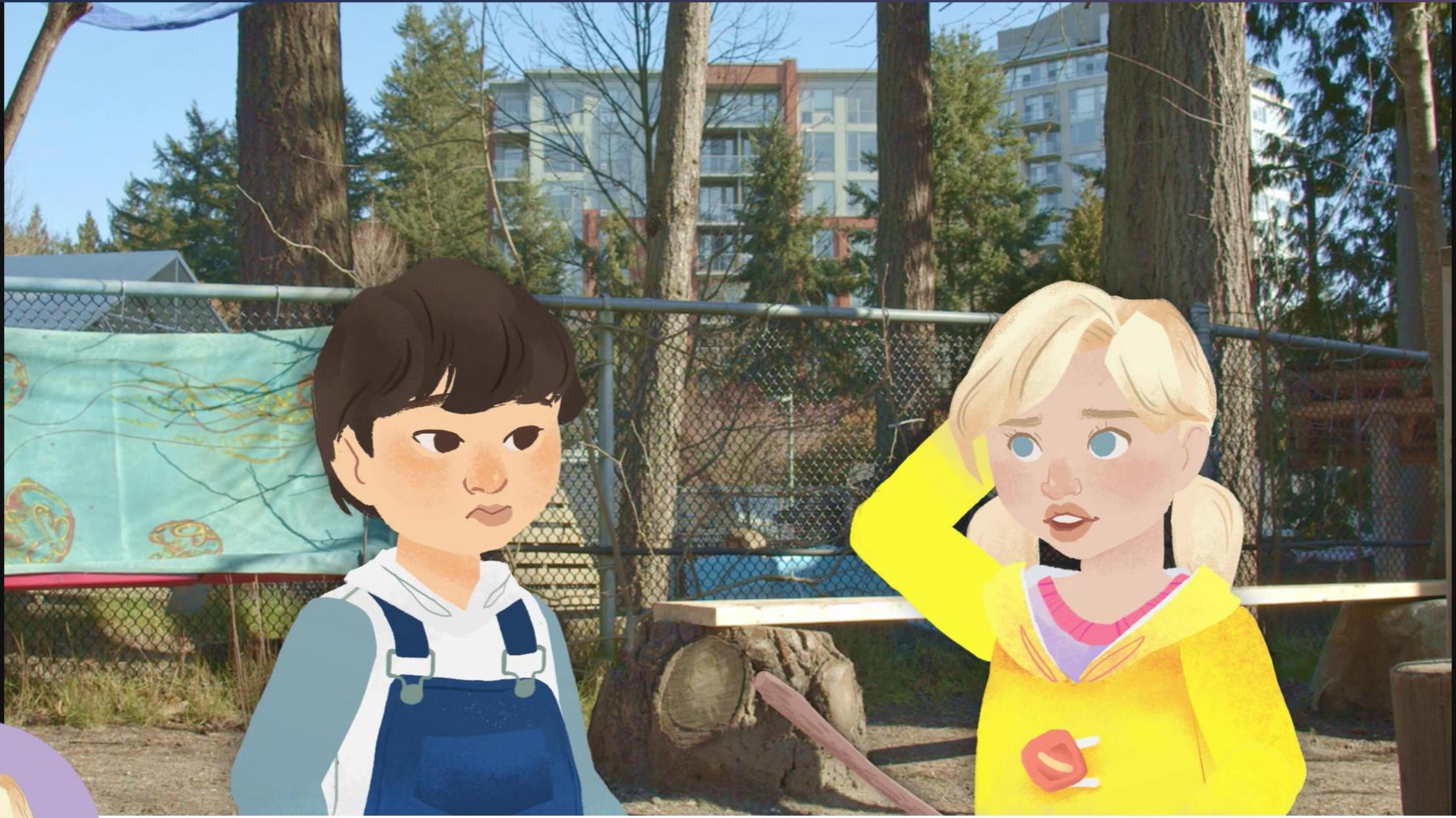

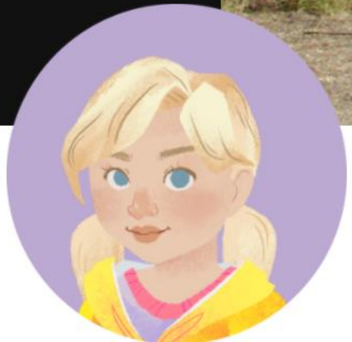

"Ow! That hurts!"

NEXT

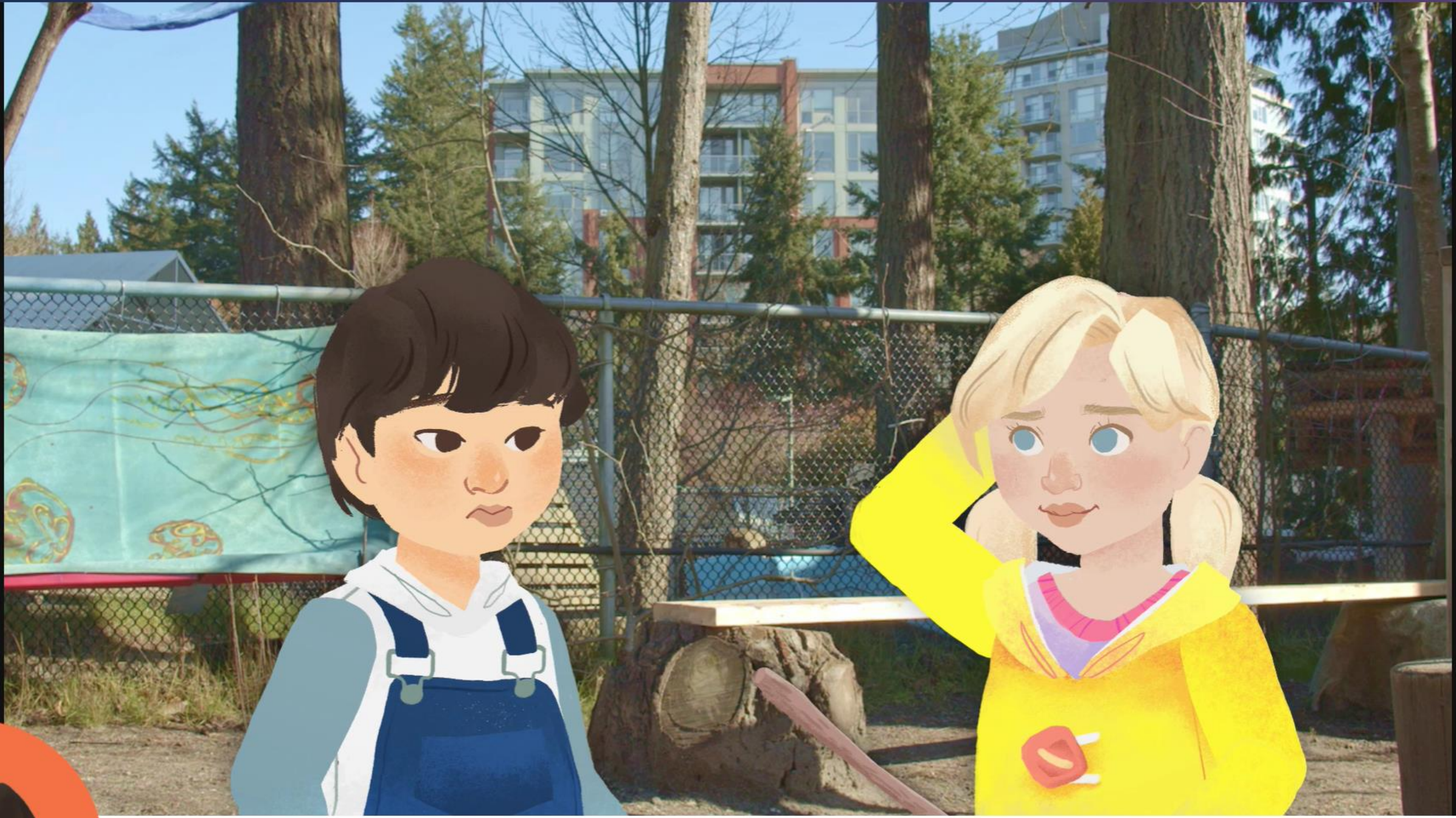

Scenarios

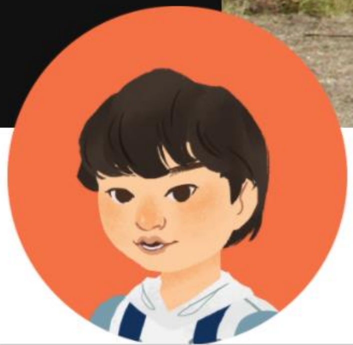

"Sorry..."

NEXT ▶

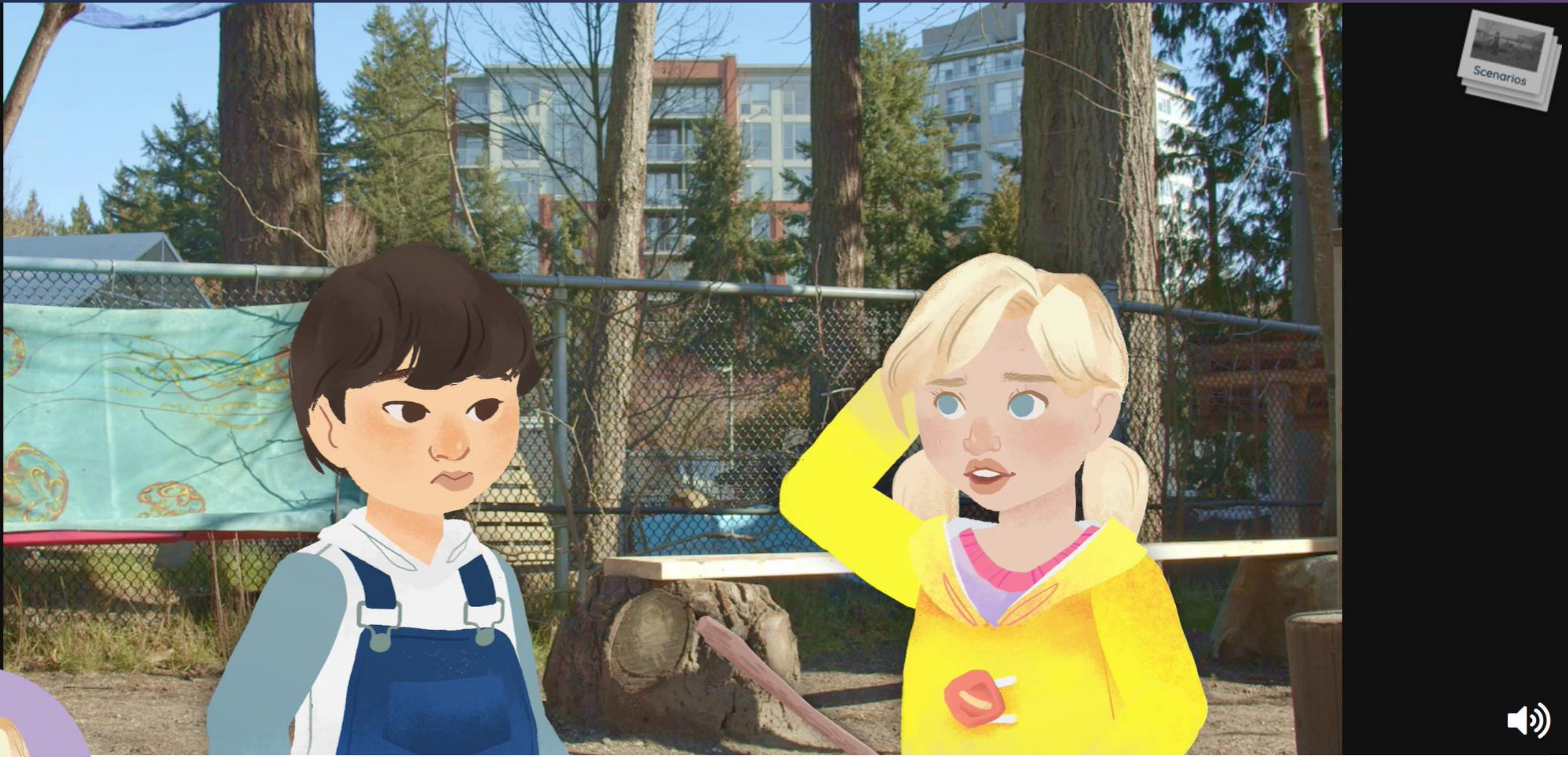

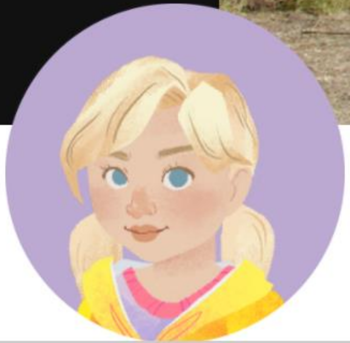

"Don't hit so hard next time."

NEXT

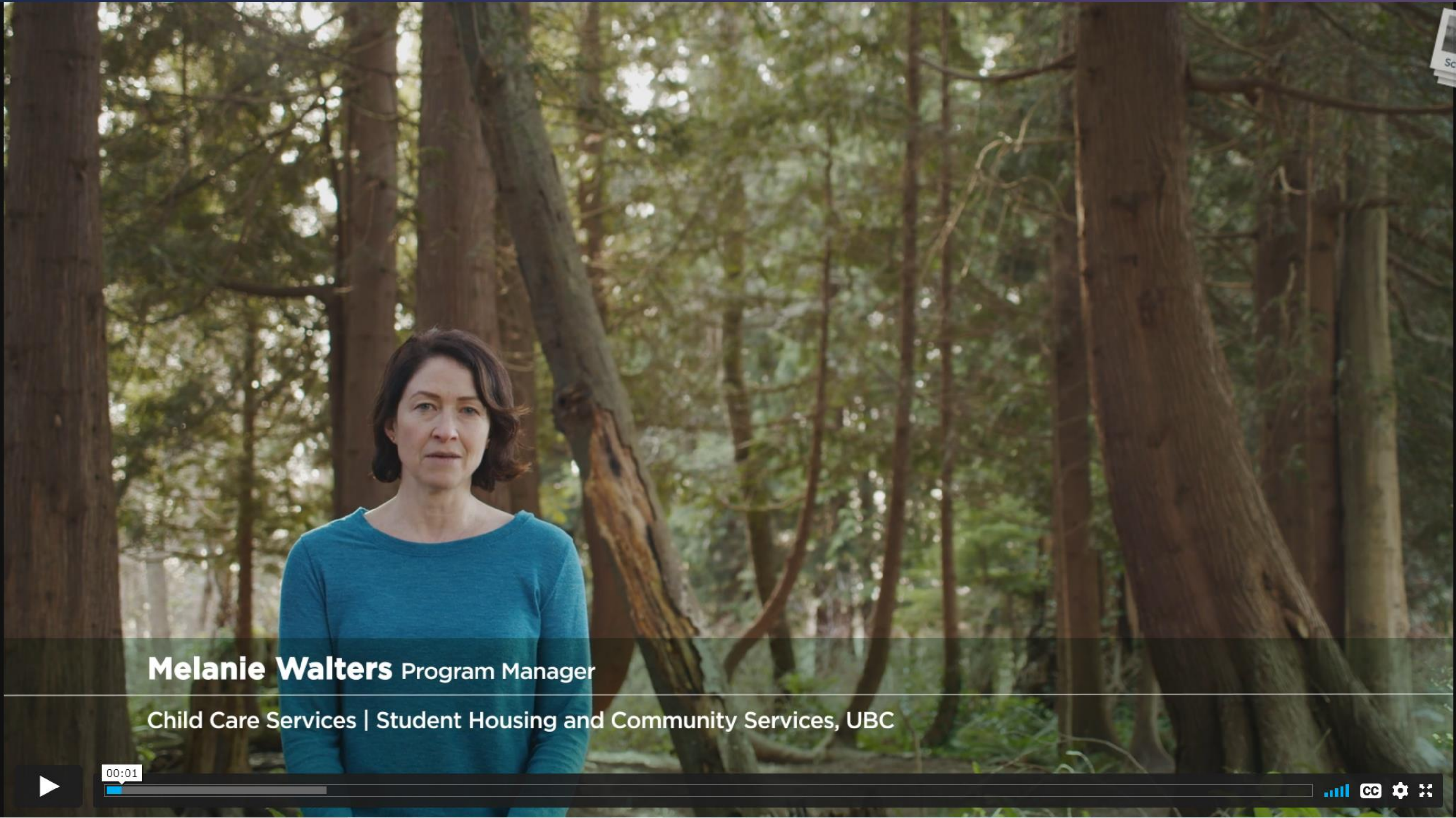
Scenarios
Melanie Walters Program Manager
Child Care Services | Student Housing and Community Services, UBC
00:01

What's the most important thing you learned?

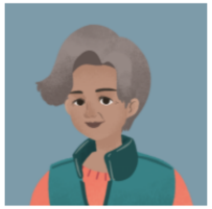

I learned...

Here are some examples if you get stuck

- There is a difference between hurting another person out of anger and willingly participating in a game of rough-and-tumble play.
- Rough-and-tumble play can teach children movement skills, build their confidence, social rules, and perspective-taking.
- Rough-and-tumble play can be an opportunity to learn about consent.
- Rough-and-tumble play can be an opportunity to learn about risk management.

Save and Continue

Chapter 2:
Scenarios

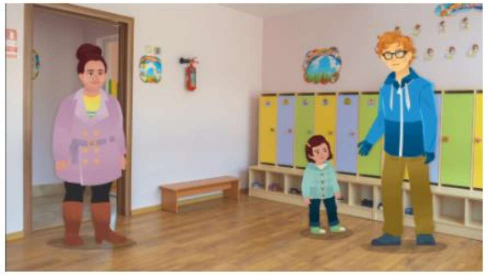

Communicating with Parents/Caregivers

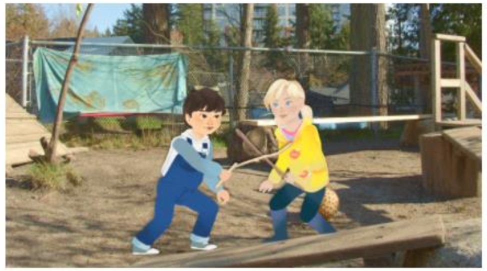

Rough and Tumble Play

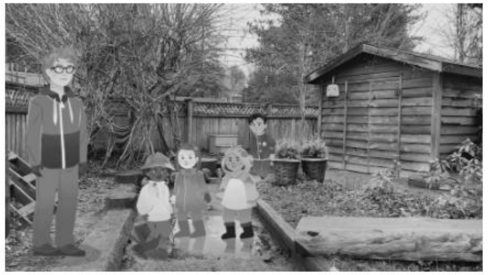

Play at Speed

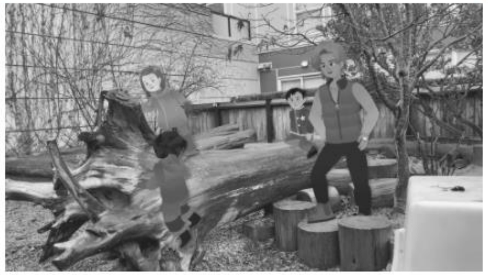

Play at Heights

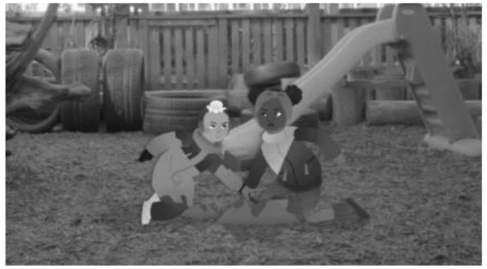

Conflict Resolution

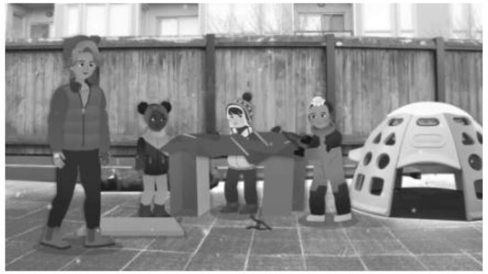

Play with Loose Parts

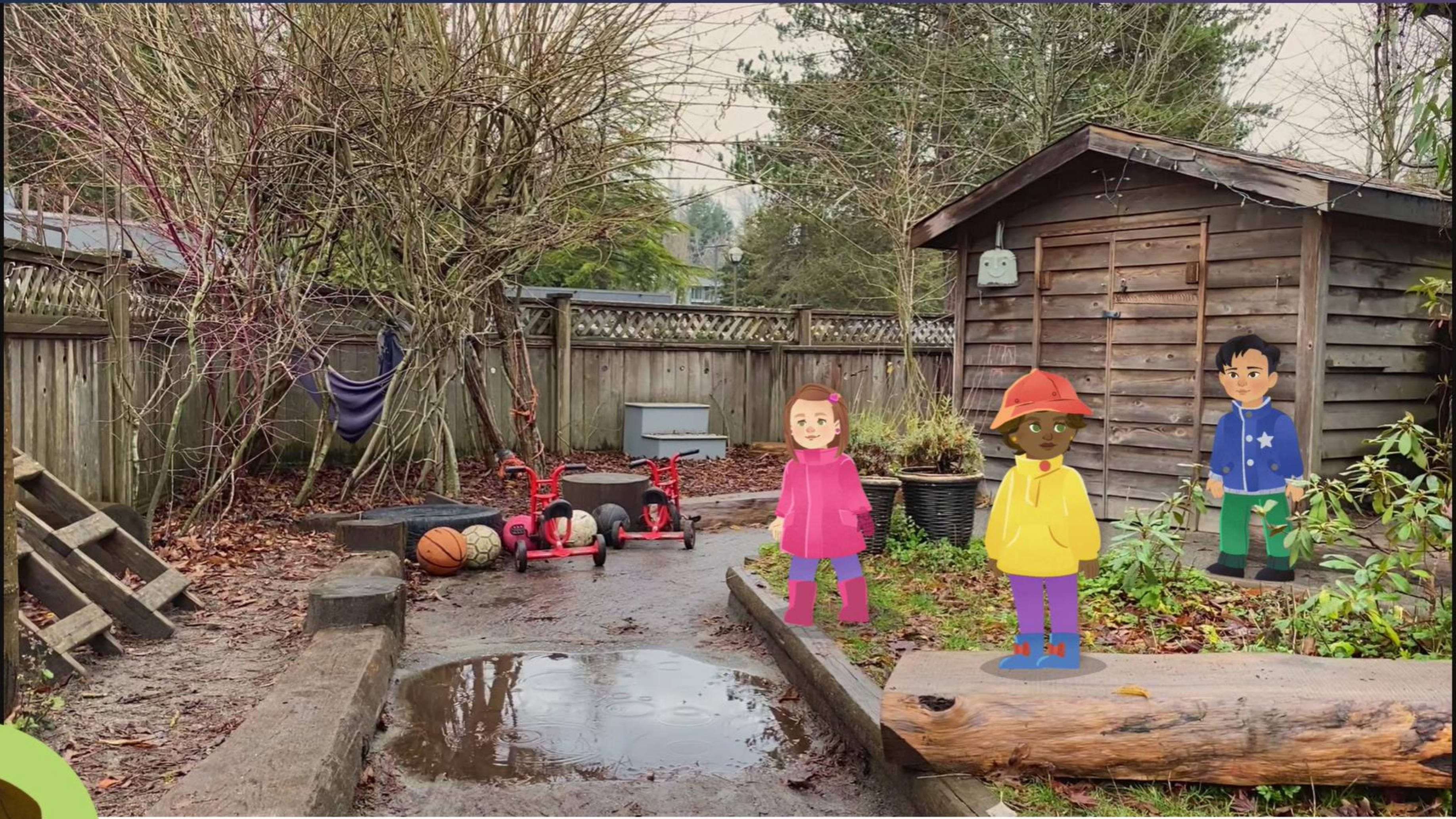

Scenarios

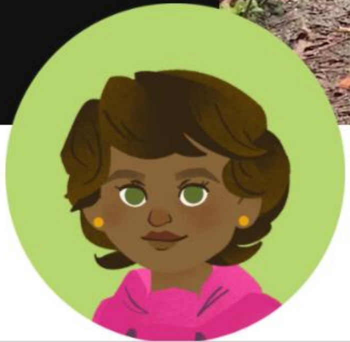

"Let's play tag!"

NEXT

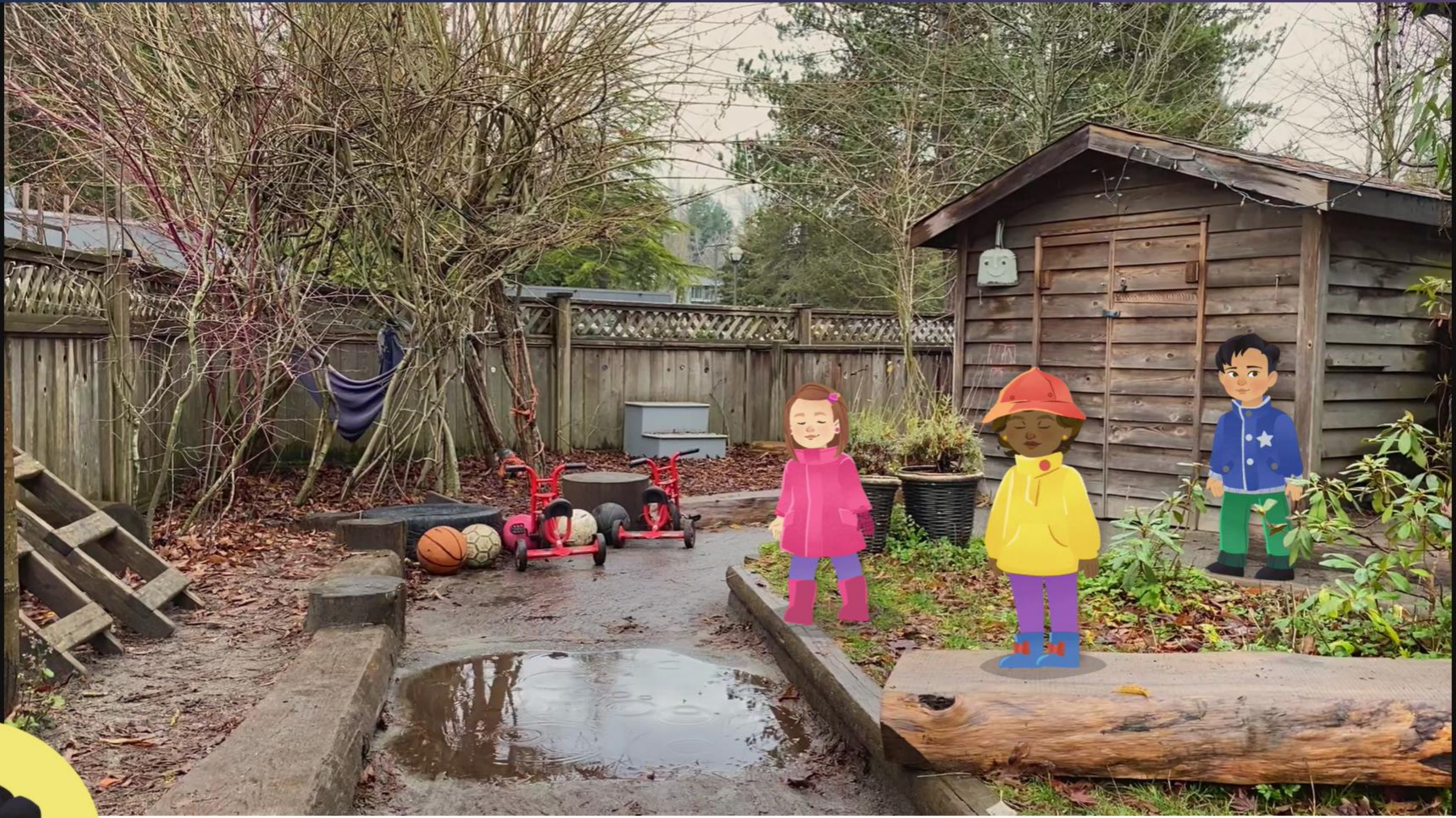

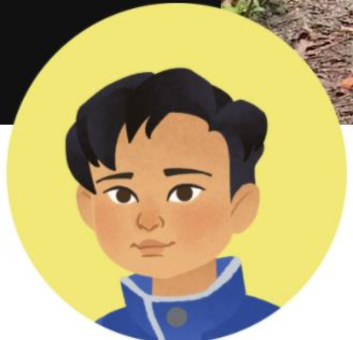

"Carlos, you're it!"

NEXT

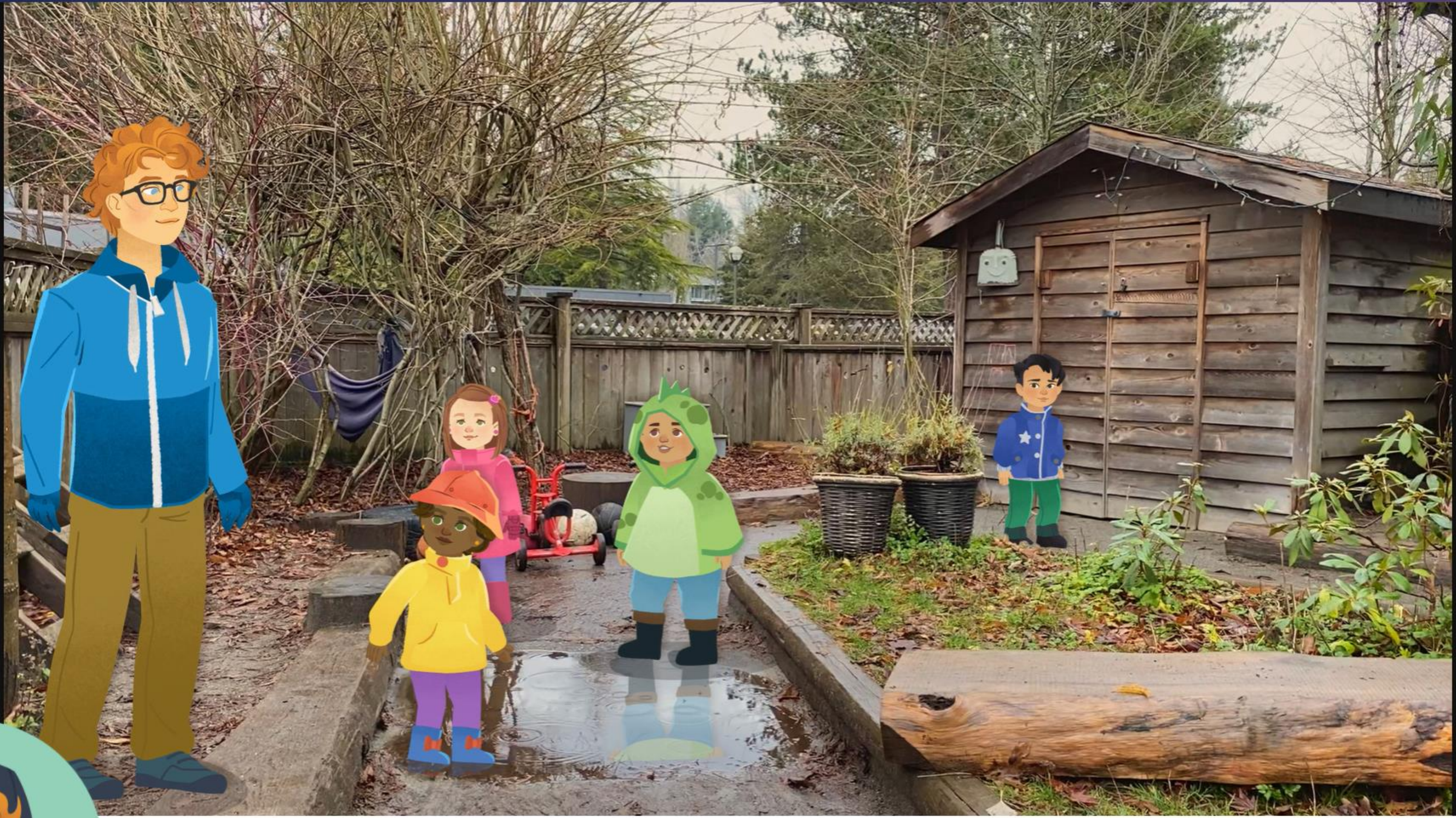

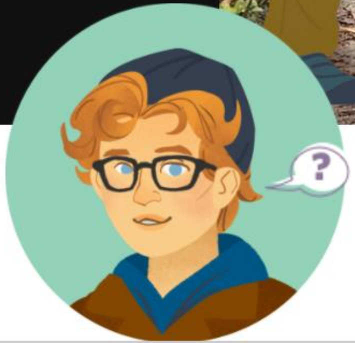

"What should I do?"

- It's wet and muddy, let's play indoors.
- Let the children keep playing

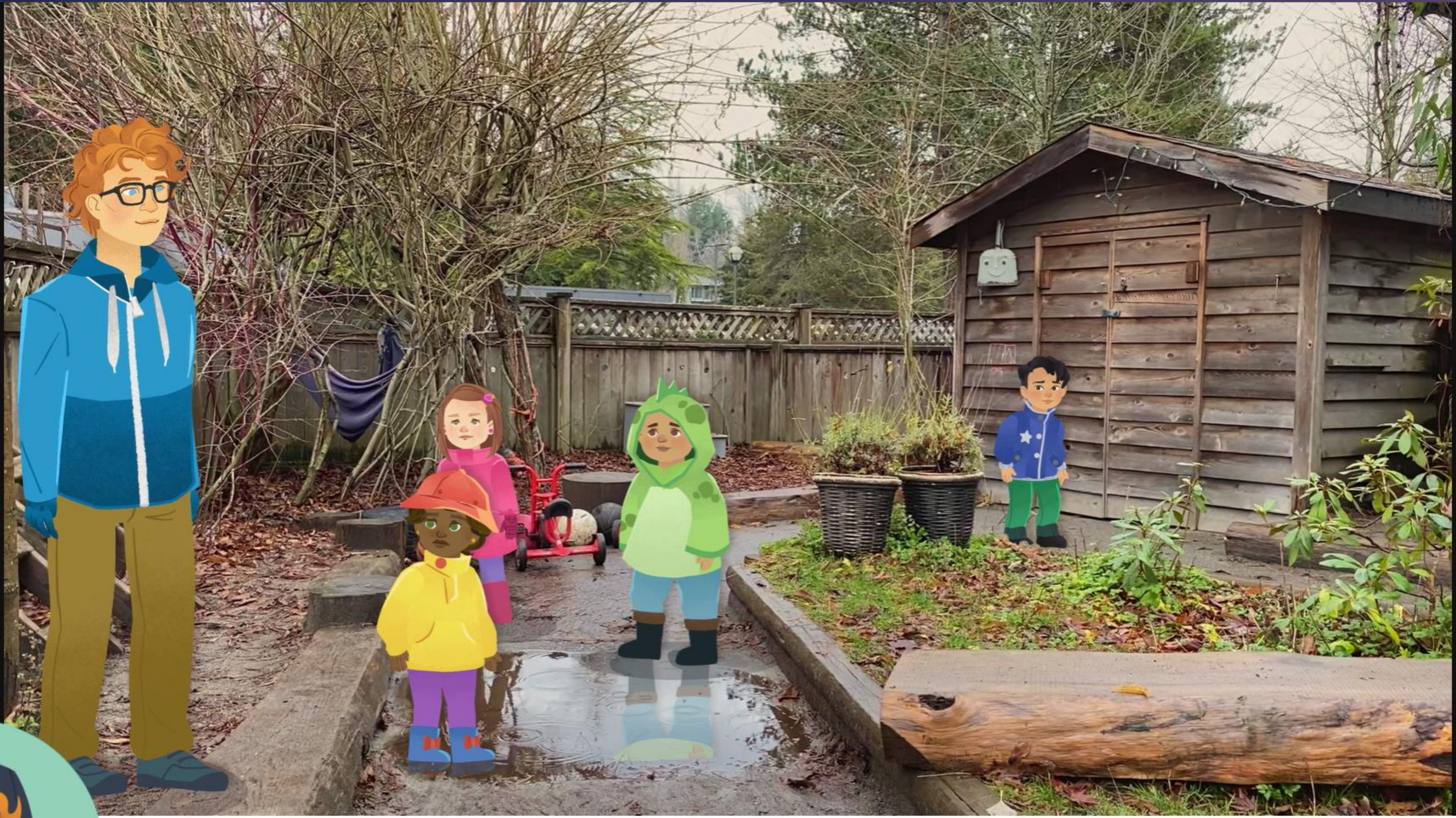

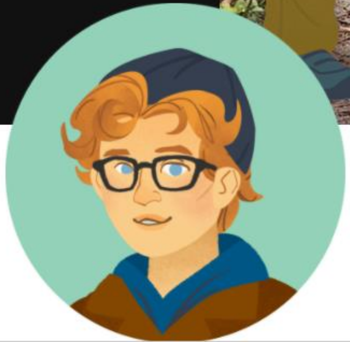

"Be careful!"

NEXT

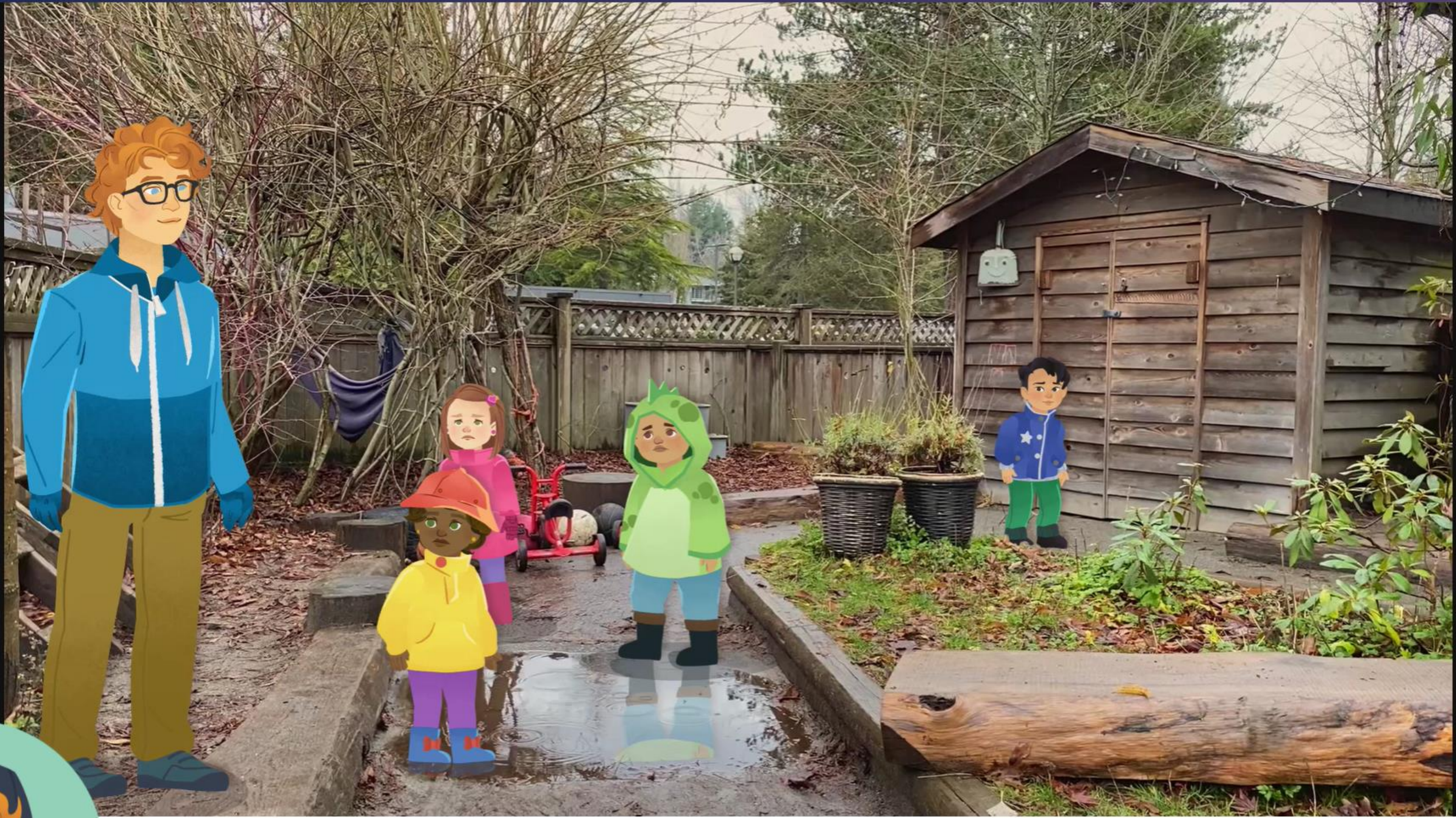

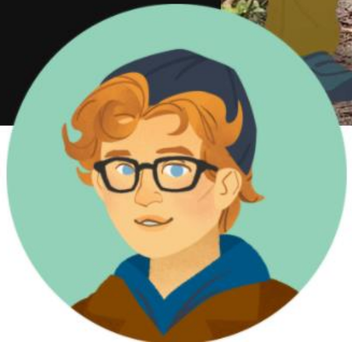

"The ground is muddy and slippery, so why don't we play tag some other time"

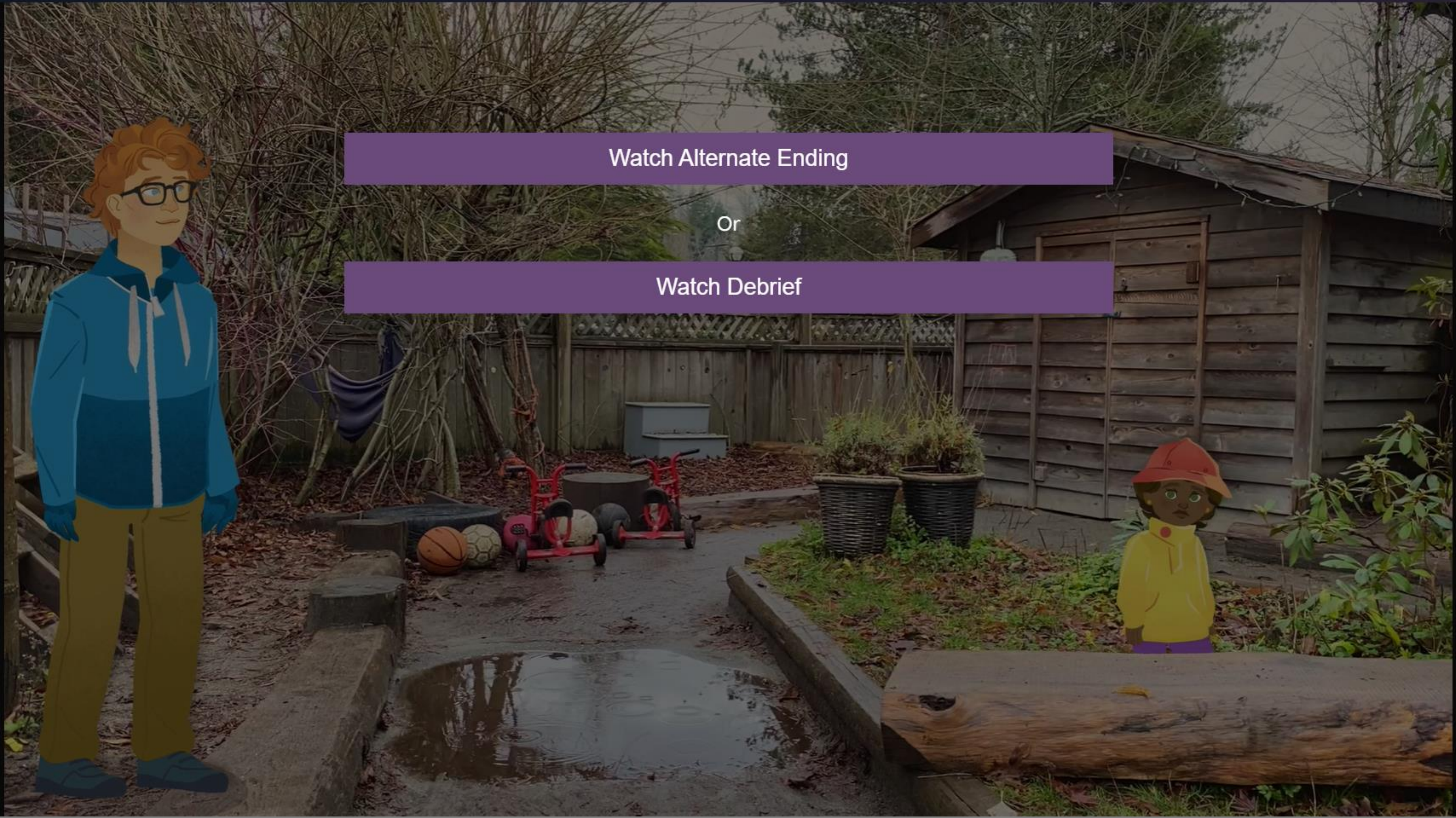

Scenarios

SKIP

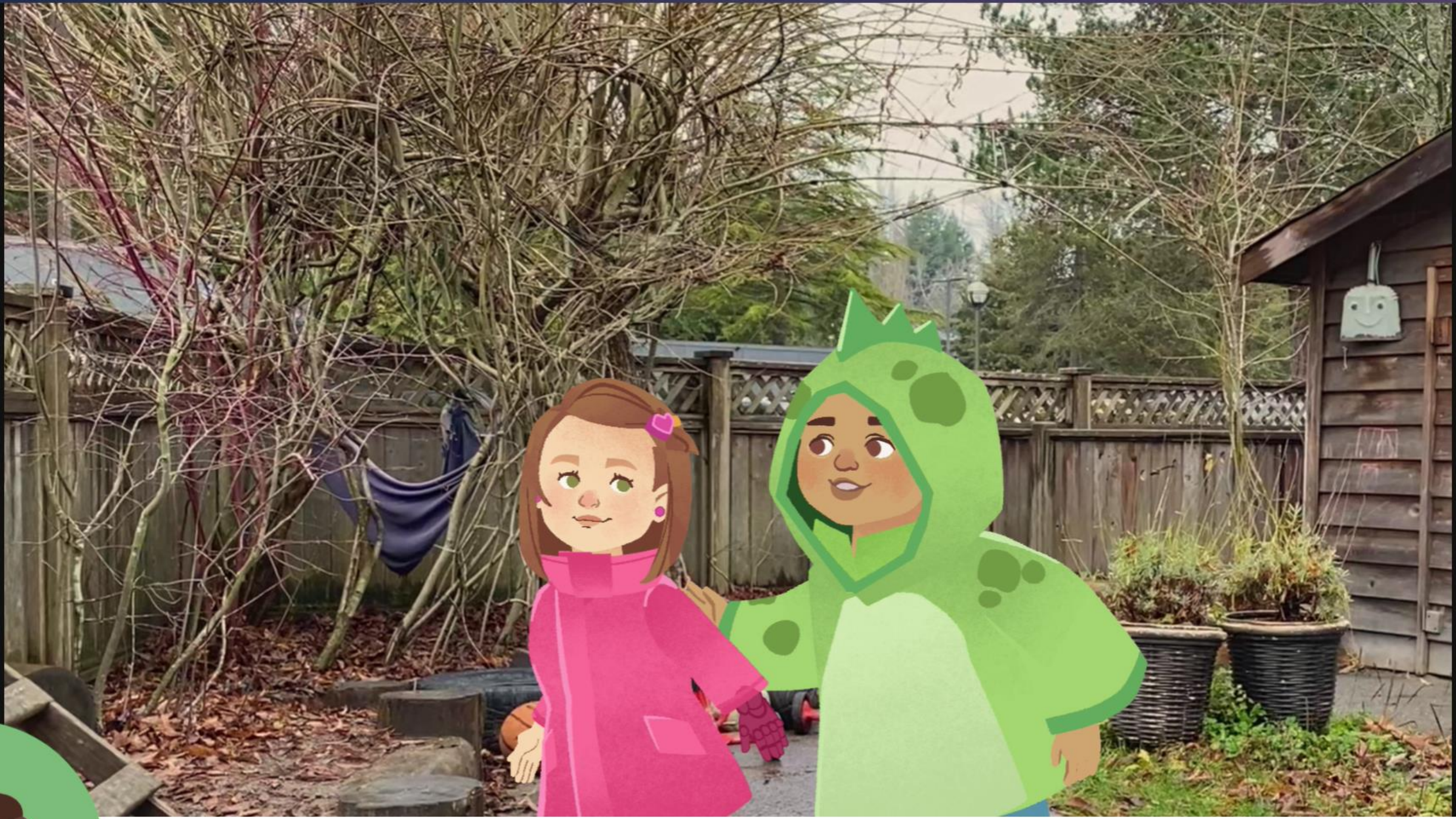

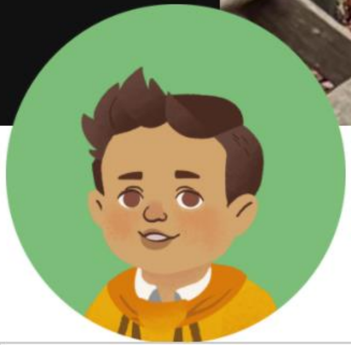

"Got you! Now you're it!"

NEXT

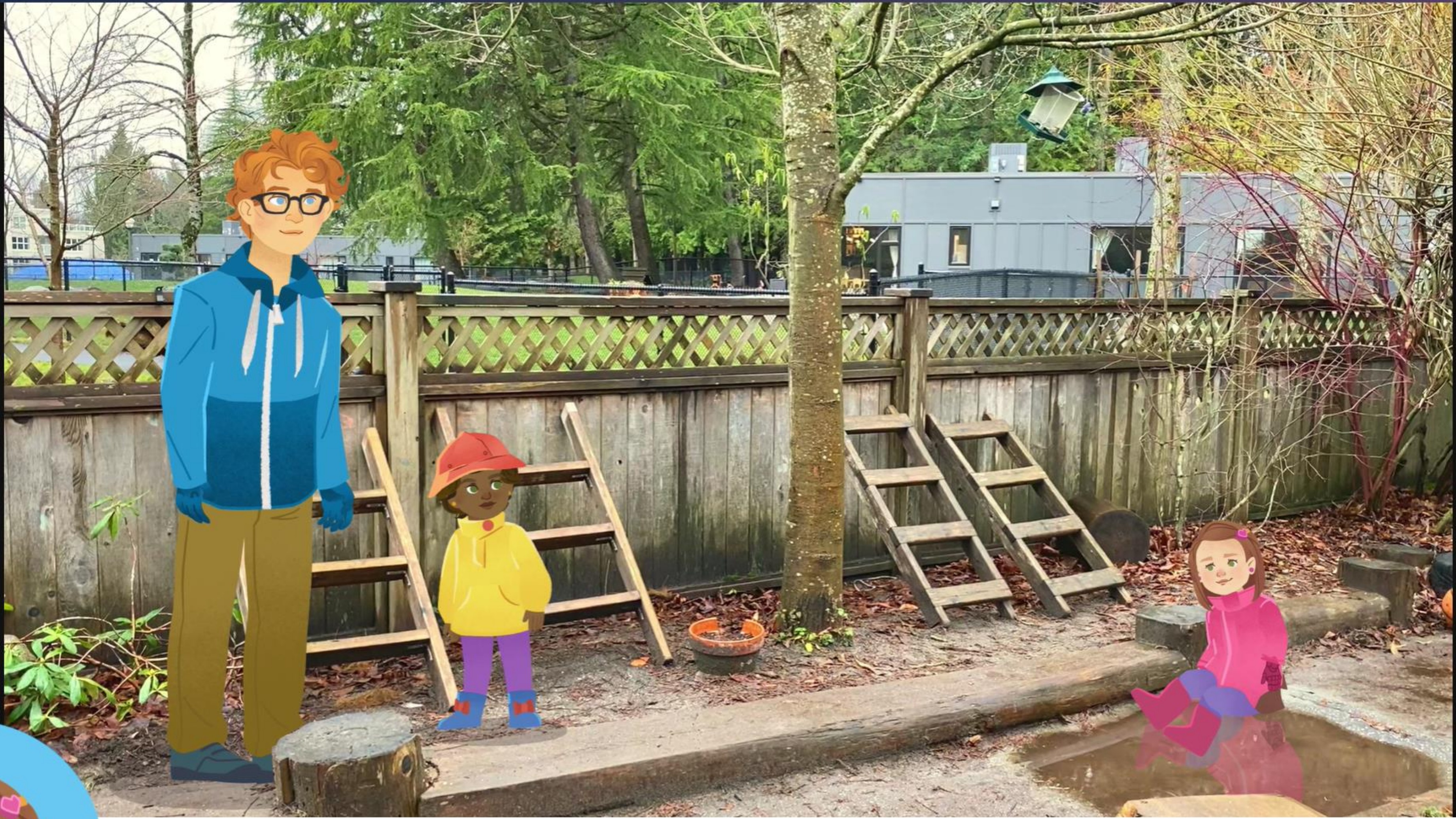

Scenarios

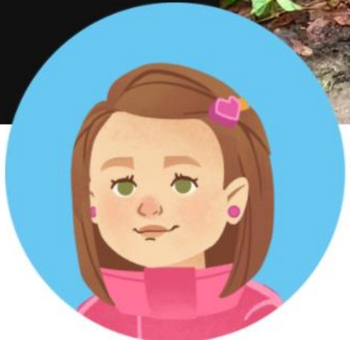

"Look at me! I'm all muddy!"

NEXT

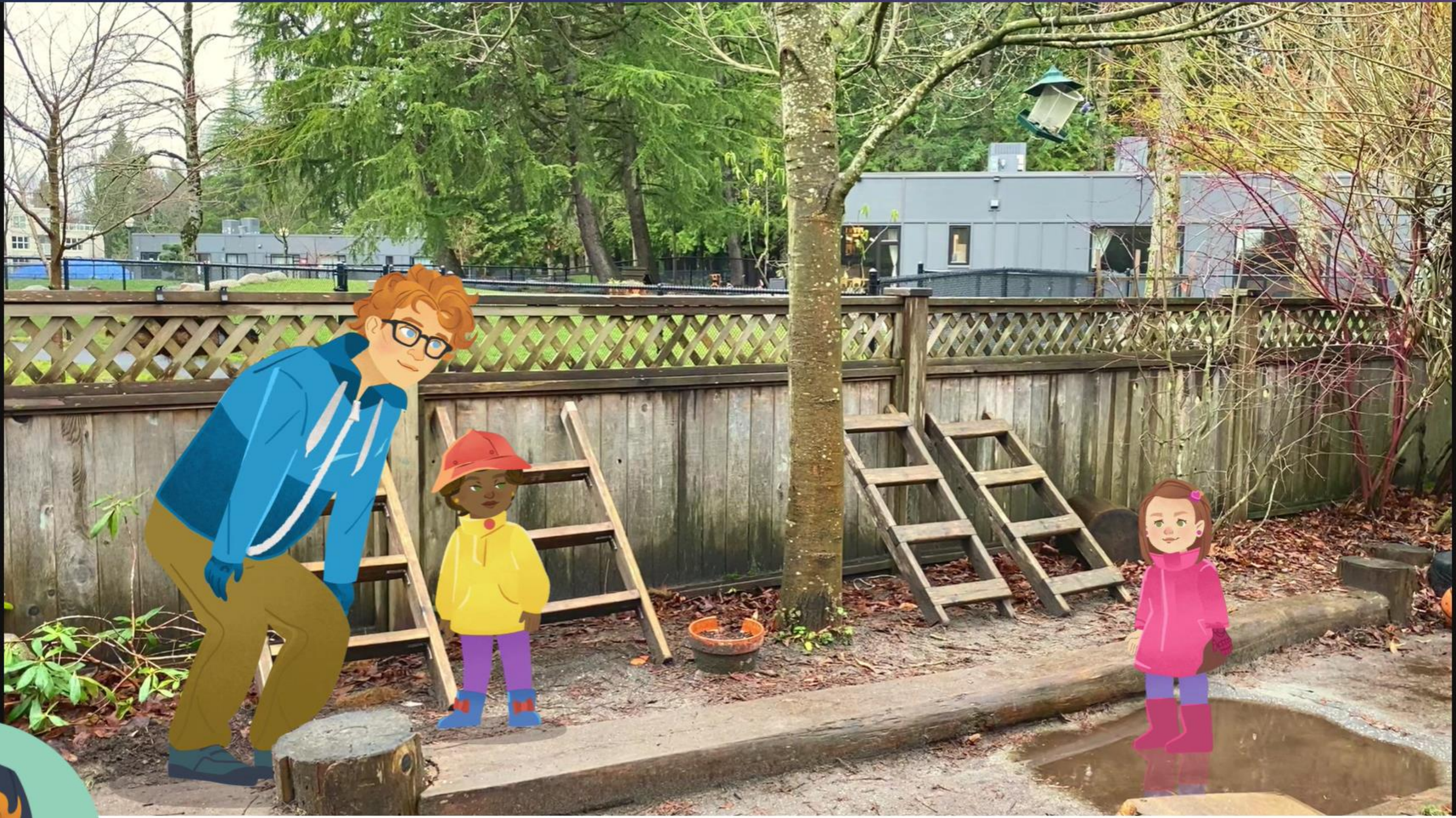

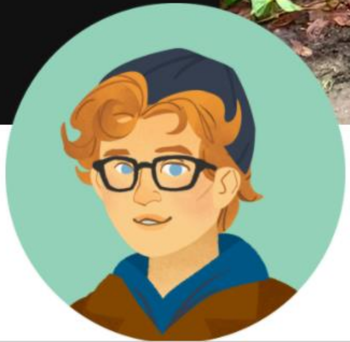

"That looks like so much fun!"

NEXT

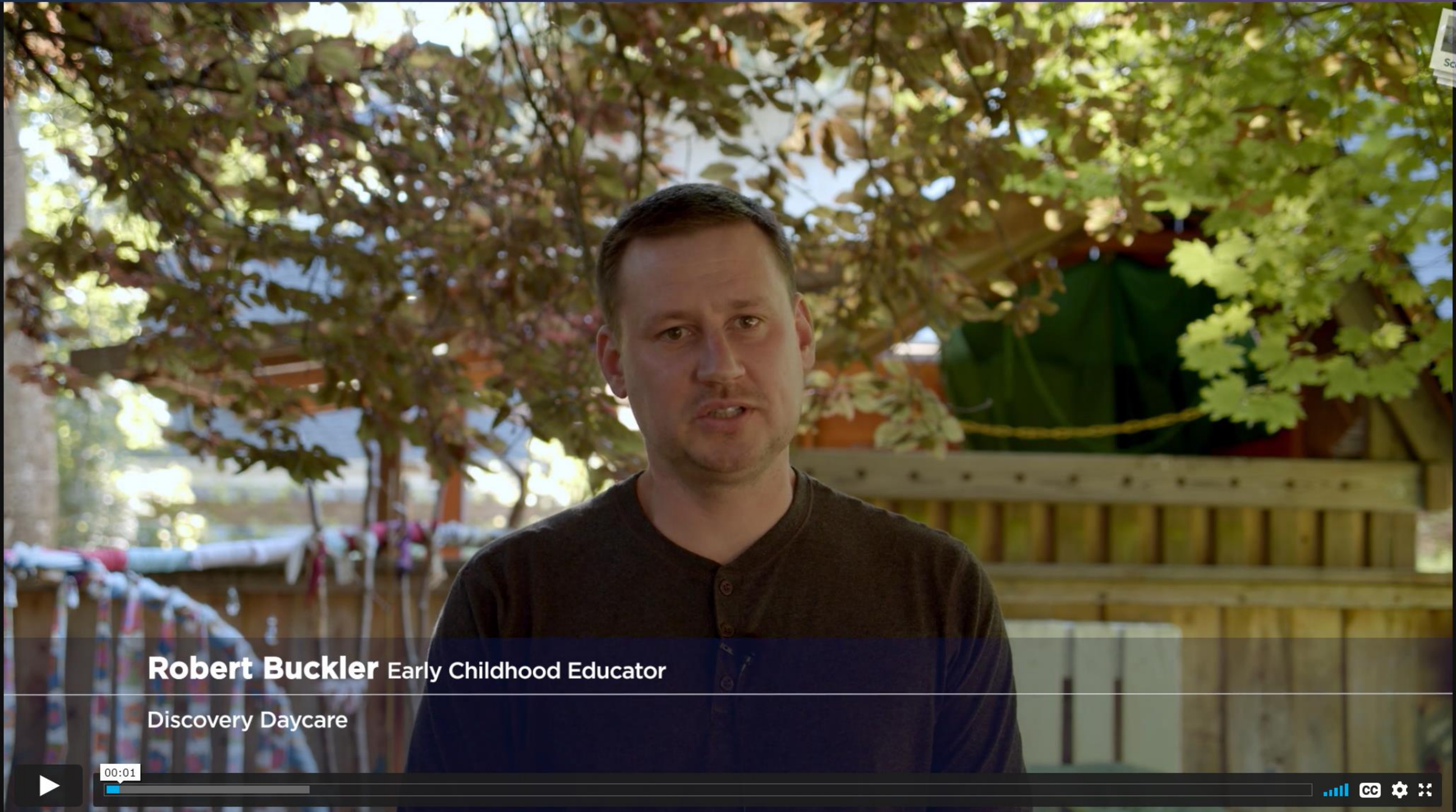
Scenarios
Robert Buckler Early Childhood Educator
Discovery Daycare
00:01

What's the most important thing you learned?

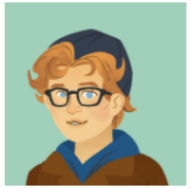

I learned...

Here are some examples if you get stuck

- The limits I put on children's play can be arbitrary and reflect my own biases.
- The likelihood of a serious injury resulting from children's risky play is extremely low.
- Loose parts, like mud, water, sticks, can provide wonderful play value and bring joy.
- Experiencing how to manage running and playing in different environments and under different conditions helps children learn how to move their bodies and develop risk management skills that they can use in other situations.
- When I say "be careful" I am saying "I am afraid for you and uncomfortable with what you are doing." The child hears "I don't trust you to keep yourself safe."
- Breathing deeply for several breaths can help me calm my anxiety and give some time for me to see if the children actually need or ask for my help.
- Children with exceptionalities also need to be able to choose their play and engage in risk taking behaviour.
- My familiarity and relationship with the children can help me learn the capabilities of each child, how much support I need to provide and help build trust in their abilities to keep themselves safe.

Save and Continue

Chapter 2:

Scenarios

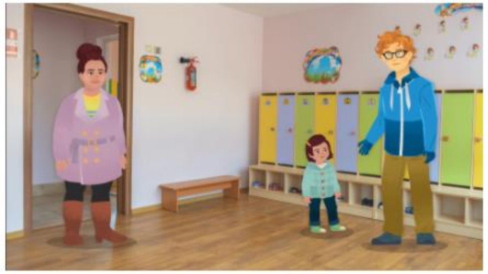

Communicating with Parents/Caregivers

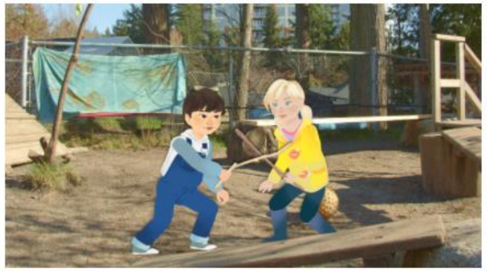

Rough and Tumble Play

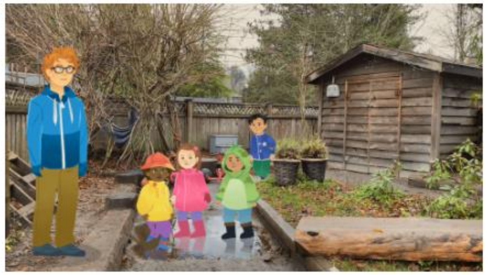

Play at Speed

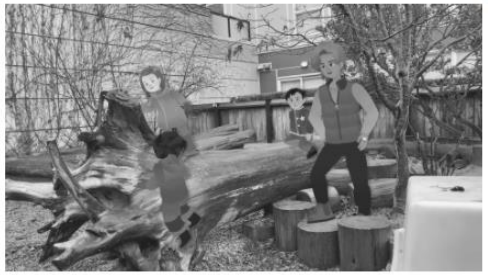

Play at Heights

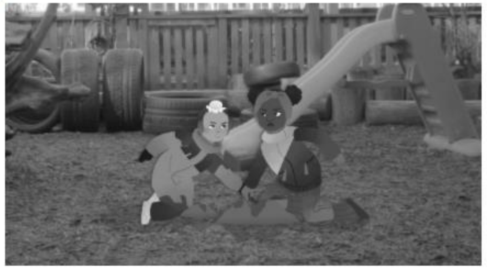

Conflict Resolution

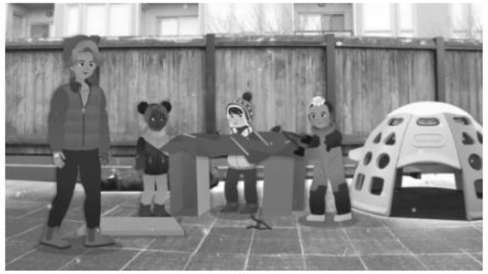

Play with Loose Parts

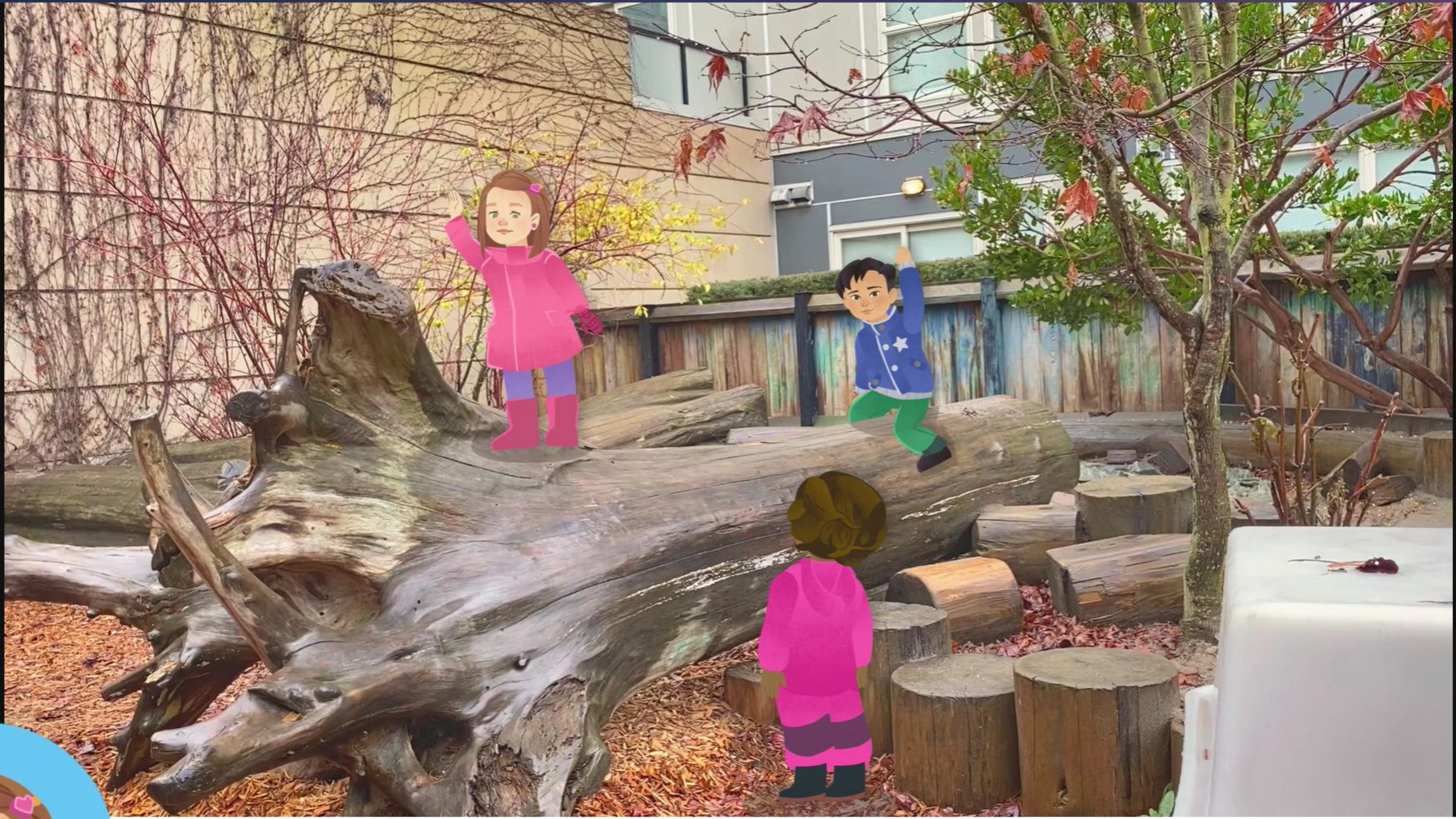

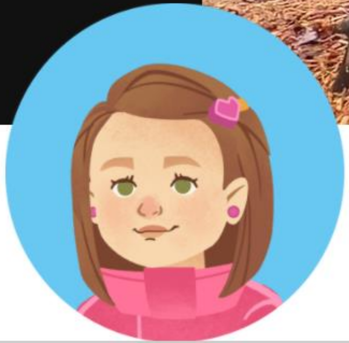

"Hey Jasmine! Do you want to come play up here too? It's fun!"

NEXT ▶

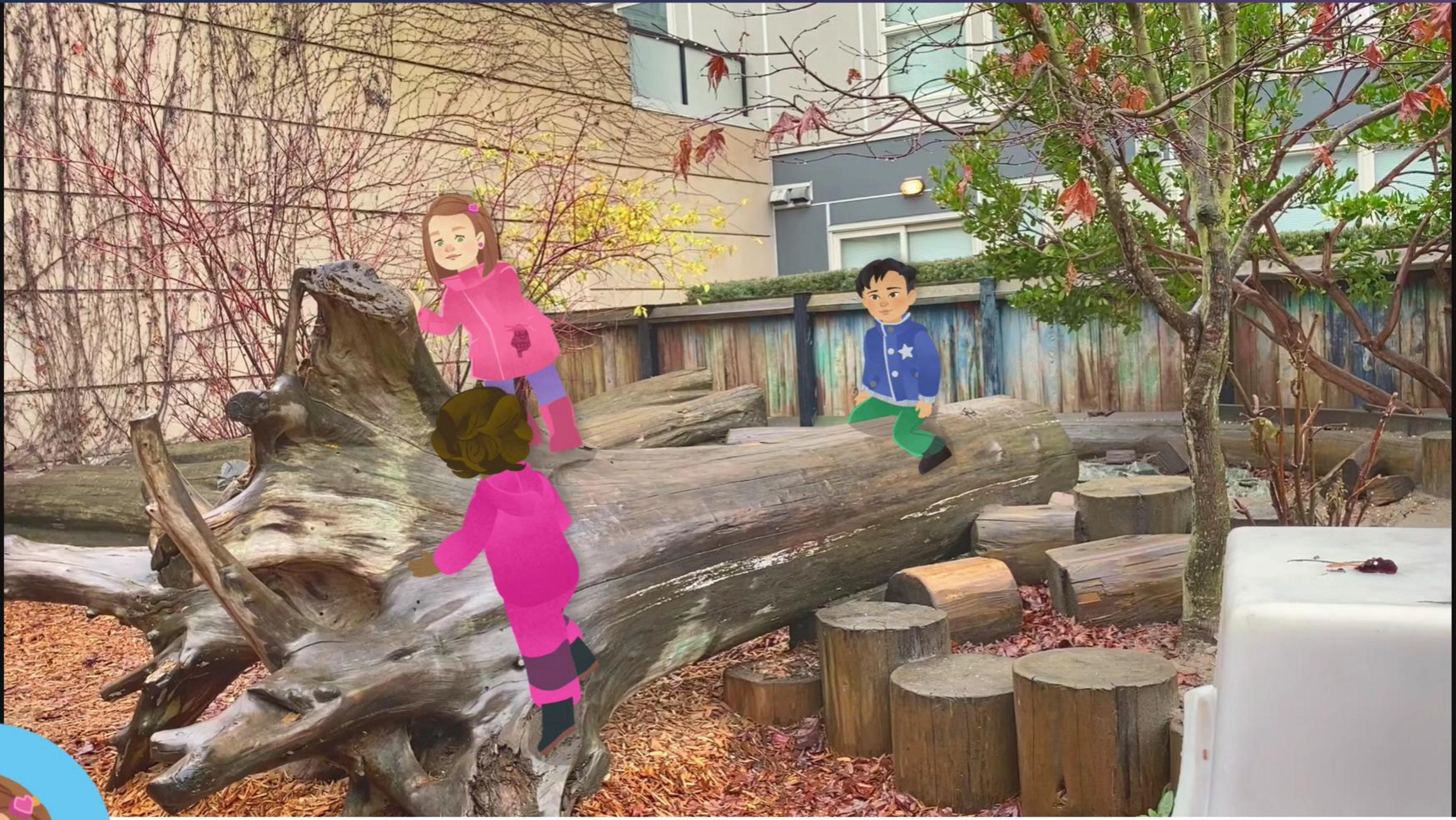

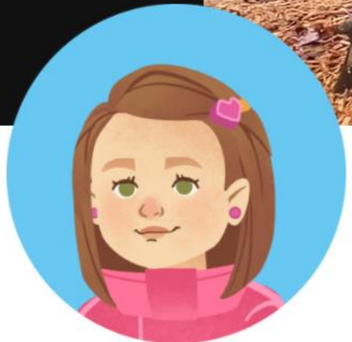

"There's a good place to grab with your hands here, and you can put your feet there."

NEXT

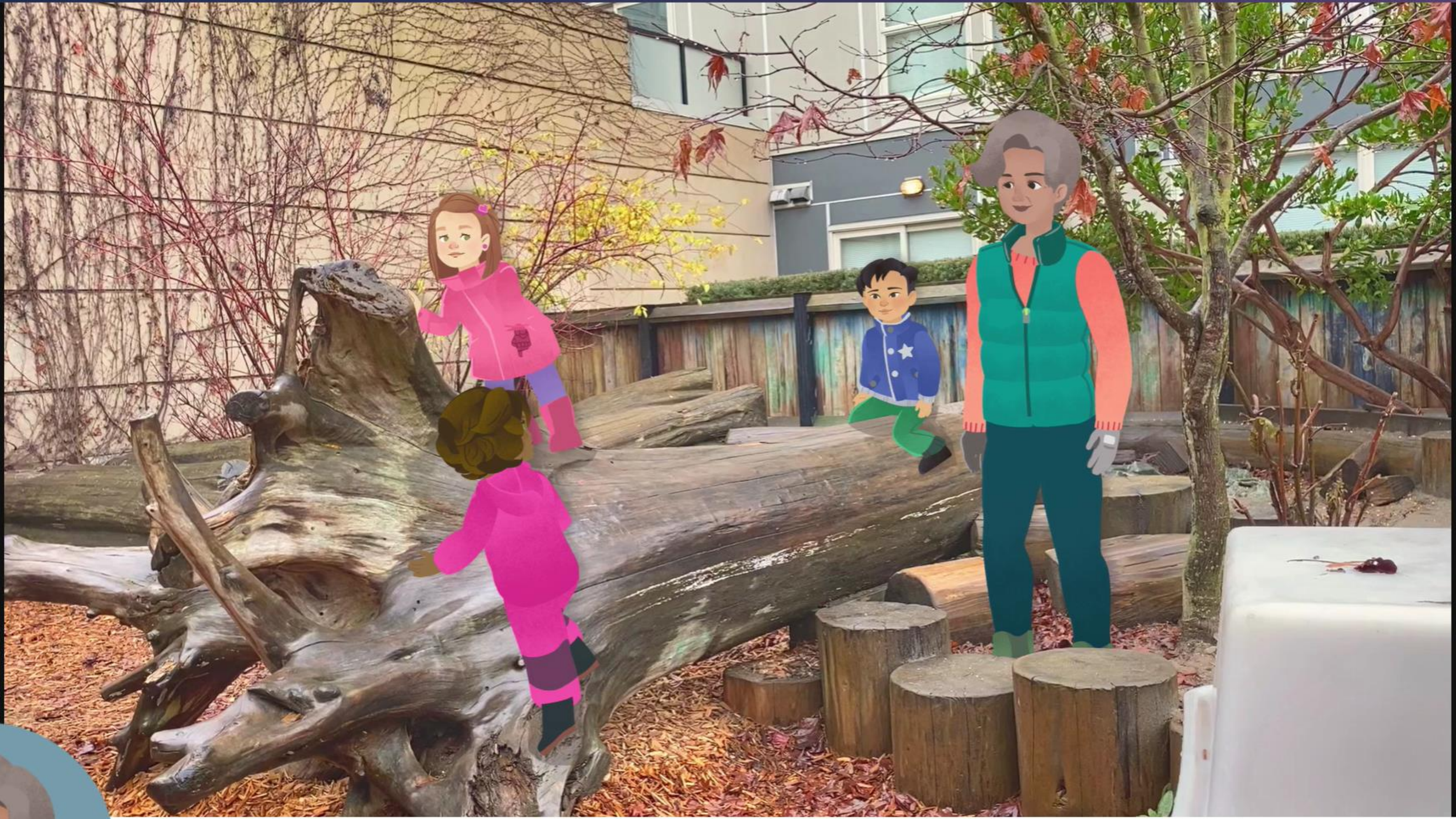

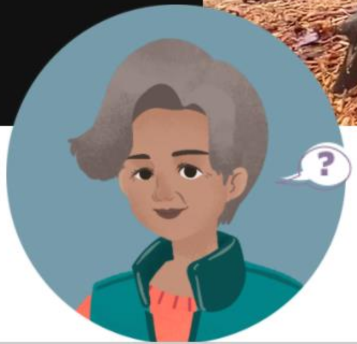

"What should I do?"

- Keep an eye on Jasmine and see how she gets on
- That was close, give Jasmine a hand

Scenarios

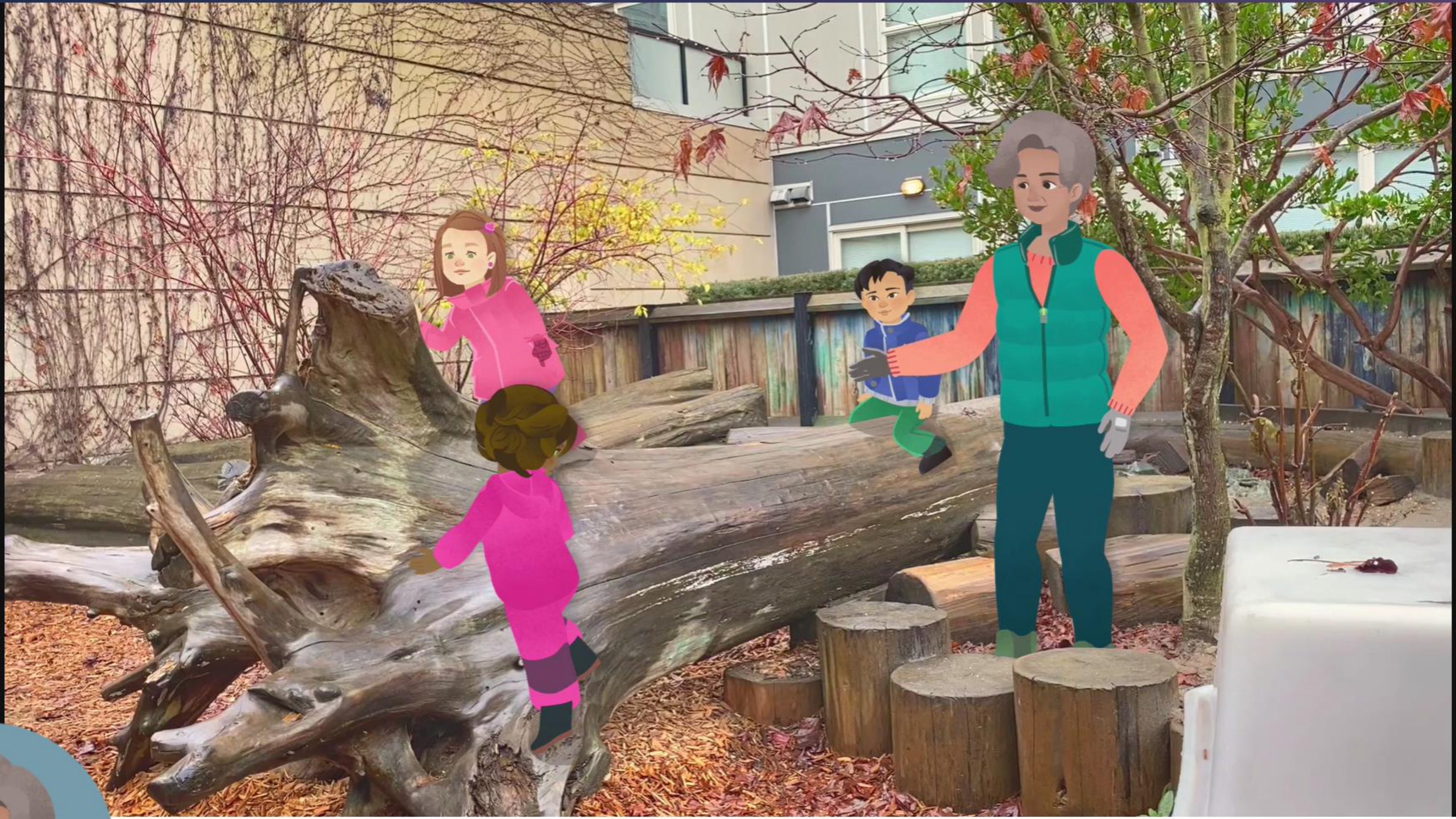

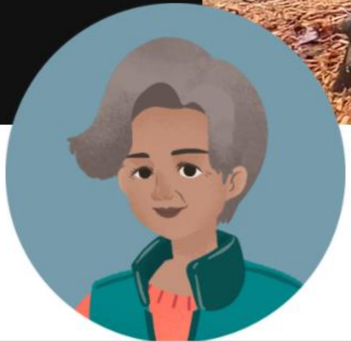

"Be careful!"

NEXT

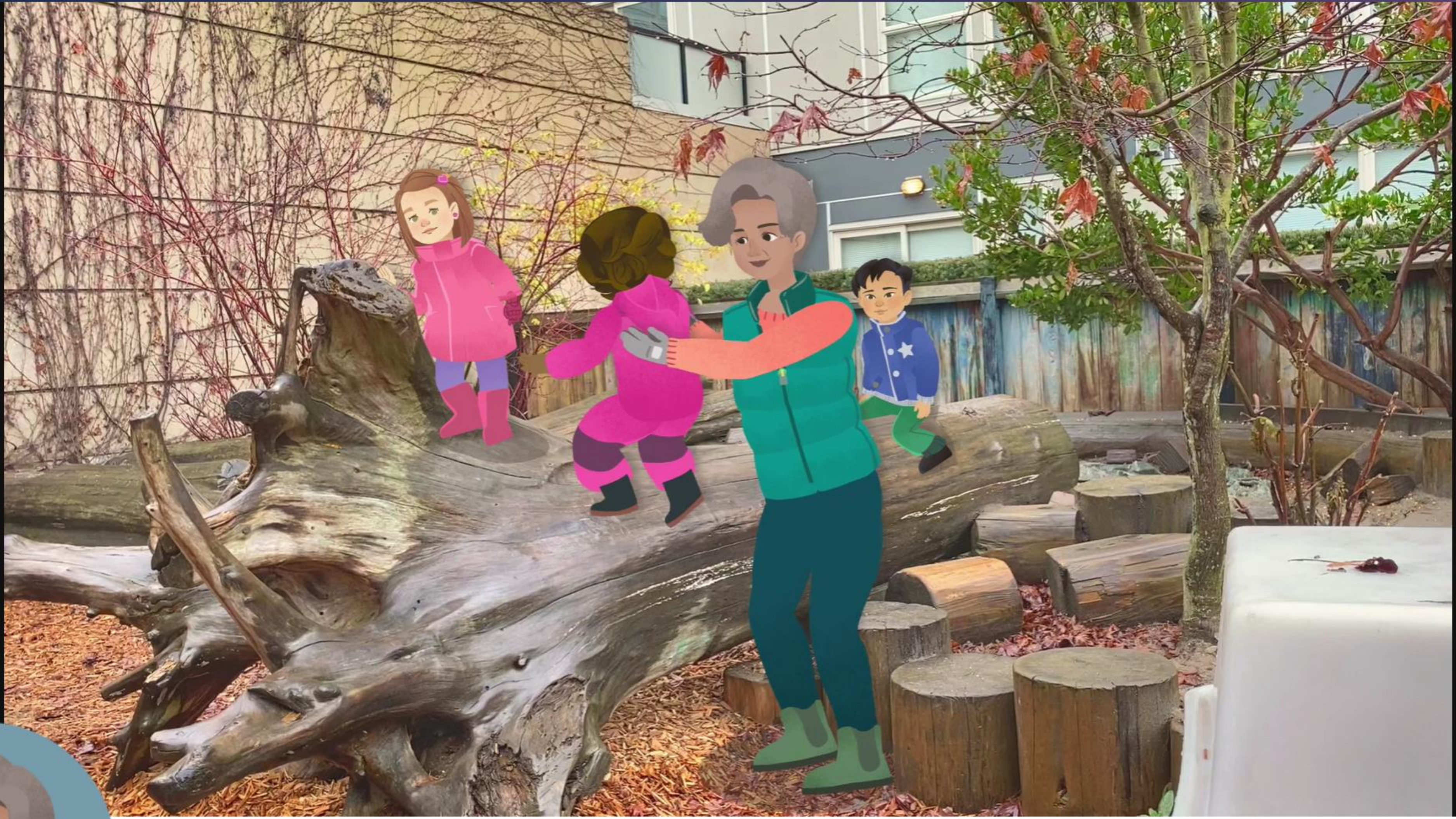

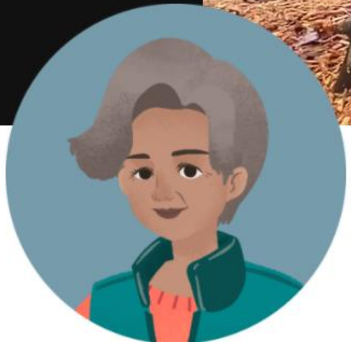

"This is too dangerous for you to climb on your own! Next time, make sure you call me so I can help you up."

NEXT

Scenarios

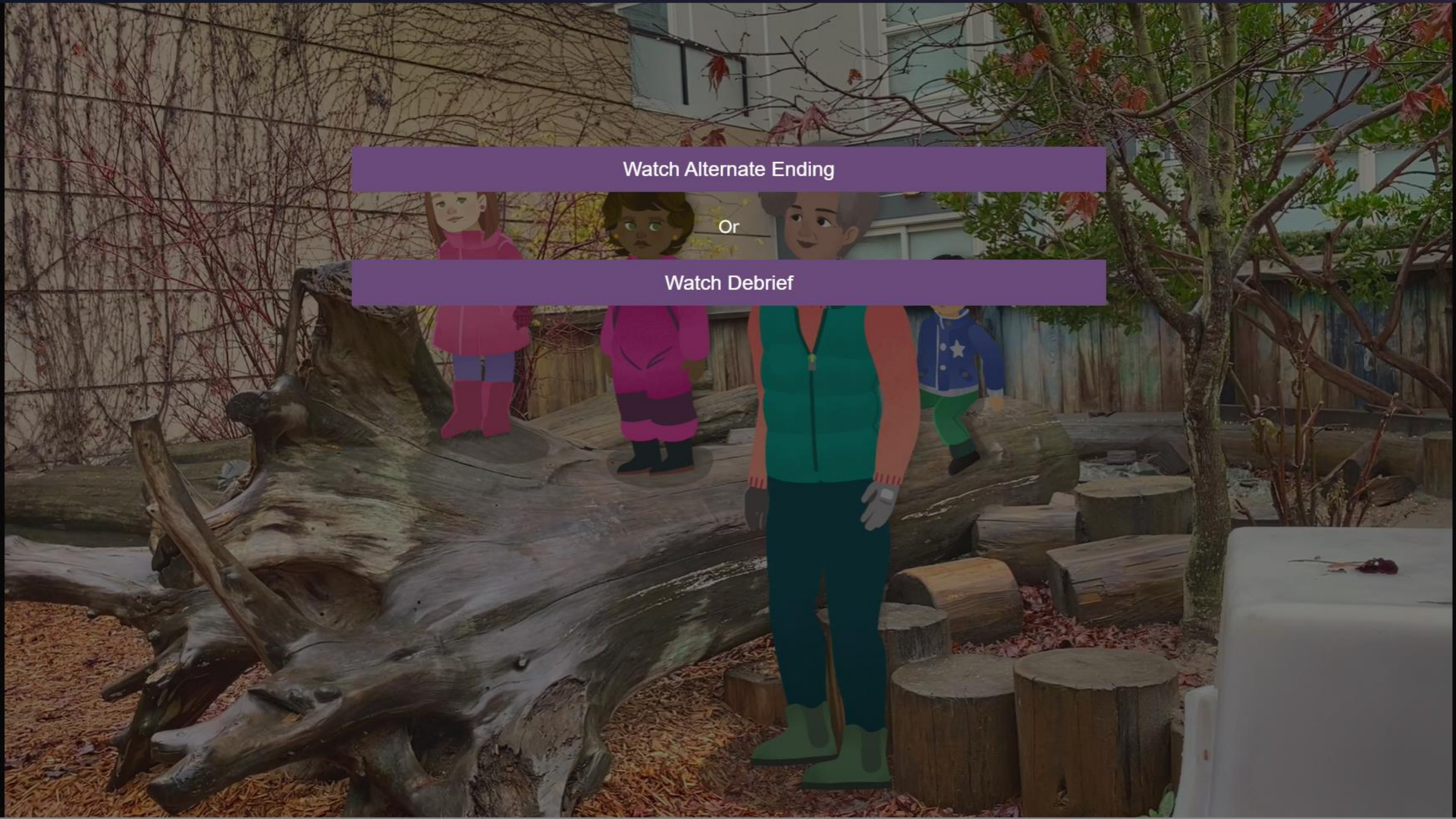

Watch Alternate Ending

Or

Watch Debrief

SKIP ▶

Scenarios

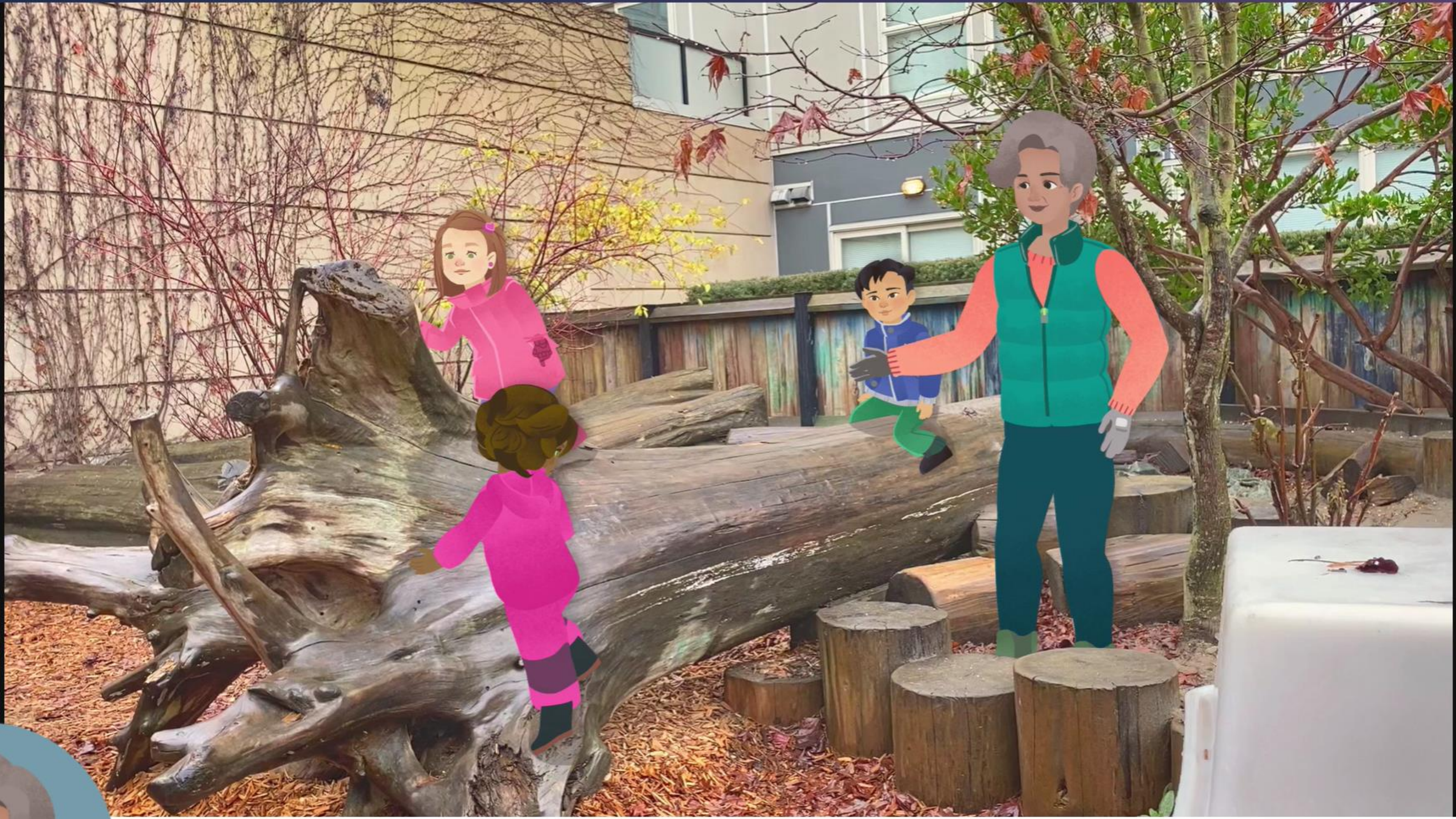

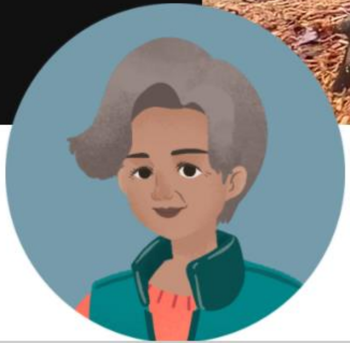

"I like the way you're climbing, using both your hands and feet and maintaining three points of contact. What's your next move?"

NEXT

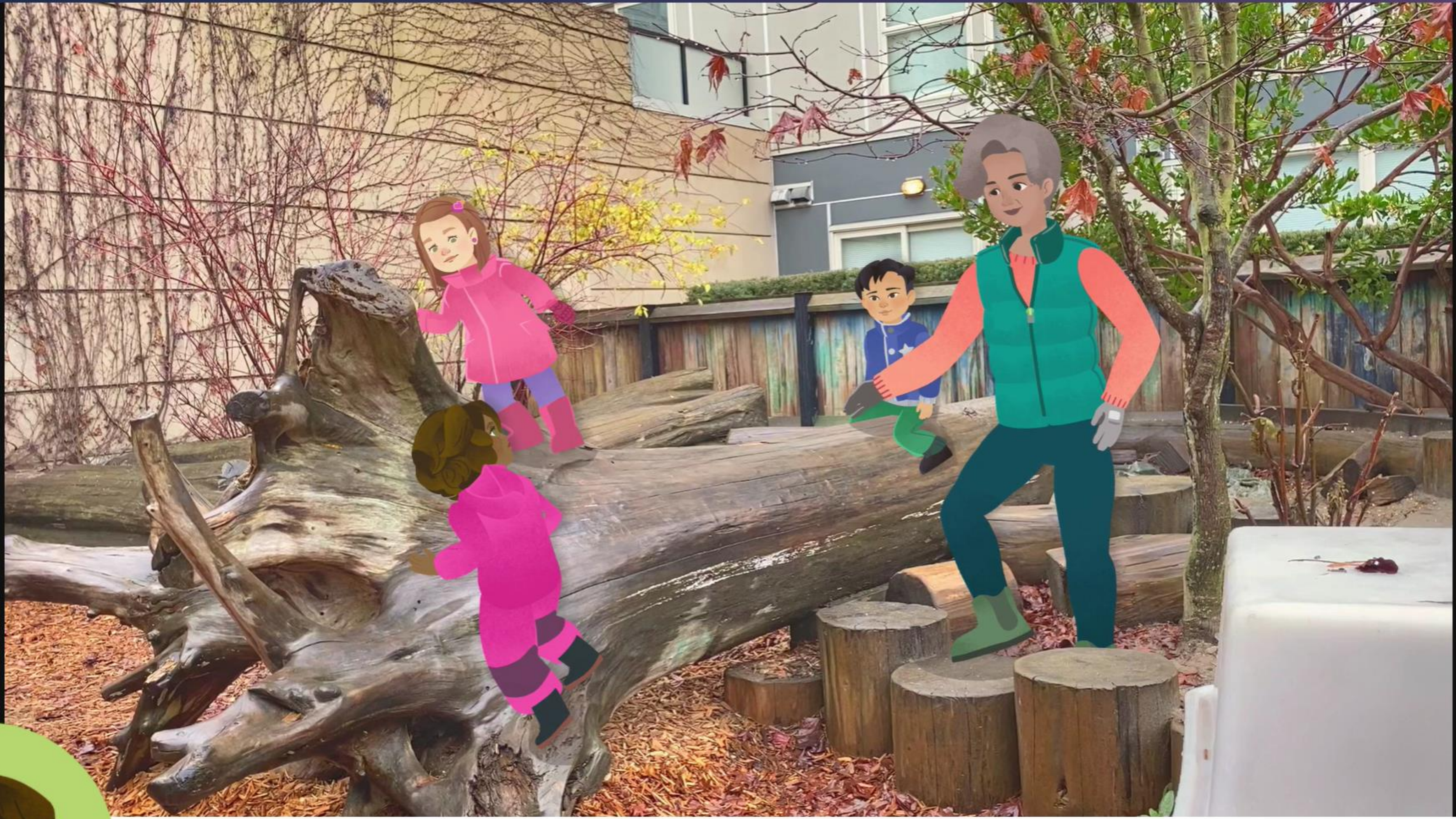

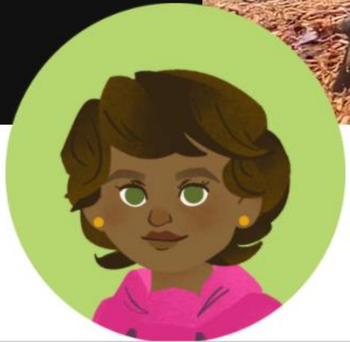

"I'm going to grab on there and pull my foot up here"

NEXT

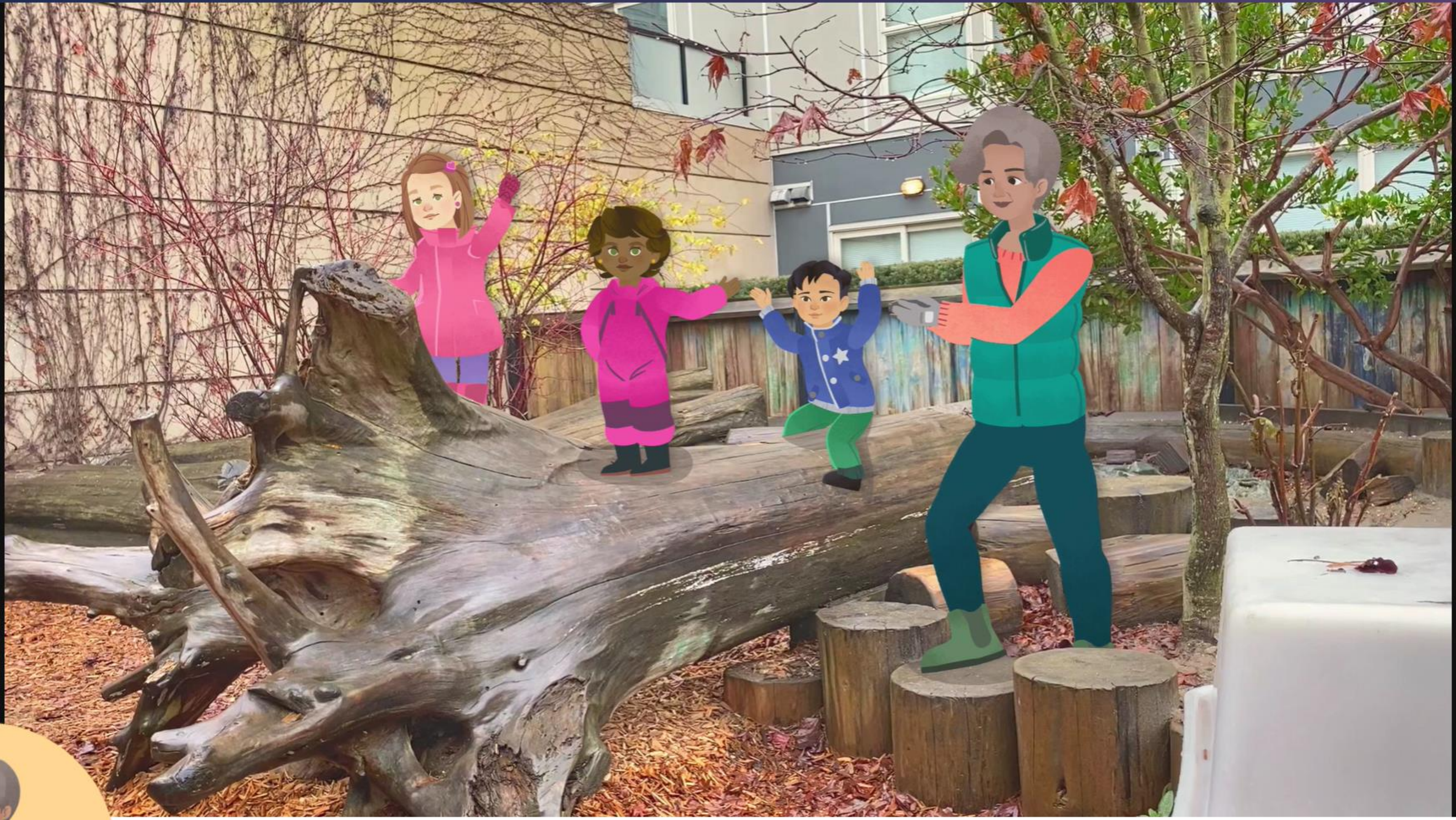

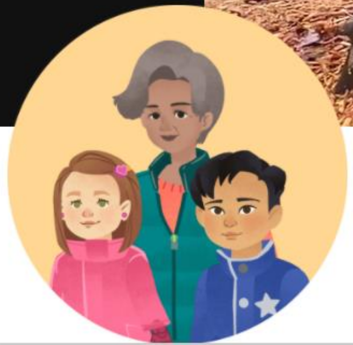

"Good for you! You were brave and made it up all by yourself!"

NEXT ▶

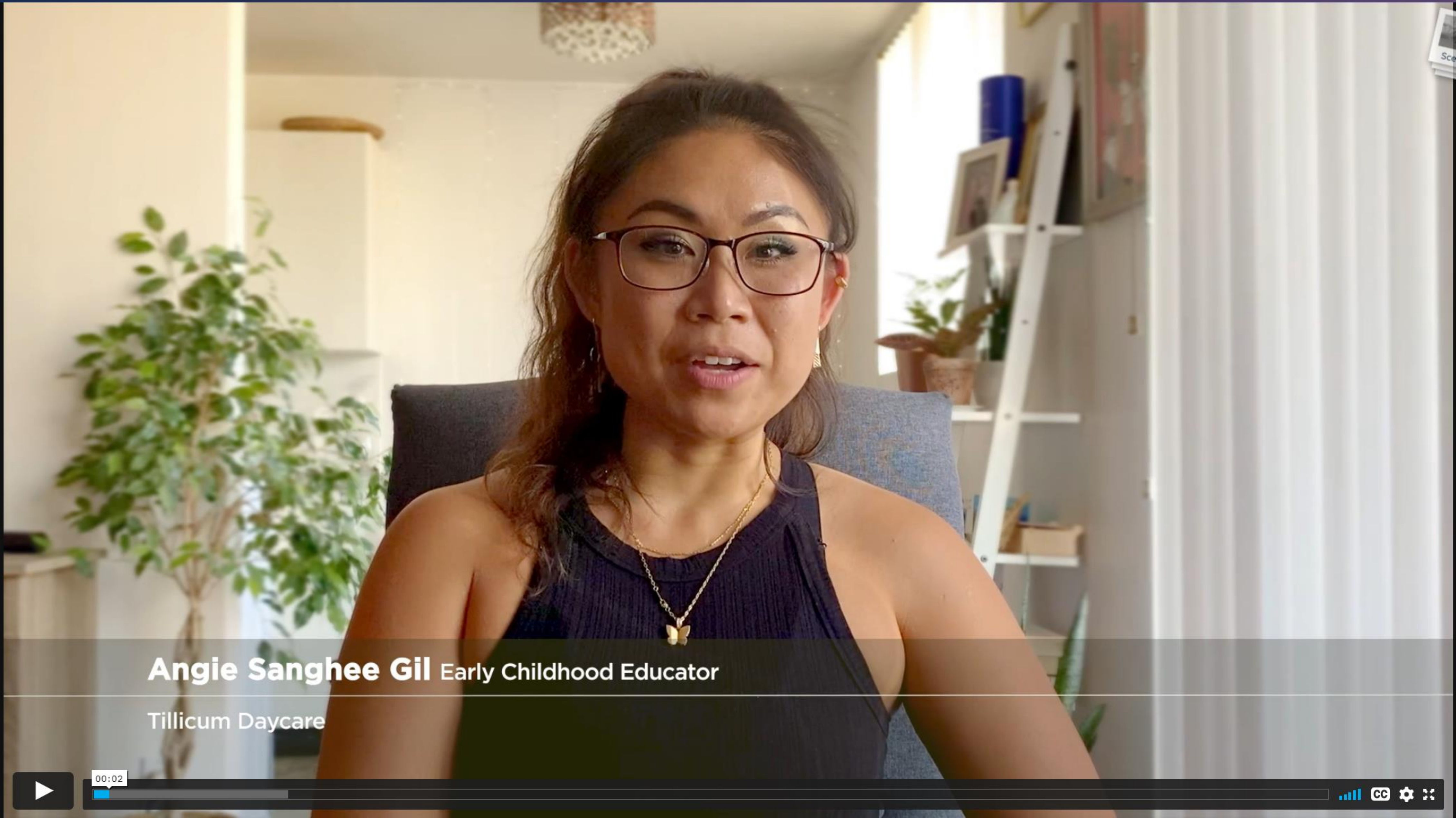
Scenarios
Angie Sanghee Gil Early Childhood Educator
Tillicum Daycare
00:02

What's the most important thing you learned?

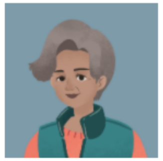

I learned...

Here are some examples if you get stuck

- Children need direct experiences with risk taking and risky play to develop risk management skills.
- When children manage their own risks, they can learn to trust themselves and increase their self-confidence by having succeeded on their own.
- Setting unnecessary limits and boundaries around the children's play can have a cost to my relationship with children. They can feel frustrated, disengaged, and may even need more of my attention to help find things to do, rather than figuring it out for themselves.
- Facilitating the children's risk taking can help me learn about what children are capable of, find new possibilities for play, and find shared joy with the children.
- Risk benefit assessment principles can guide how I facilitate the play.
- My familiarity and relationship with the children can help me learn the capabilities of each child, how much support I need to provide and help build trust in their abilities to keep themselves safe.
- Projecting my anxiety or fear through words or body language can increase the children's fear and make it harder for them to trust their own risk management decisions.
- It is important to not lift children up, but rather let them climb on their own.
- I can provide helpful prompts for children's own risk management (e.g., "What is your next move?"), rather than making the decisions for them.
- Breathing deeply and counting to 17 can help calm my anxiety and give some time for me to see if the children actually need or ask for my help.

Save and Continue

Chapter 2:

Scenarios

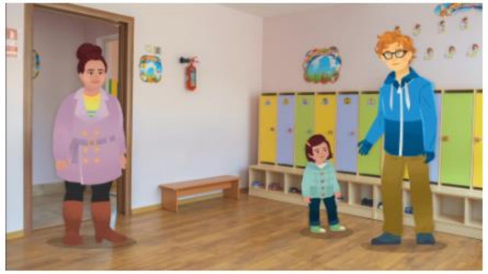

Communicating with Parents/Caregivers

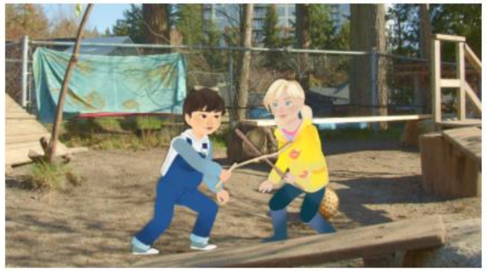

Rough and Tumble Play

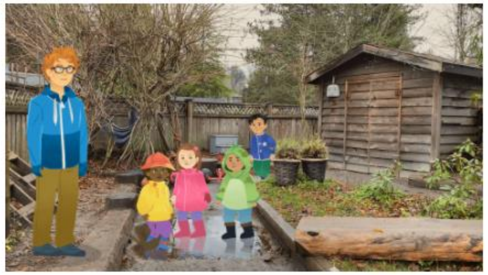

Play at Speed

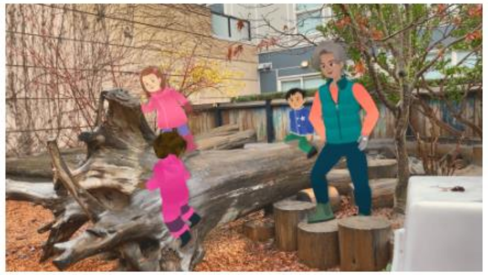

Play at Heights

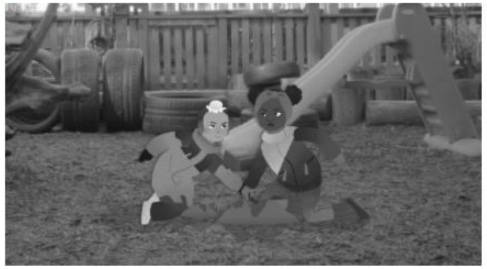

Conflict Resolution

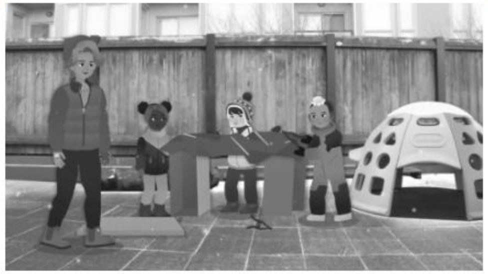

Play with Loose Parts

Scenarios

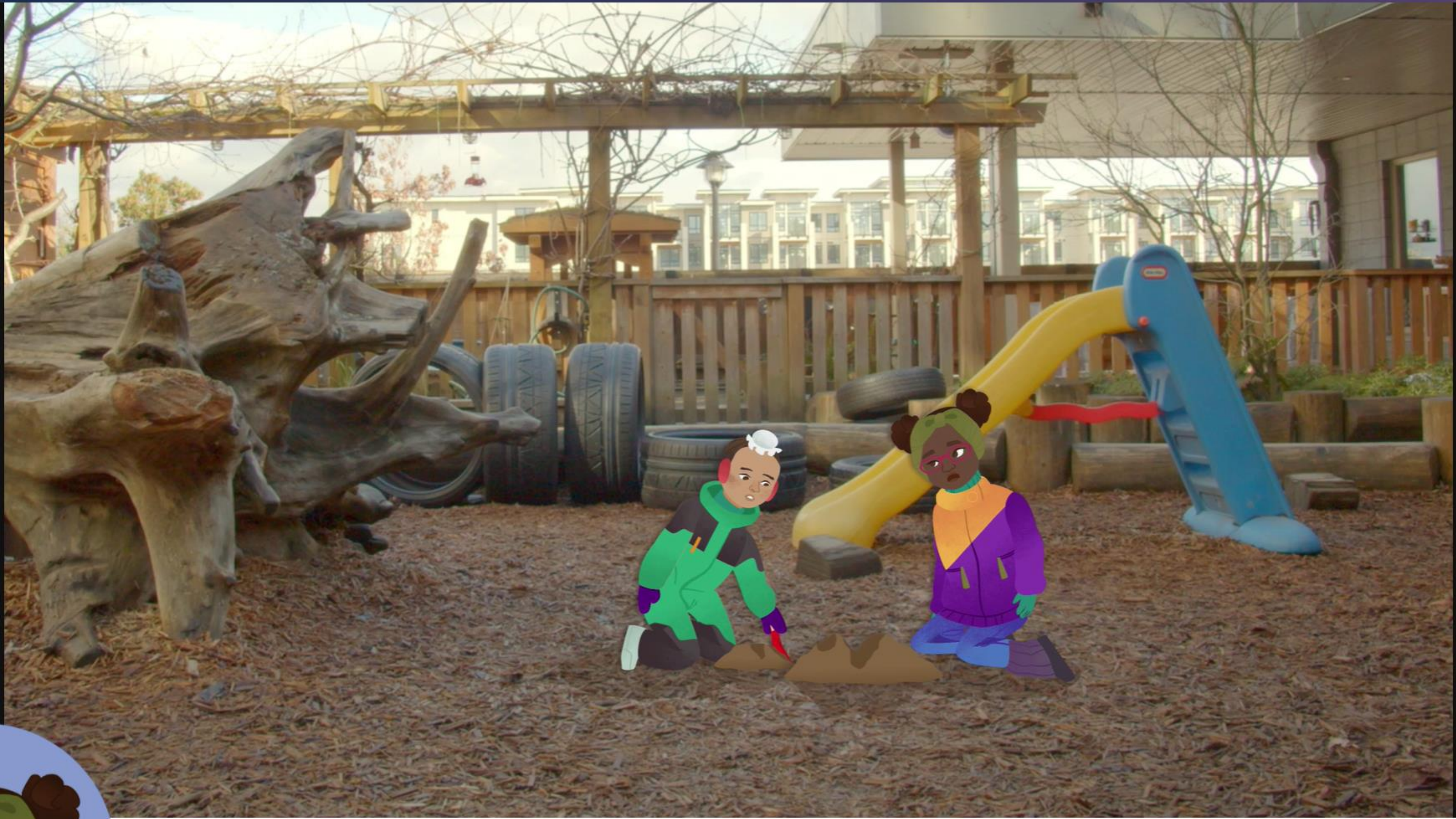

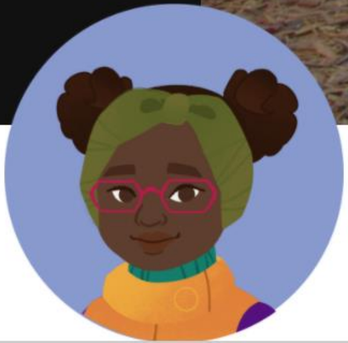

"I was using that! Give it back!"

NEXT ▶

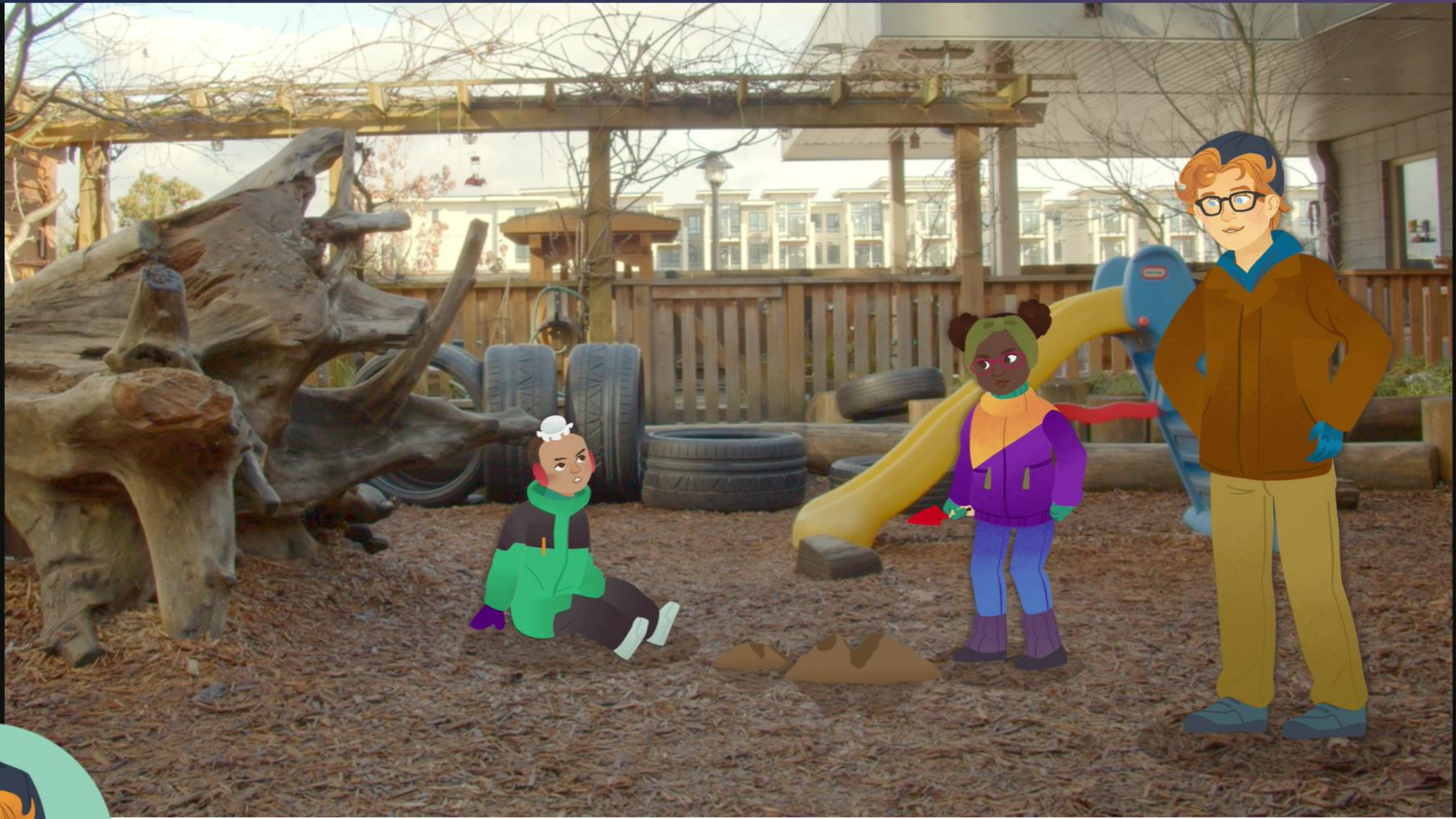

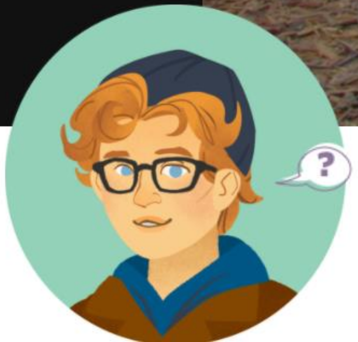

"What should I do?"

▶ Help the children talk through what to do next

▶ Remind the children they have to share

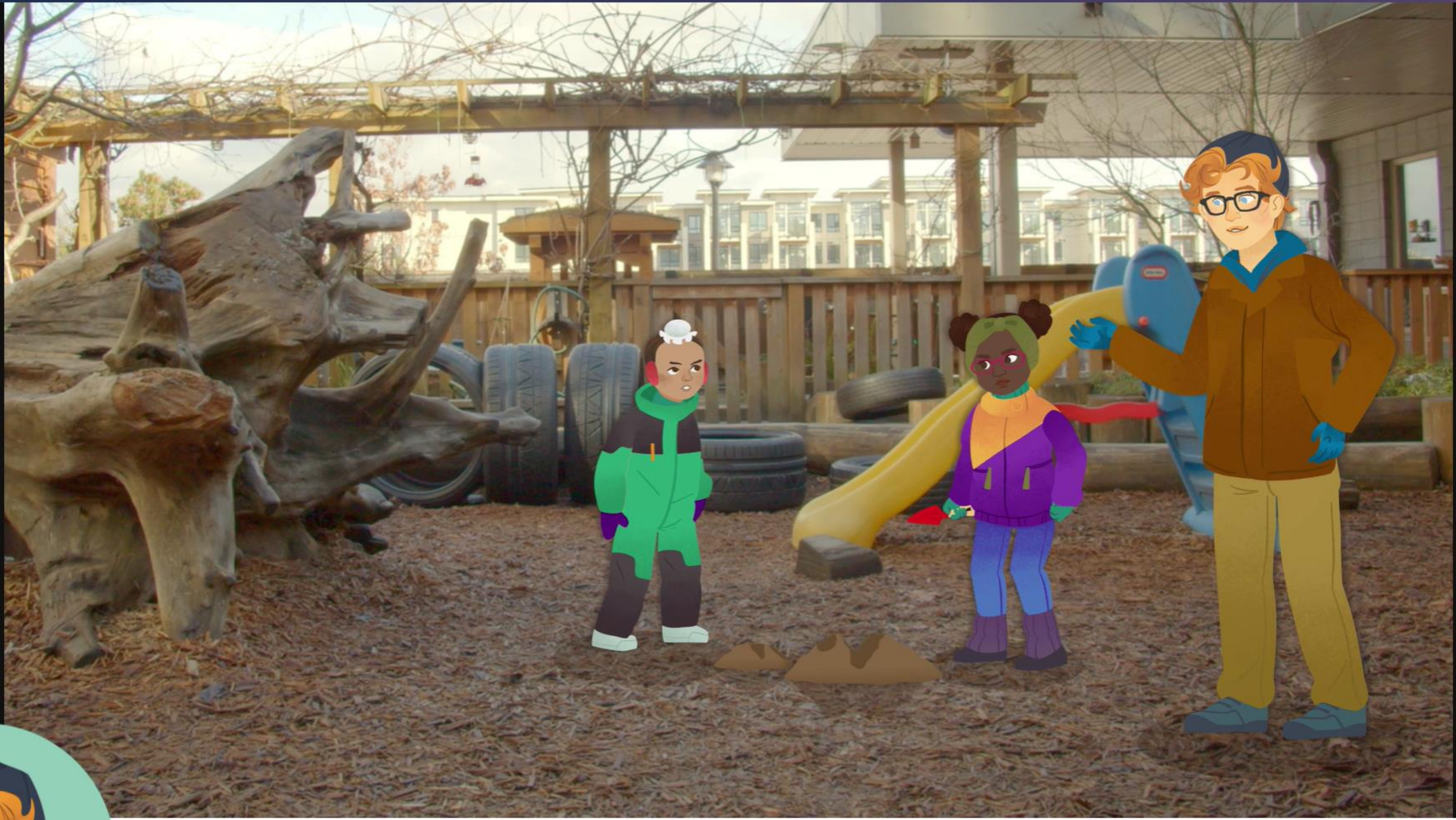

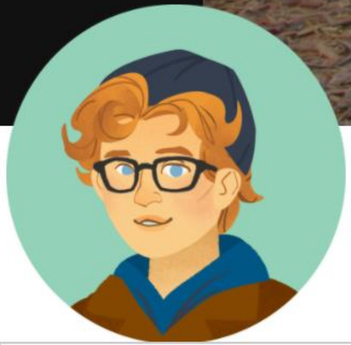

"There aren't enough shovels to go around. It is important to share so everyone gets a turn."

NEXT

Take a risk, go play outside!

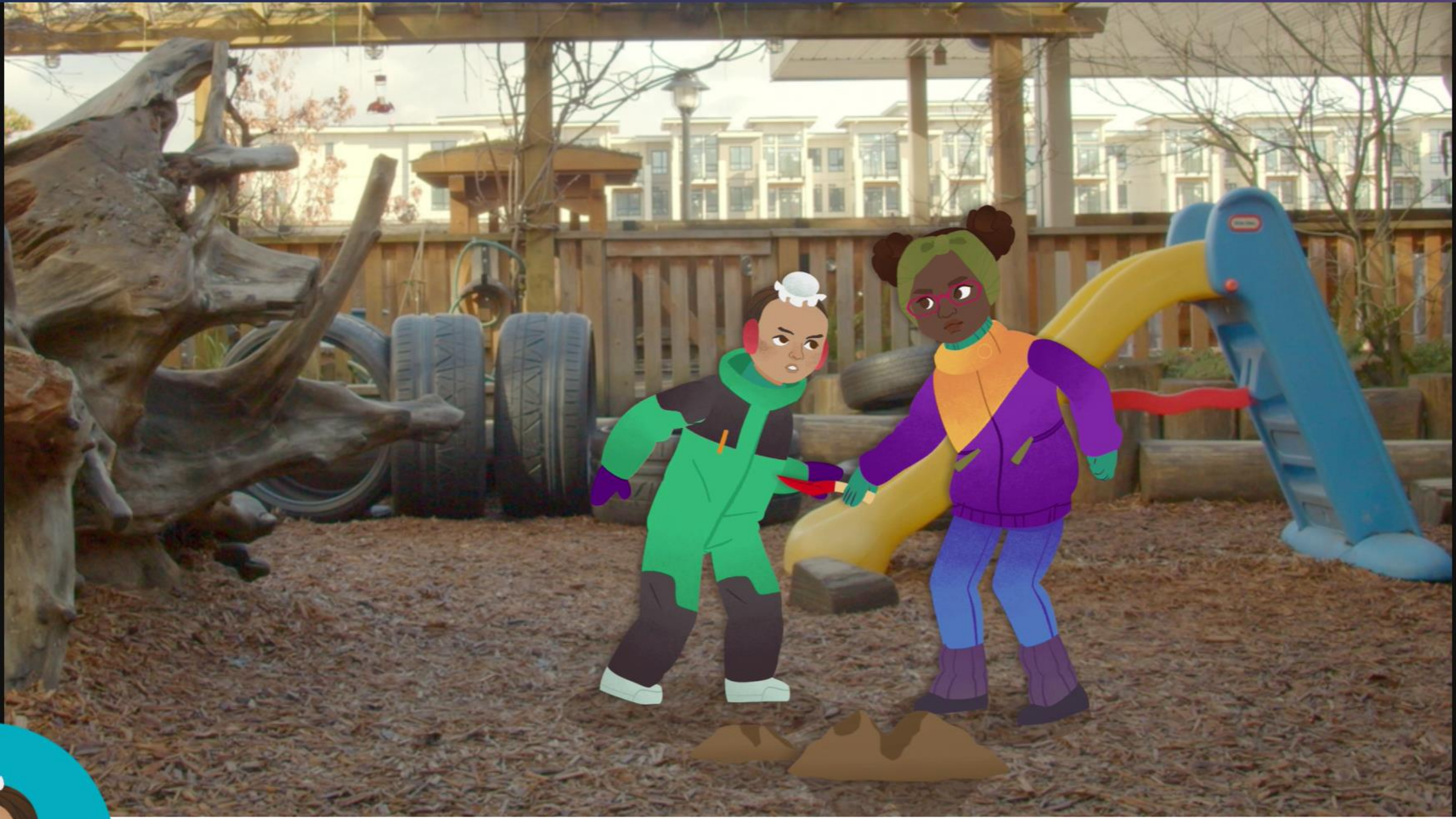

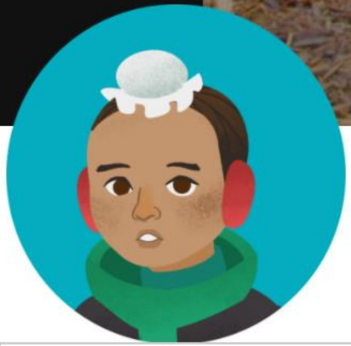

"My turn!"

NEXT ▶

Scenarios

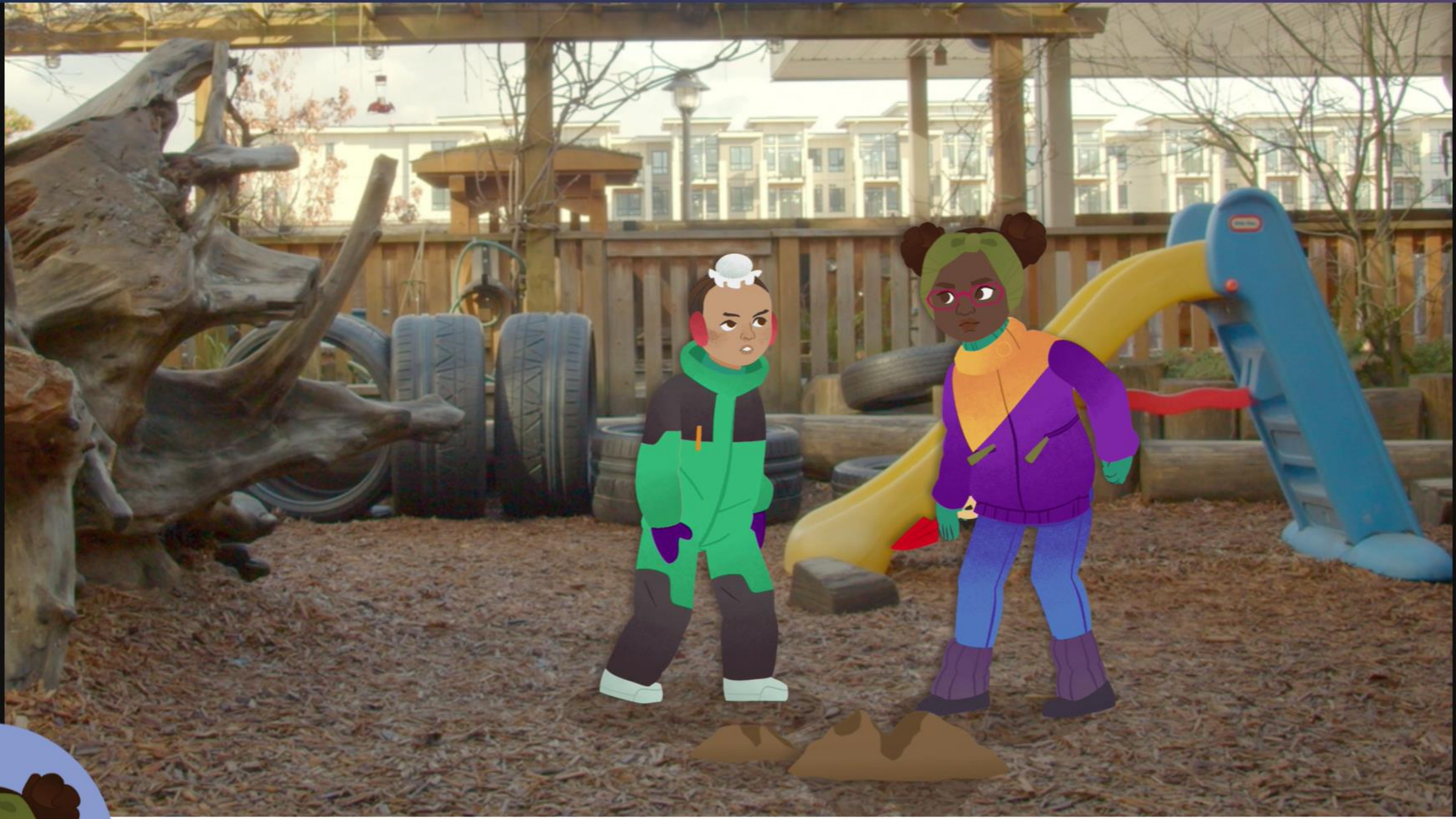

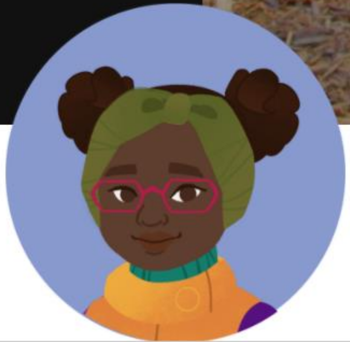

"No! I get to finish my turn first!"

NEXT ▶

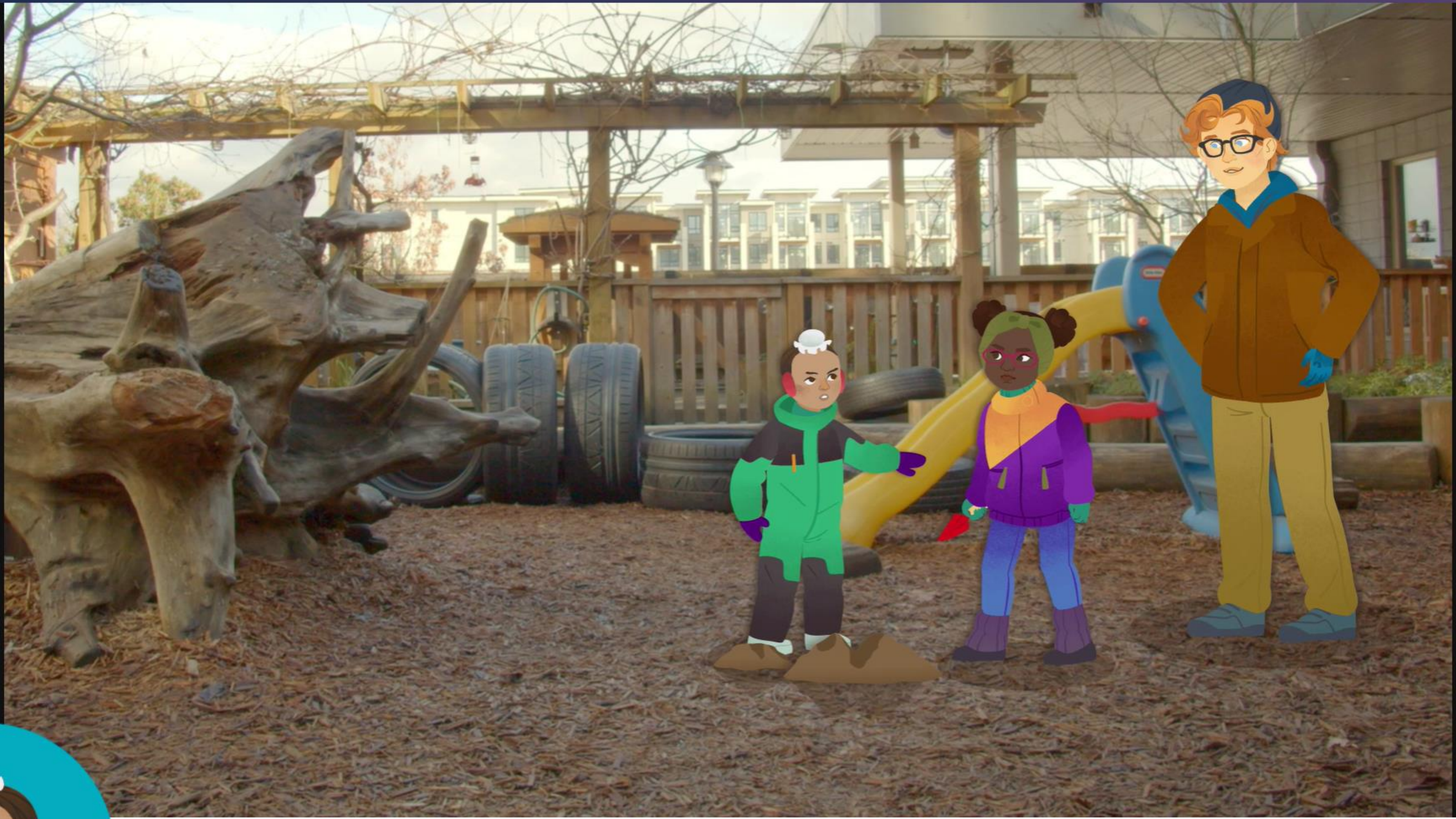

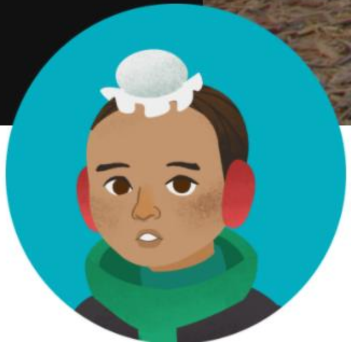

"Lea won't share! She's not being fair!"

NEXT ▶

Scenarios

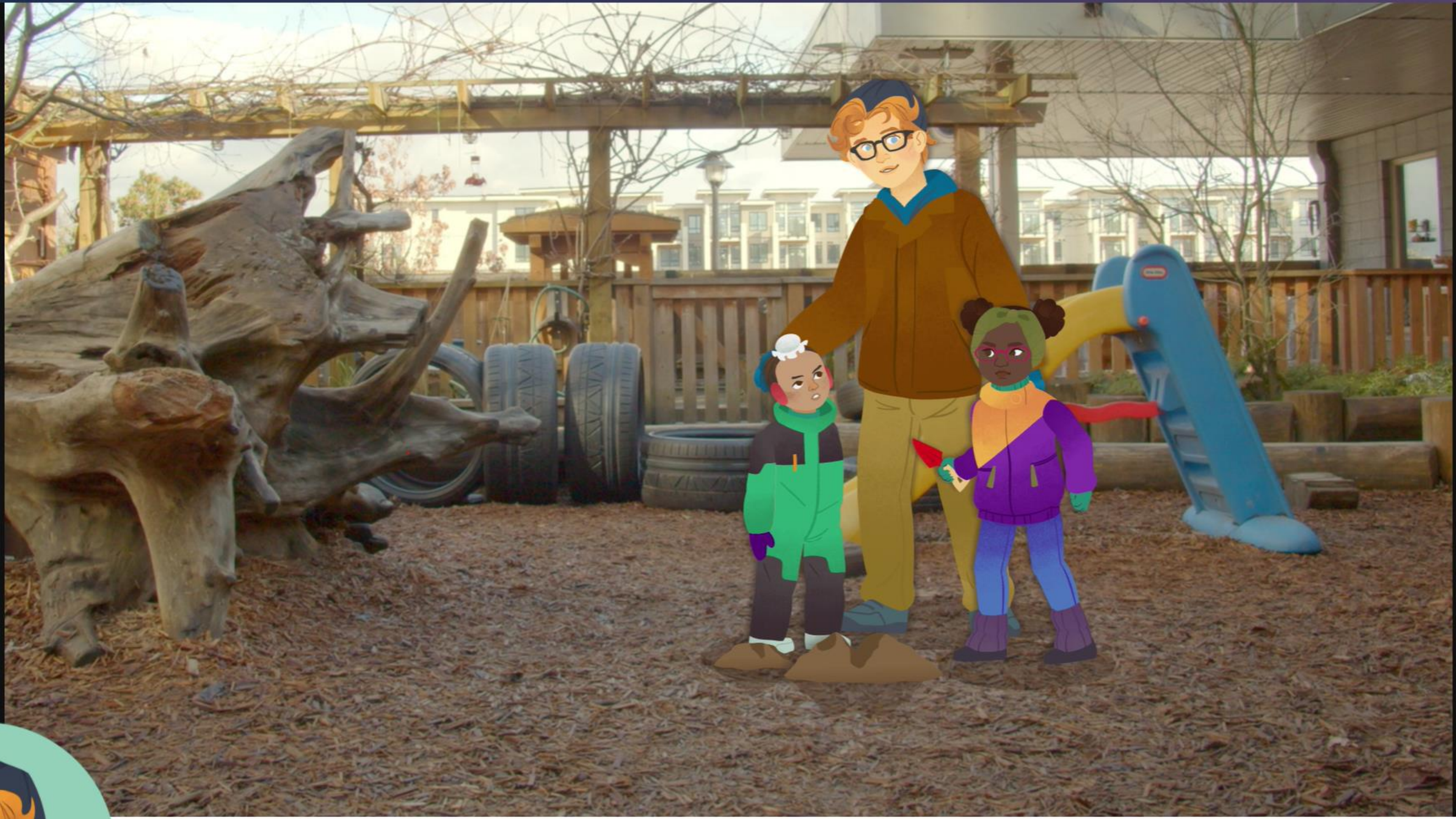

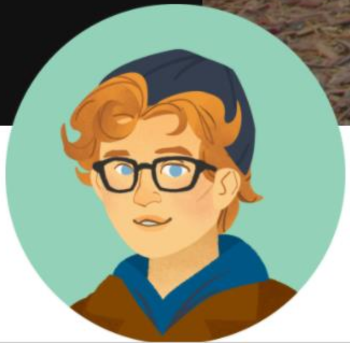

"If you can't share and take turns nicely, then I'm going to have to take the shovel away and no one gets to play with it."

NEXT ▶

Scenarios

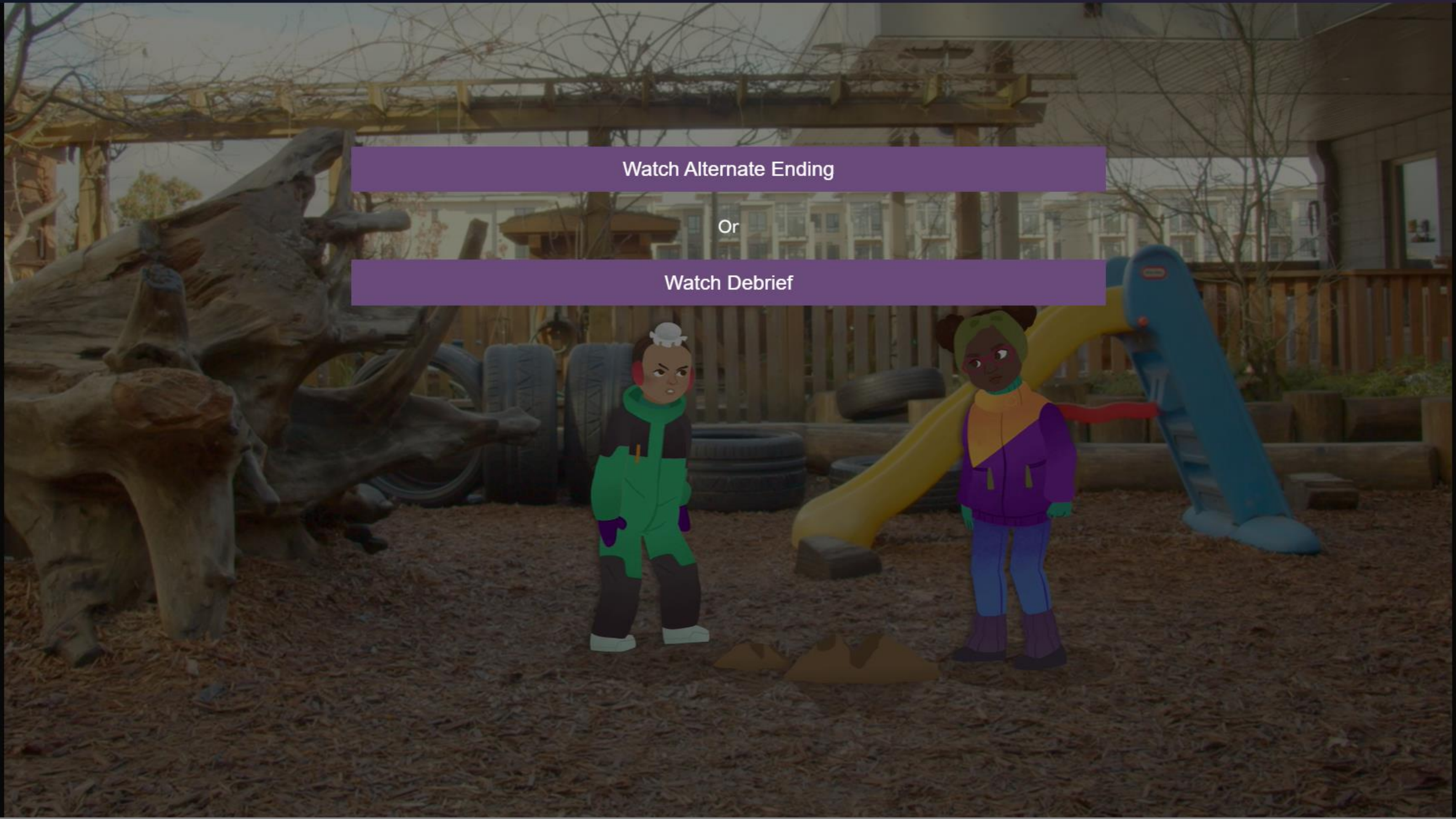

SKIP

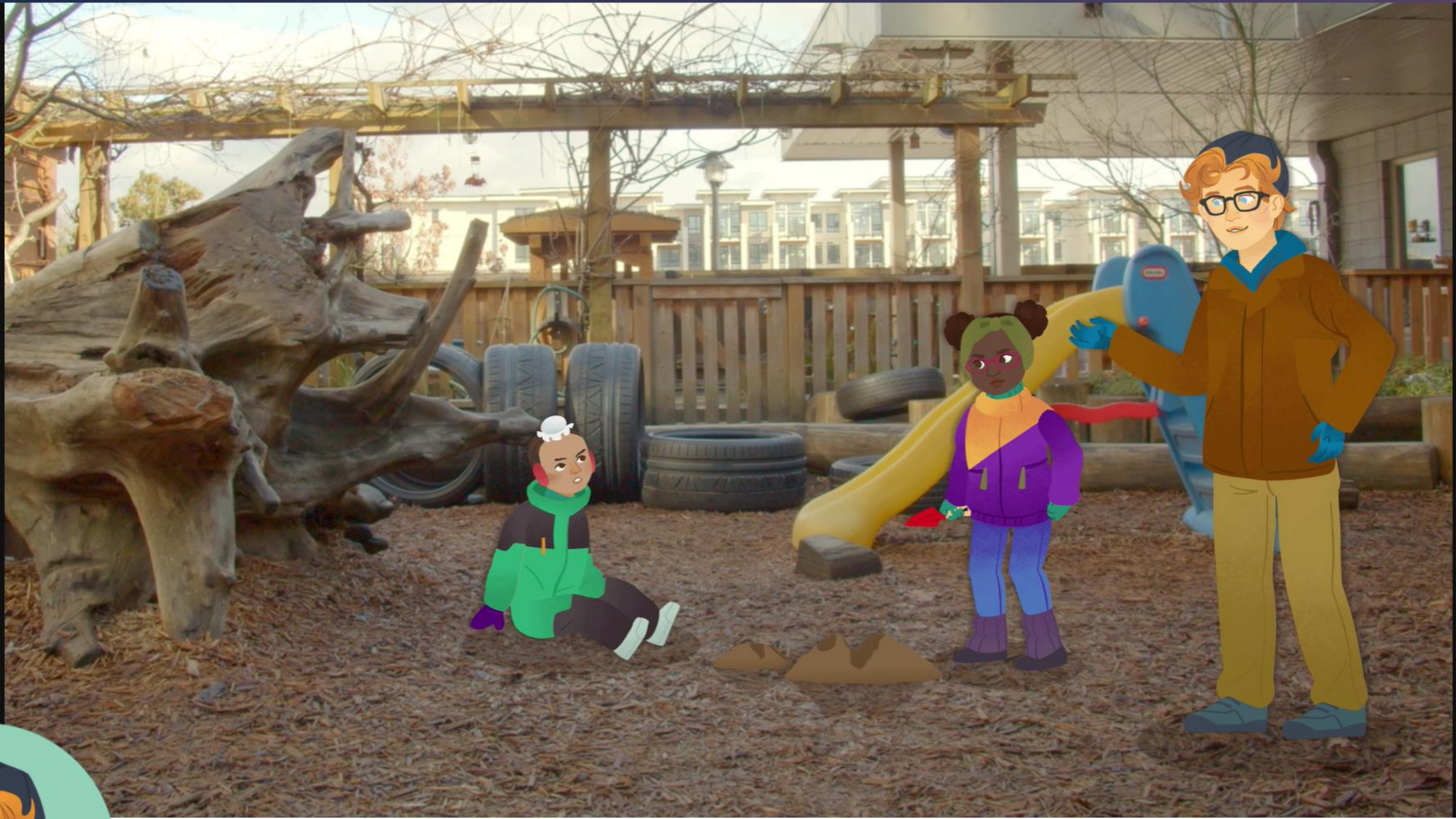

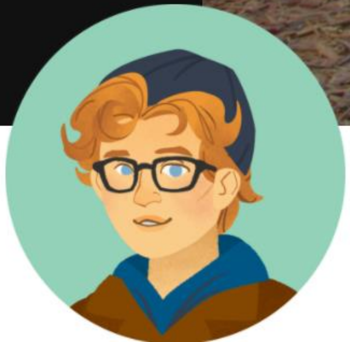

"It can be hard when there aren't enough of what you want to go around. We have to remember that these things don't belong to any one of us, even if you got there first."

NEXT ▶

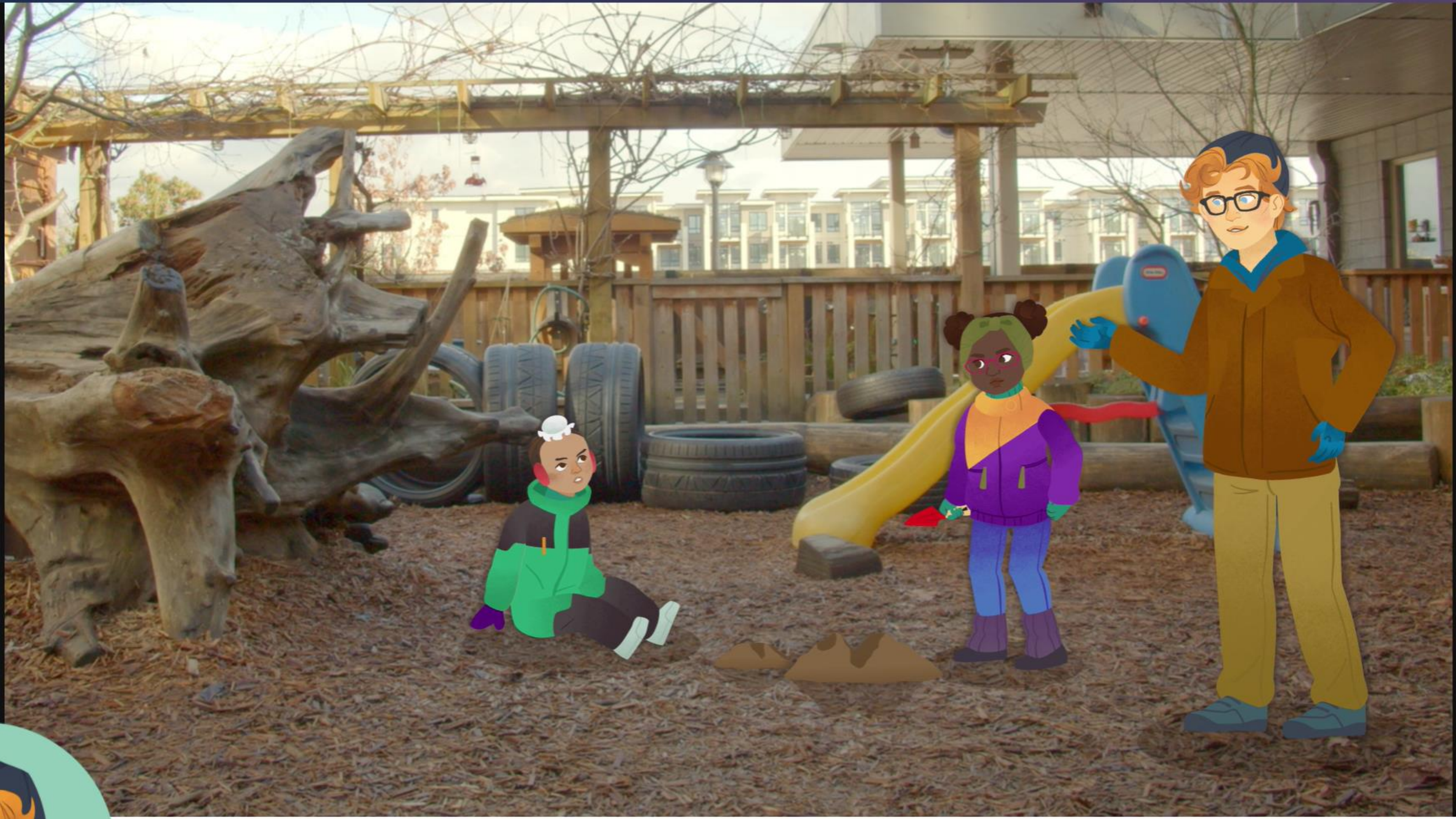

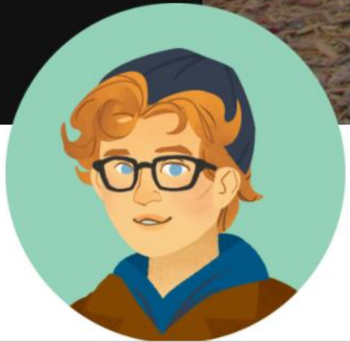

"We have to be patient in taking turns and not grab something from someone but rather check in to see if they're ready to let us use it."

NEXT ▶

Scenarios

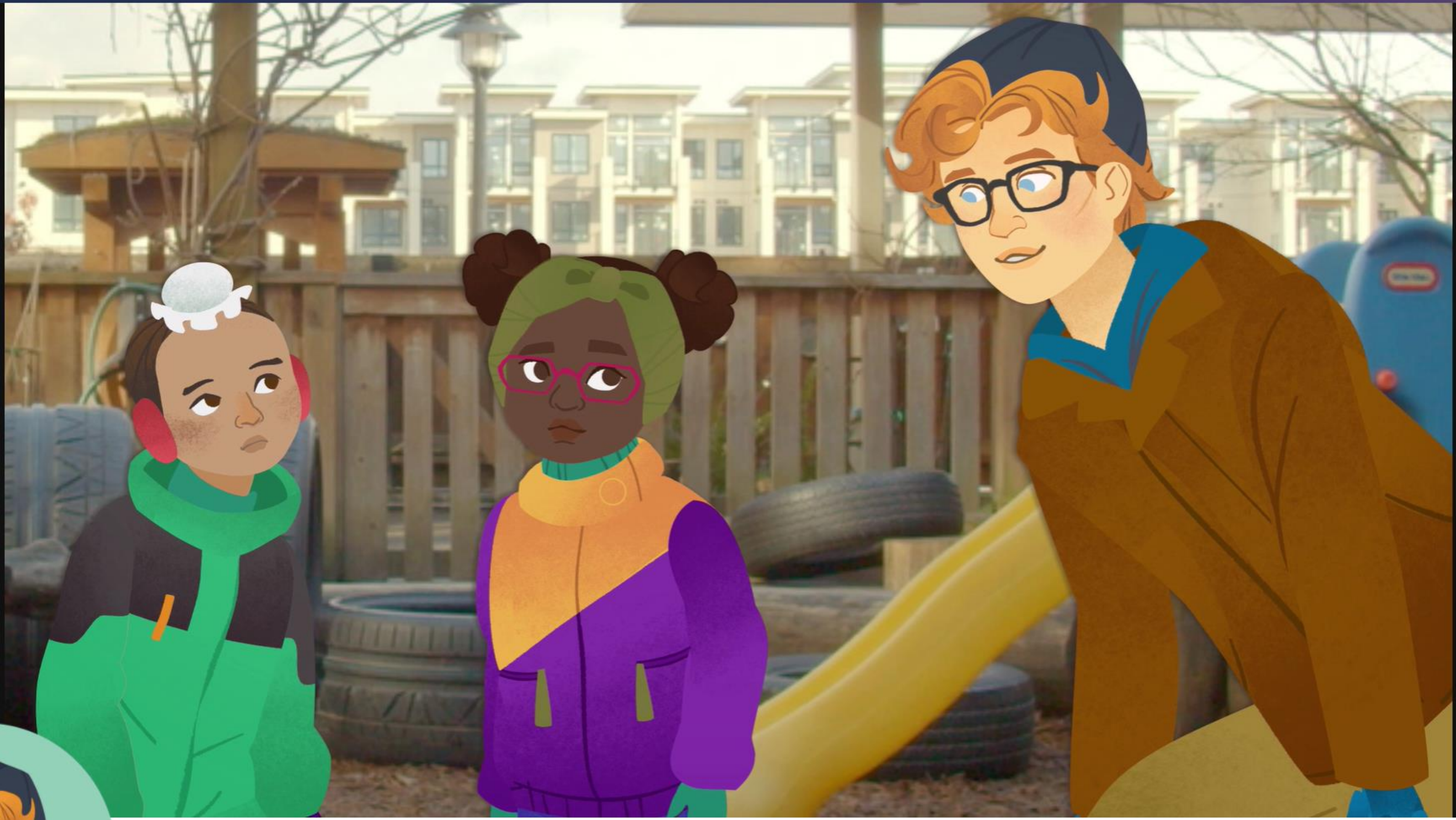

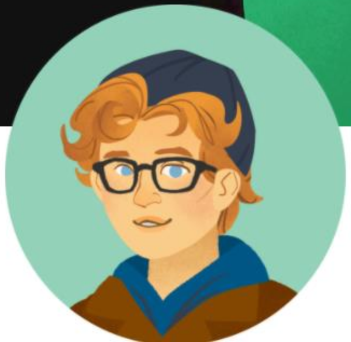

"Alex, what words could you use to see if Lea is ready for you to use the shovel?"

NEXT

Take a risk, go play outside!

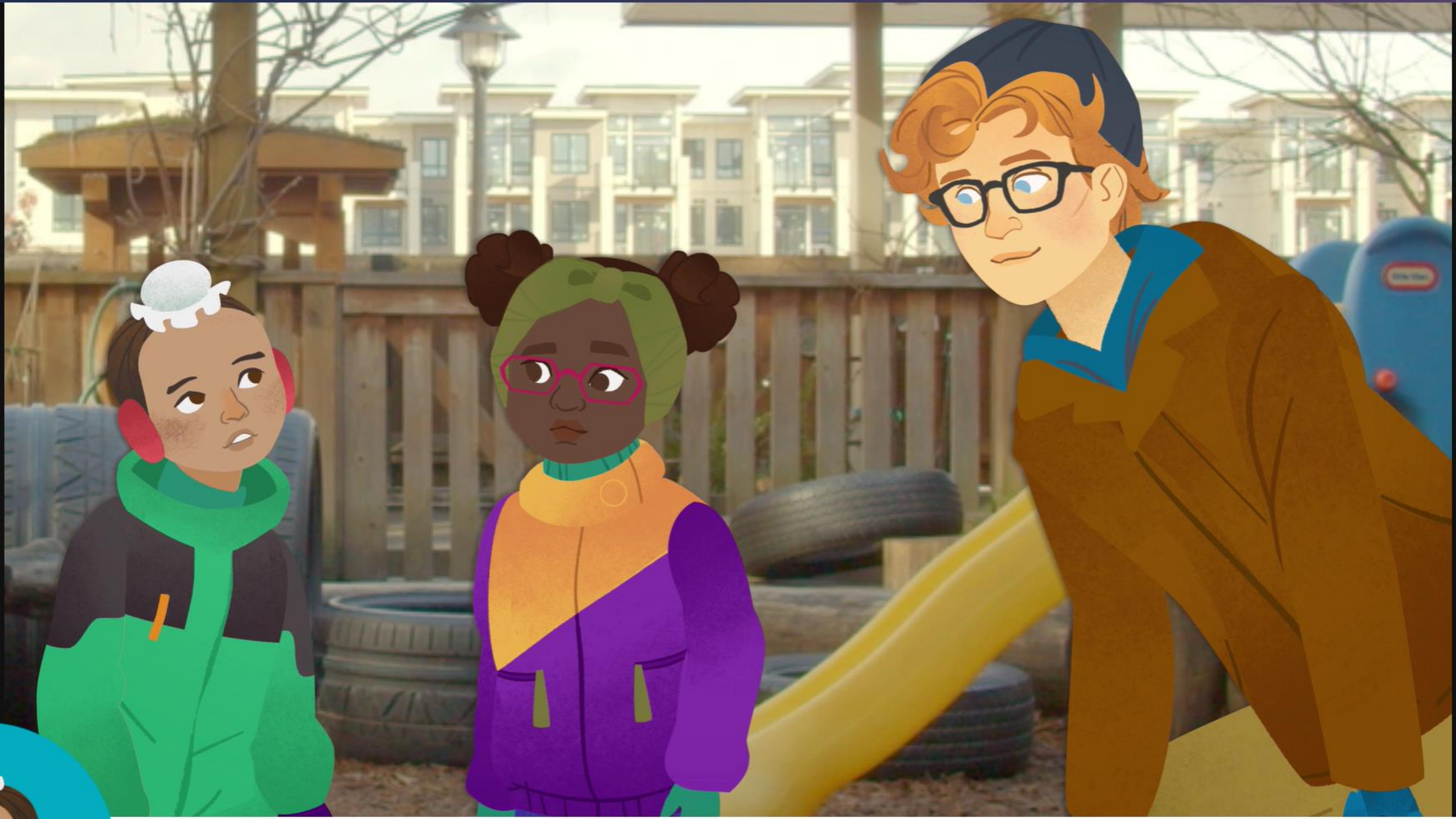

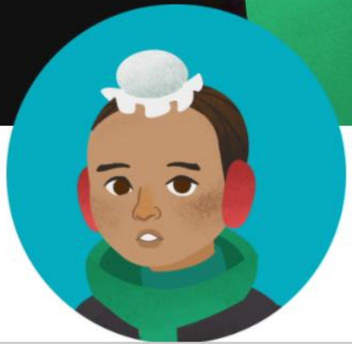

"Can I have the shovel?"

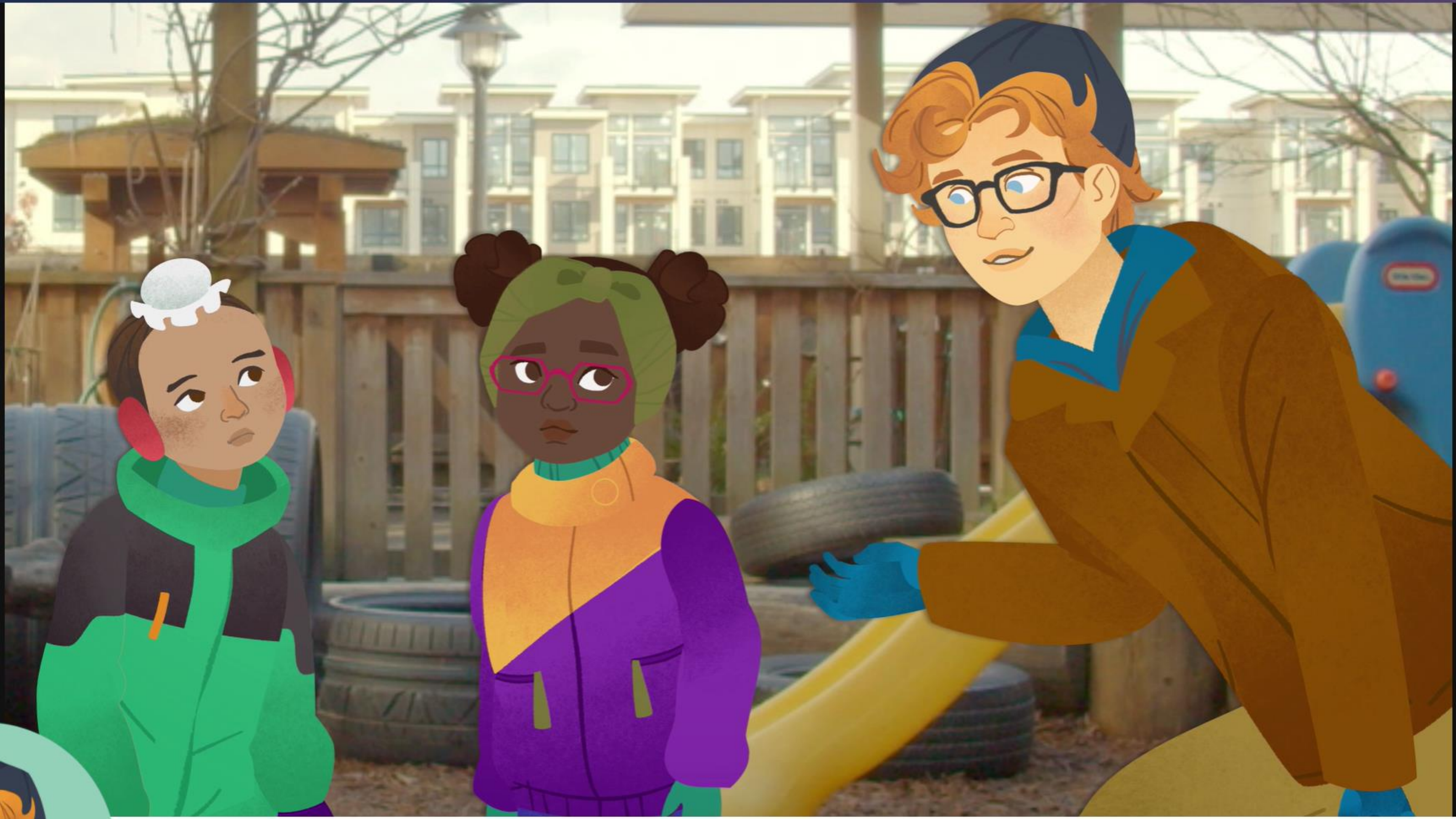

Scenarios

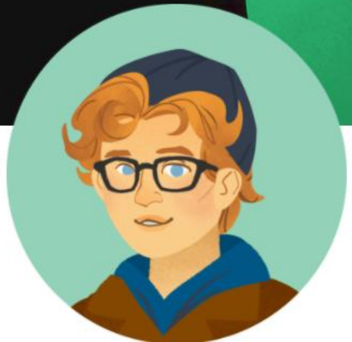

"Yes, you could ask Lea if it's being used for anything. Do you want to try asking that now?"

NEXT ▶

Take a risk, go play outside!

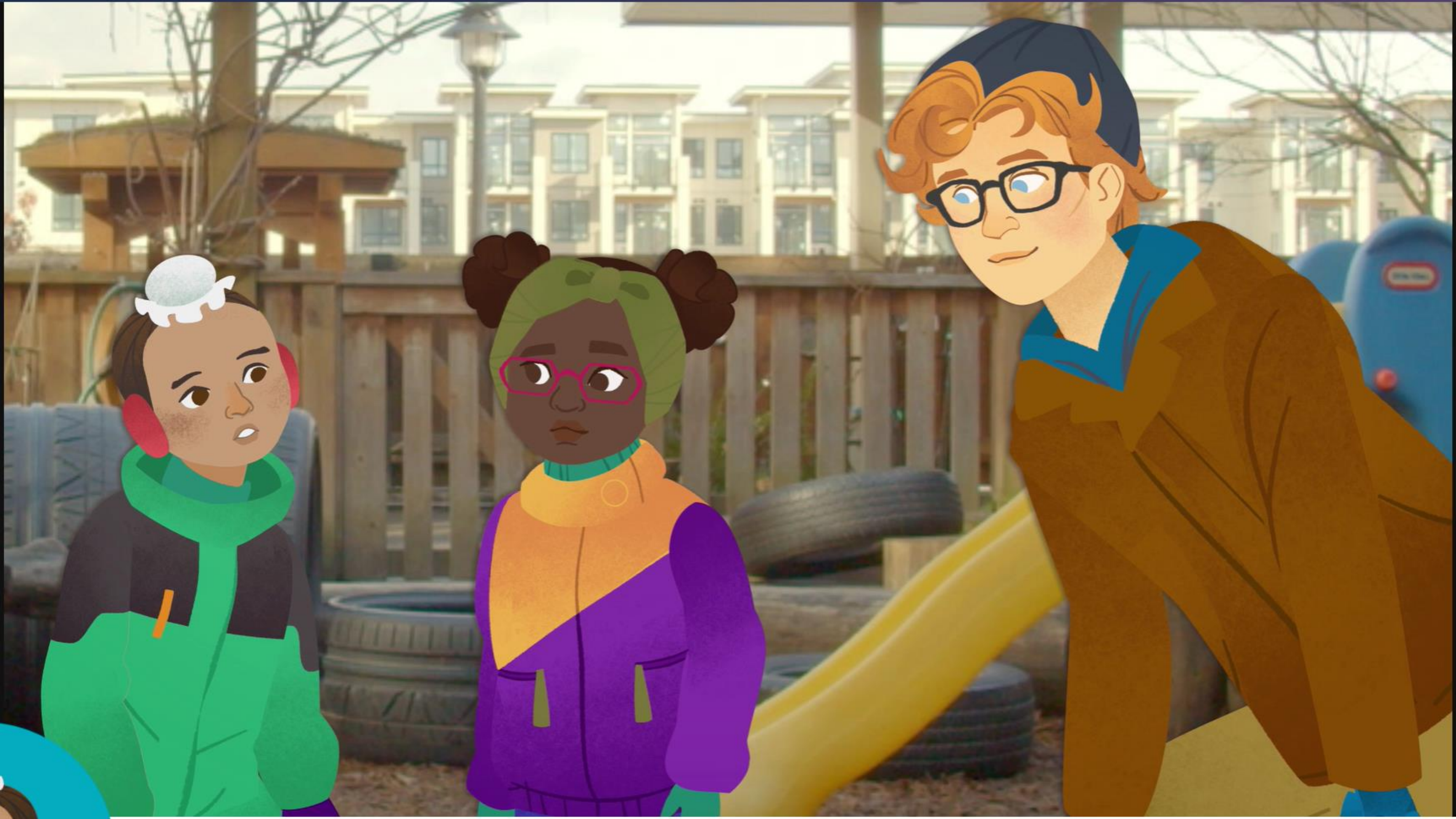

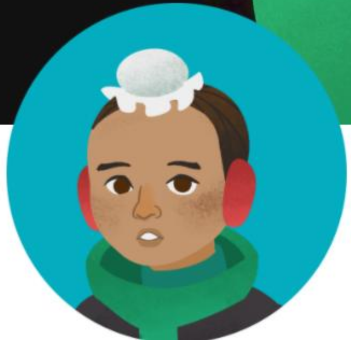

"Is it being used for anything?"

NEXT

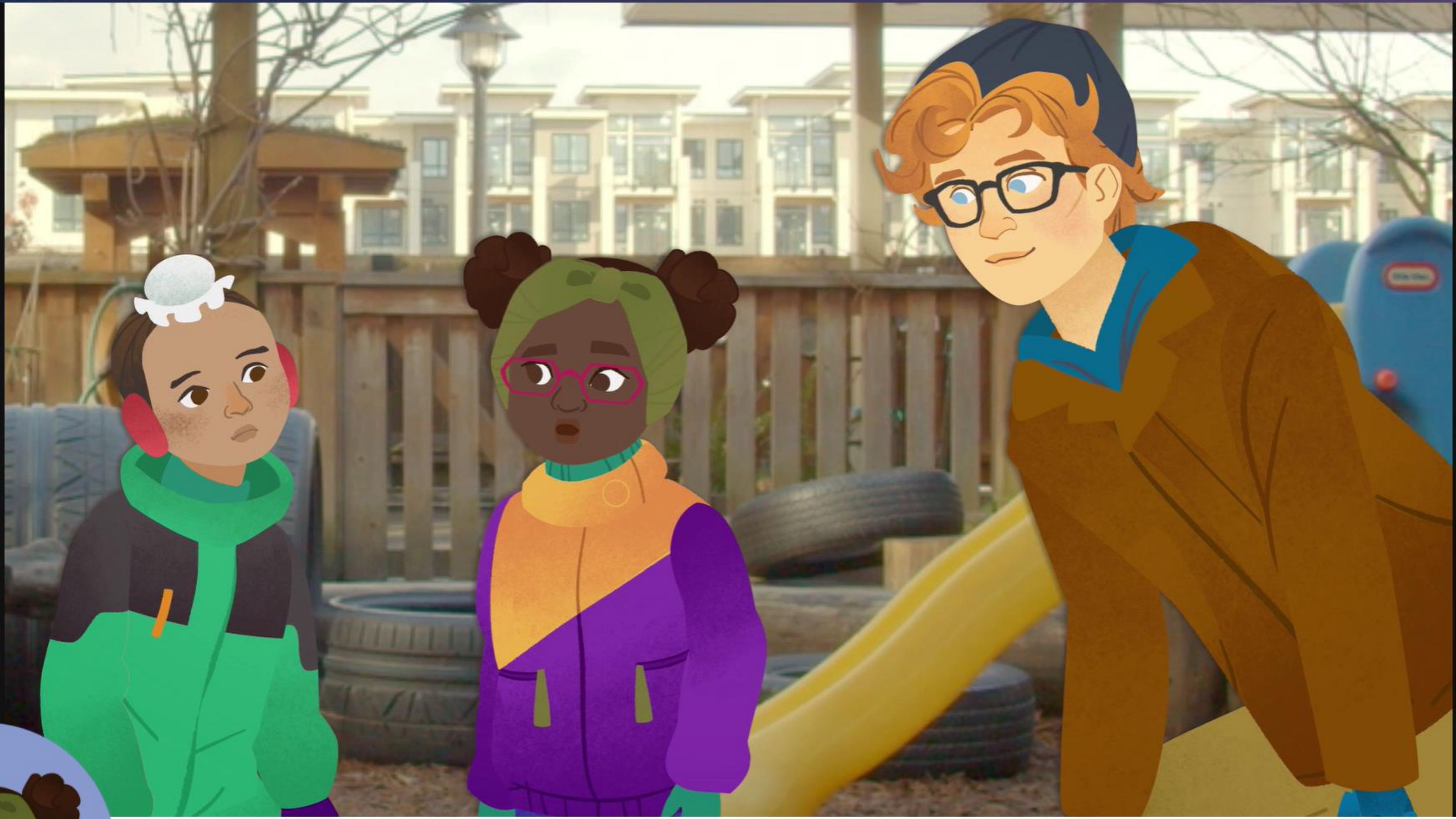

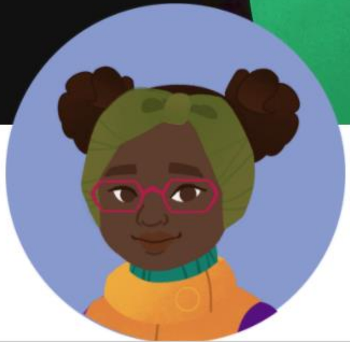

"Yeah, I need it"

NEXT ▶

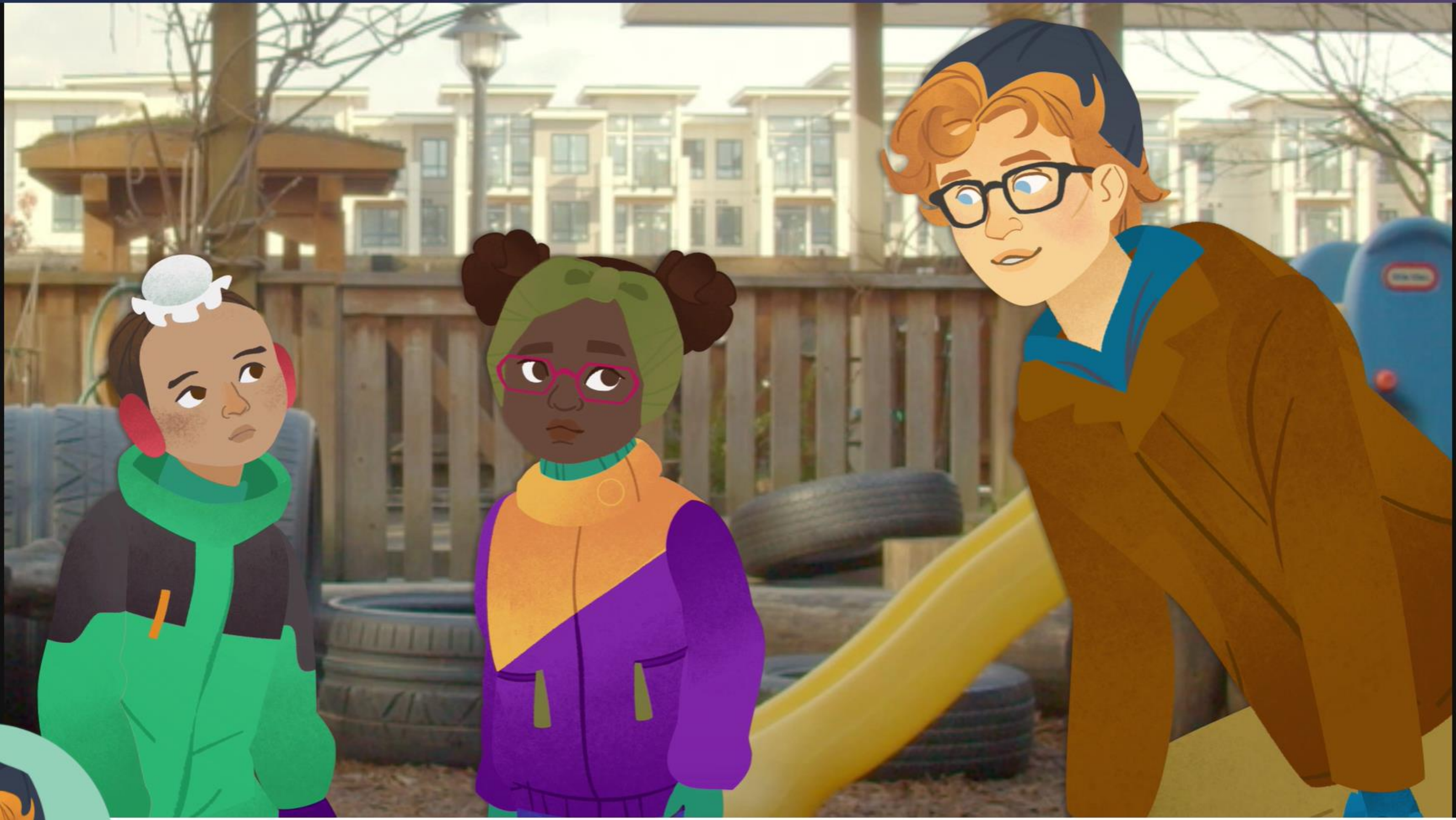

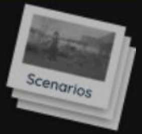

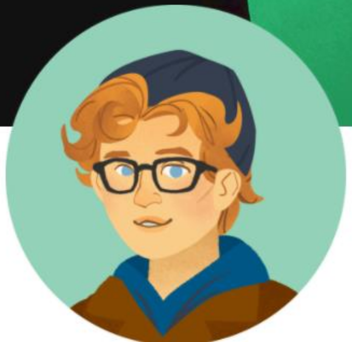

"Lea, maybe you could let Alex know when you're finished?"

NEXT

Scenarios

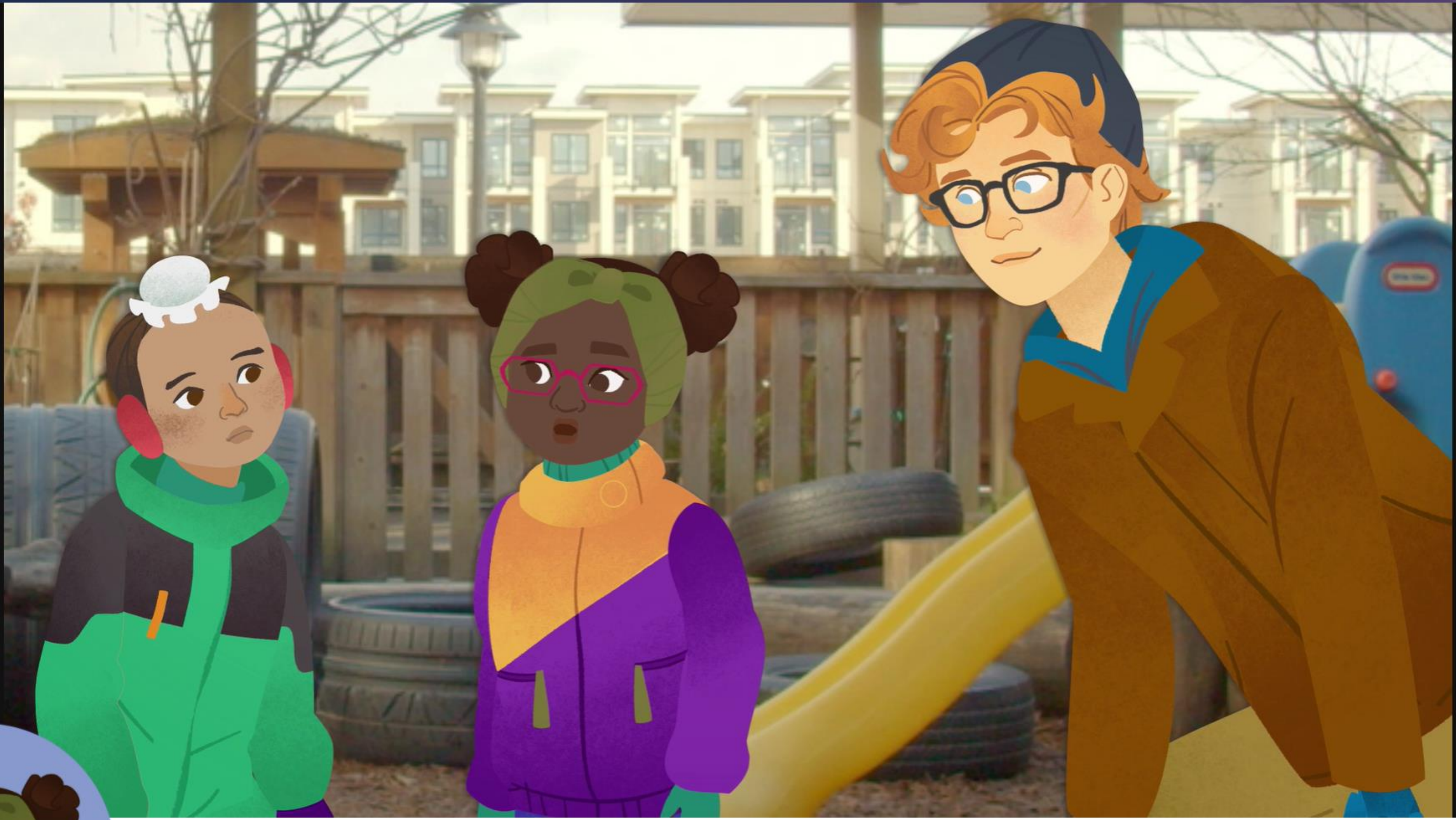

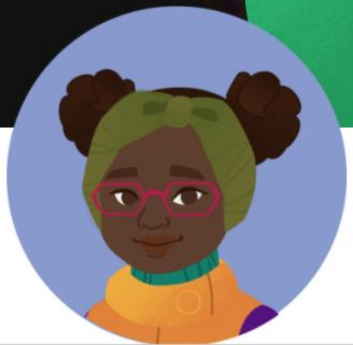

"I'm digging. I don't know when I'll be done."

NEXT ▶

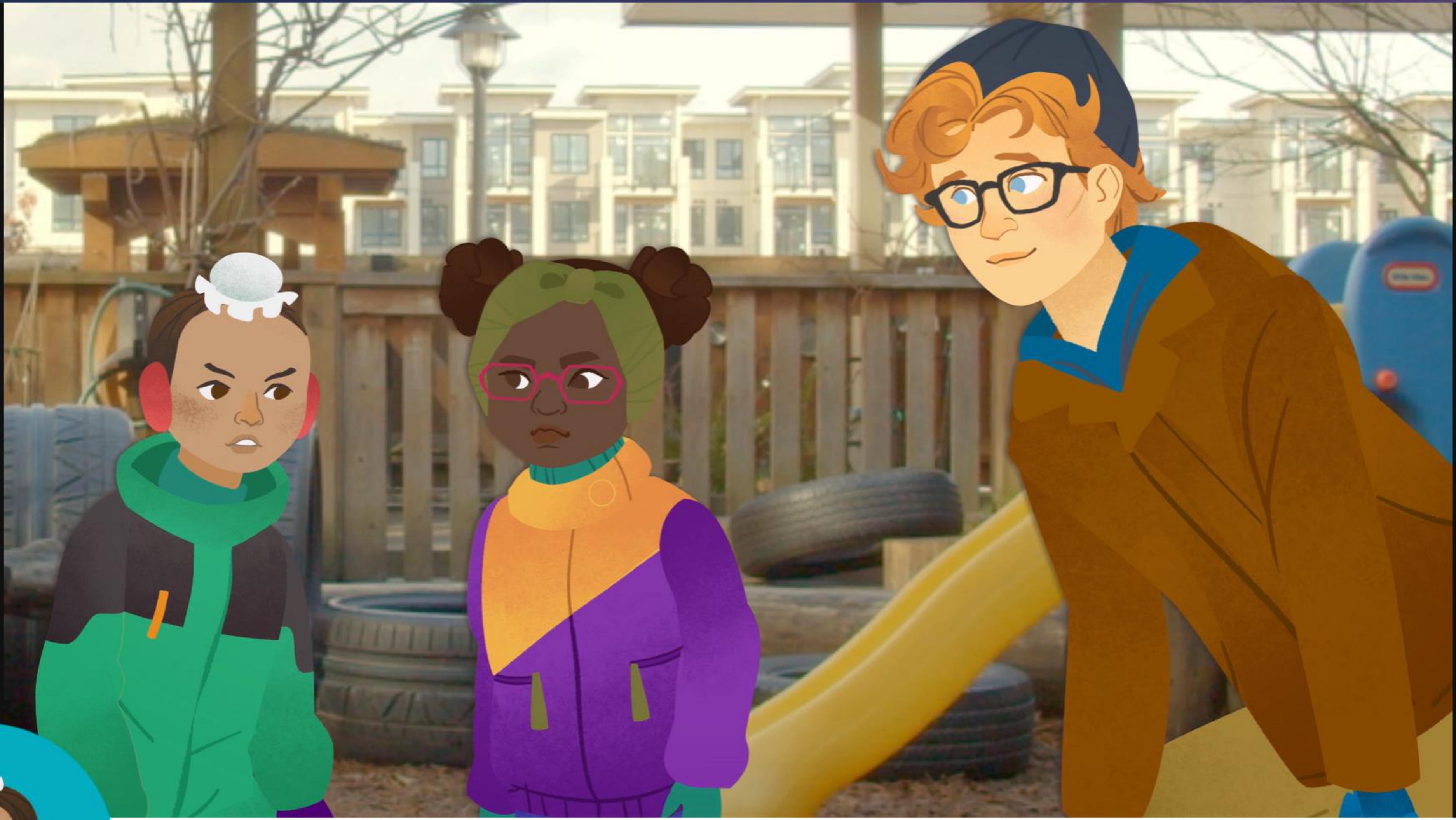

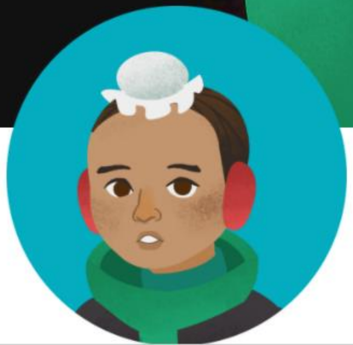

"That's not fair! I want to use the shovel. When's it my turn?"

NEXT ▶

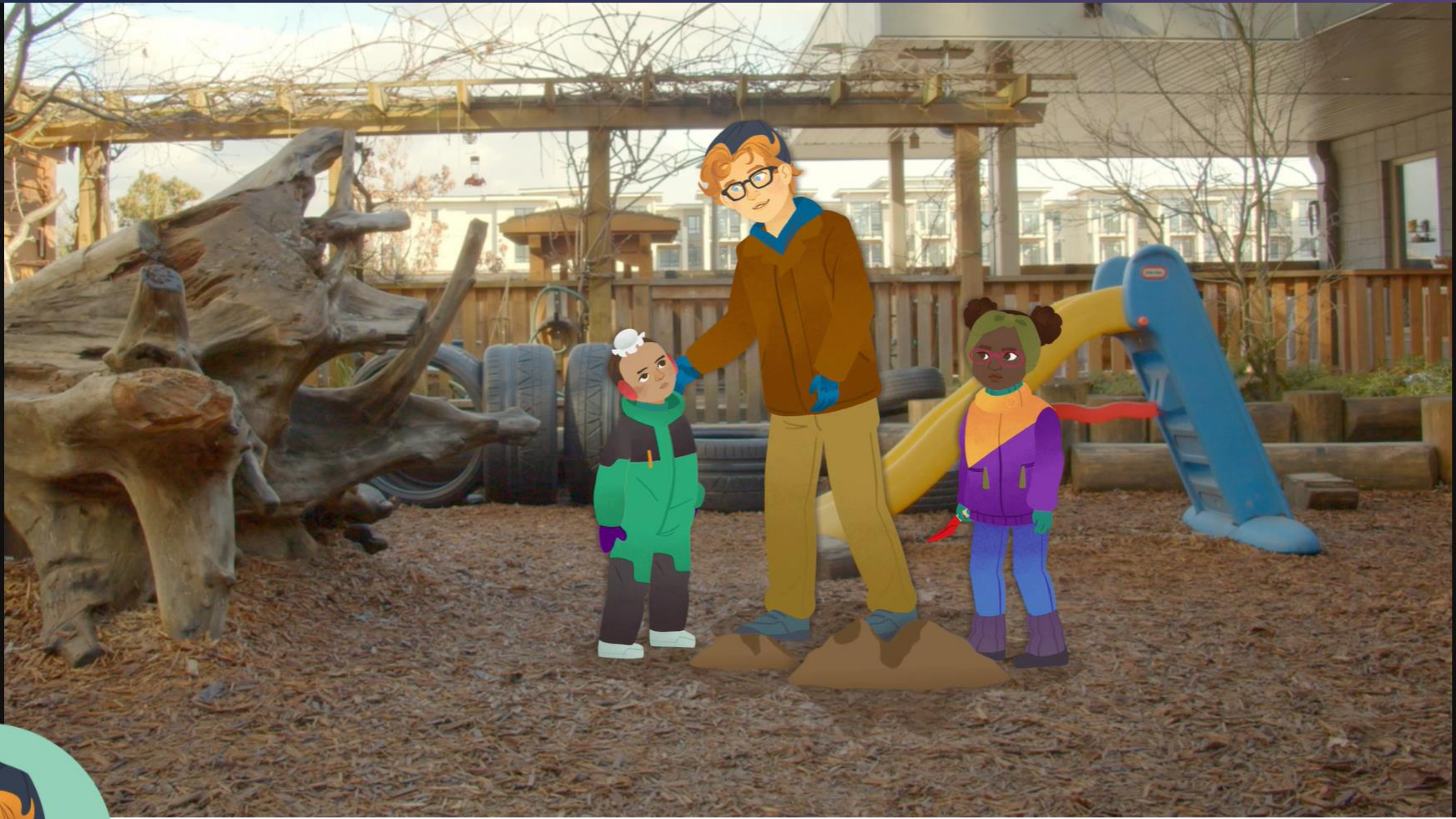

Scenarios

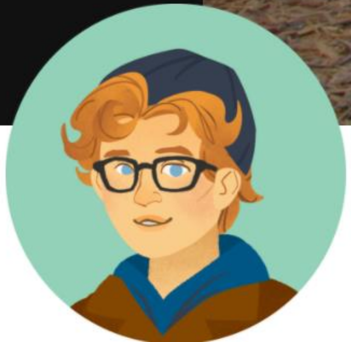

"Lea isn't finished yet. Is there something else that you could use? Where do we keep the shovels?"

NEXT

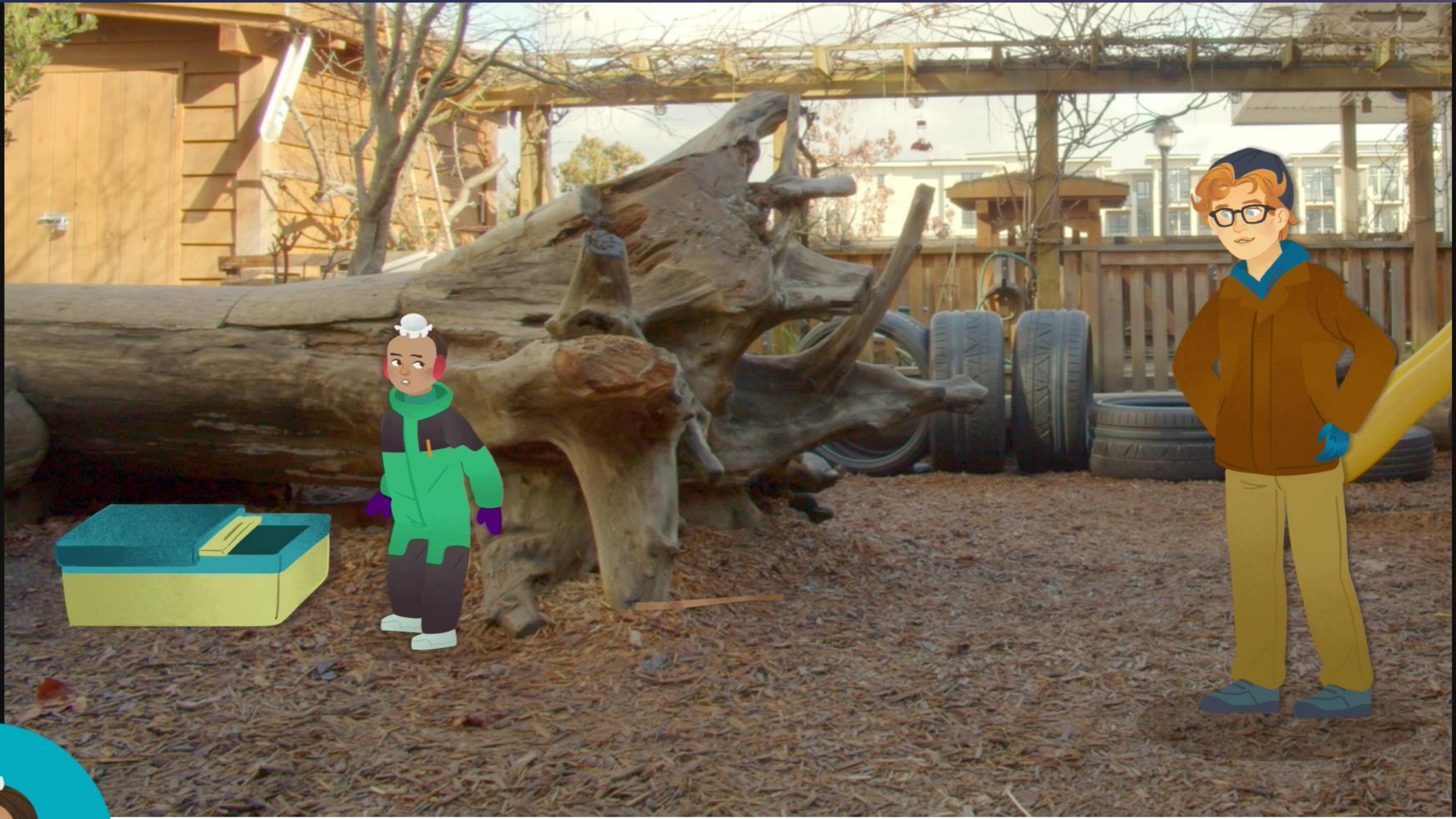

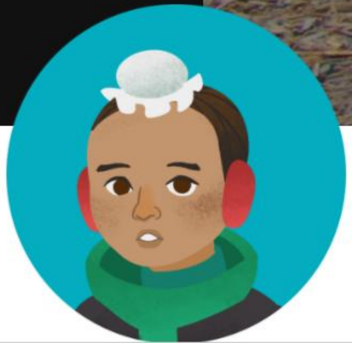

"There are no more shovels!"

NEXT

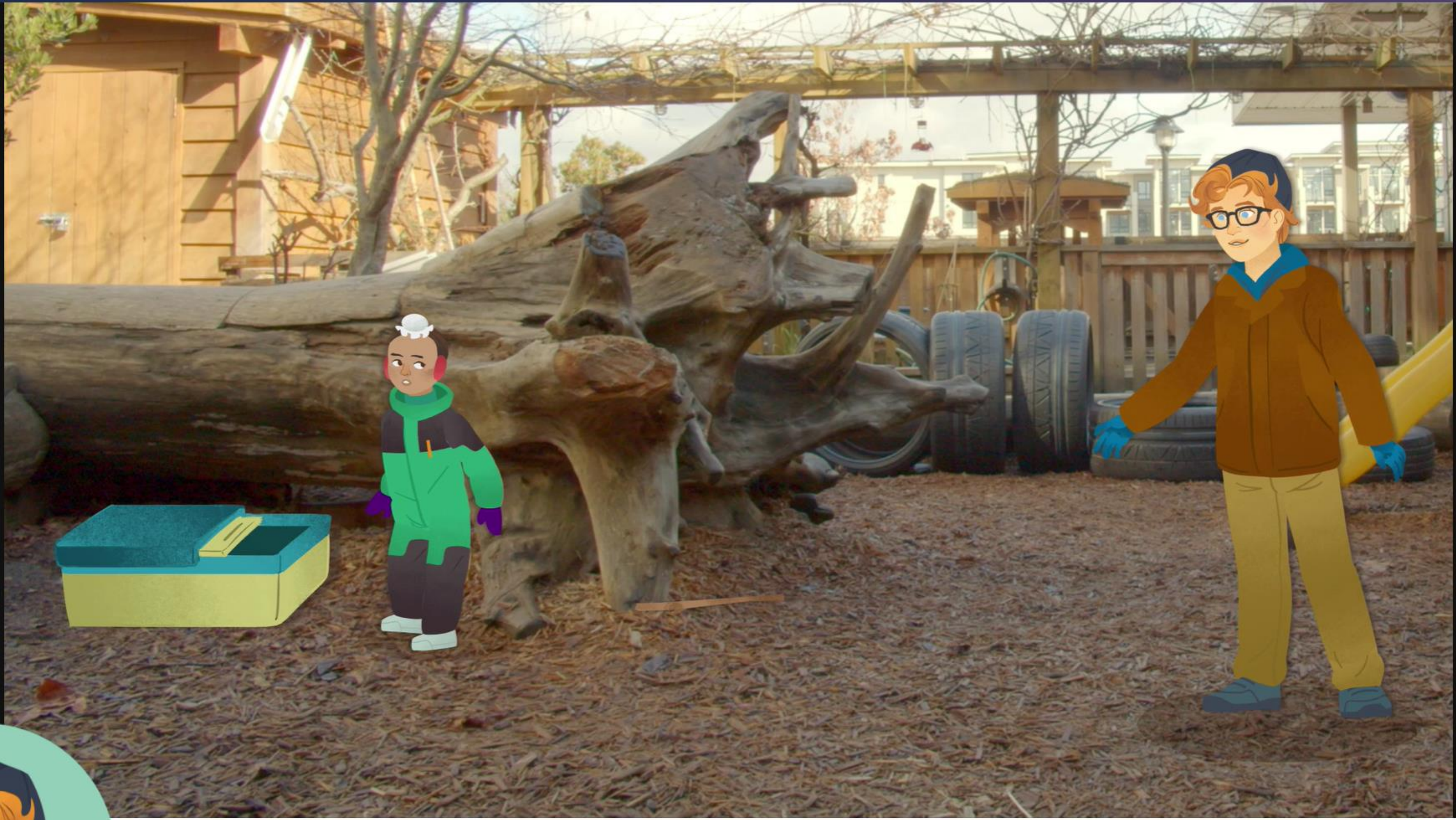

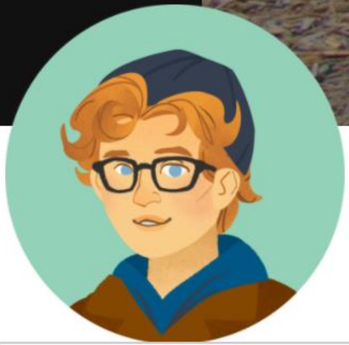

"Let's take a look and see if we can find something else you can use."

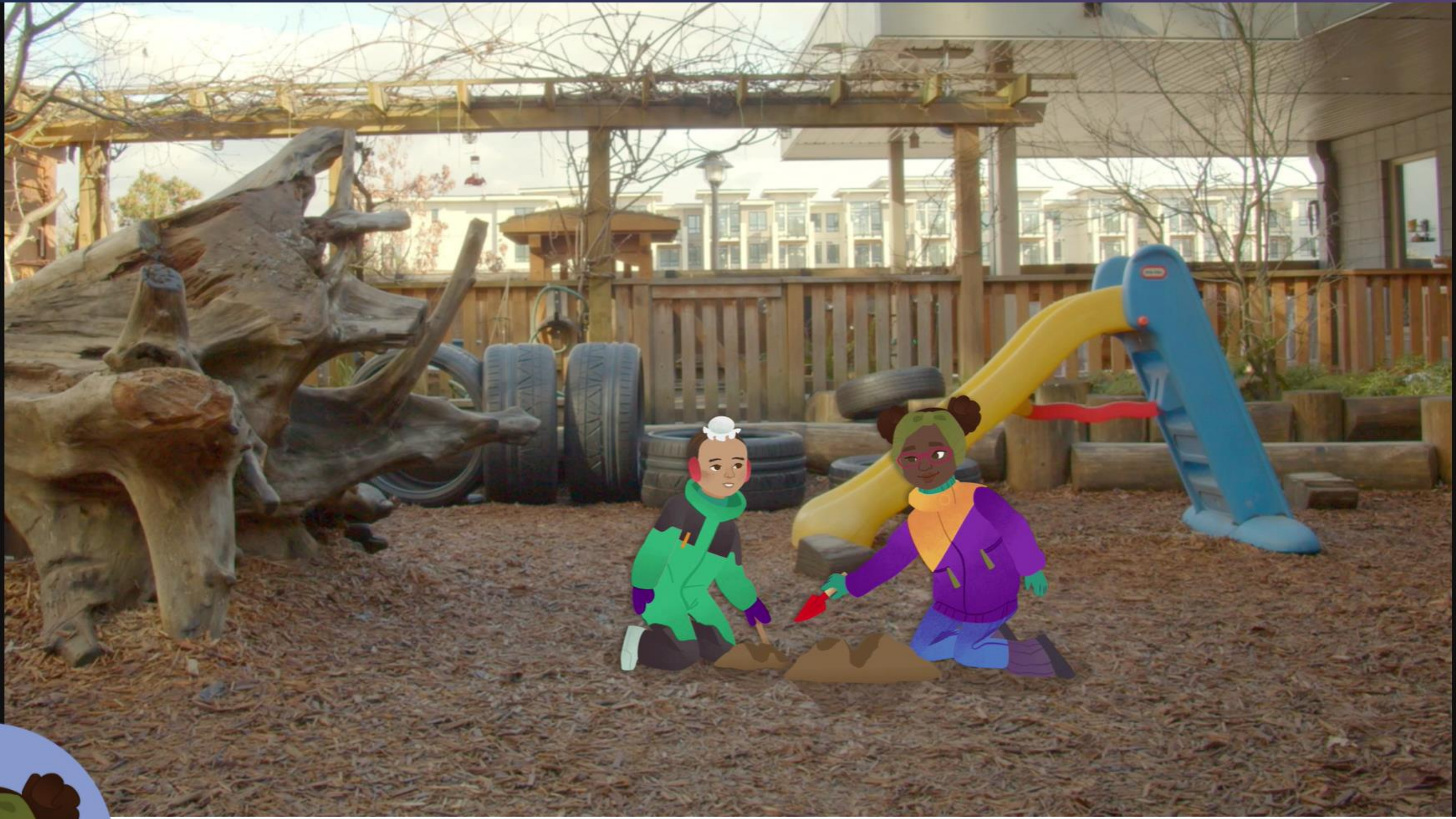

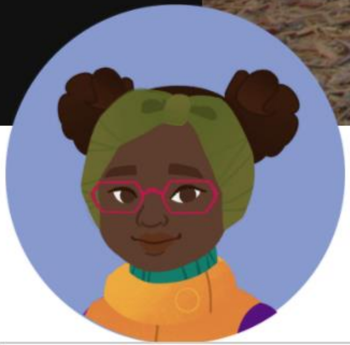

"I'm done with the shovel now. You can have it."

NEXT ▶

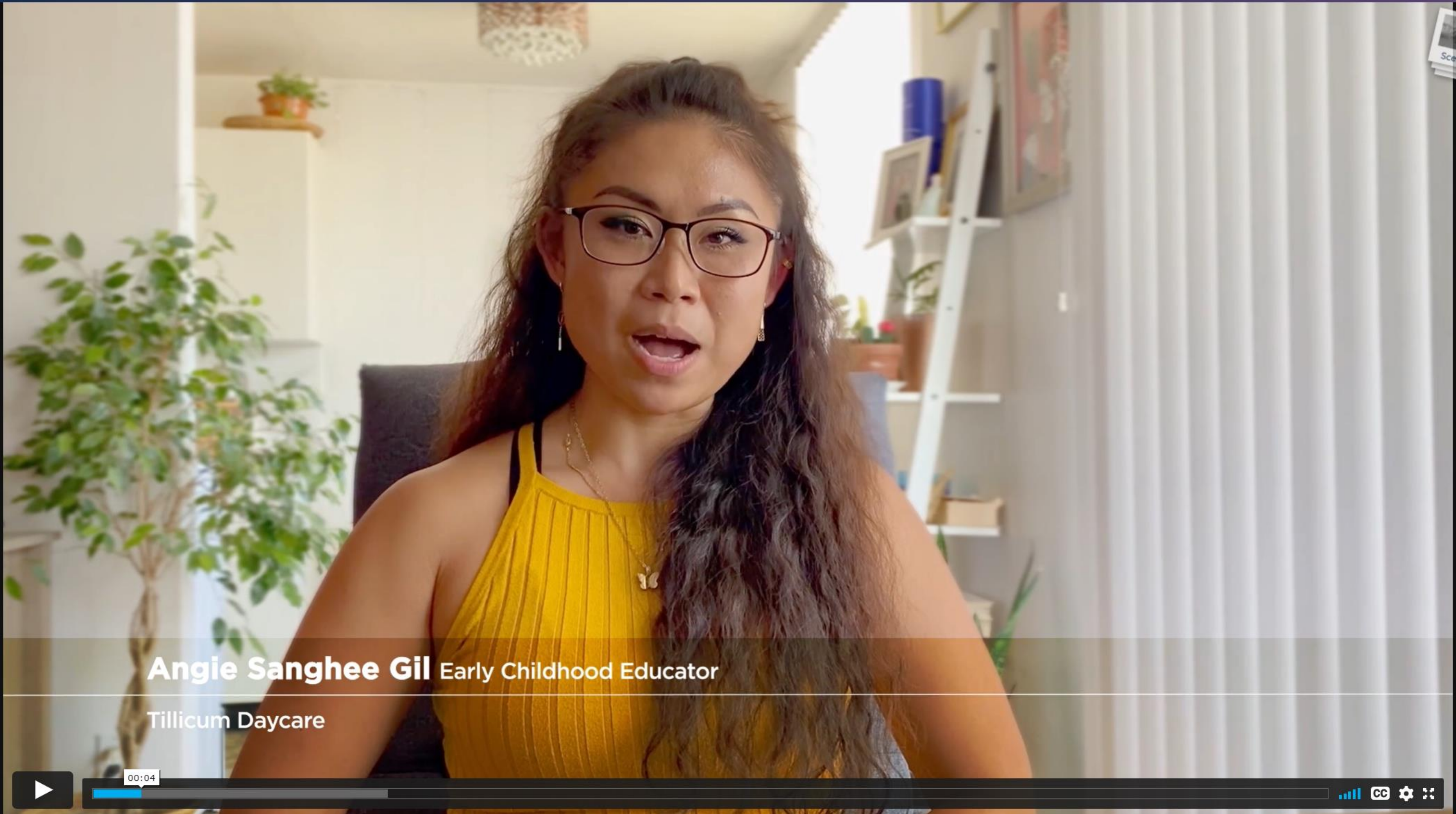
Scenarios
Angie Sanghee Gil Early Childhood Educator
Tillicum Daycare
00:04

What's the most important thing you learned?

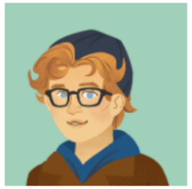

I learned...

Here are some examples if you get stuck

- Conflicts are caused by a struggle over power, rather than materials.
- Focus on creating a norm that all materials belong to everyone and sharing requires turn-taking, patience and consideration.
- Rather than forcing children to share, I can support children in resolving their own conflicts, help them build their negotiation skills, or help them identify other materials that they can use instead.
- I don't have to step into every conflict, but rather give children time to sort it out on their own.

Save and Continue

Chapter 2:

Scenarios

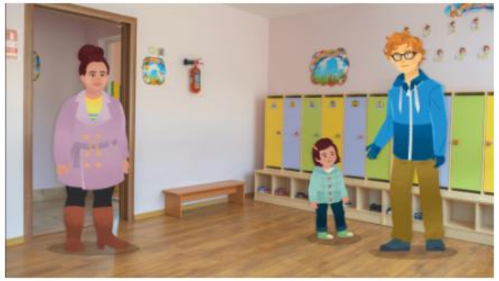

Communicating with Parents/Caregivers

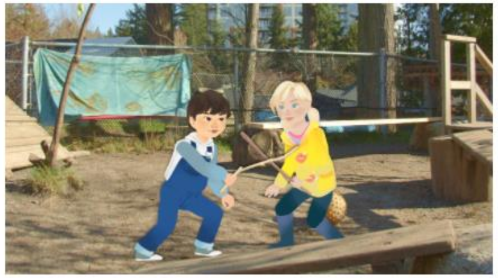

Rough and Tumble Play

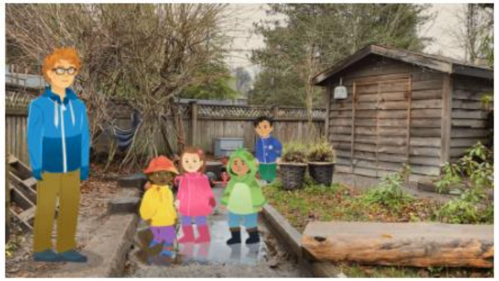

Play at Speed

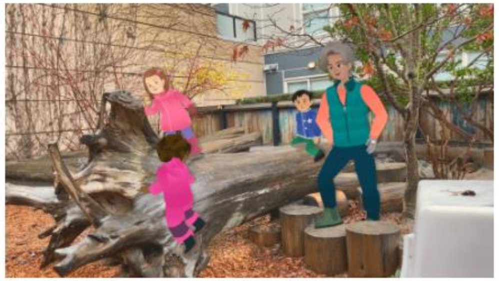

Play at Heights

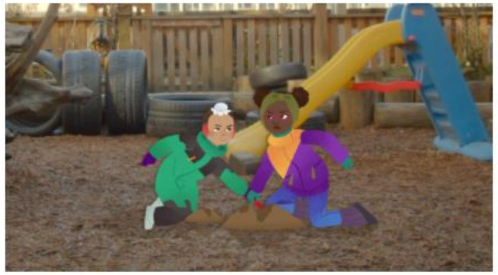

Conflict Resolution

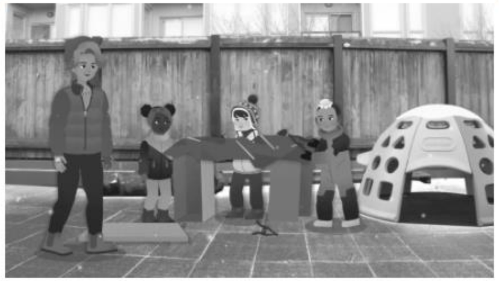

Play with Loose Parts

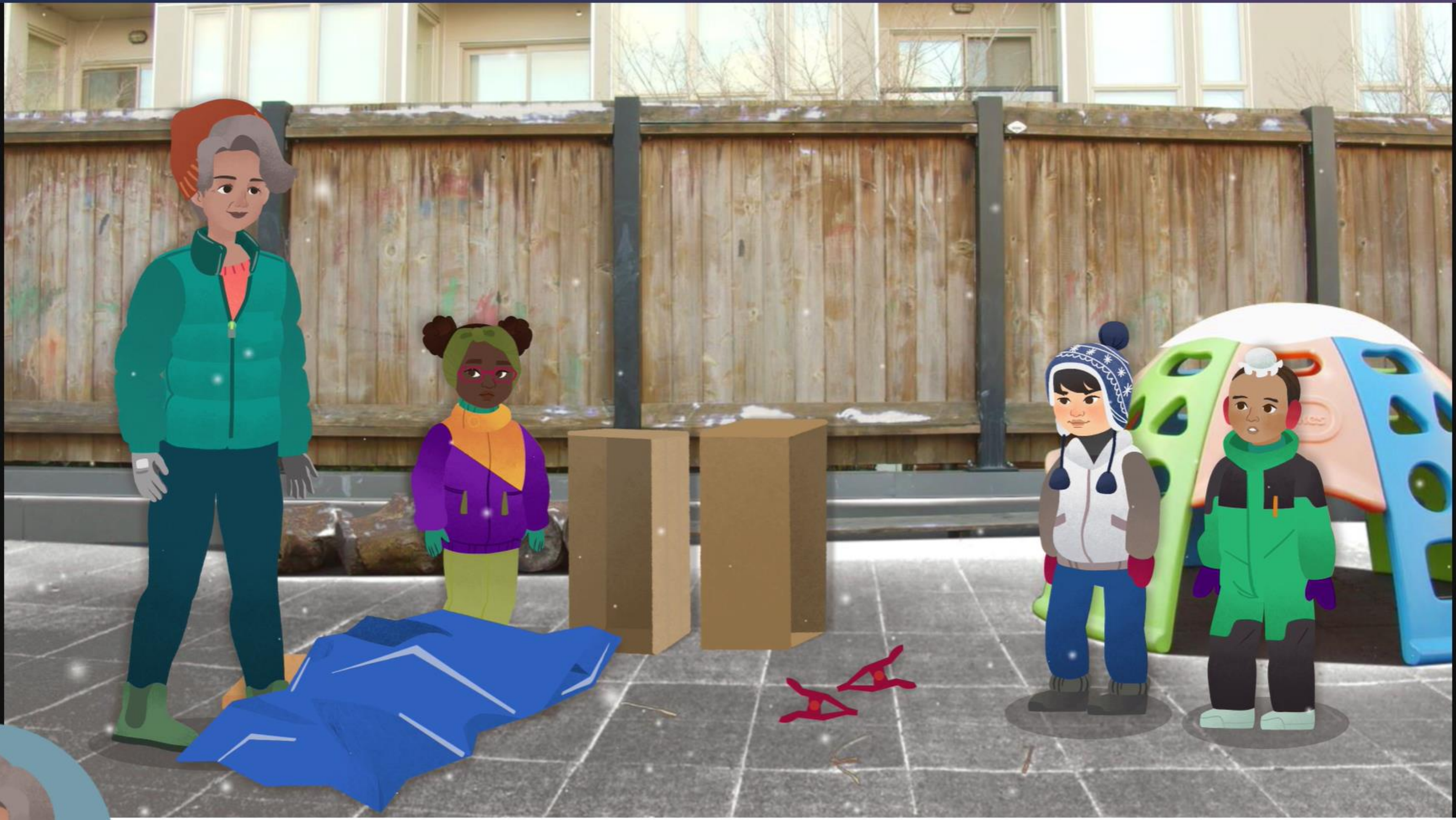

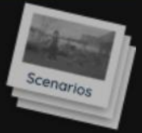

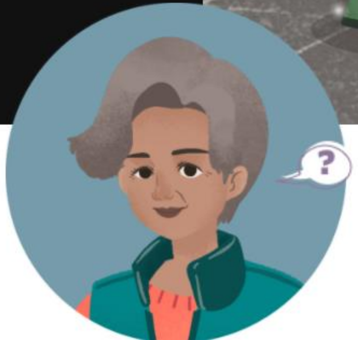

"What should I do?"

- Support the children's risk management as they build
- Take over to show the children how to build safely

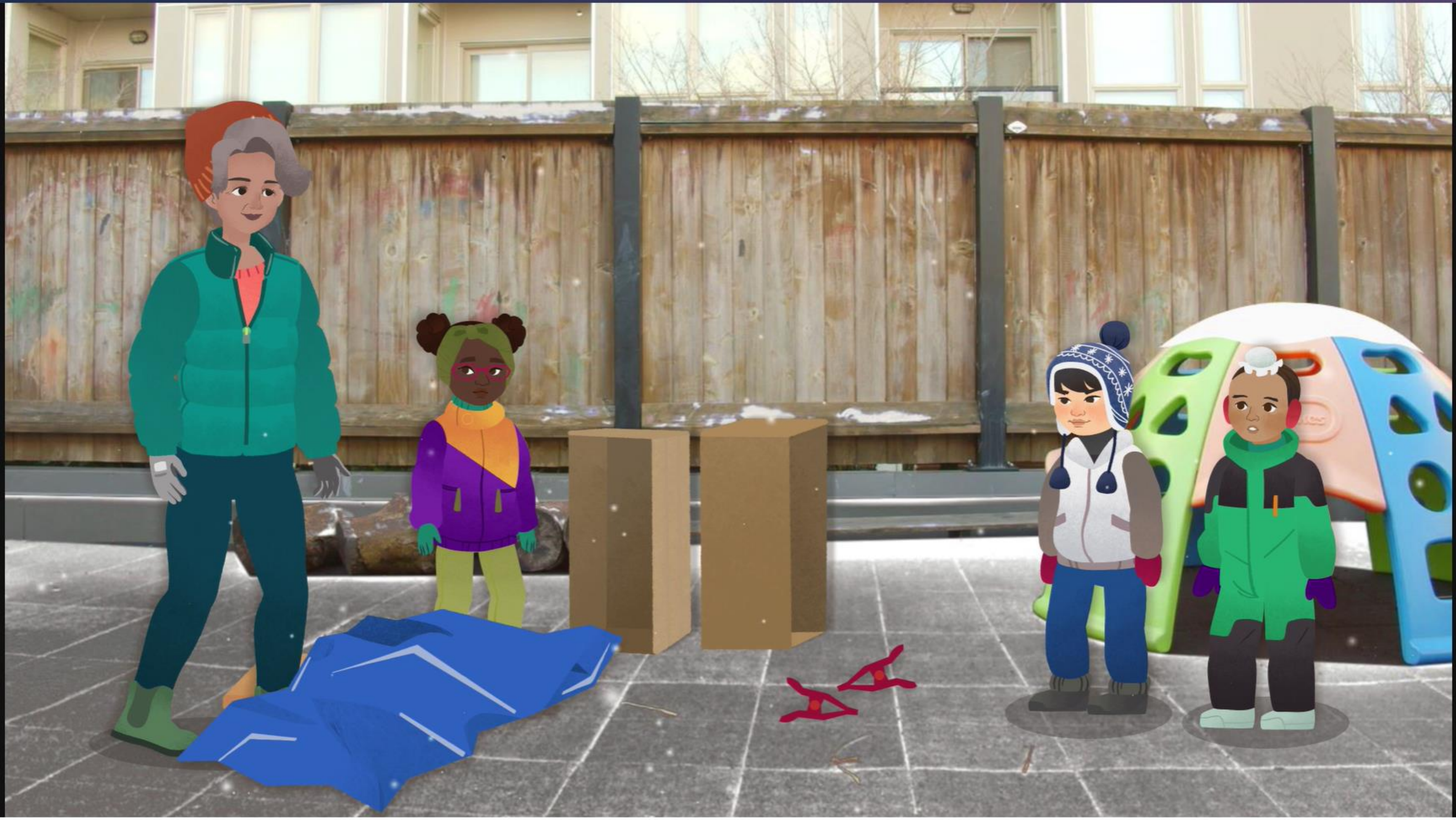

"That looks like fun! Let me help you put this together."

NEXT

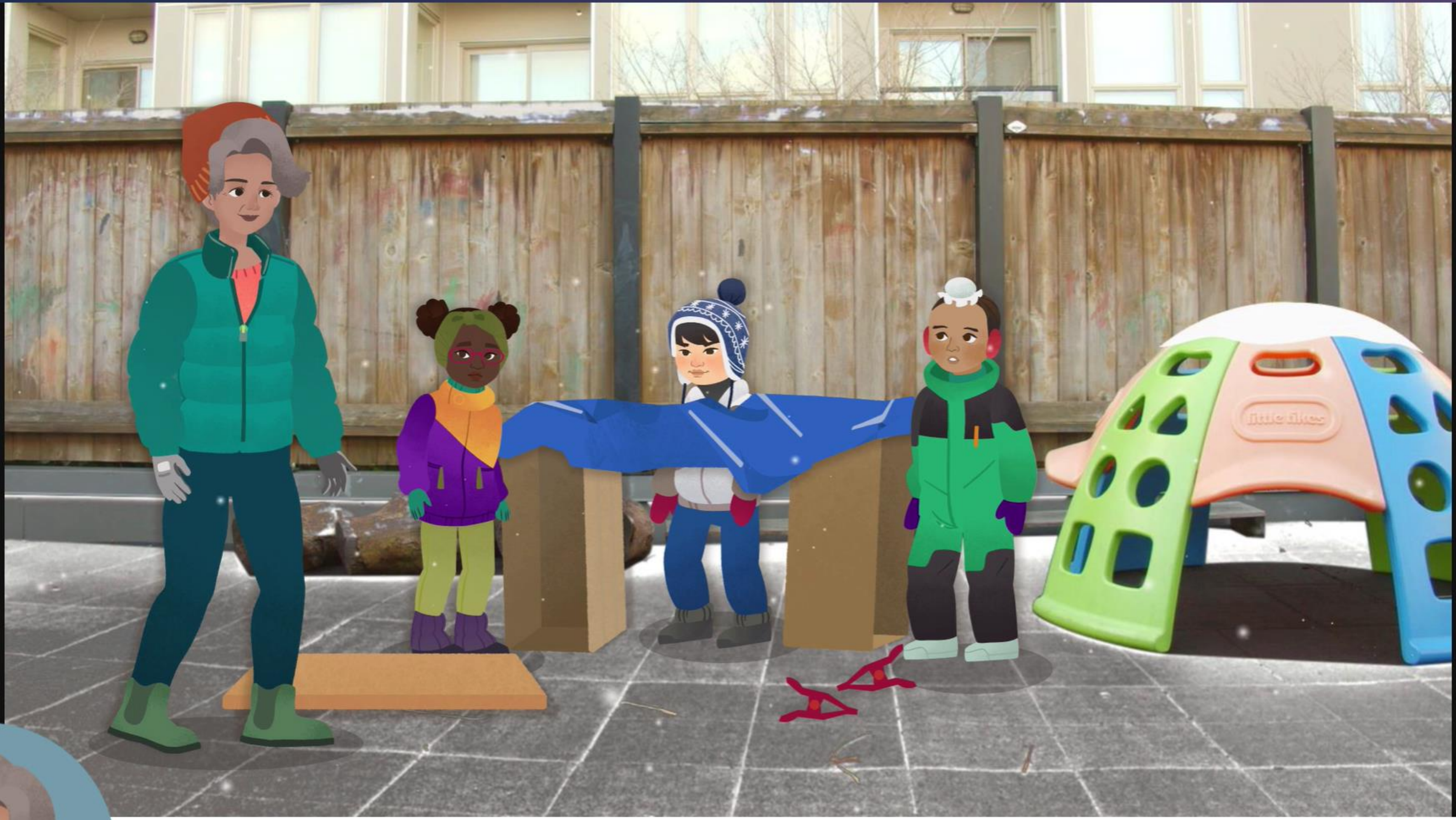

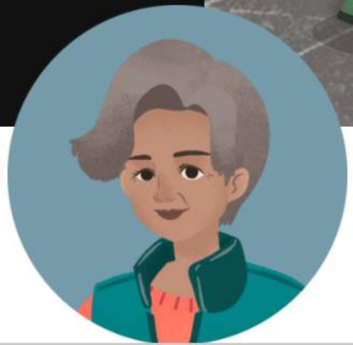

"You need to put these crates separate from each other, then take this tarp and put it over the top"

NEXT

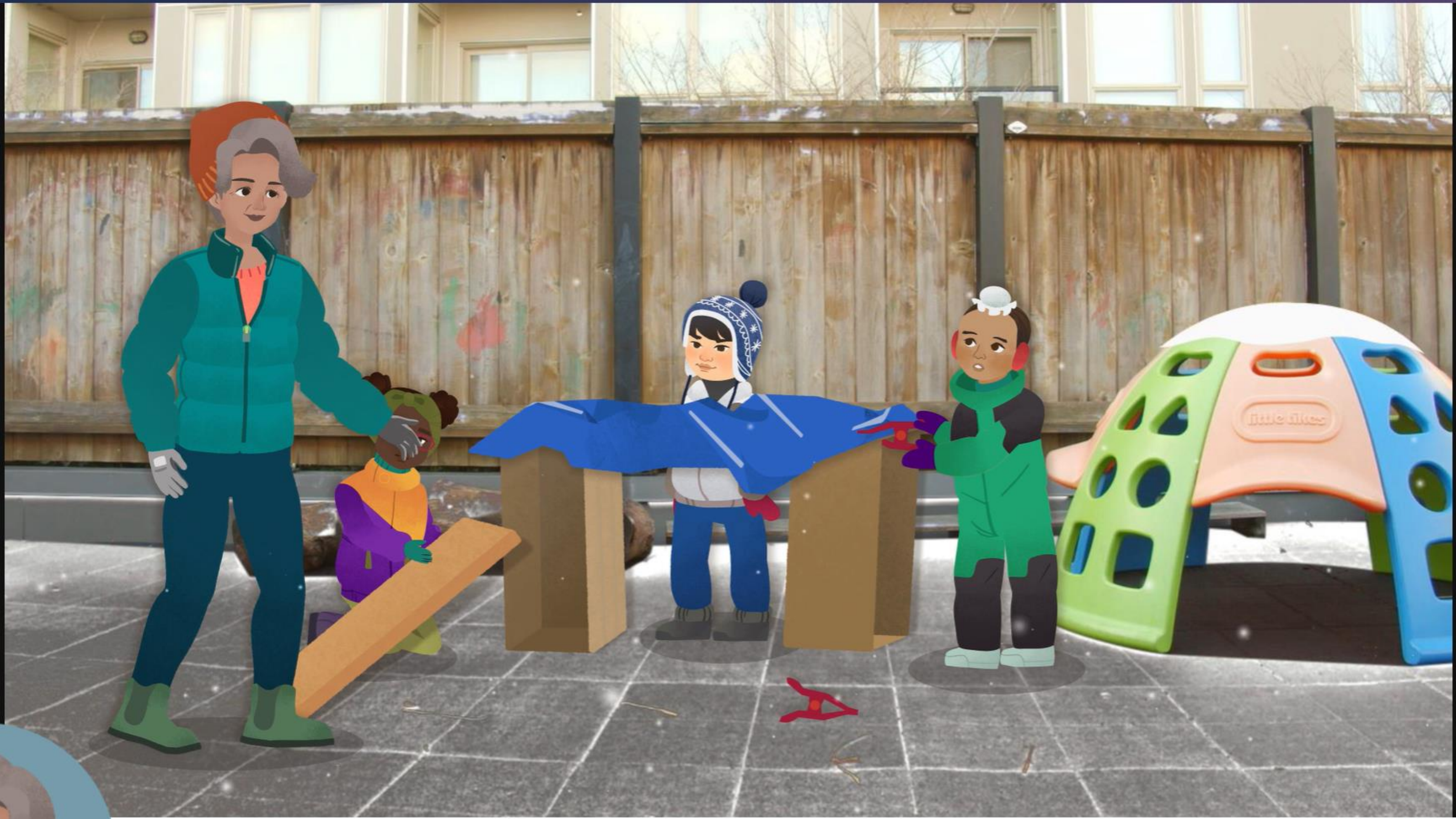

Scenarios

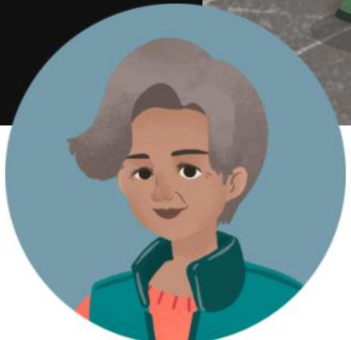

"Make sure to clip the tarp down"

NEXT ▶

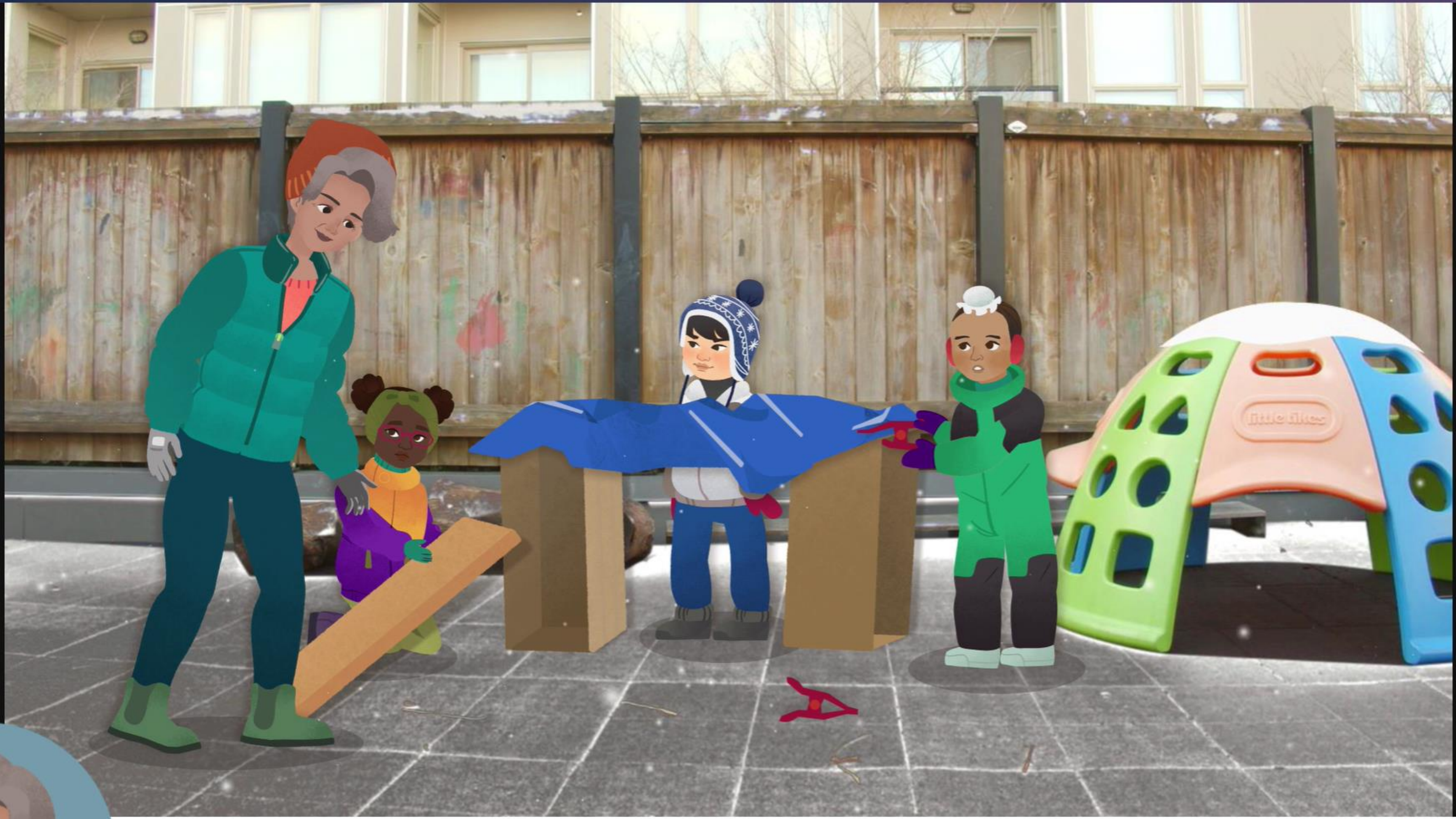

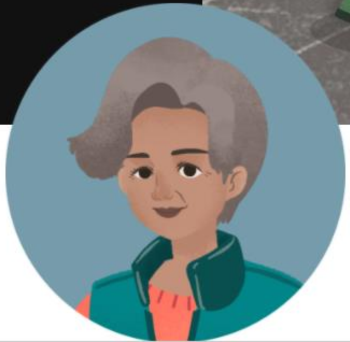

"Let me place that plank. It looks too heavy for you."

NEXT ▶

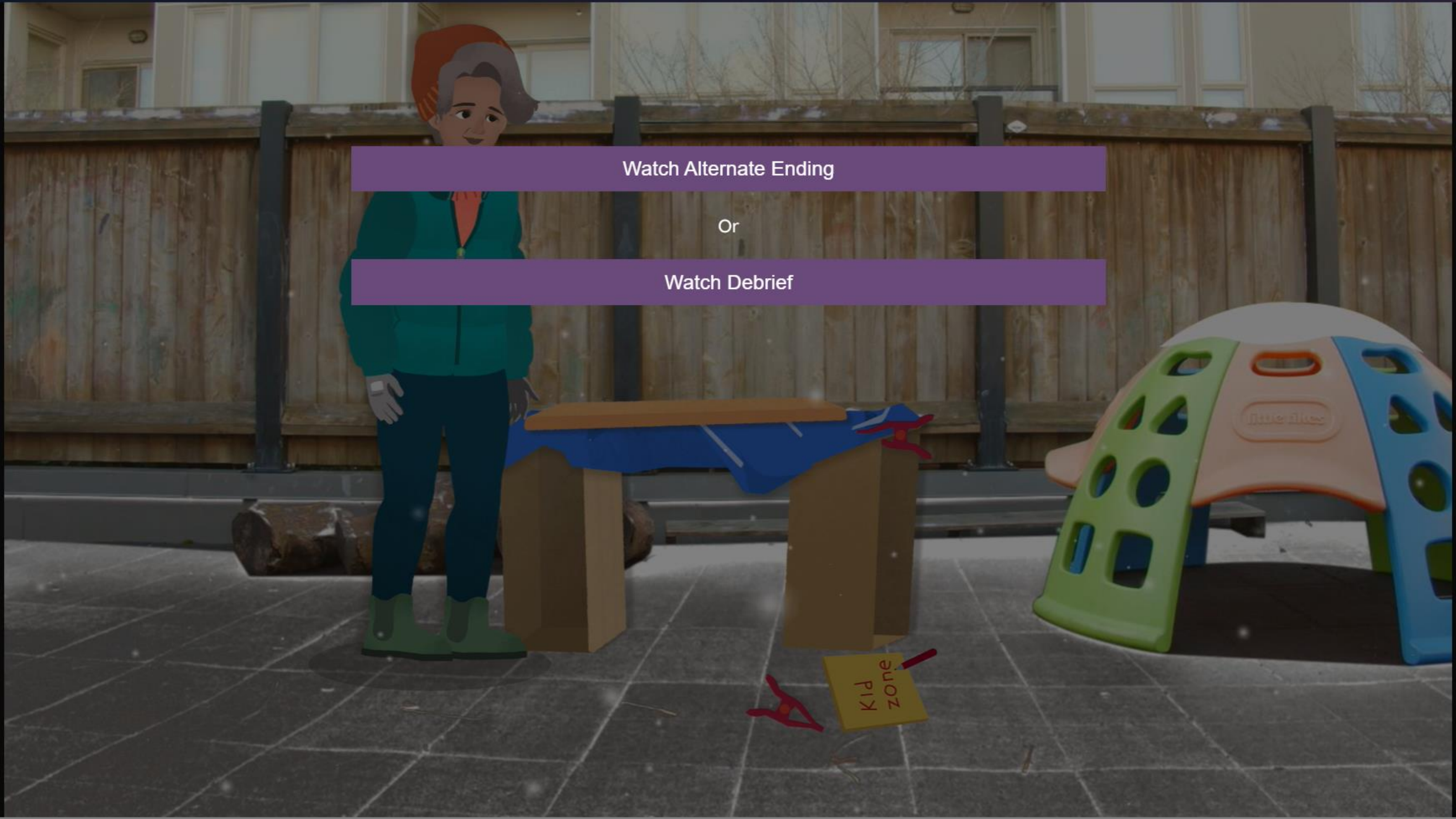

Watch Alternate Ending

Or

Watch Debrief

Scenarios

SKIP ▶

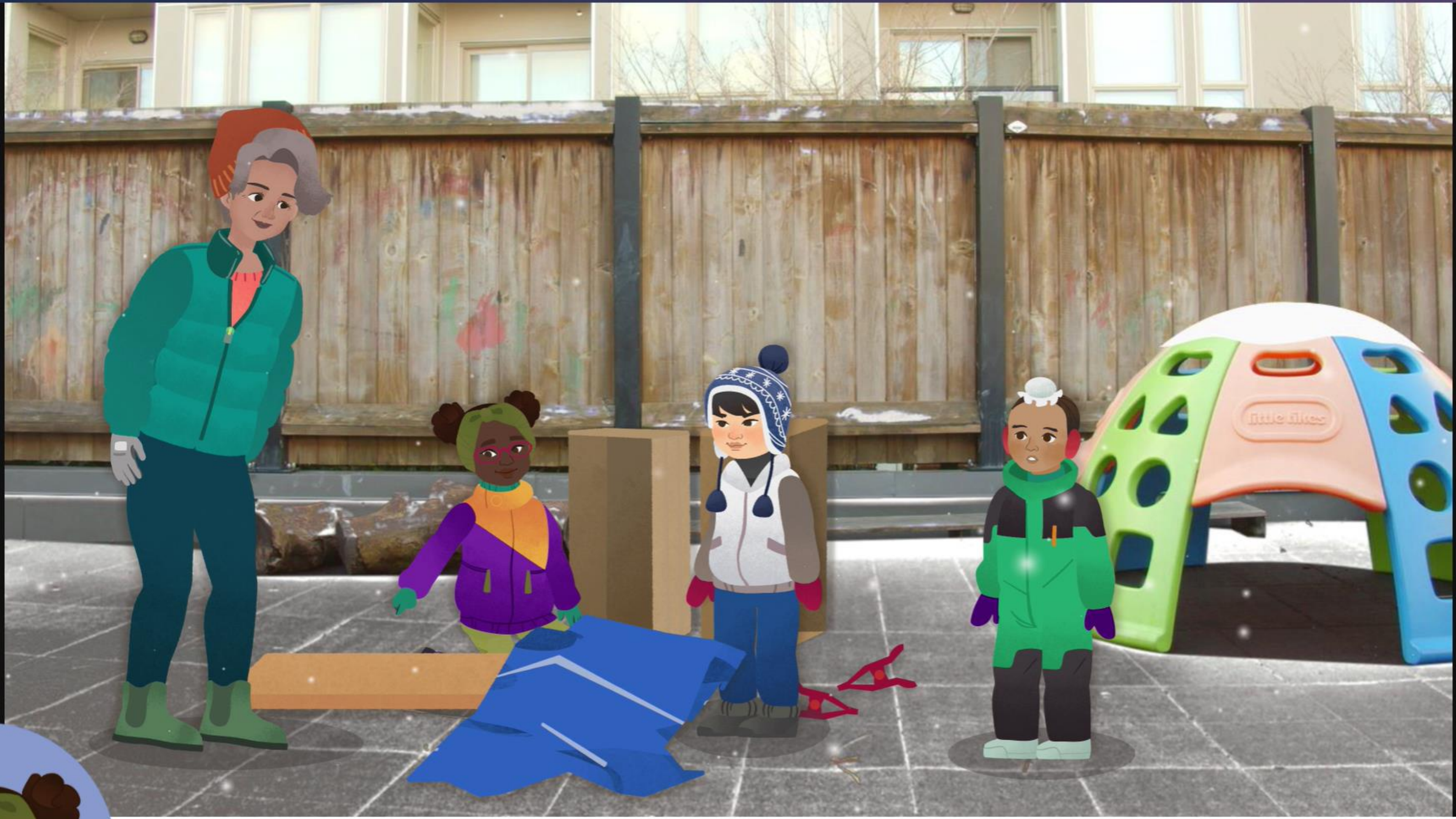

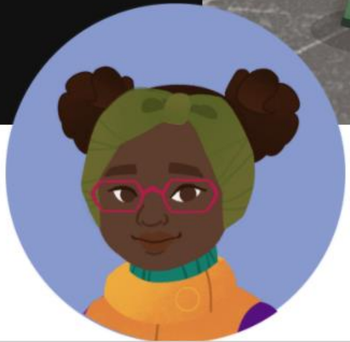

"We need to put this wood on top for a roof!"

NEXT ▶

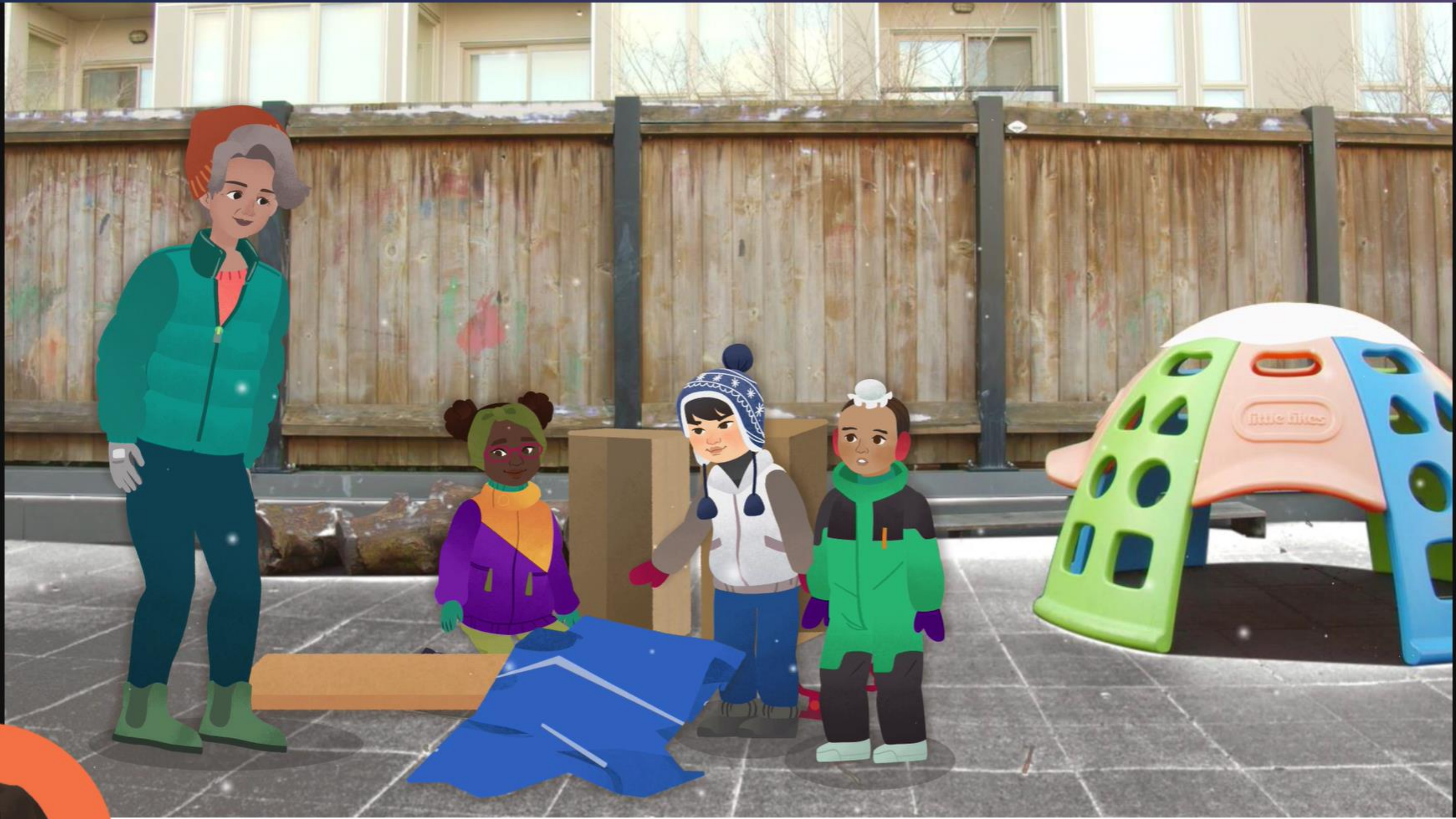

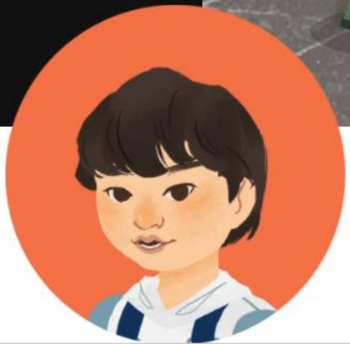

"Let's lift it together!"

NEXT ▶

Scenarios

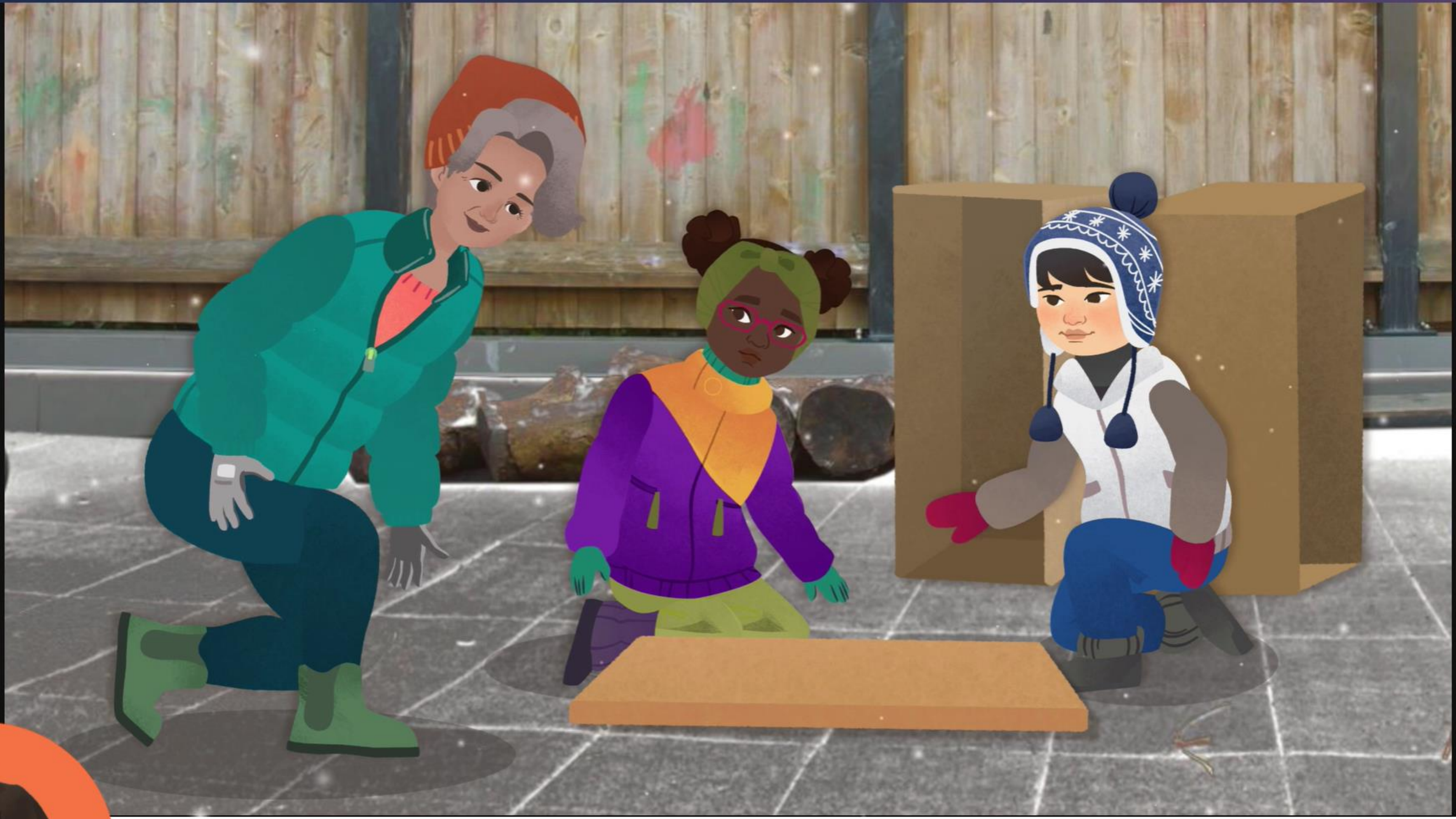

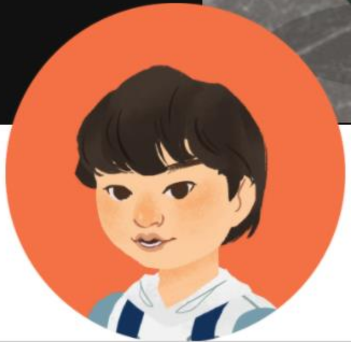

"It's too heavy! Can you help us?"

NEXT

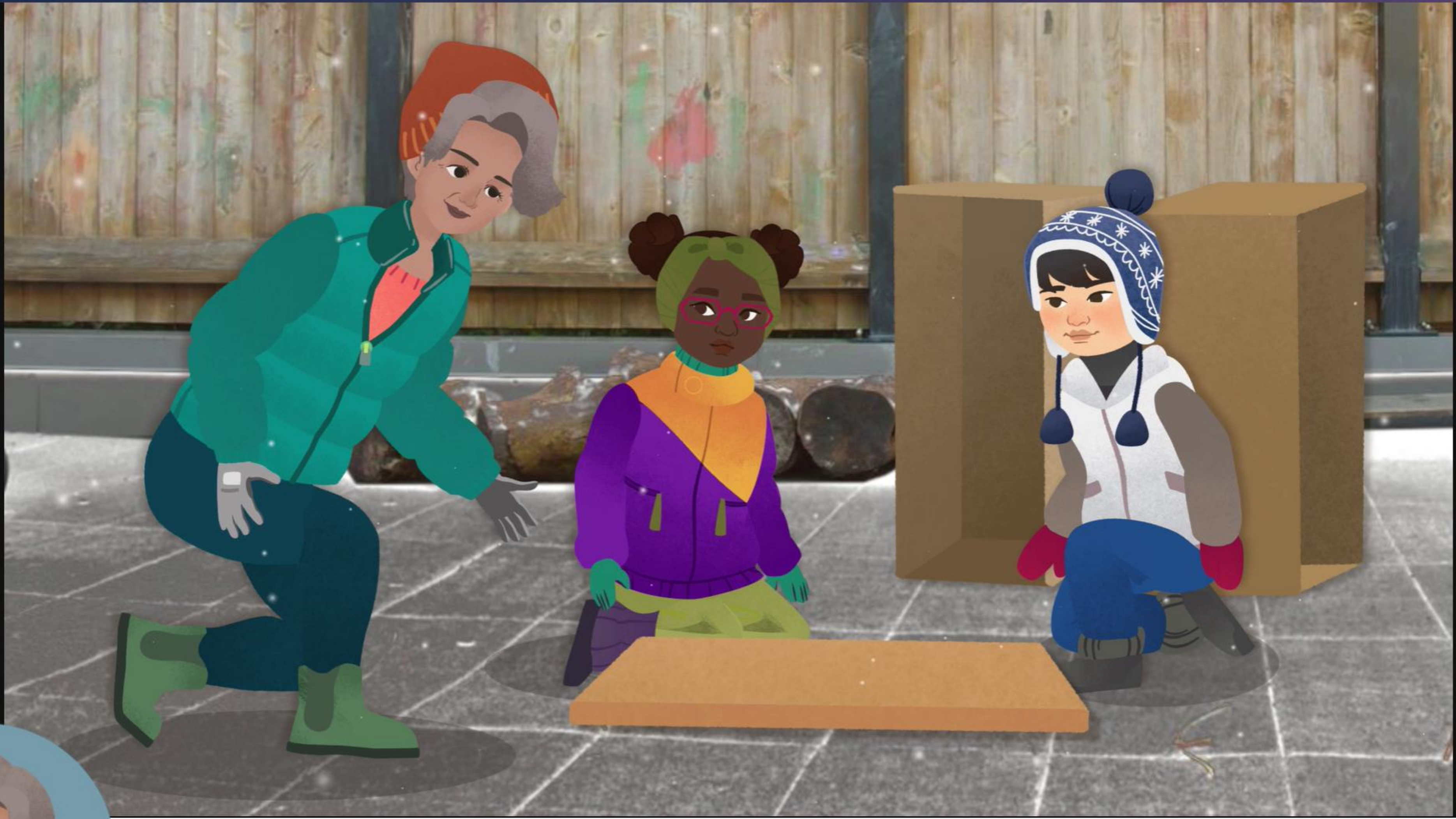

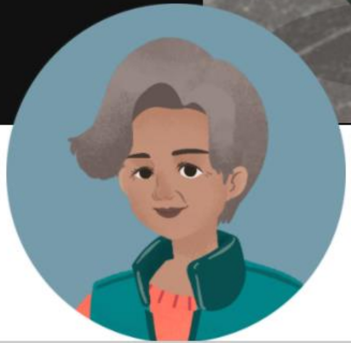

"What are you trying to do?"

NEXT

Take a risk, go play outside!

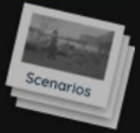

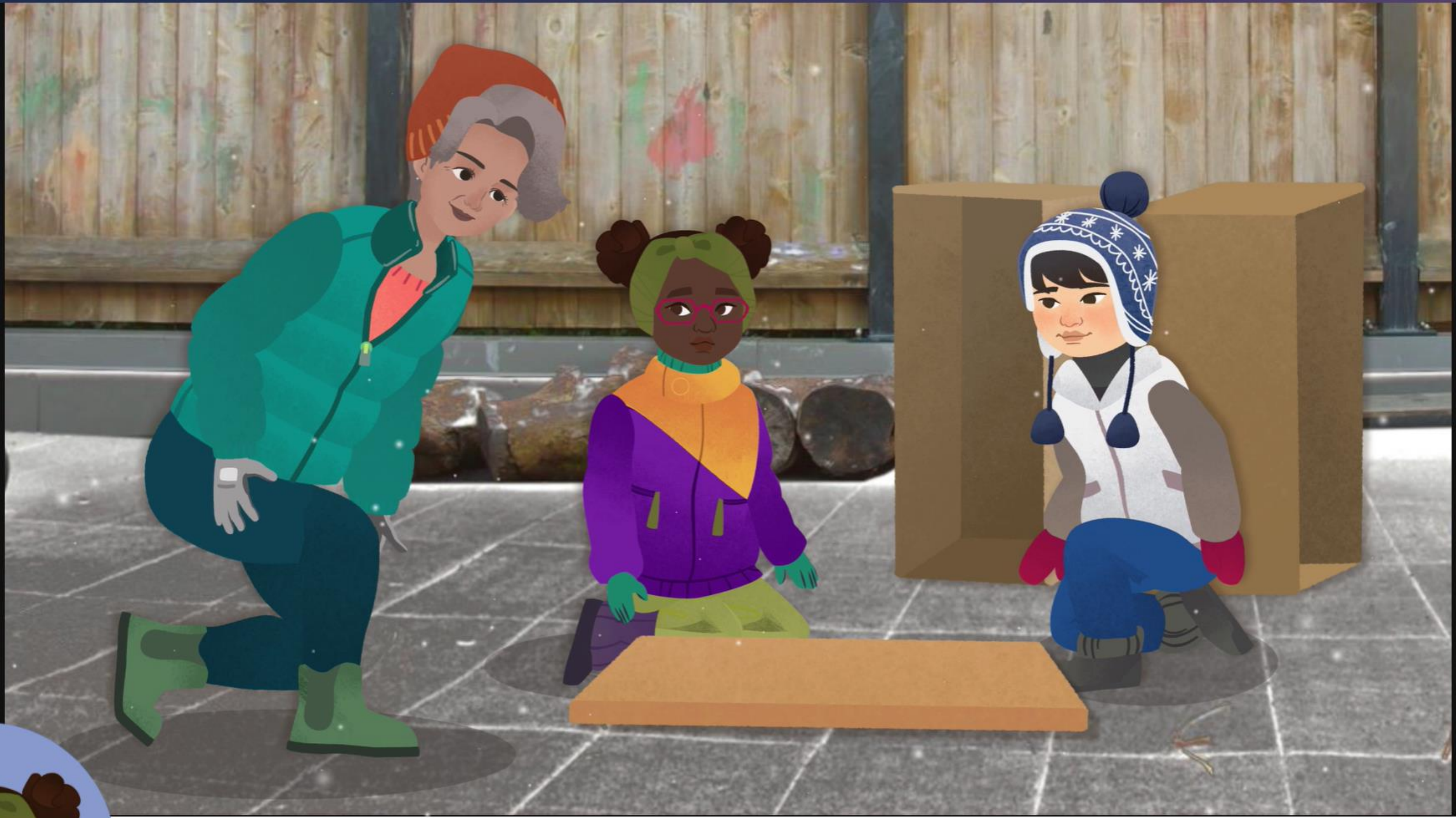

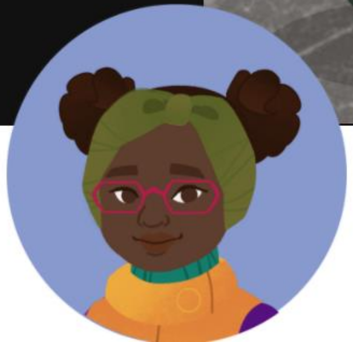

"We're trying to put a roof on our den"

NEXT

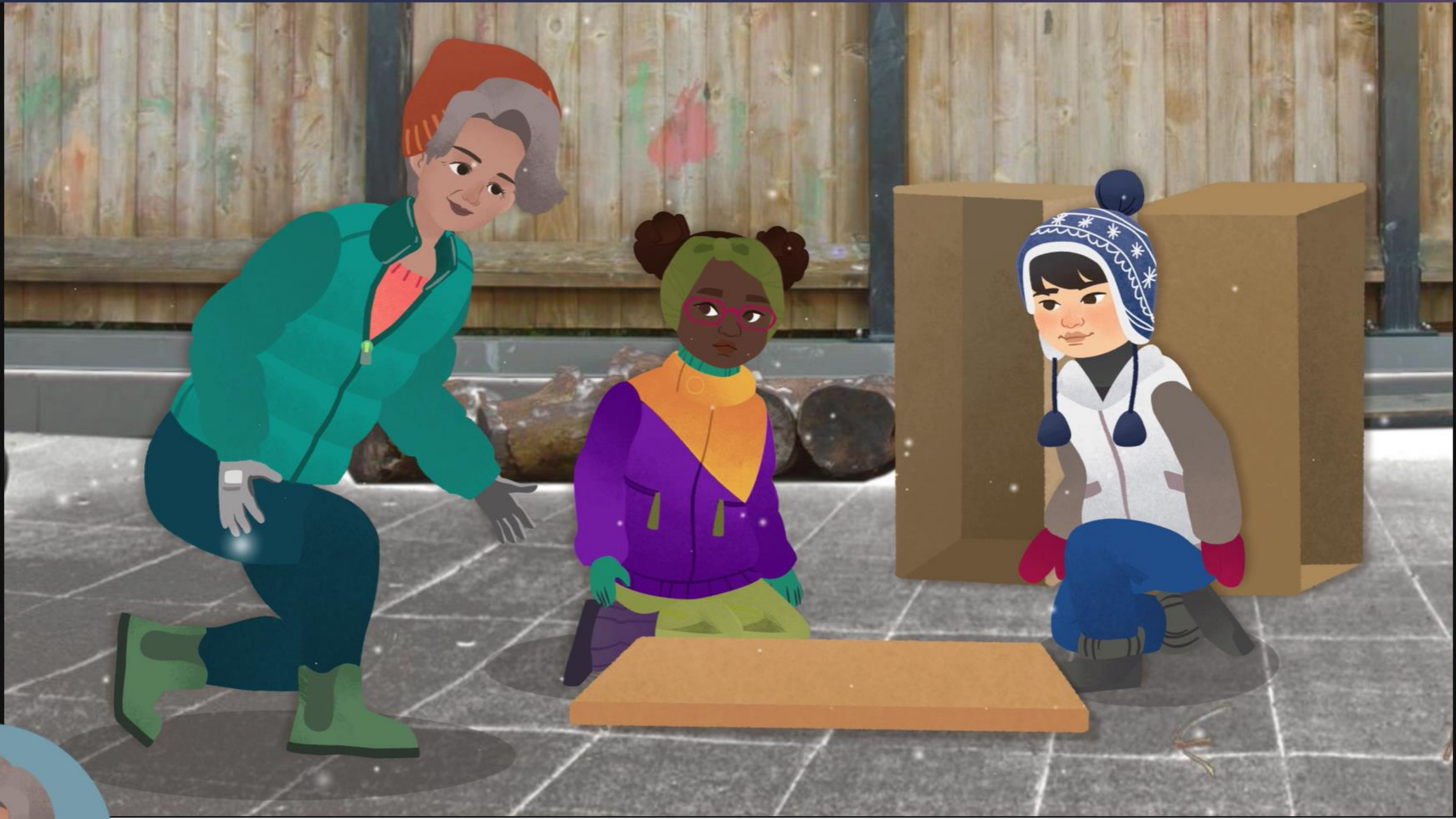

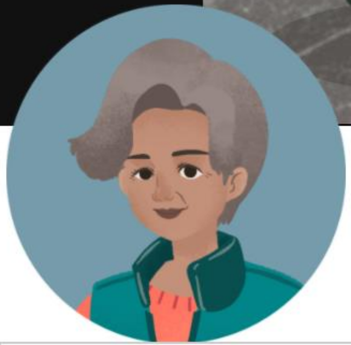

"Do you think those crates will be stable enough to hold that plank?"

NEXT

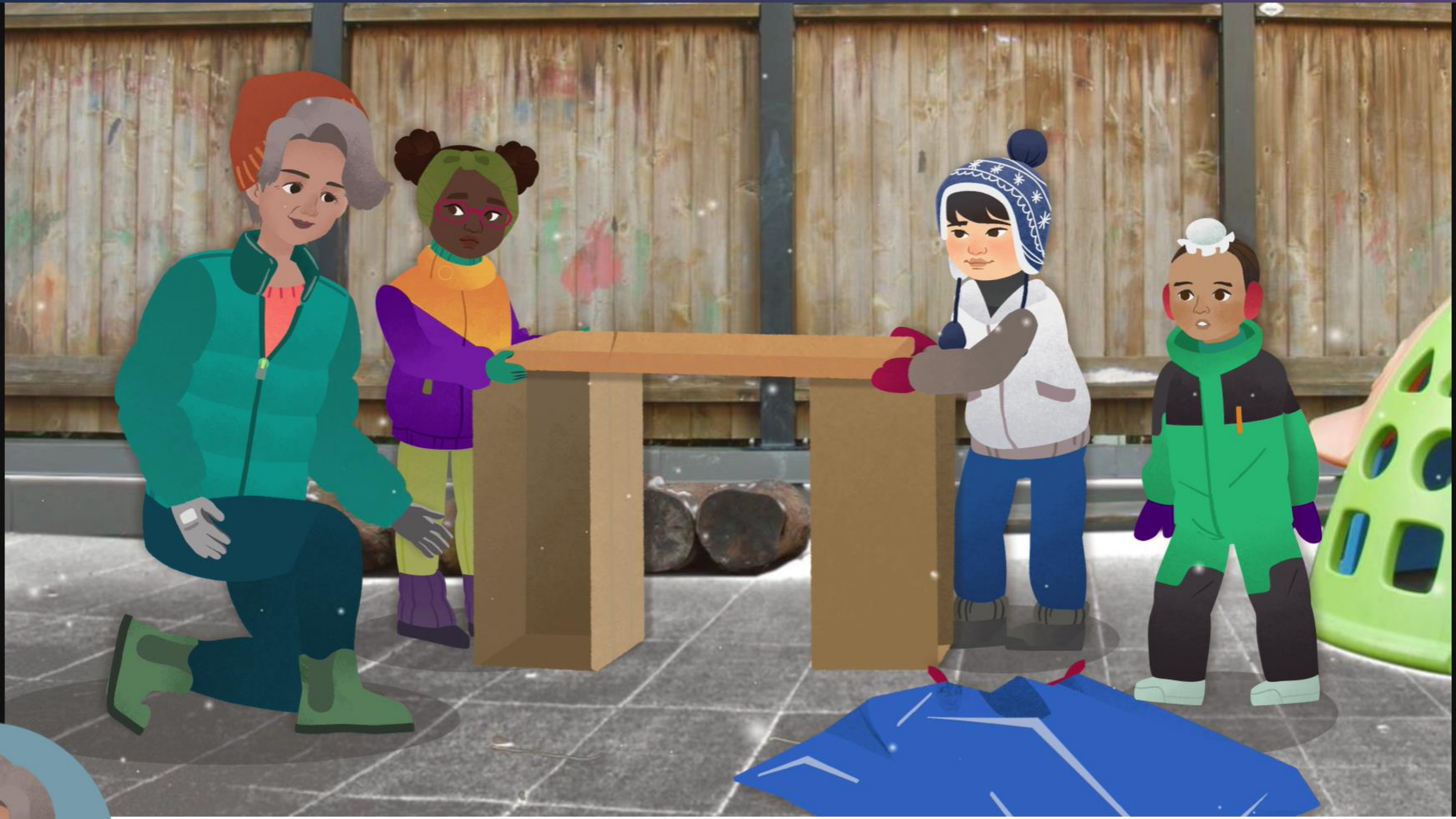

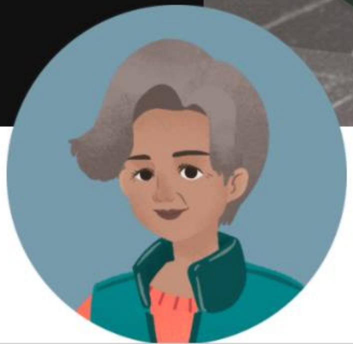

"What could happen if it's not stable enough?"

NEXT

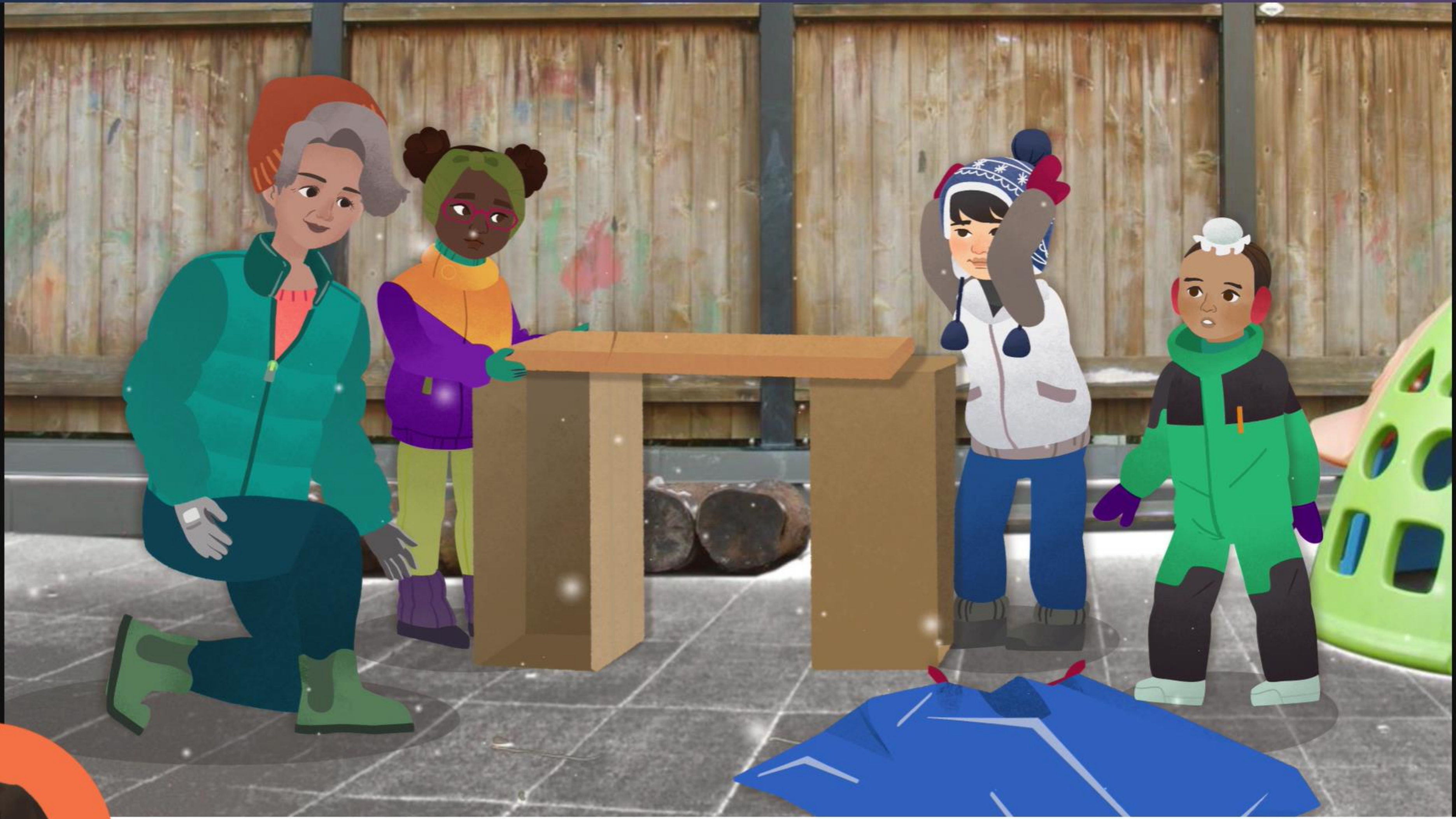

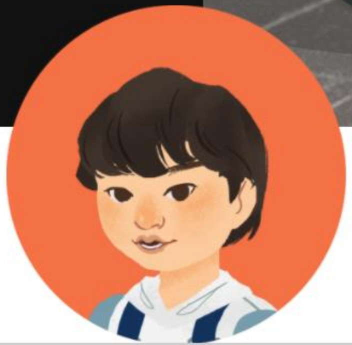

"It could fall on our heads!"

NEXT

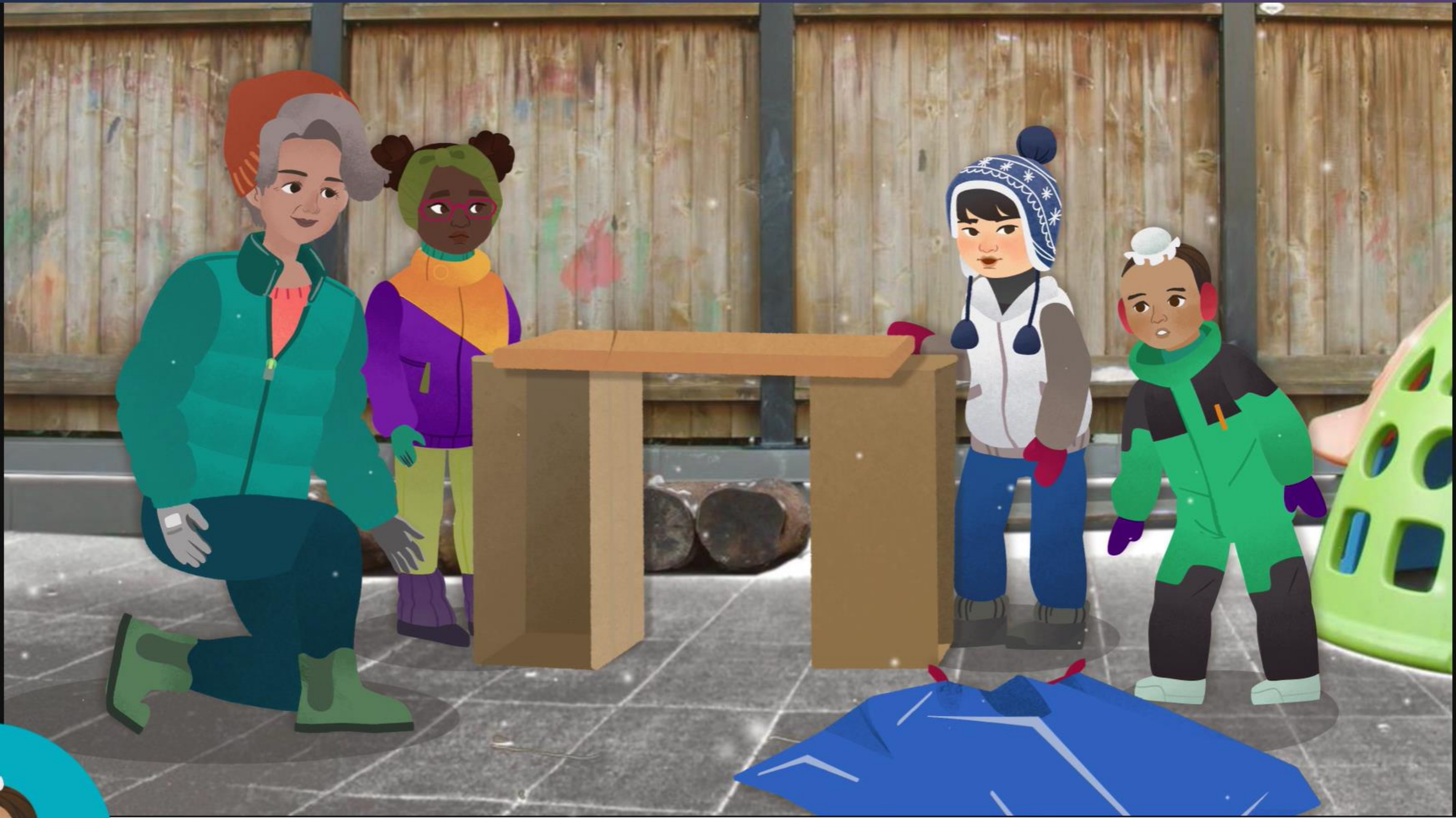

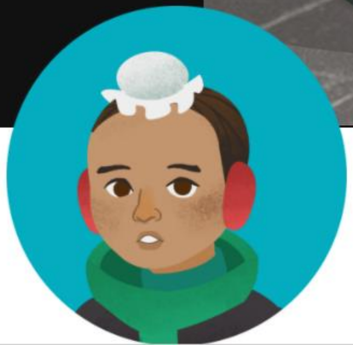

"What if we use the tarp as a roof instead?"

NEXT ▶

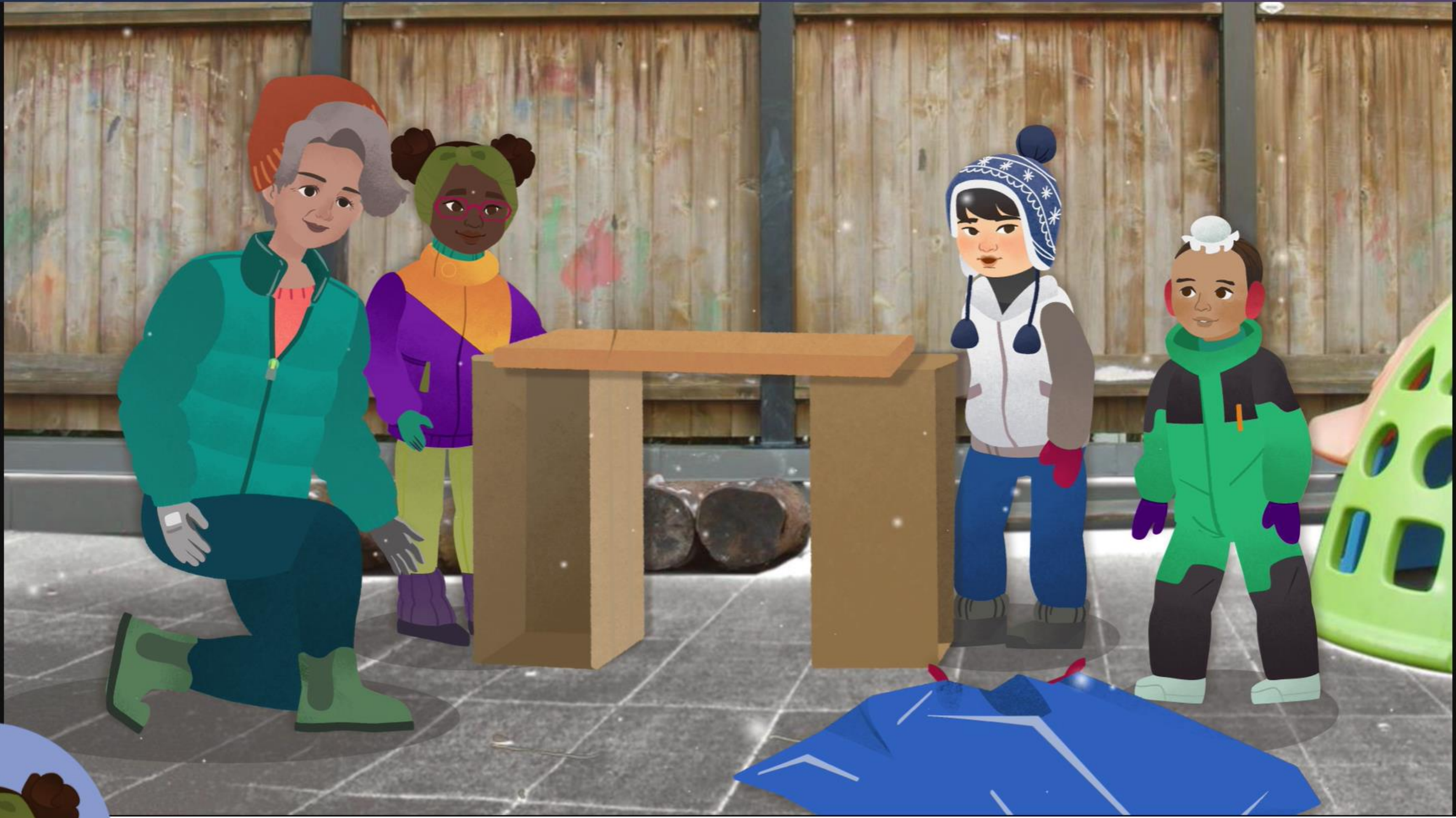

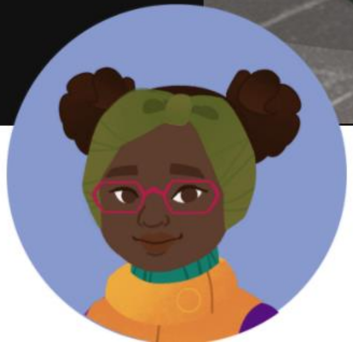

"Good idea!"

NEXT

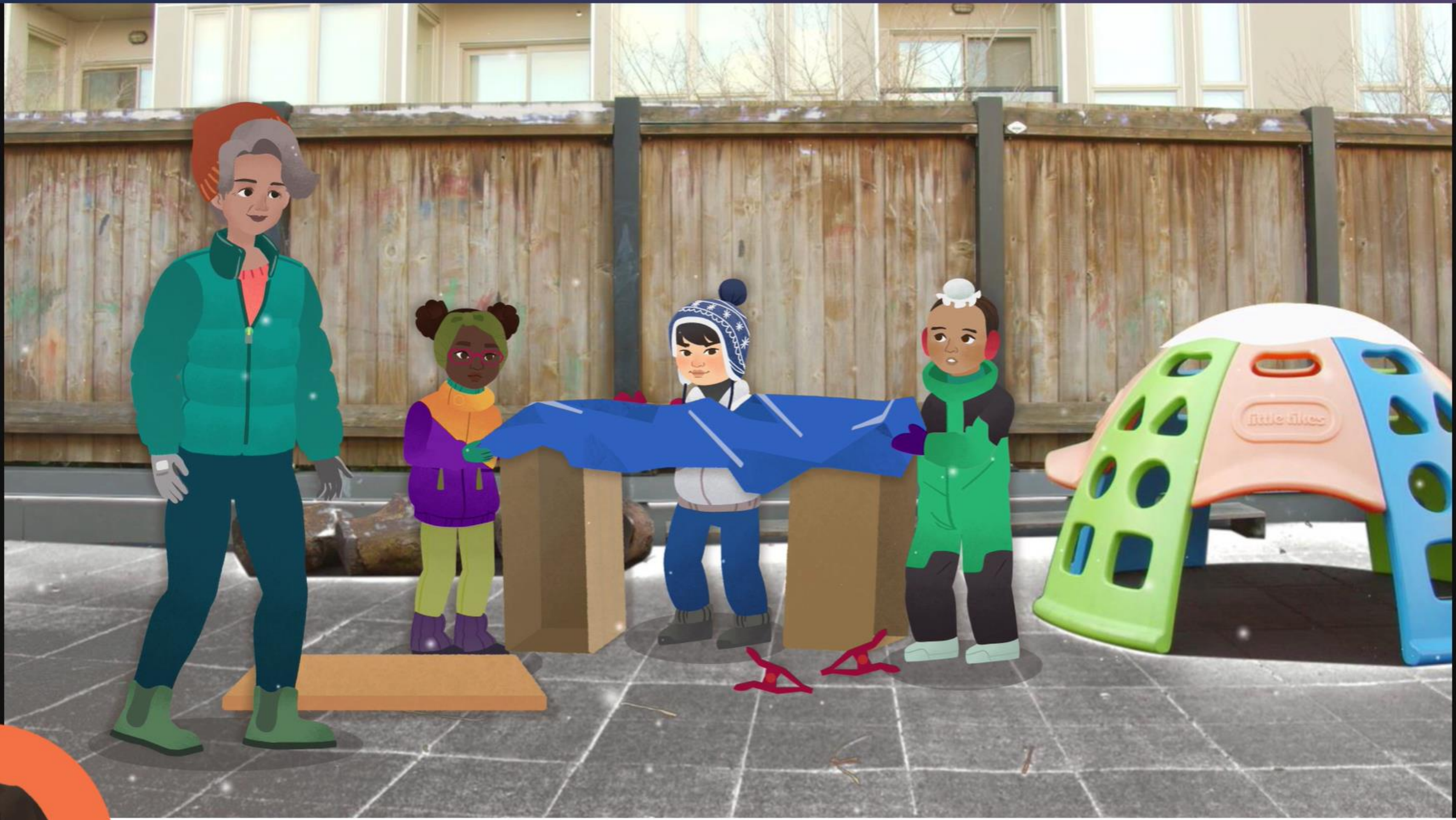

Scenarios

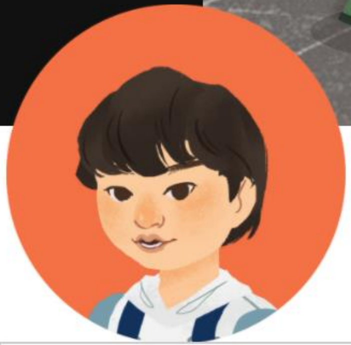

"How can we keep the roof from flying away?"

NEXT

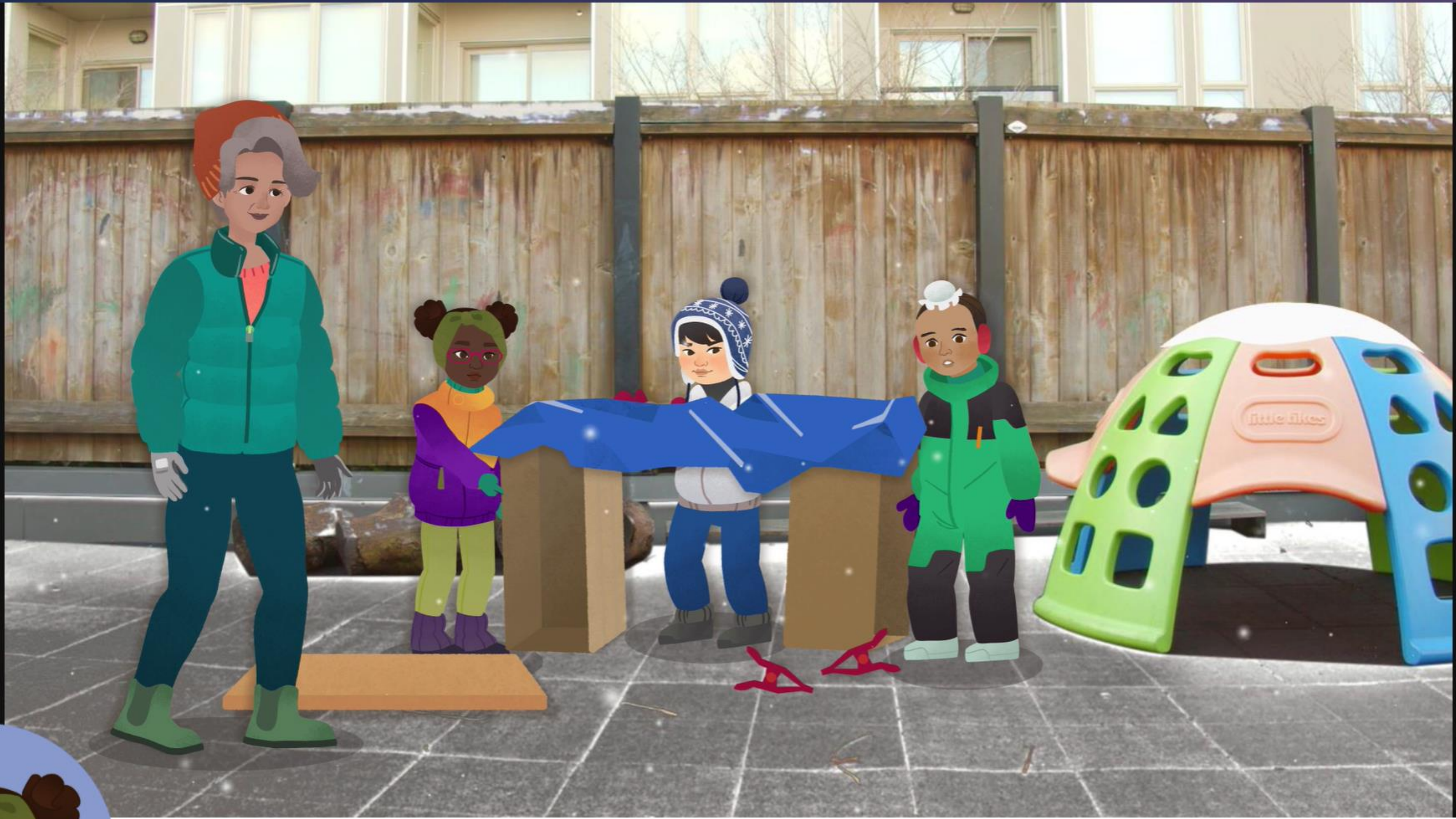

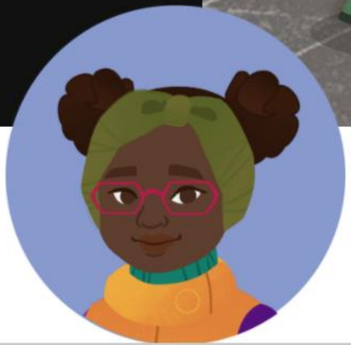

"What about those clamps?"

NEXT

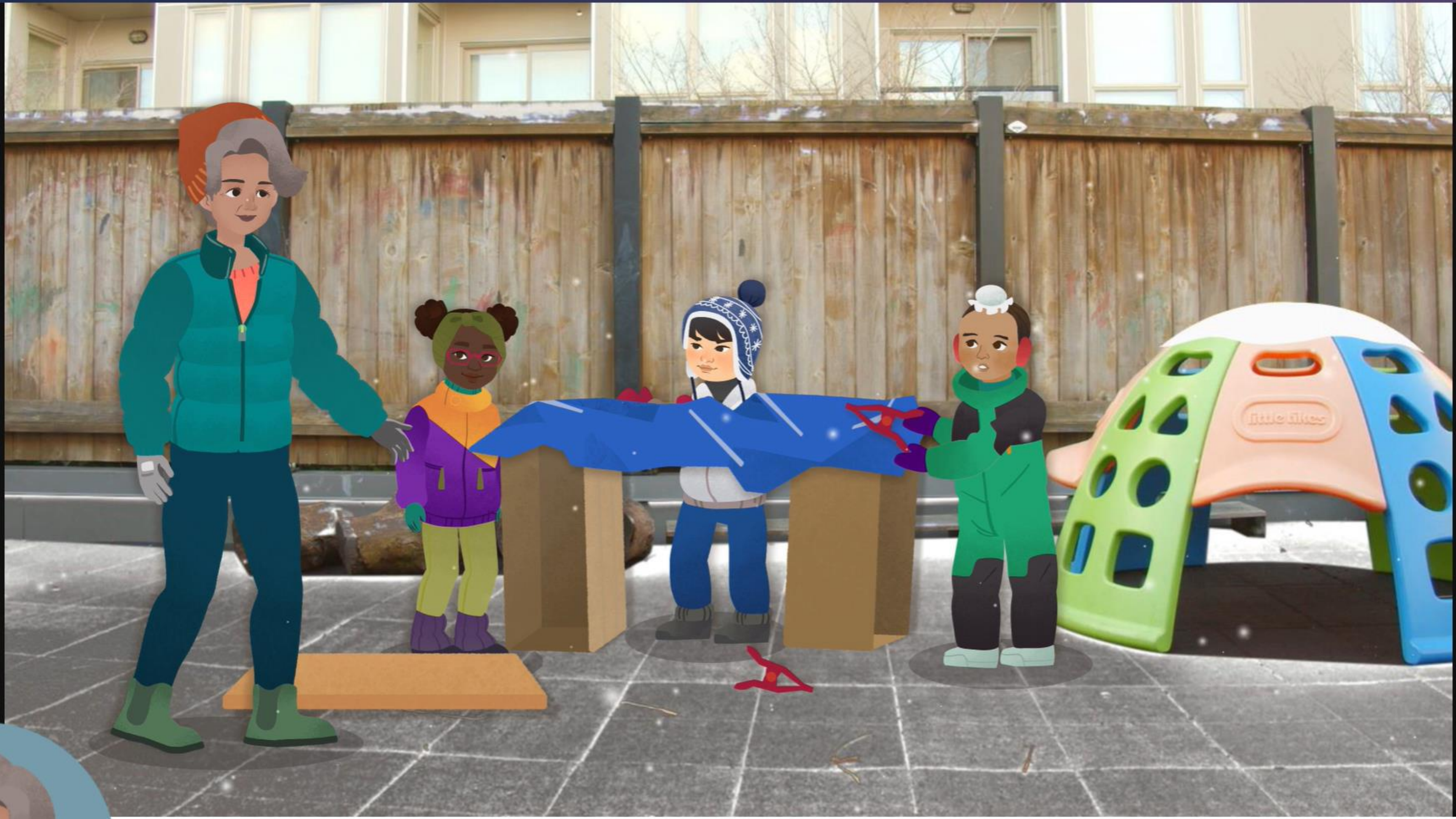

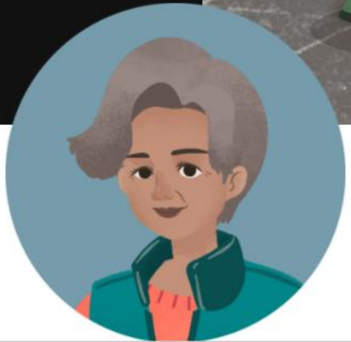

"What would you want to make sure of when you are finding places to attach the clamps?"

NEXT ▶

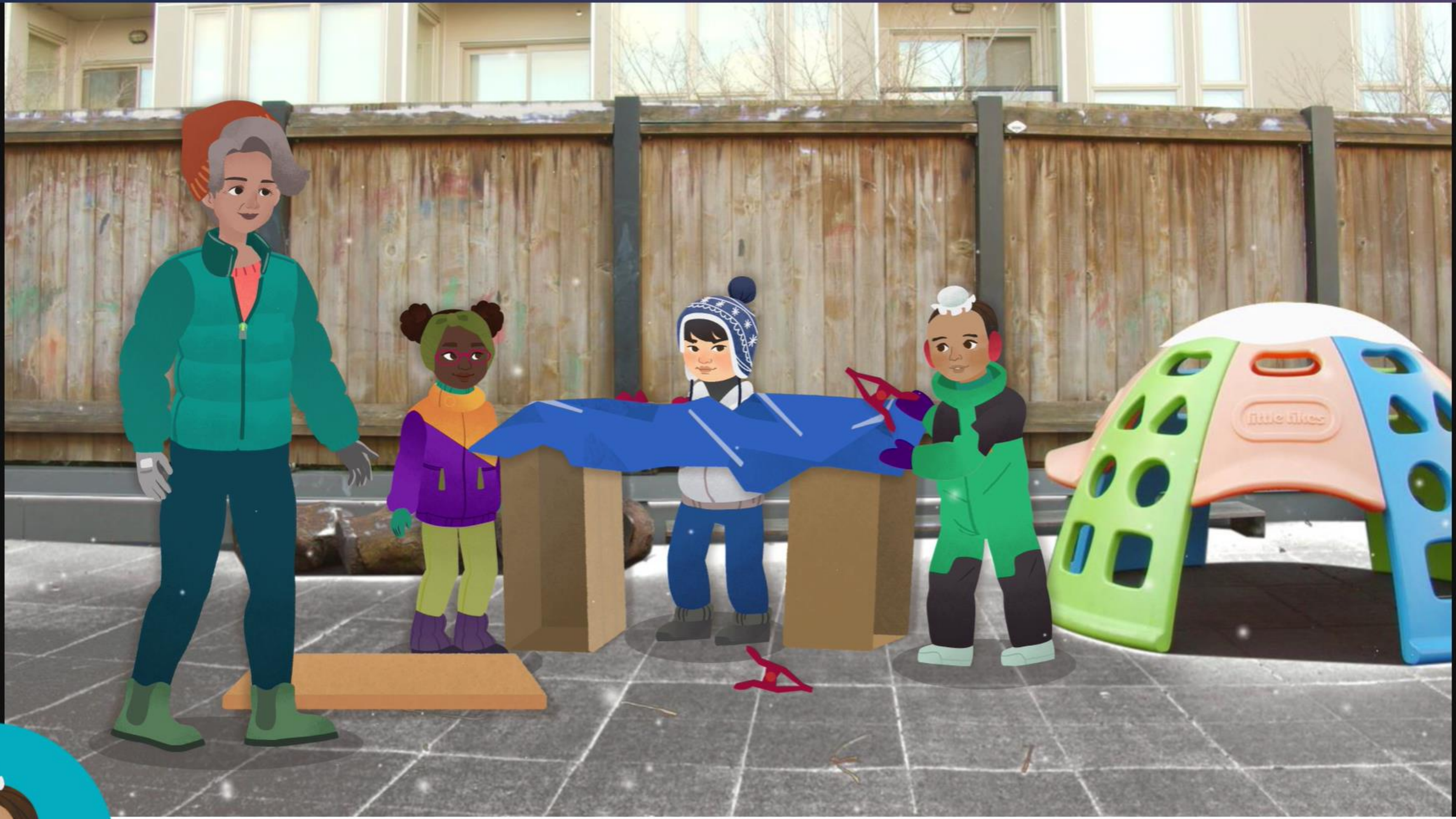

Scenarios

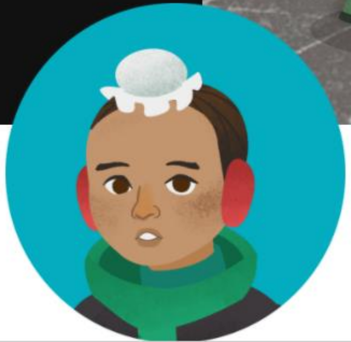

"We want to find places where they can attach to and make sure they won't slip"

NEXT

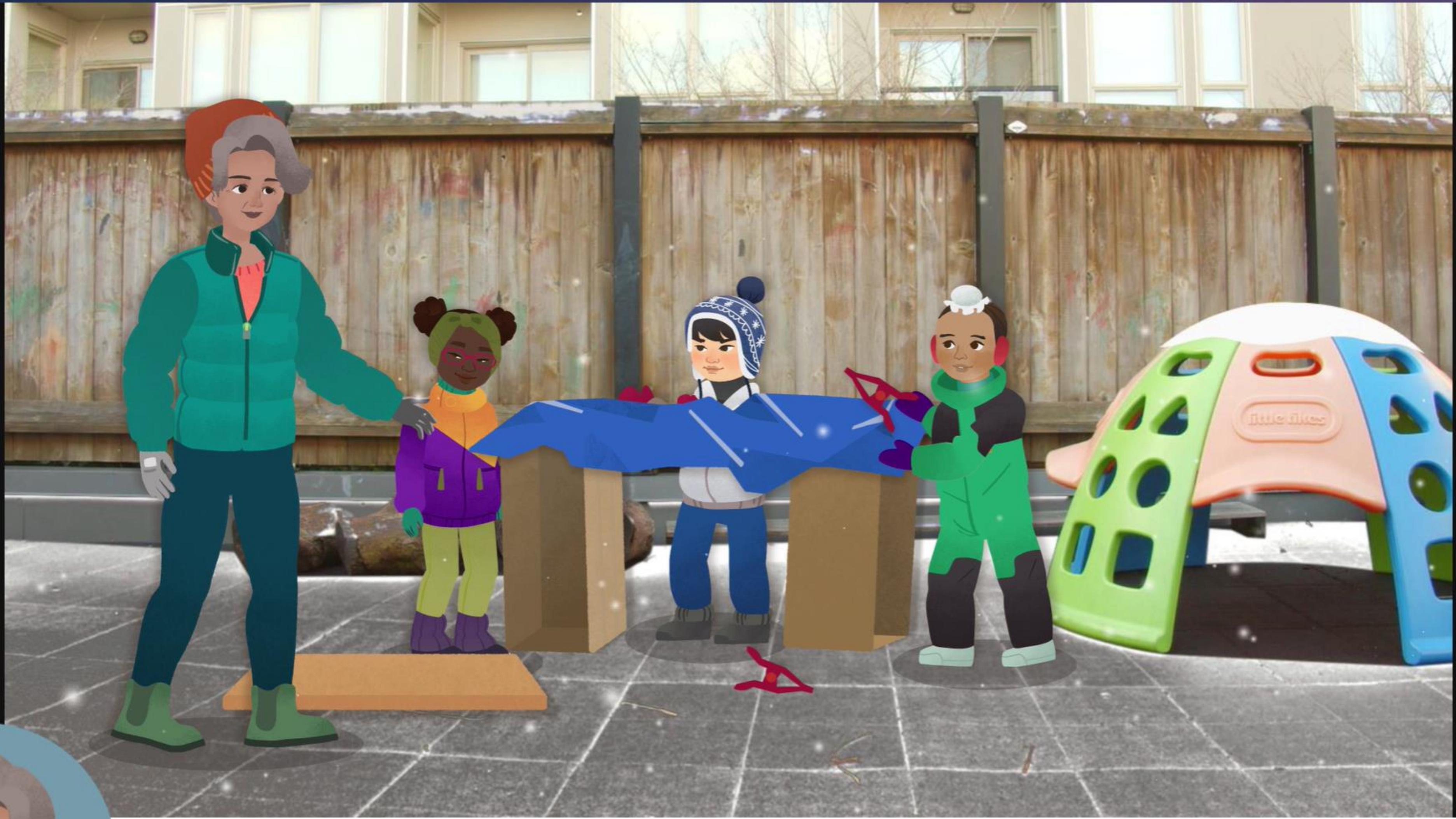

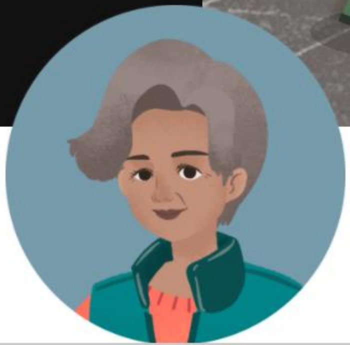

"Yes, where might be the strongest places to attach the clamps to?"

NEXT ▶

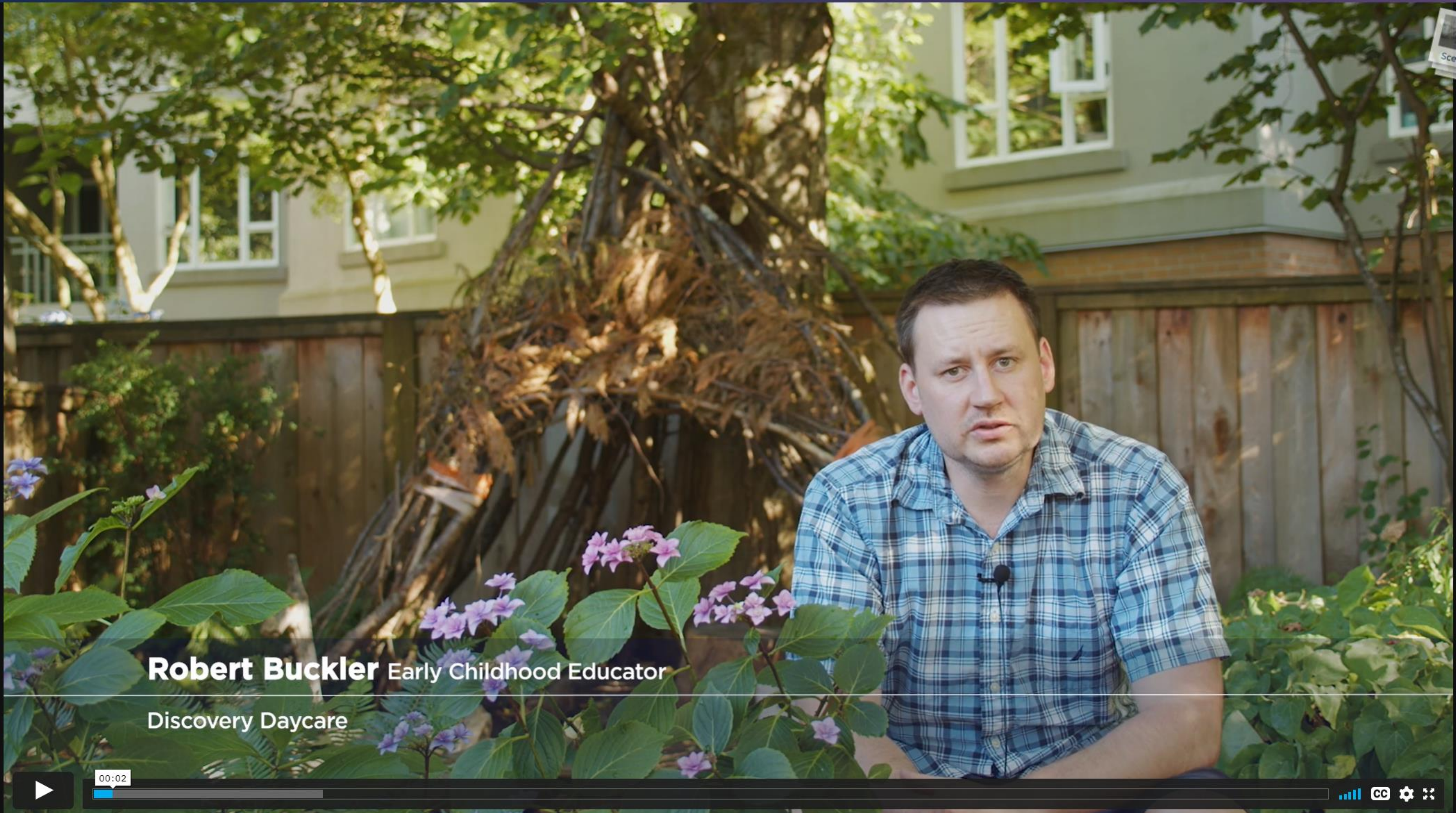
Scenarios
Robert Buckler Early Childhood Educator
Discovery Daycare
00:02

What's the most important thing you learned?

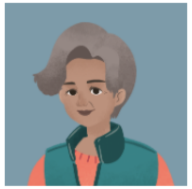

I learned...

Here are some examples if you get stuck

- Loose parts, like mud, water, sticks, can provide wonderful play value and bring joy.
- Children's risky play experiences help them develop risk management skills
- Risk benefit assessment principles can guide how I facilitate the play.
- My familiarity and relationship with the children can help me learn the capabilities of each child, how much support I need to provide and help build trust in their abilities to keep themselves safe.
- It's ok if children experiment "fails". This can be an incredible learning opportunity.
- Recognizing when risks become too hazardous depends on the context and the child's abilities.

Save and Continue

Chapter 2:

Scenarios

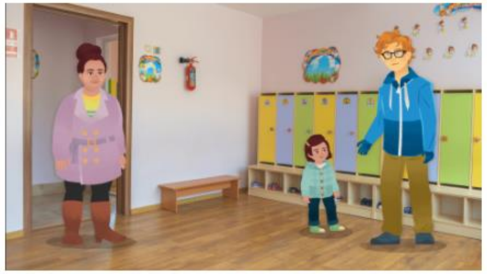

Communicating with Parents/Caregivers

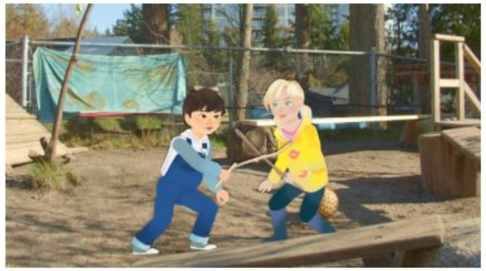

Rough and Tumble Play

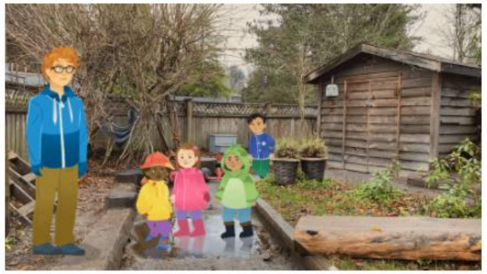

Play at Speed

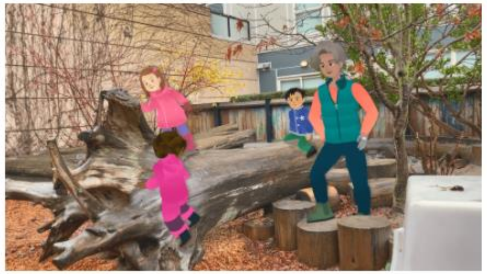

Play at Heights

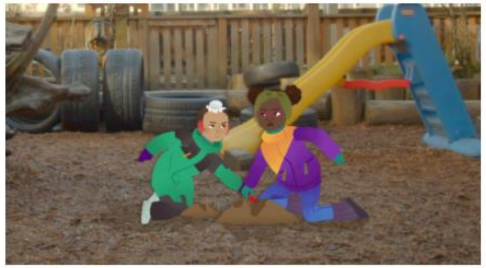

Conflict Resolution

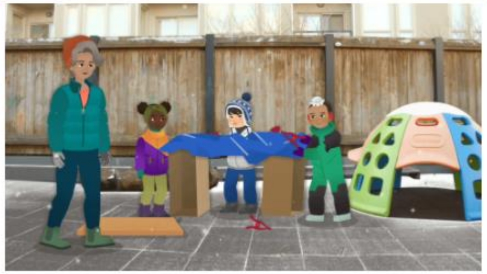

Play with Loose Parts

CONTINUE TO CHAPTER 3

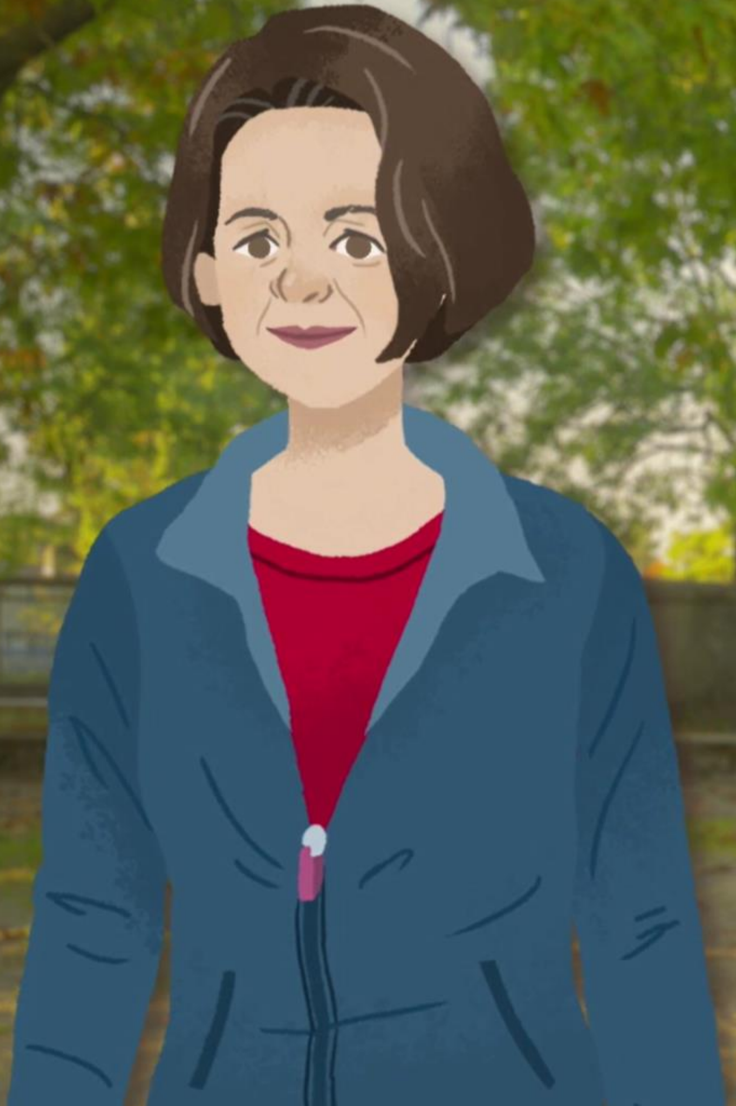

What do you think the children get out of these experiences when their outdoor play was supported? (Select all that apply)

Confidence	Connection to the land	Courage
Creativity	Curiosity	Empathy
Independence	Joy	Leadership
Physical competence	Problem-solving	Resilience
Sense of belonging	Sense of freedom	Spirituality

Next

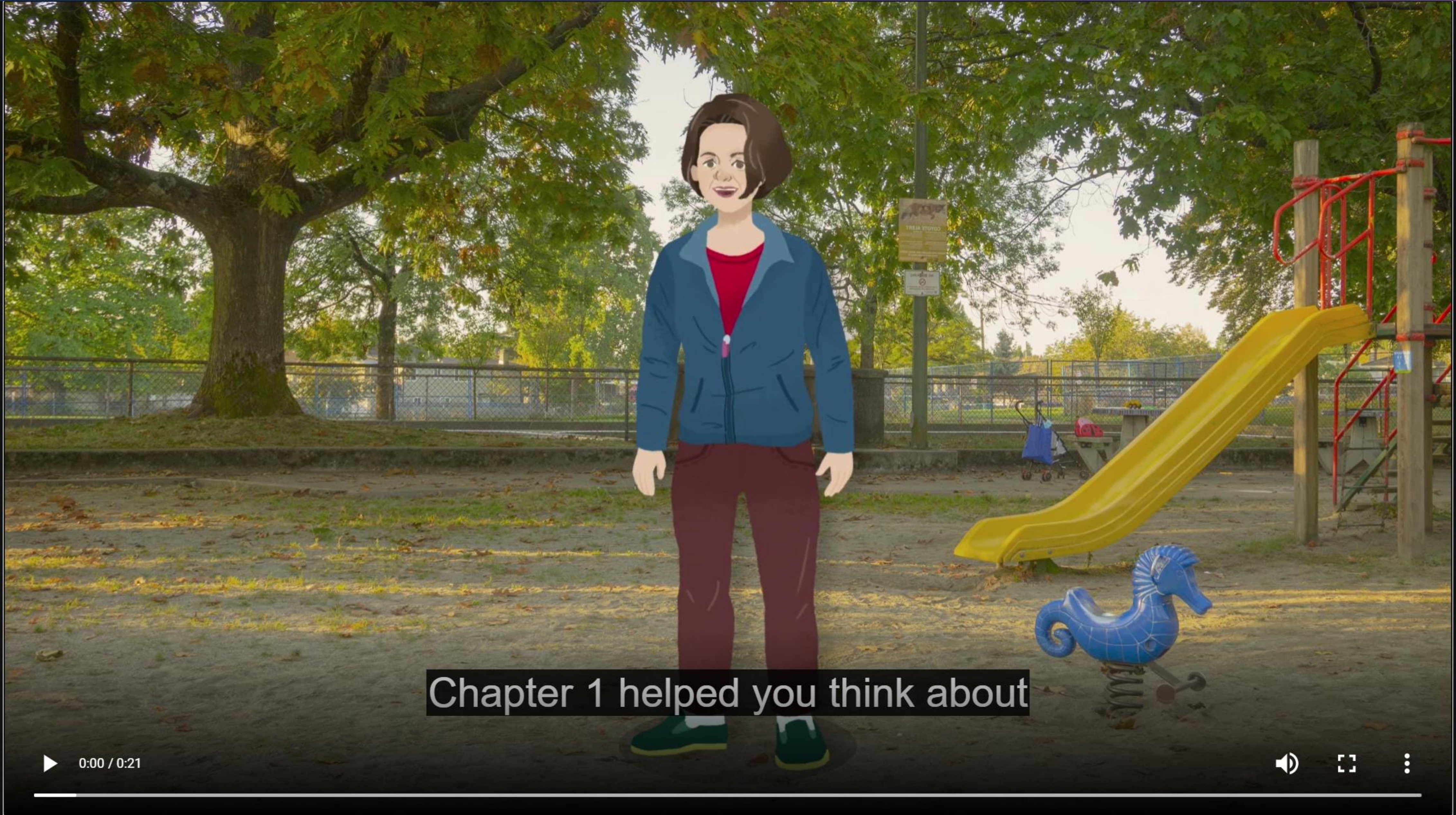
Chapter 1 helped you think about
0:00 / 0:21

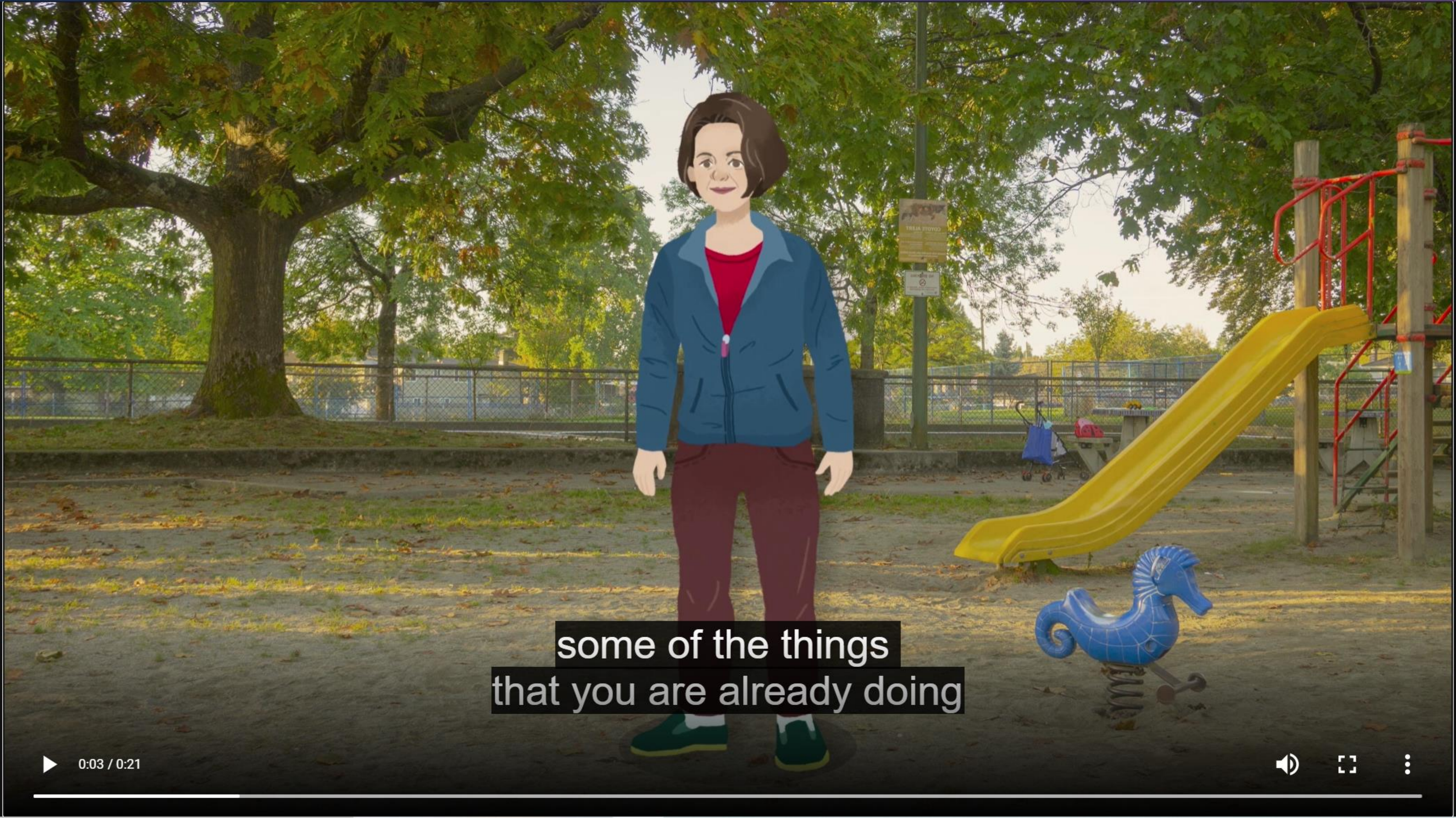
some of the things
that you are already doing
0:03 / 0:21

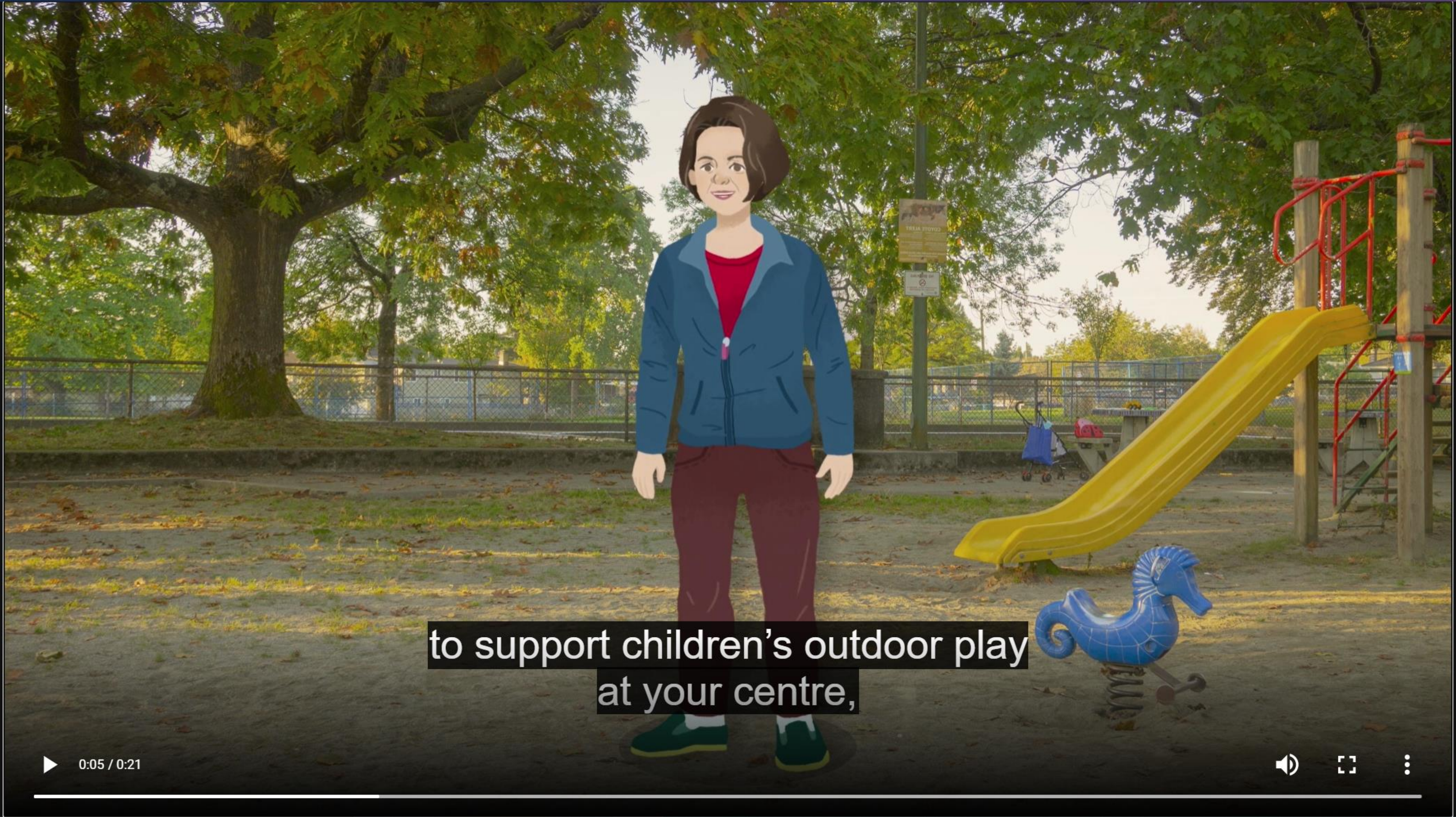
to support children’s outdoor play
at your centre,
0:05 / 0:21

Take a risk, go play outside!

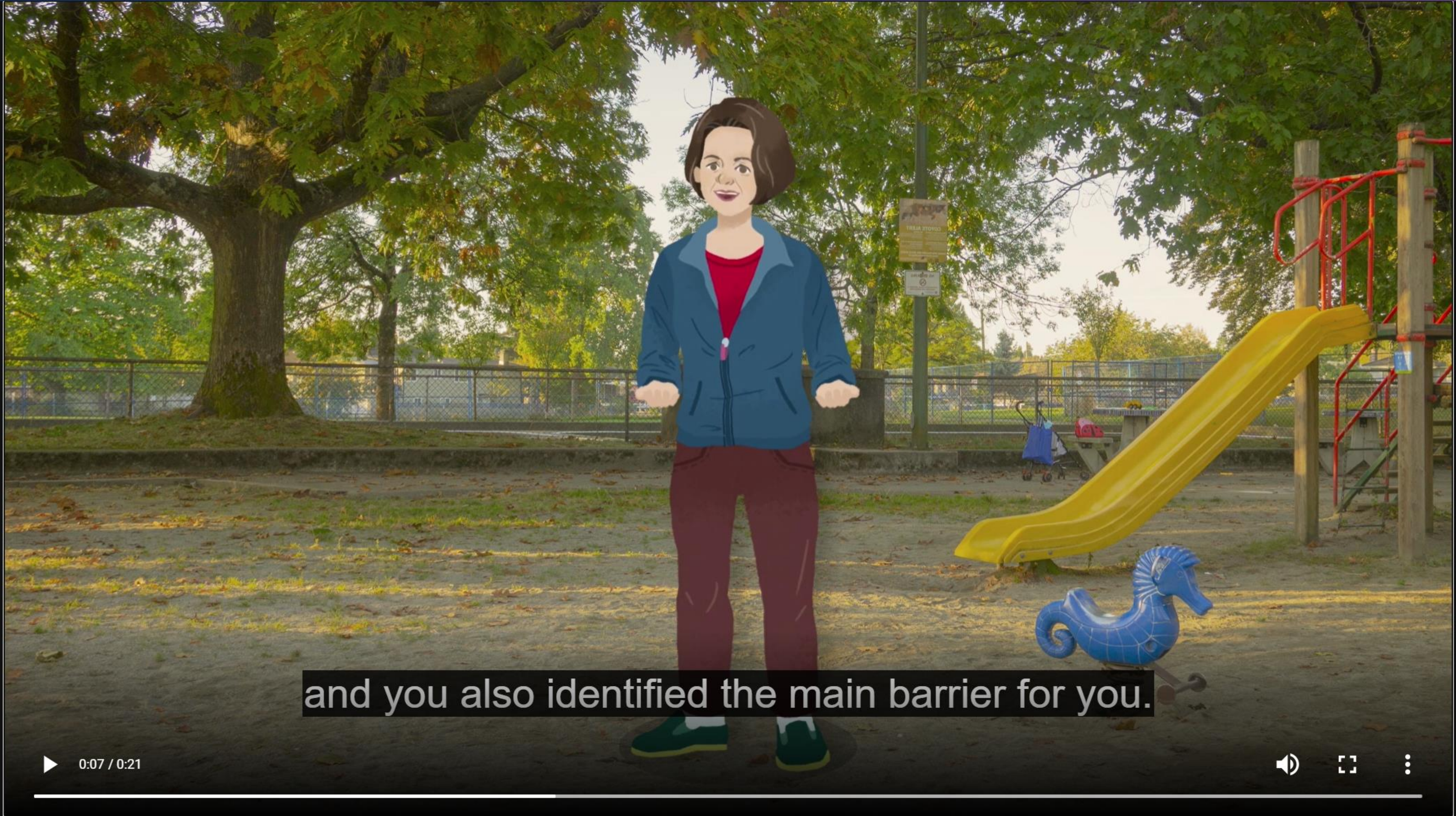

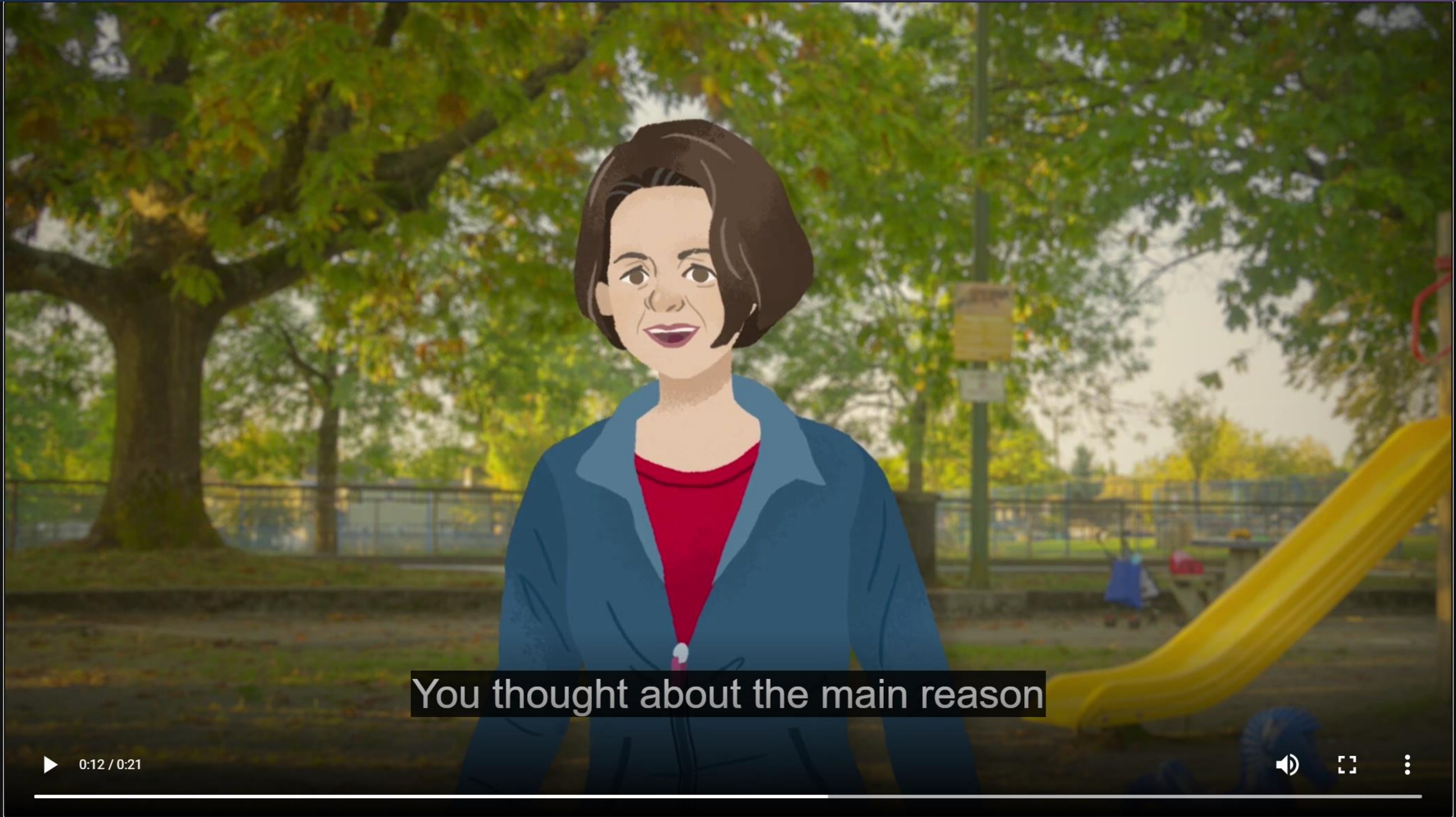
You thought about the main reason
0:12 / 0:21

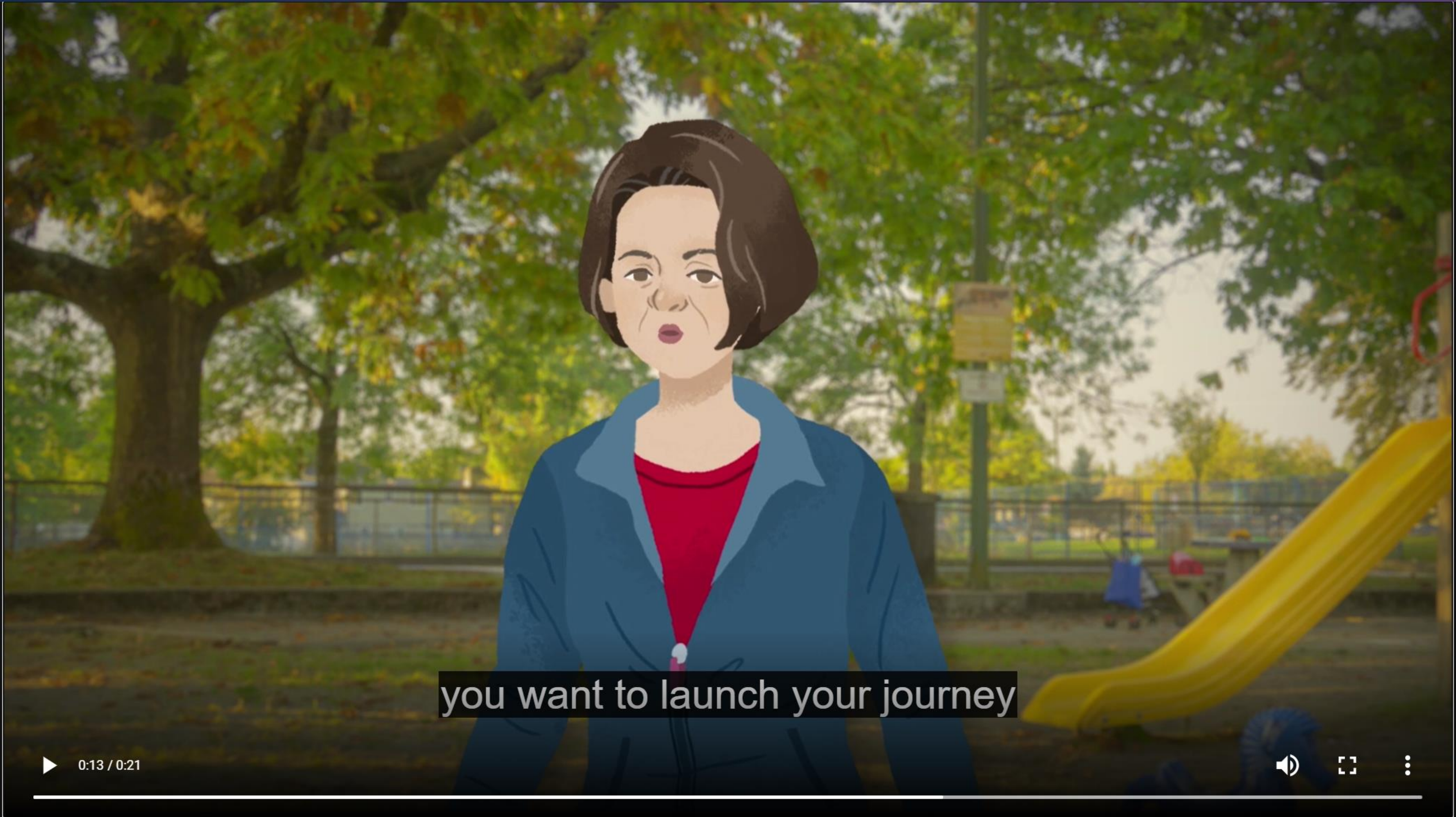
you want to launch your journey
0:13 / 0:21

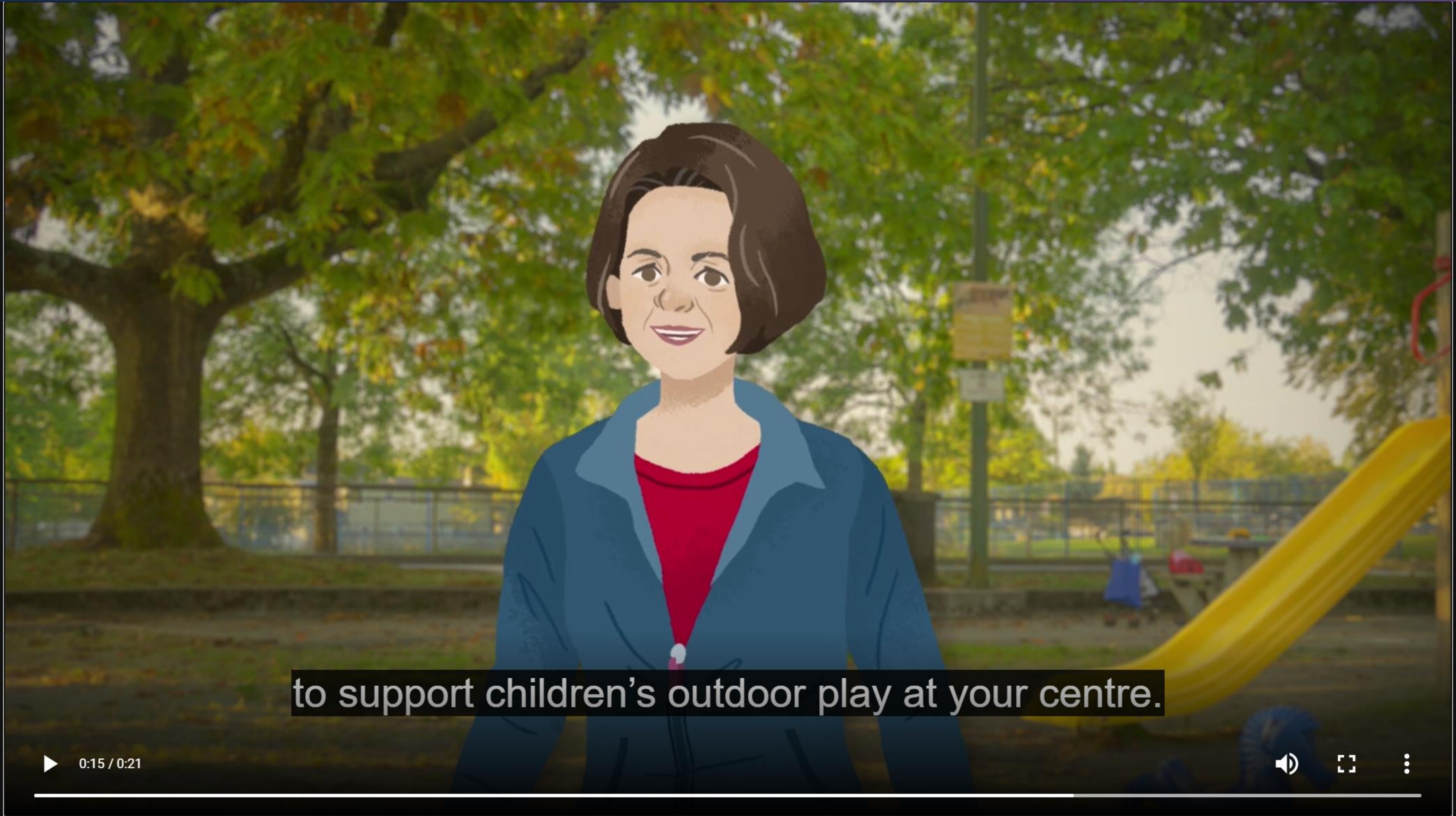
to support children’s outdoor play at your centre.
0:15 / 0:21

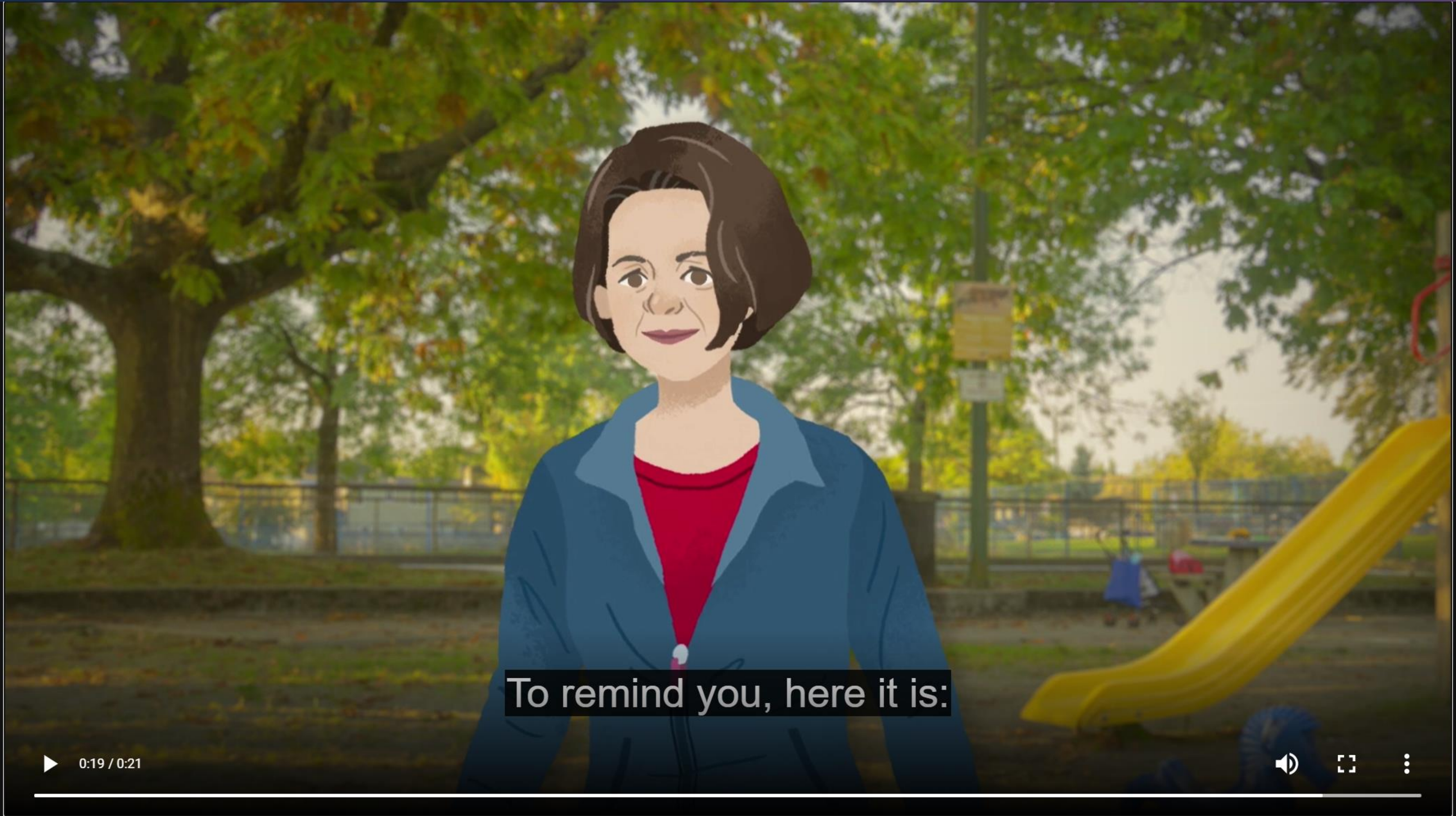
To remind you, here it is:
0:19 / 0:21

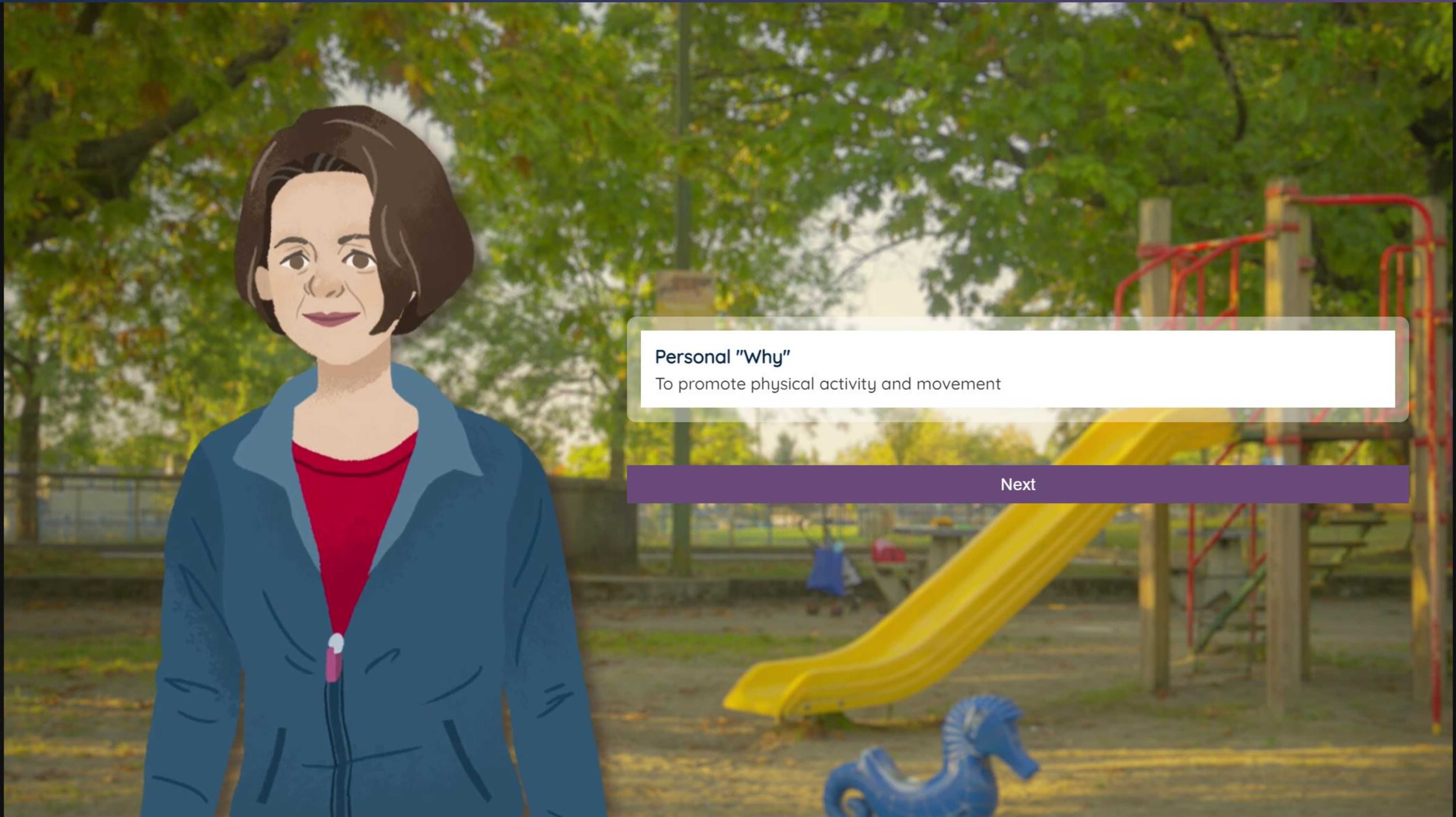
Personal "Why"
To promote physical activity and movement
Next

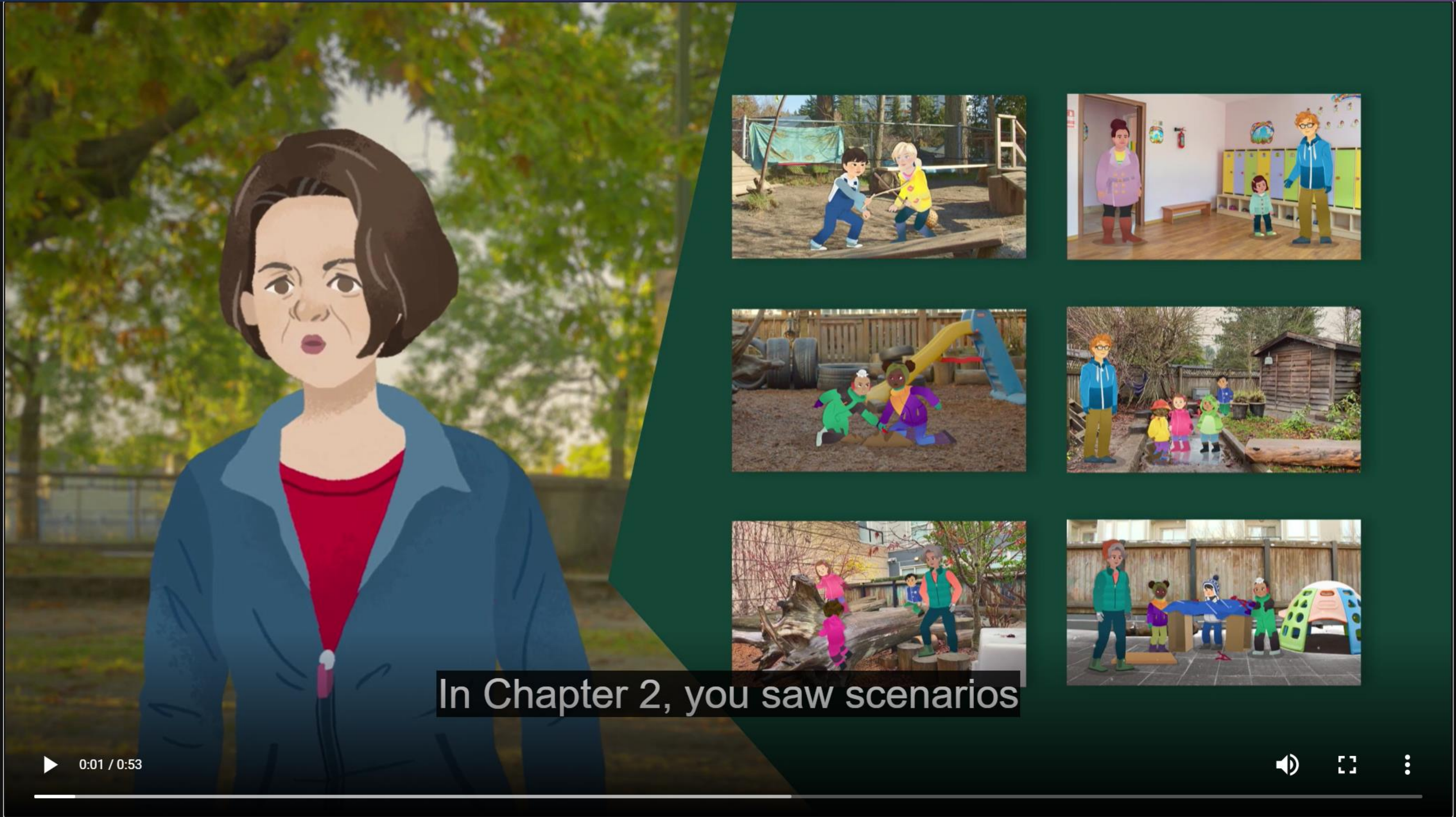
In Chapter 2, you saw scenarios
0:01 / 0:53

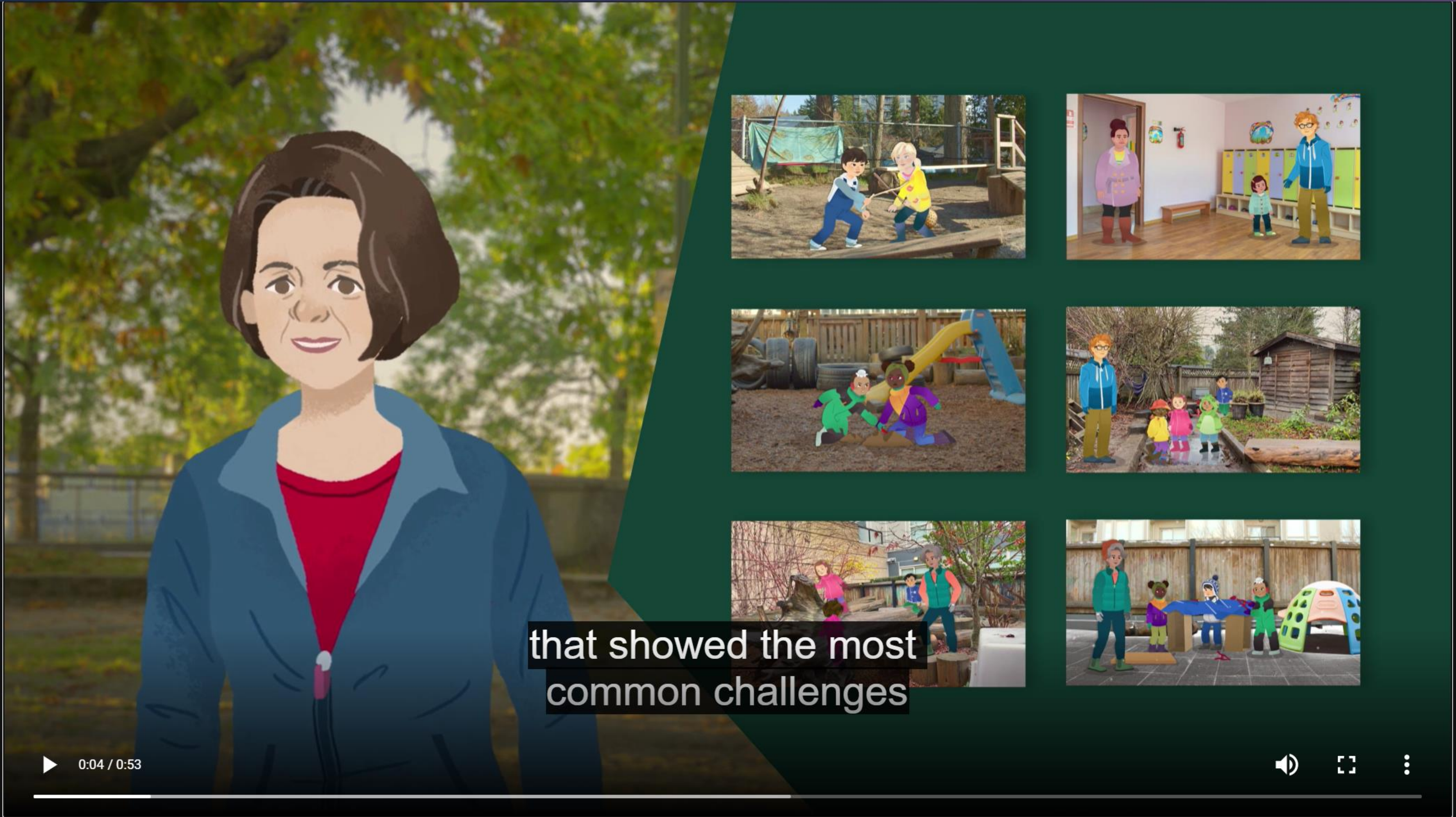
that showed the most
common challenges
0:04 / 0:53

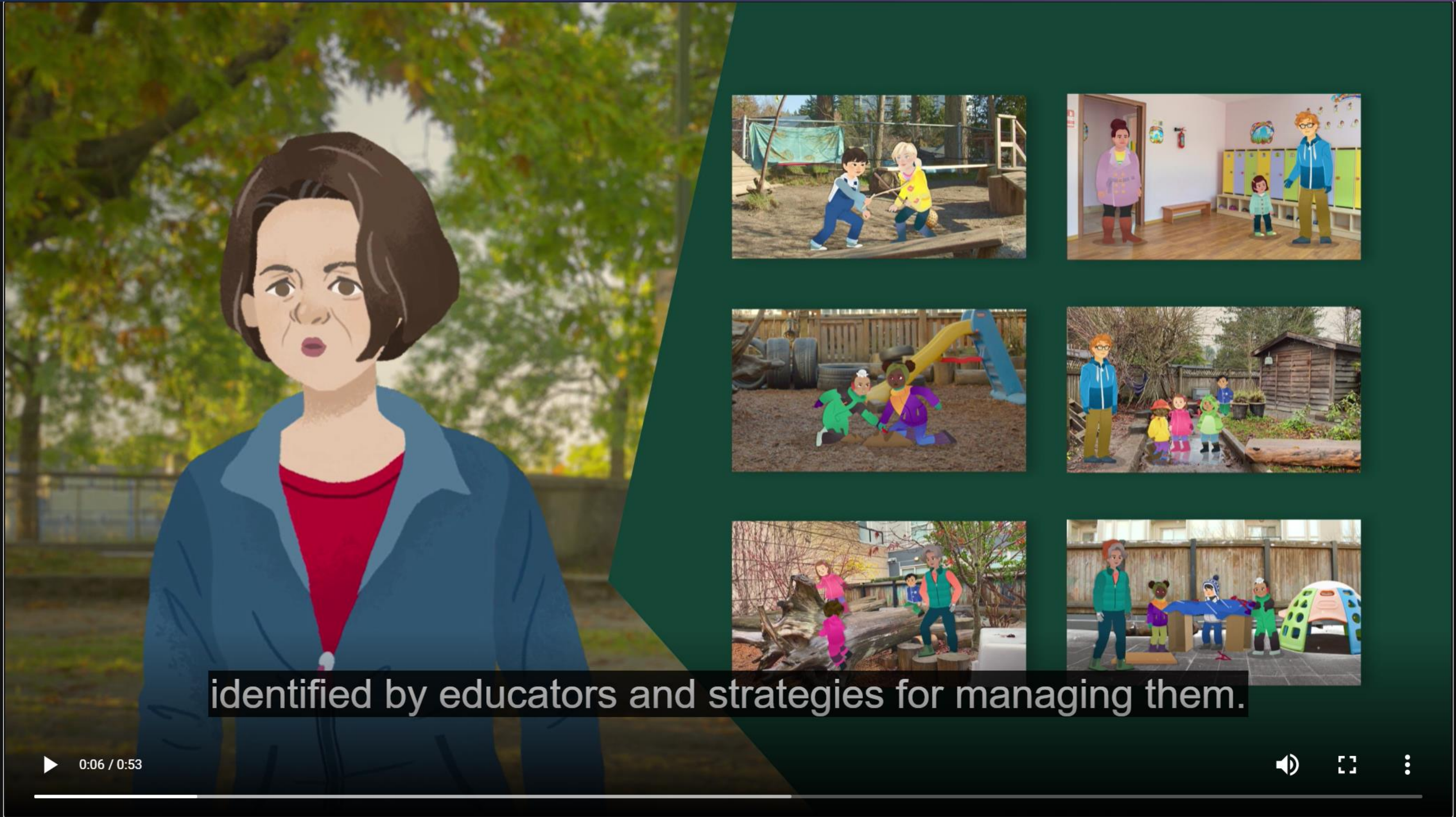
identified by educators and strategies for managing them.
0:06 / 0:53

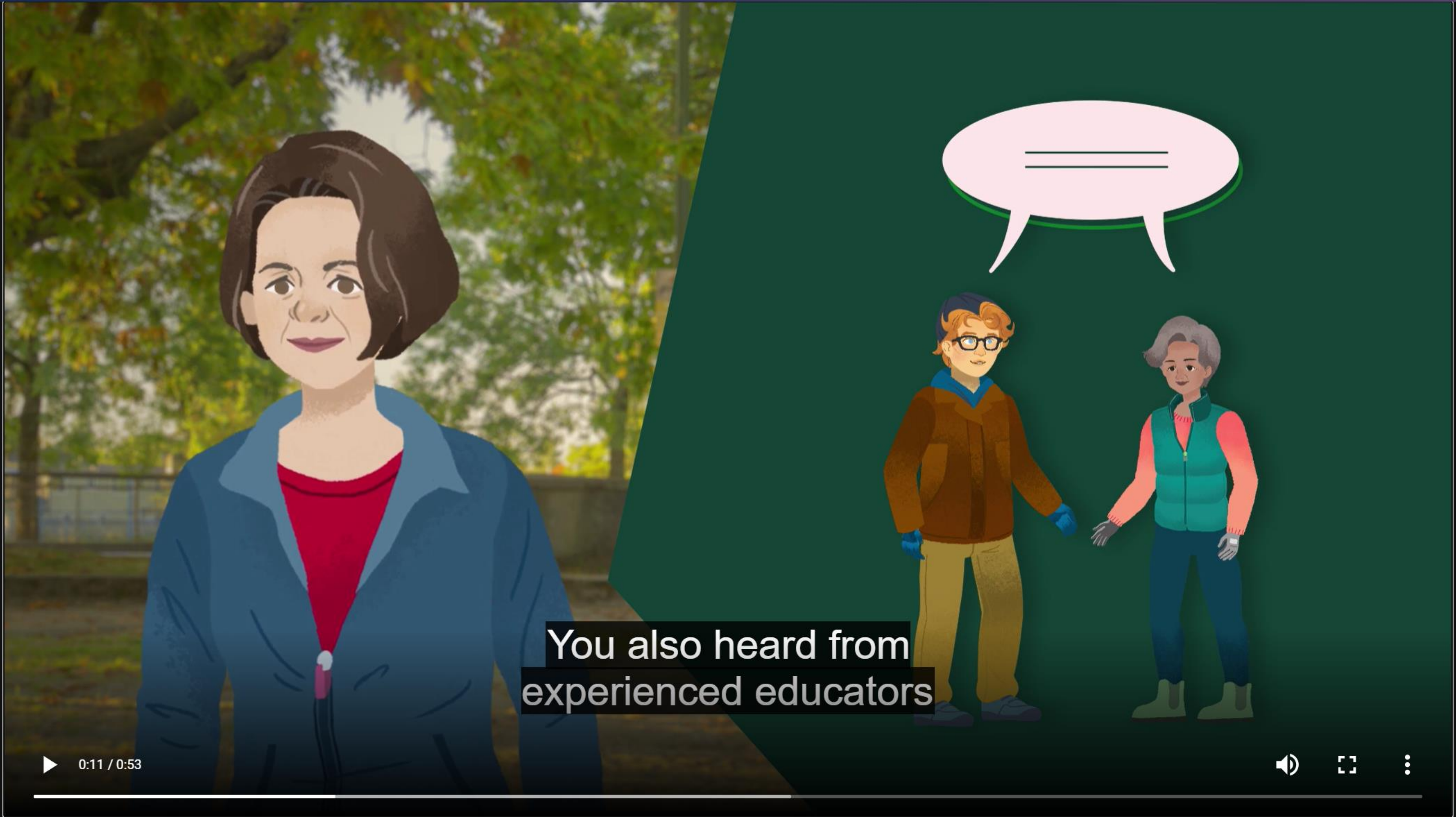
You also heard from
experienced educators
0:11 / 0:53

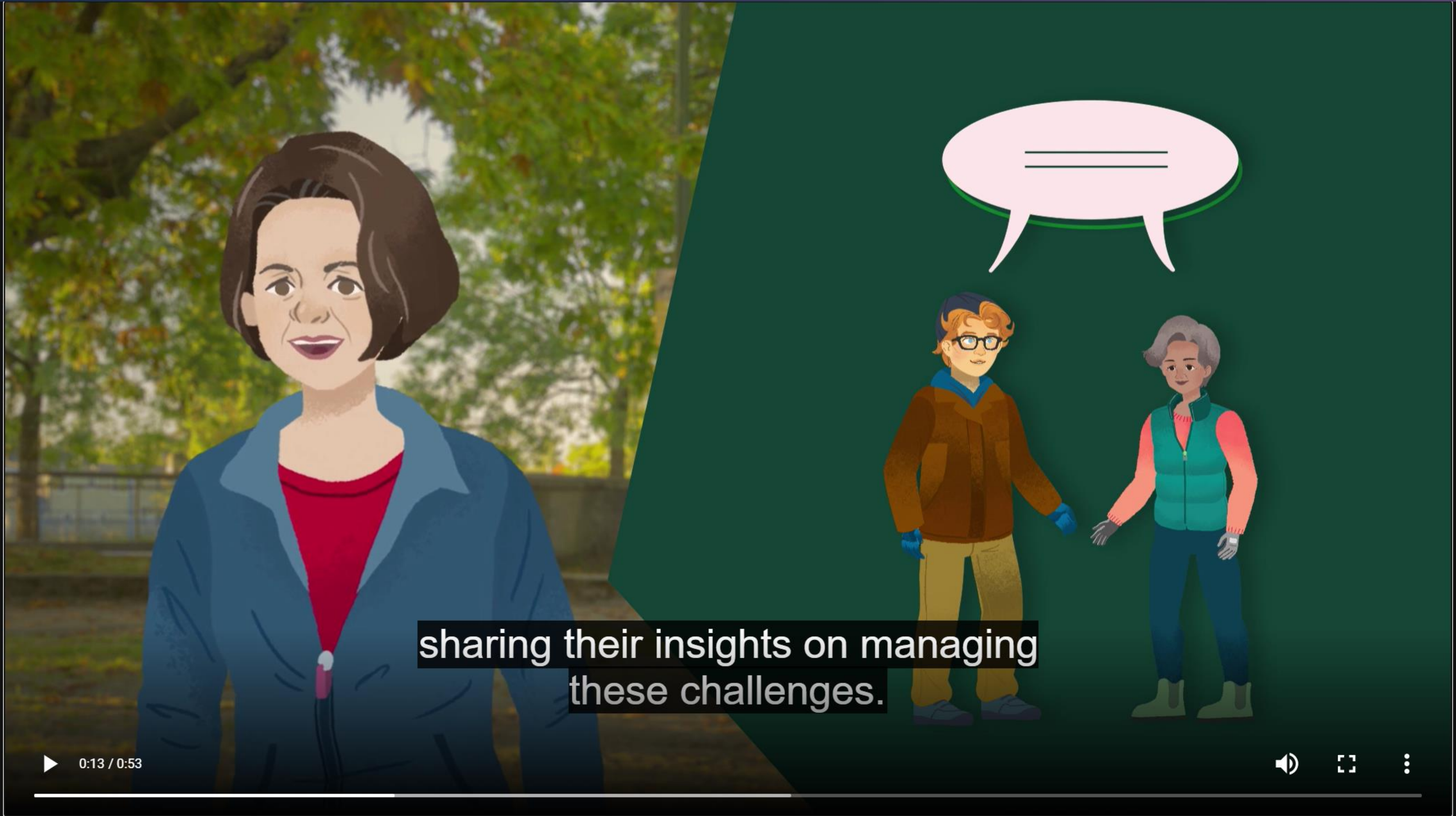
sharing their insights on managing
these challenges.
0:13 / 0:53

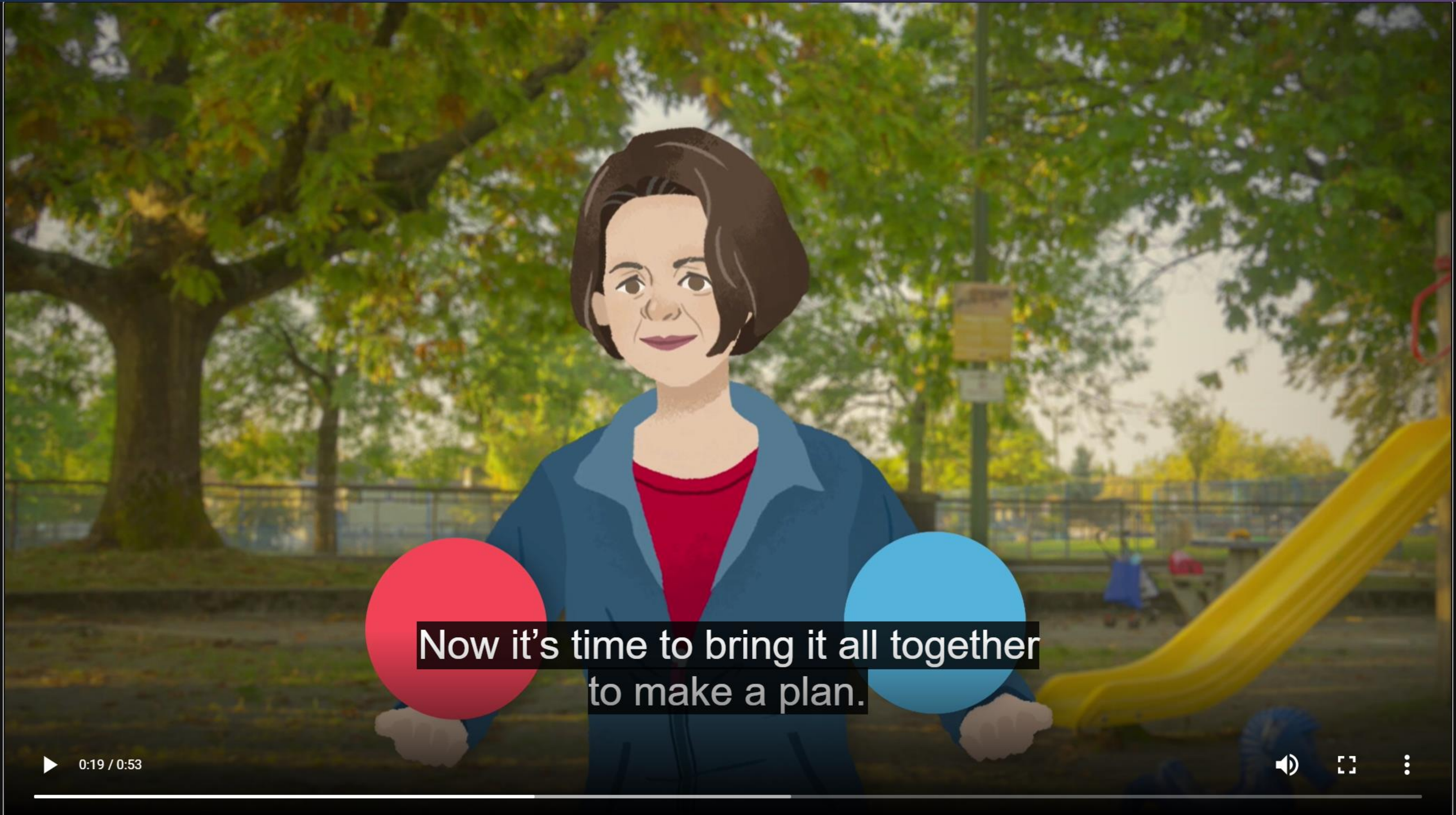
Now it's time to bring it all together
to make a plan.
0:19 / 0:53

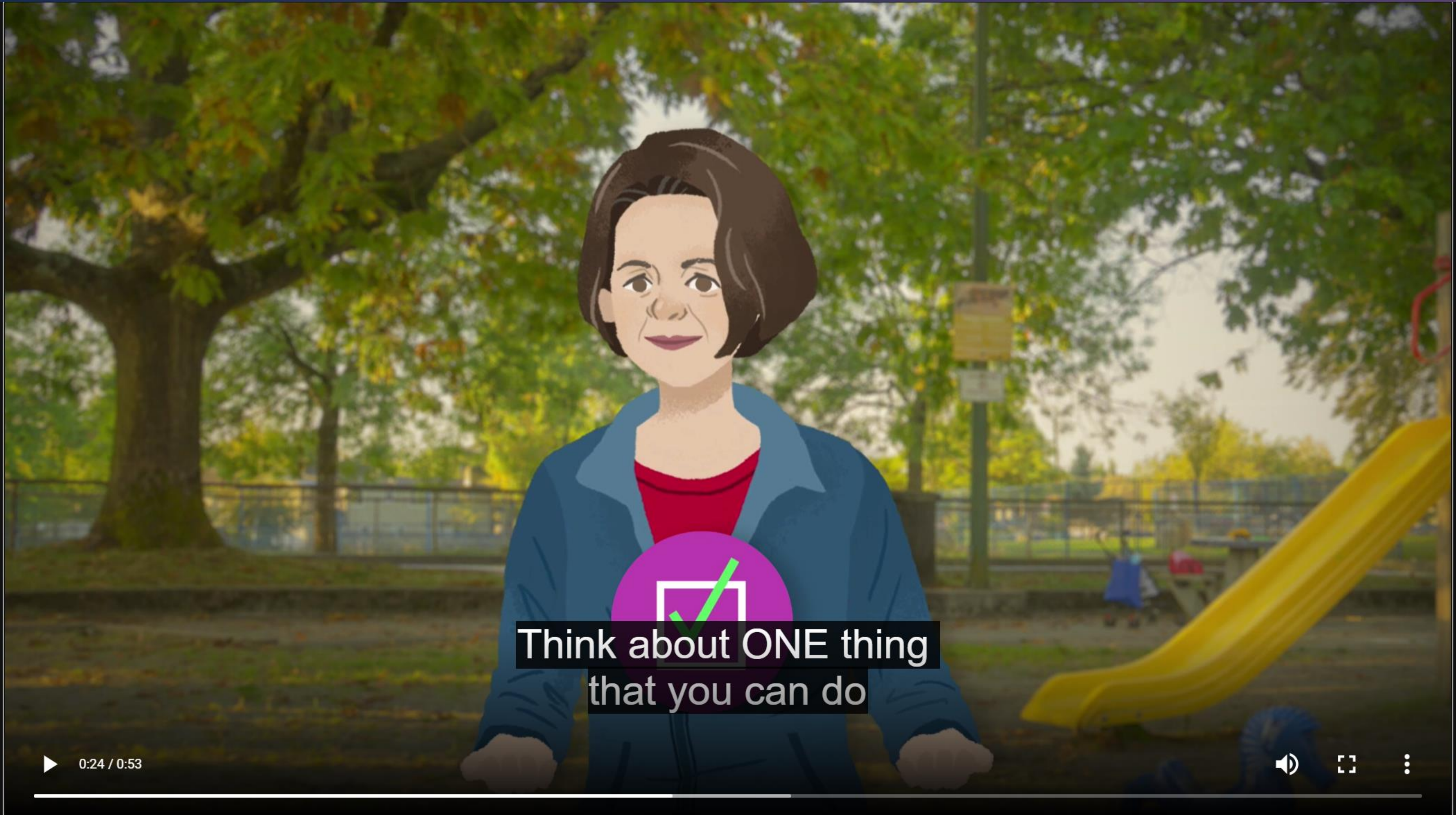
Think about ONE thing
that you can do
0:24 / 0:53

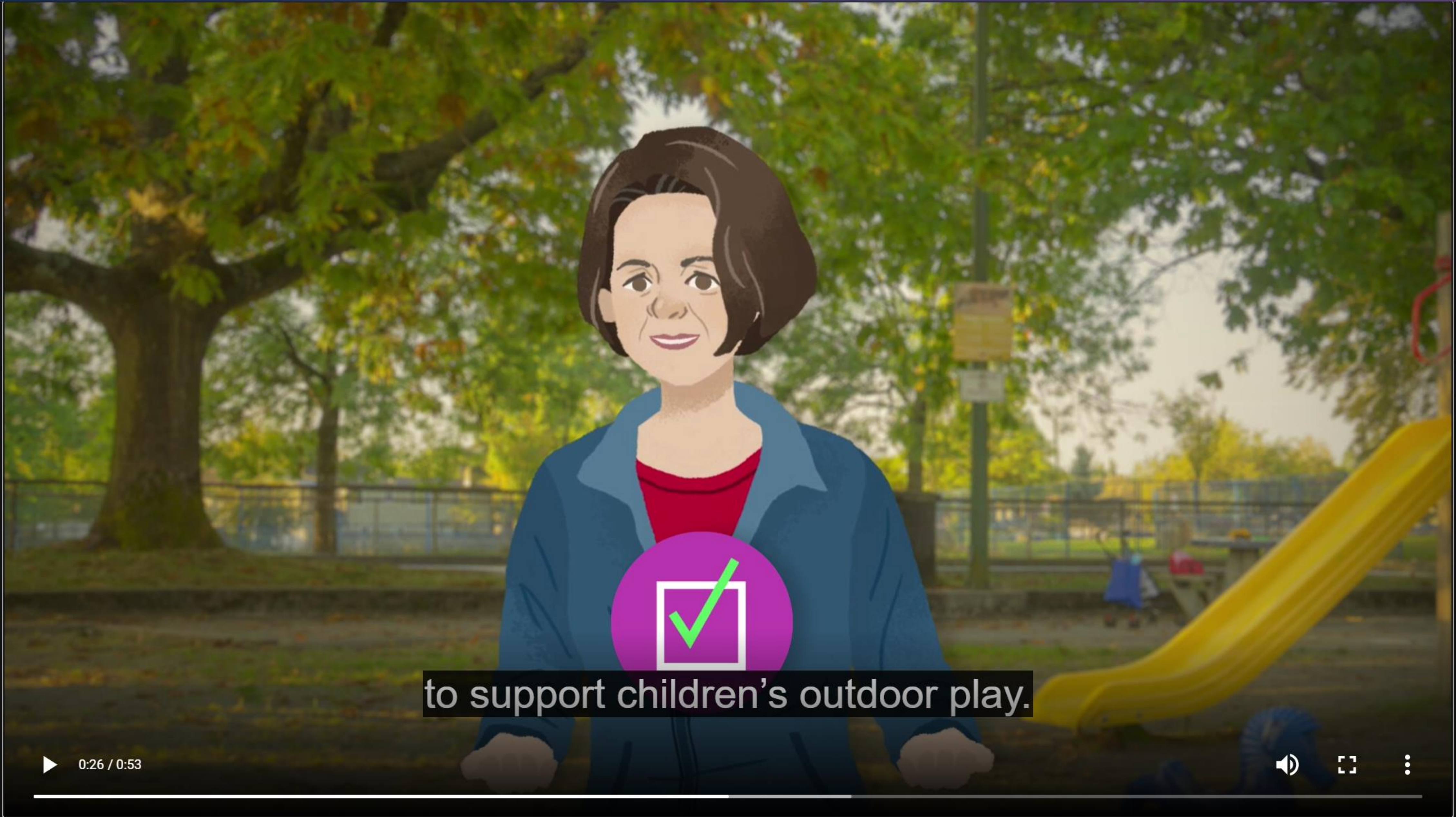
to support children's outdoor play.
0:26 / 0:53

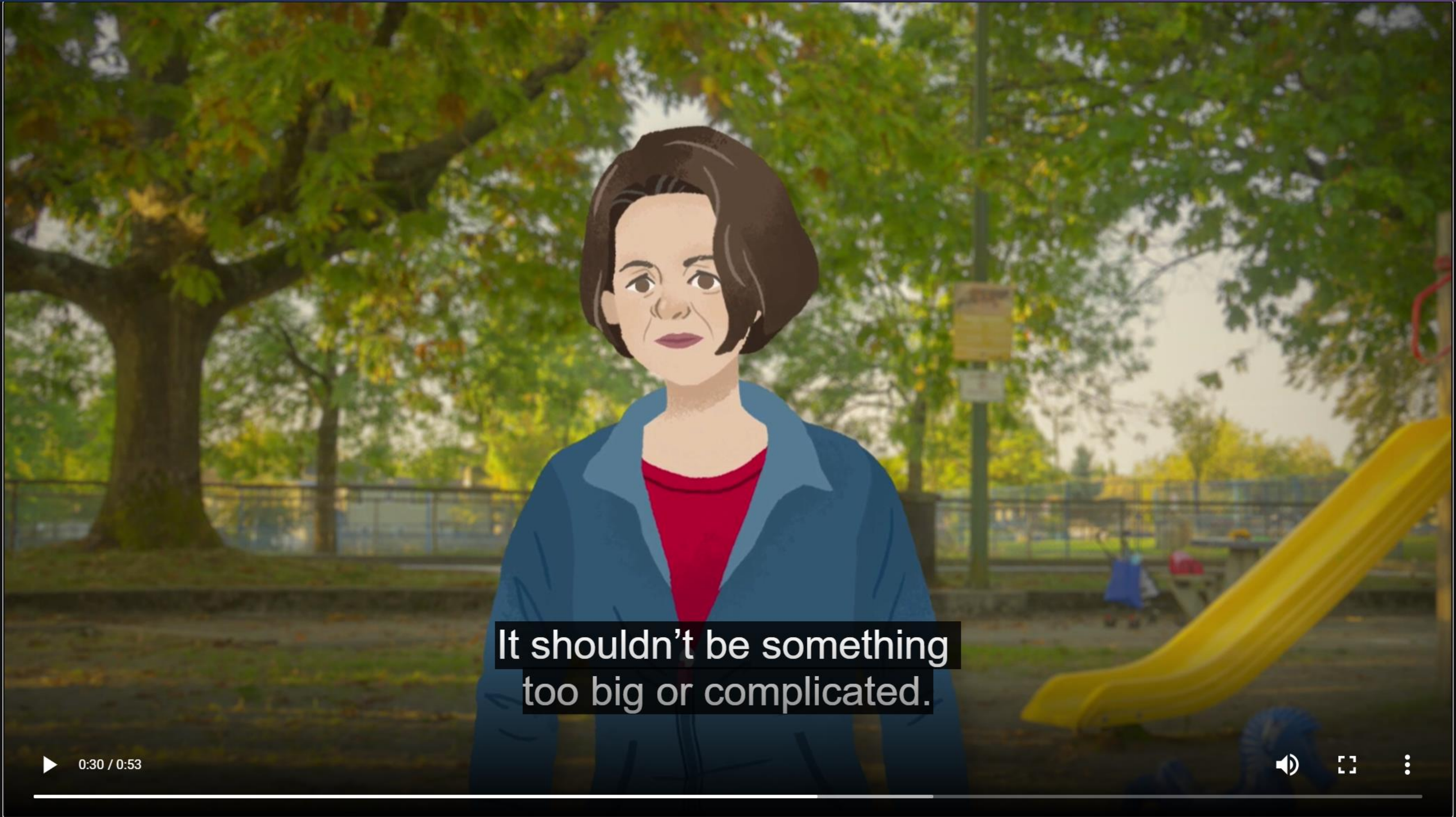
It shouldn't be something
too big or complicated.
0:30 / 0:53

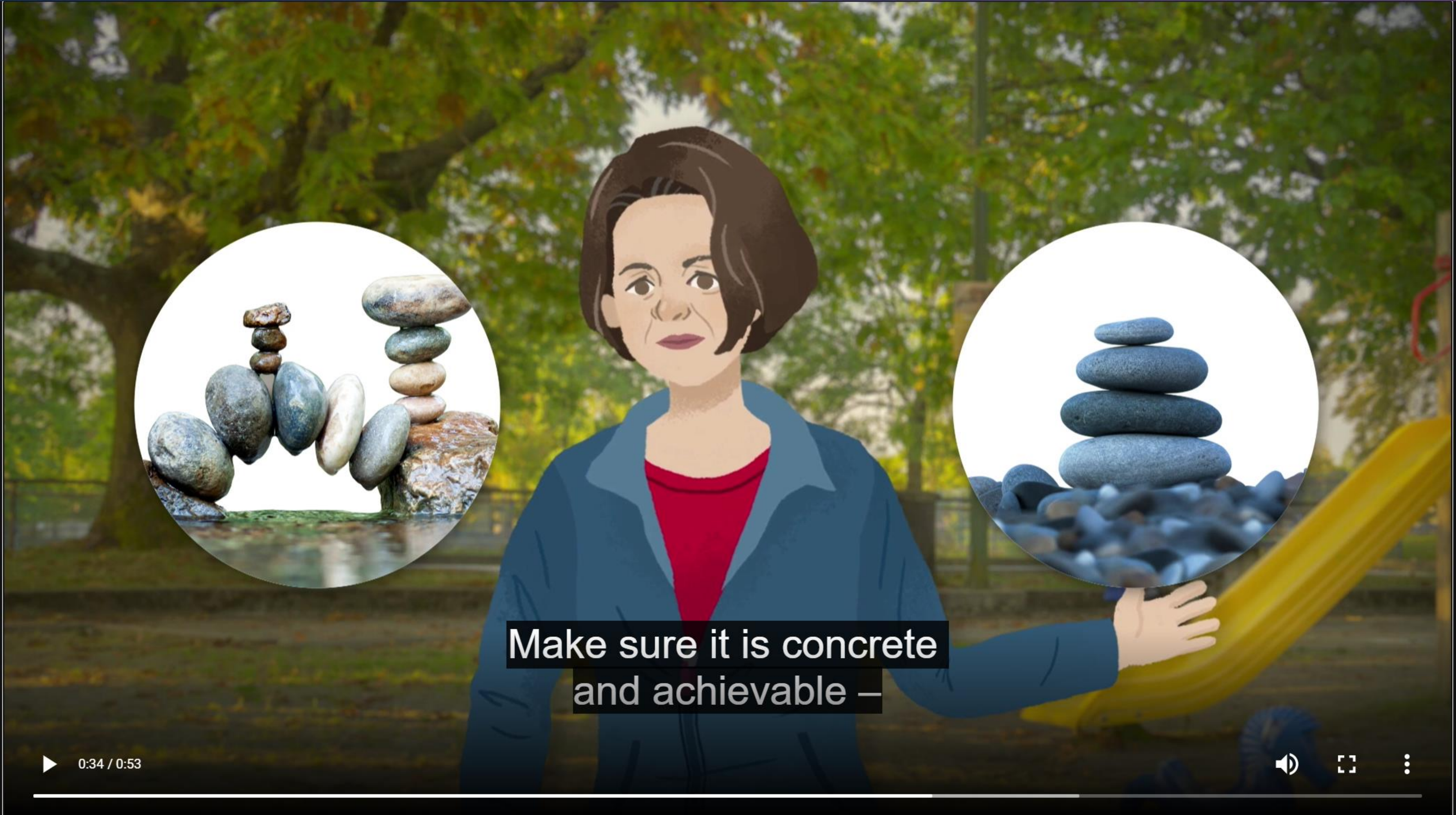
Make sure it is concrete
and achievable –
0:34 / 0:53

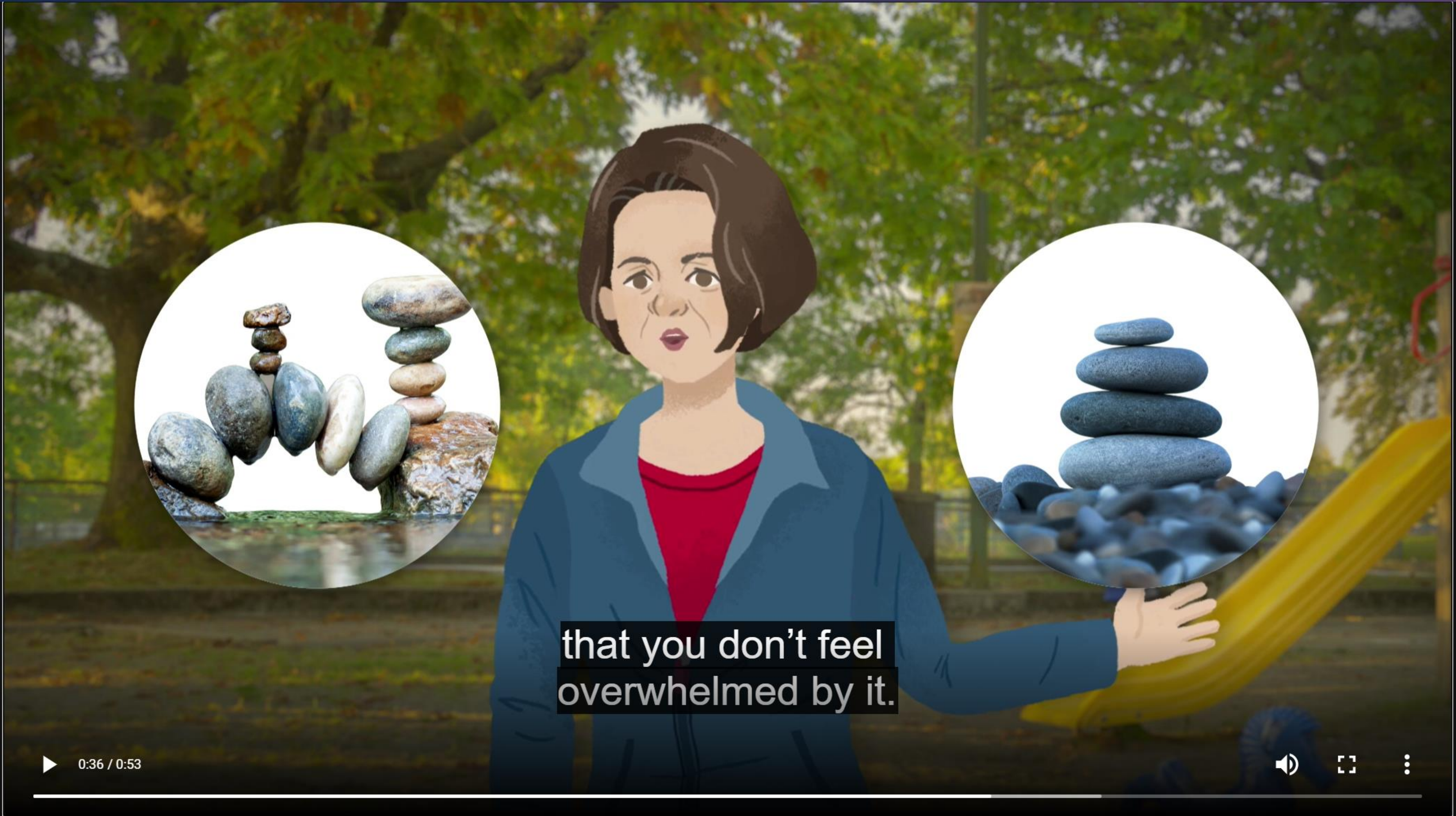
that you don't feel
overwhelmed by it.
0:36 / 0:53

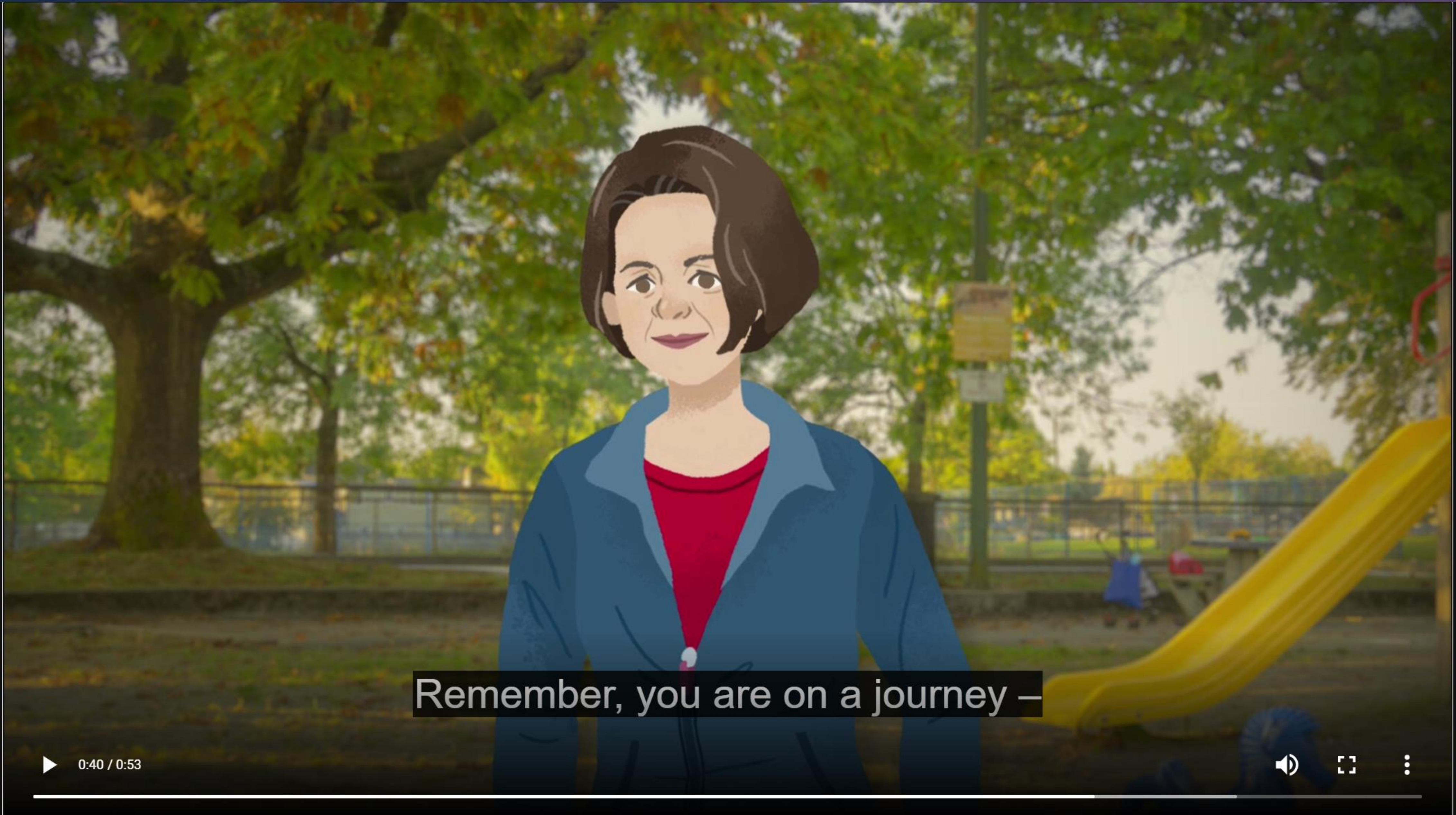
Remember, you are on a journey –
0:40 / 0:53

About Us FAQ / Resources

OUTSIDEPLAY.ca

Take a risk, go play outside!

Journey Map EN | FR

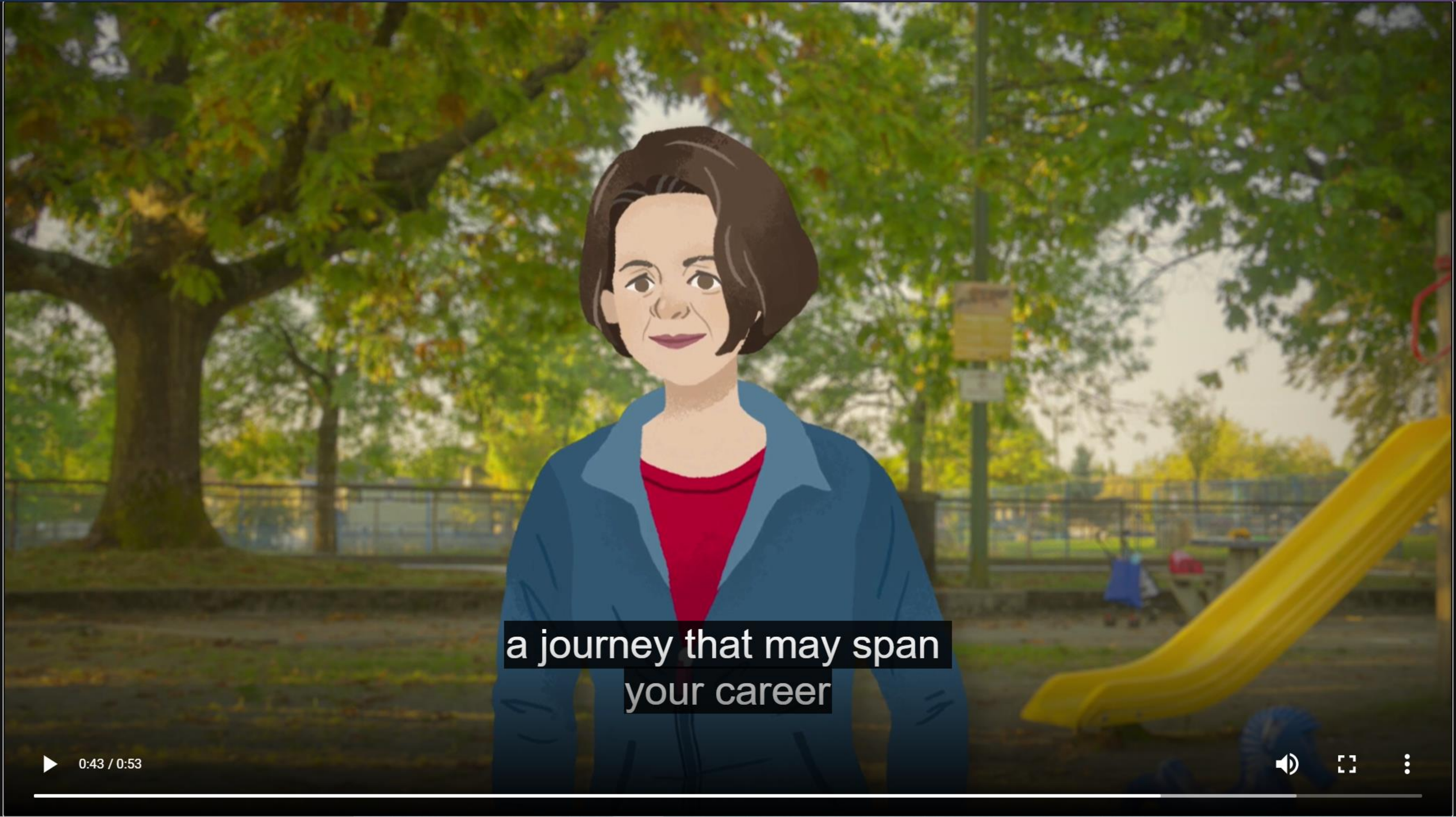

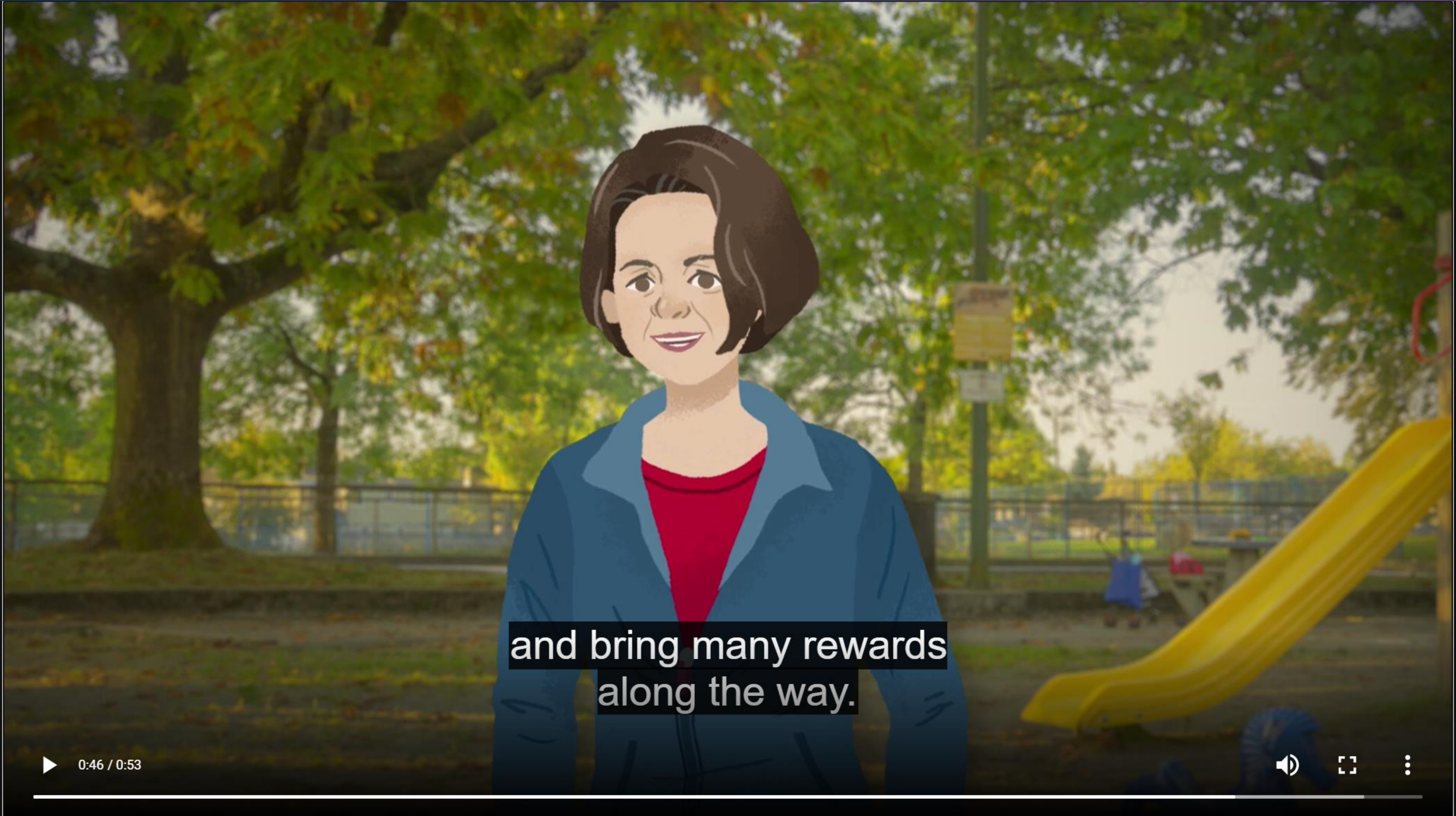
and bring many rewards
along the way.
0:46 / 0:53

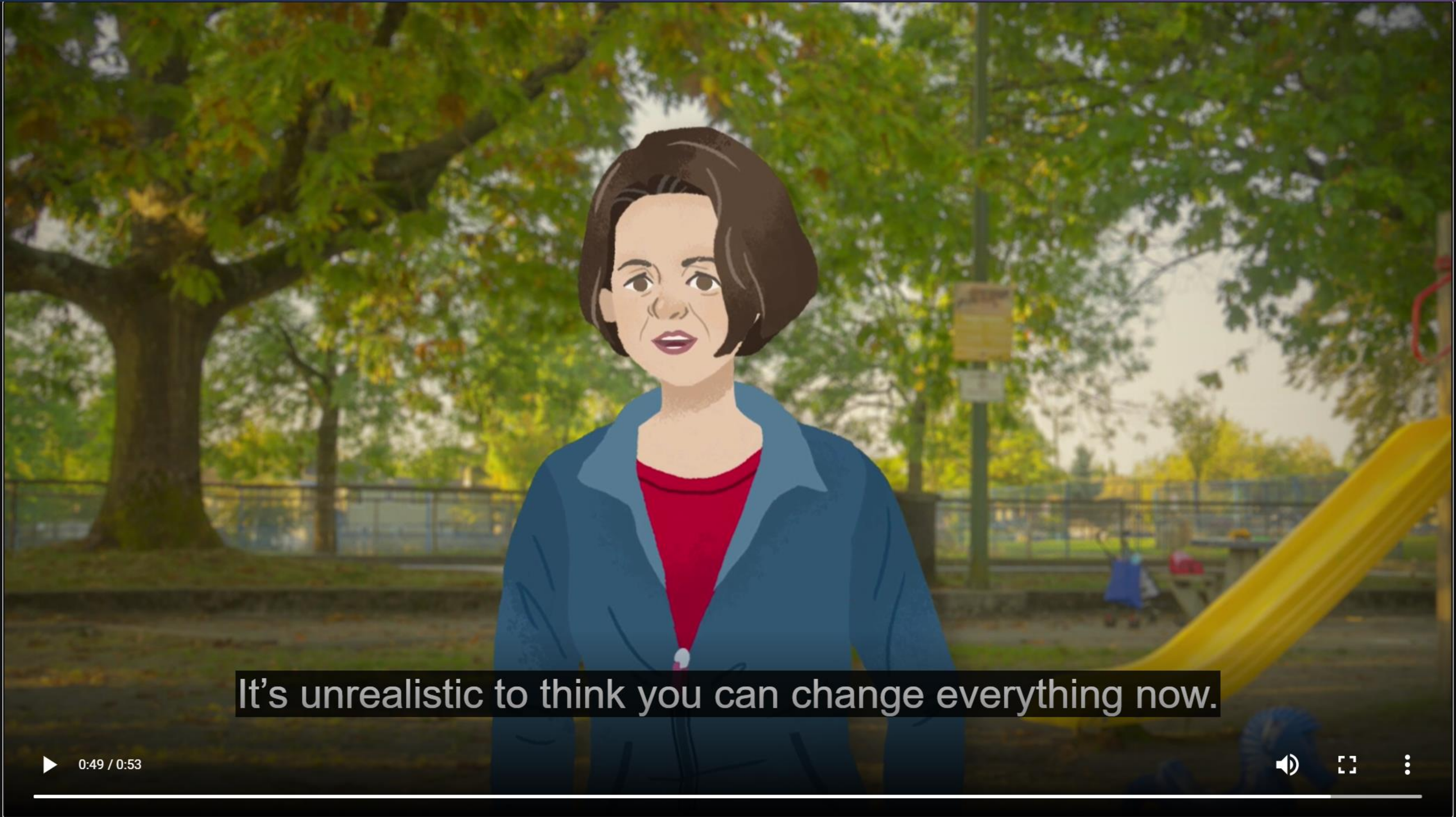
It’s unrealistic to think you can change everything now.
0:49 / 0:53

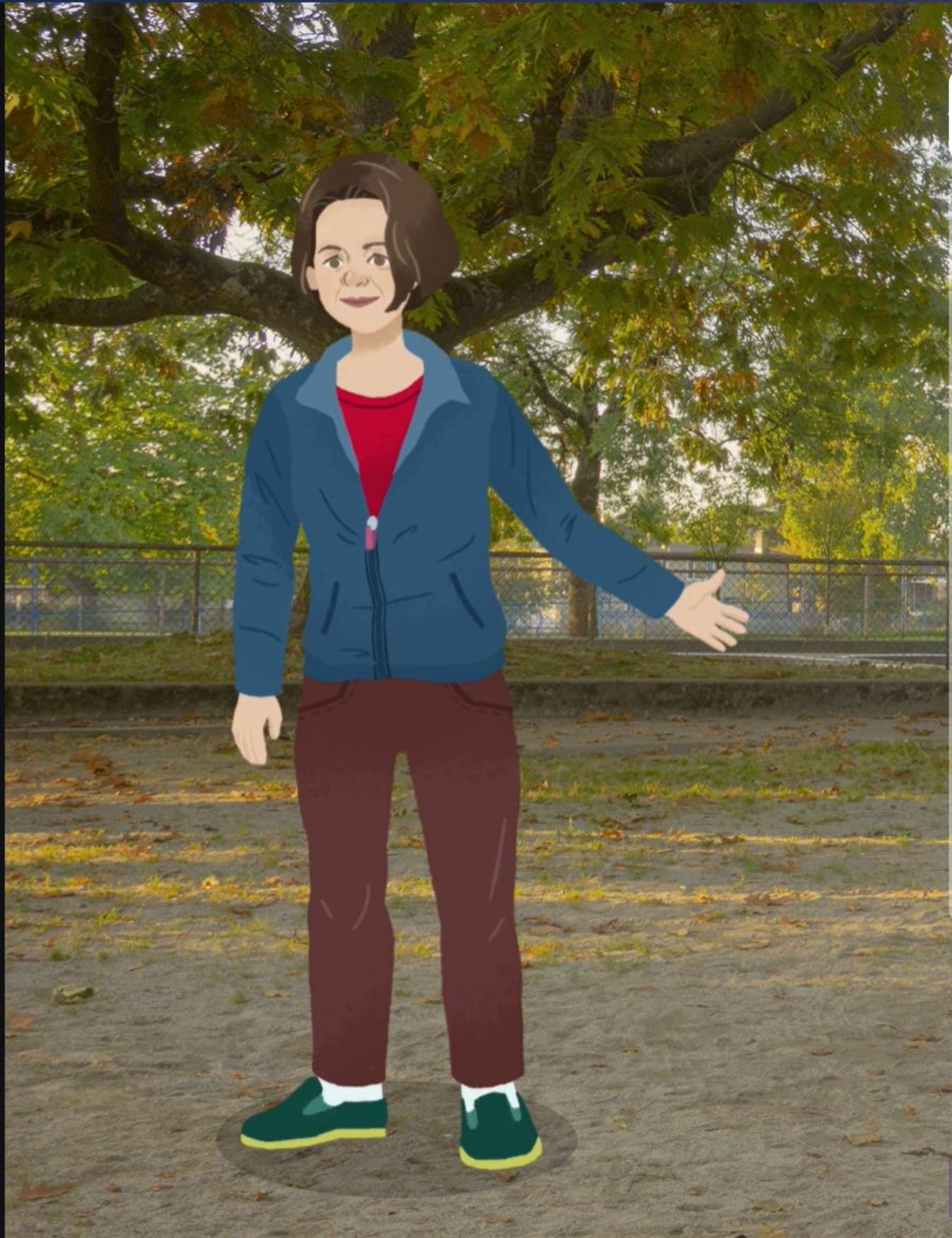

What is ONE thing you can do to support children's outdoor play?

My one thing is...

Here are some examples if you get stuck

- Get clear on my "why" and what it means for my practice.
- Take deep breaths and count to 17 next time I'm nervous about the children's play.
- Start the journey toward developing a play charter/policy on outdoor play for my centre.
- Collect at least five different loose parts for each child at my centre.
- Include outdoor play as an agenda item at all of our staff meetings (to help colleagues understand importance, to develop policy, to support each other, and, to discuss emerging challenges).
- Talk to parents/caregivers/licensing officer about outdoor play and why it is important.
- Start to document children's learning to share with parents/co-workers.

Next

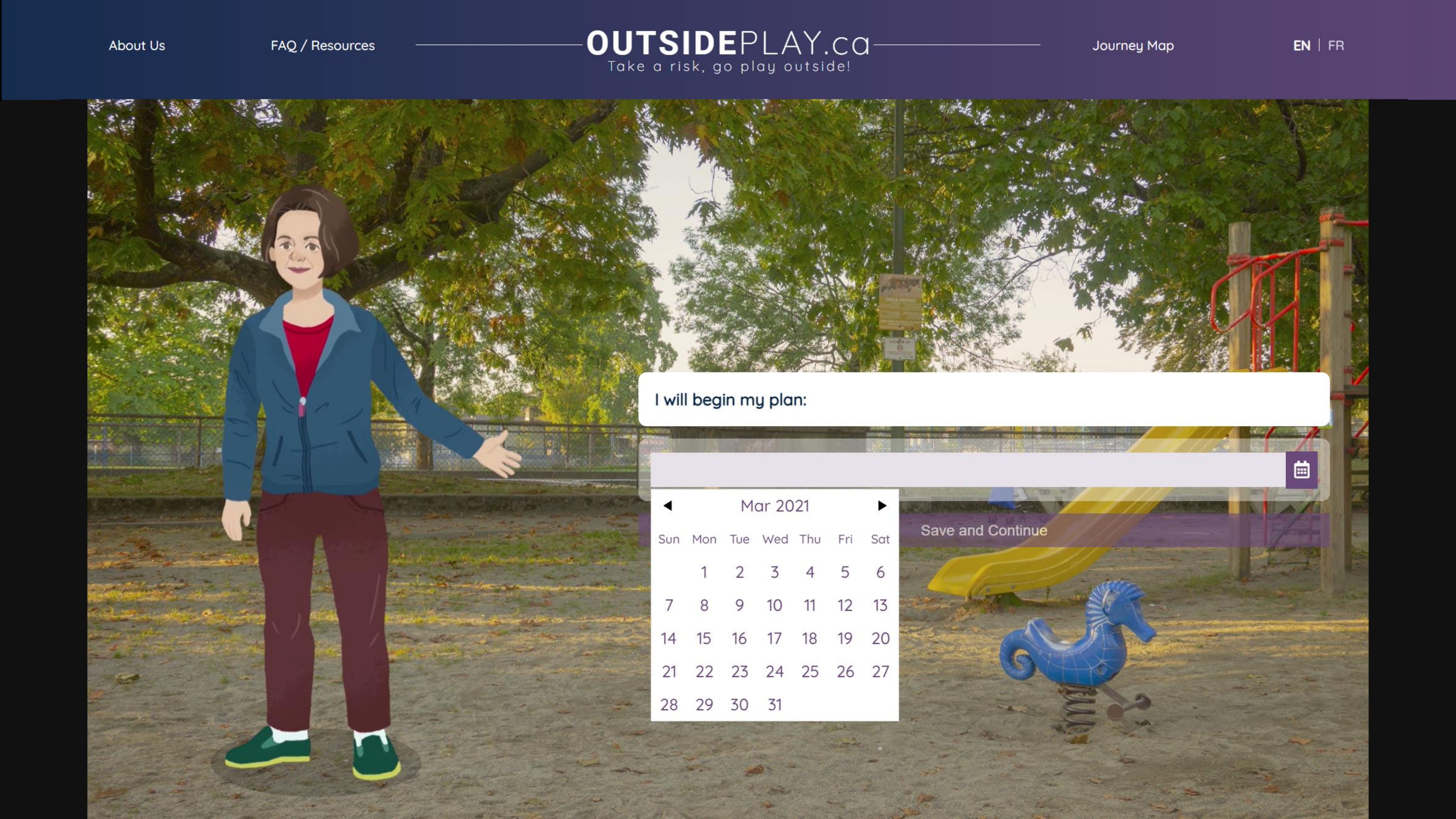
About Us
FAQ / Resources
OUTSIDEPLAY.ca
Take a risk, go play outside!
Journey Map
EN | FR
I will begin my plan:
Mar 2021
Sun Mon Tue Wed Thu Fri Sat
1 2 3 4 5 6
7 8 9 10 11 12 13
14 15 16 17 18 19 20
21 22 23 24 25 26 27
28 29 30 31
Save and Continue

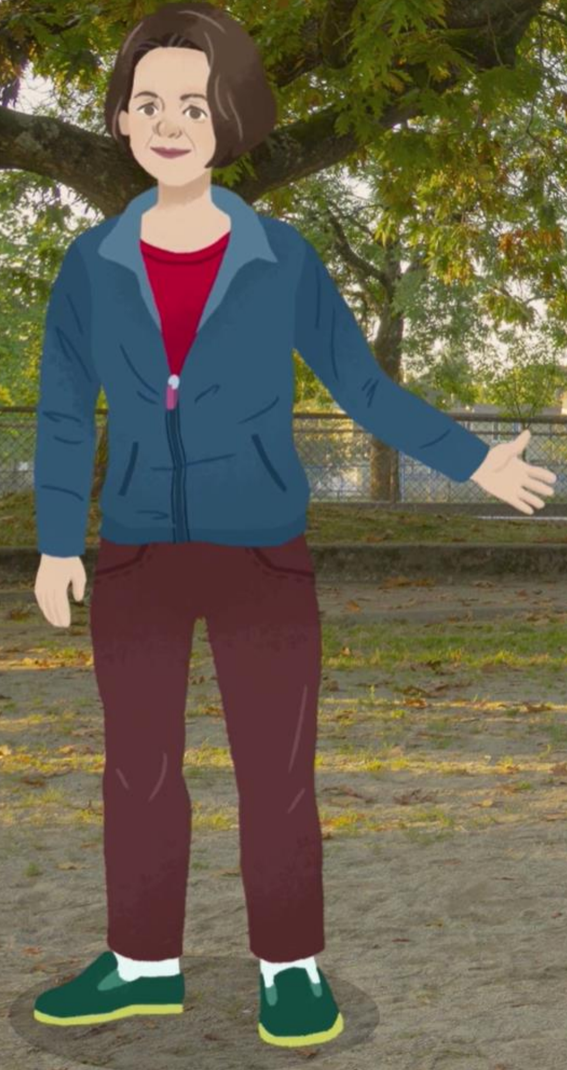

ACTION PLAN COMPLETE!

See Journey Map

Resources

Save Action Plan

Download Certificate
